# Human and preclinical studies of the host–gut microbiome co-metabolite hippurate as a marker and mediator of metabolic health

**DOI:** 10.1136/gutjnl-2020-323314

**Published:** 2021-05-11

**Authors:** François Brial, Julien Chilloux, Trine Nielsen, Sara Vieira-Silva, Gwen Falony, Petros Andrikopoulos, Michael Olanipekun, Lesley Hoyles, Fatima Djouadi, Ana L Neves, Andrea Rodriguez-Martinez, Ghiwa Ishac Mouawad, Nicolas Pons, Sofia Forslund, Emmanuelle Le-chatelier, Aurélie Le Lay, Jeremy Nicholson, Torben Hansen, Tuulia Hyötyläinen, Karine Clément, Matej Oresic, Peer Bork, Stanislav Dusko Ehrlich, Jeroen Raes, Oluf Borbye Pedersen, Dominique Gauguier, Marc-Emmanuel Dumas

**Affiliations:** 1 UMRS 1124 INSERM, Université de Paris Descartes, Paris, France; 2 Section of Biomolecular Medicine, Department of Metabolism, Digestion and Reproduction, Imperial College London, London, UK; 3 Novo Nordisk Foundation Centre for Basic Metabolic Research, University of Copenhagen, Kobenhavn, Denmark; 4 Laboratory of Molecular Bacteriology, Department of Microbiology and Immunology, Rega Institute for Medical Research, Katholieke Universiteit Leuven, Leuven, Belgium; 5 National Heart & Lung Institute, Section of Genomic & Environmental Medicine, Imperial College London, London, UK; 6 Department of Biosciences, Nottingham Trent University, Nottingham, UK; 7 Centre de Recherche des Cordeliers, Université Paris Descartes, Paris, France; 8 Centre de Recherche des Cordeliers, INSERM, Sorbonne Université, Paris, France; 9 Metagenopolis, INRAE, Paris, Île-de-France, France; 10 Forslund Lab, Max Delbrück Centrum für Molekulare Medizin Experimental and Clinical Research Center, Berlin, Berlin, Germany; 11 Department of Chemistry, Örebro University, Örebro, Sweden; 12 INSERM, U1166, team 6 Nutriomique, Université Pierre et Marie Curie-Paris 6, Paris, France; 13 Institute of Cardiometabolism and Nutrition (ICAN), Assistance Publique-Hôpitaux de Paris, Pitié-Salpêtrière Hospital, Paris, France; 14 School of Medical Sciences, Örebro Universitet, Orebro, Sweden; 15 Structural and Computational Biology Unit, European Molecular Biology Laboratory, Heidelberg, Germany; 16 Center for Host Microbiome Interactions, King's College London Dental Institute, London, UK; 17 Center for Microbiology, Vlaams Instituut voor Biotechnologie, Leuven, Belgium; 18 McGill Genome Centre & Department of Human Genetics, McGill University, Montréal, Québec, Canada; 19 European Genomics Institute for Diabetes, INSERM U1283, CNRS UMR8199, Institut Pasteur de Lille, Lille University Hospital, Unversity of Lille, Lille, France

**Keywords:** intestinal microbiology, glucose metabolism, obesity, colonic microflora

## Abstract

**Objective:**

Gut microbial products are involved in regulation of host metabolism. In human and experimental studies, we explored the potential role of hippurate, a hepatic phase 2 conjugation product of microbial benzoate, as a marker and mediator of metabolic health.

**Design:**

In 271 middle-aged non-diabetic Danish individuals, who were stratified on habitual dietary intake, we applied ^1^H-nuclear magnetic resonance (NMR) spectroscopy of urine samples and shotgun-sequencing-based metagenomics of the gut microbiome to explore links between the urine level of hippurate, measures of the gut microbiome, dietary fat and markers of metabolic health. In mechanistic experiments with chronic subcutaneous infusion of hippurate to high-fat-diet-fed obese mice, we tested for causality between hippurate and metabolic phenotypes.

**Results:**

In the human study, we showed that urine hippurate positively associates with microbial gene richness and functional modules for microbial benzoate biosynthetic pathways, one of which is less prevalent in the *Bacteroides* 2 enterotype compared with Ruminococcaceae or *Prevotella* enterotypes. Through dietary stratification, we identify a subset of study participants consuming a diet rich in saturated fat in which urine hippurate concentration, independently of gene richness, accounts for links with metabolic health. In the high-fat-fed mice experiments, we demonstrate causality through chronic infusion of hippurate (20 nmol/day) resulting in improved glucose tolerance and enhanced insulin secretion.

**Conclusion:**

Our human and experimental studies show that a high urine hippurate concentration is a general marker of metabolic health, and in the context of obesity induced by high-fat diets, hippurate contributes to metabolic improvements, highlighting its potential as a mediator of metabolic health.

**Significance of the study**

**What is already known about this subject?**

- Previous reports have demonstrated the role of the microbiome in obesity, non-alcoholic fatty liver disease, insulin resistance and type 2 diabetes
- A microbial-host co-metabolite, hippurate, has been associated with health in studies with fatty liver disease, insulin resistance, diabetes, obesity and metabolic syndrome.

**What are the new findings?**

- Hippurate showed the strongest association with microbial gene richness and microbial genes associated with hippurate belong to the phenylpropanoid pathway
- High hippurate levels are associated with metabolic health in volunteers consuming a high-meat diet rich in saturated fats
- Chronic pharmacological treatments with hippurate provide metabolic benefits in high-fat diet contexts
- Hippurate also specifically increases β cell area in pancreas and function in high-fat diet conditions.

**How might it impact on clinical practice in the foreseeable future?**

- Hippurate can be used a marker of metabolic health in stratified studies and its levels can be monitored in lifestyle interventions
- Specific dietary advice could eventually be given to increase hippurate production potential by the microbiome

## Introduction

Human obesity is an epidemic that raises the risk of type 2 diabetes and cardiovascular disease. Gut microbiome dysbiosis is now recognised as a key feature of these disorders. The gut microbiota is a complex ecosystem, harbouring thousands of microbial species and strains.^1^ It is a dynamic system described as a continuum between core and rare participants,^2^ with an overall ecosystem structure alternately described in terms of gradients^3^ or enterotypes across populations.^4–6^ The microbiome collectively encodes up to 10 million different microbial genes.^7 8^ Microbial gene richness has been proposed as a marker of ecological diversity mirroring improvements in metabolic health.^9 10^ Many factors affect the gut microbiota,^11^ including diet,^12 13^ age,^14 15^ lifestyle,^16^ dietary supplements such as sweeteners^17^ and drugs.^16 18^ Although the microbiota directly impacts various biological processes of the host through production or degradation of a multitude of compounds, the vast majority of molecules involved in this chemical crosstalk remain elusive.^19–22^

Hippurate is one of the most abundant microbial–host co-metabolites, produced by conjugation from glycine and microbial benzoate in the liver and kidney through phase 2 detoxification.^23^ Hippurate has been shown to be associated positively with microbial diversity but negatively with blood pressure, non-alcoholic fatty liver disease, visceral fat mass and Crohn’s disease.^24–28^ This suggests the potential role of hippurate in metabolic health.

Despite recent progress, there is a critical need for an in-depth characterisation of the complex nutrition–microbiome–host interaction involving the hippurate pathway, in particular related to (1) associations with microbial gene richness, biosynthetic gene modules and their harbouring enterotypes; (2) population stratification to identify patient subgroups in which hippurate improves metabolic health; and (3) biological characterisation of the effect of hippurate on host phenotypes.

To address these points, we characterised the urinary metabolome and faecal metagenome of 271 middle-aged non-diabetic participants from the Metagenomics of the Human Intestinal Tract (MetaHIT) study in the context of a broad range of body weight, immune and metabolic markers as well as habitual dietary intake data.^9^ We evaluated the interplay between diet, microbiome and metabolome in general and for the hippurate pathway in particular. We show that hippurate beneficially impacts on bioclinical phenotypes, which we further confirm through in vivo studies in a preclinical model of obesity and diabetes.

## Methods

### Human subjects

All analyses were done on non-diabetic Danish individuals from the MetaHIT study (N=271),^9 29^ including the subset of 193 individuals who completed a validated Food Frequency Questionnaire (FFQ).^18^ The study was approved by the Ethical Committees of the Capital Region of Denmark (HC-2008-017 and H-15000306) and was in accordance with the principles of the Declaration of Helsinki. All participants gave written informed consent. Sampling and clinical phenotyping were performed as described previously.^9 29^ Briefly, all study participants were recruited from the population-based Inter99 study.^30^ The study programme consisted of two visits, approximately 14 days apart. At the first visit, all participants were examined in the morning after an overnight fast. At the second visit, a dual-energy X-ray absorptiometry scan was performed. Serum glycine levels were previously assessed.^29^ Estimated glomerular filtration rate (eGFR) was calculated with the chronic kidney disease - epidemiology collaboration (CKD-EPI) formula without the ethnicity factor.^31^

### Dietary data

A subset of the study participants (n=193) completed a validated FFQ in order to obtain information on their habitual diet.^32^ The FFQ gathered dietary information from all meals during a day and recorded the intake frequencies within the past months. The consumed quantity was determined by multiplying the portion size with the reported consumption frequency in the FFQ. Standard portion sizes for women and men, separately, were used in this calculation; all food items in the FFQ were linked to food items in the Danish Food Composition Databank as previously described.^18^ Estimation of daily intake of macronutrients and micronutrients for each participant was based on calculations using FoodCalc (V.1.3) (http://www.ibt.ku.dk/jesper/FoodCalc/Default.htm).

### Sample collection

Faecal samples were collected at home by the study participants and immediately frozen after collection. The samples were transferred to the research centre using insulating polystyrene foam containers and stored at −80°C until analysis. DNA extraction was performed as described.^9^ Blood sampling was performed at the fasting state, and urine was collected at the first visit mid-void on arrival at the study centre and frozen immediately with no preservatives added. Samples were stored at −80°C until analyses.

### Metabolic profiling

Urine samples were randomised, prepared and measured on a ^1^H-NMR spectrometer (Bruker GmbH) operating at 600.22 MHz following Bruker IVDr standard operating procedures (SOPs) as described.^33^ Briefly, 540 µL urine was mixed with 60 µL buffer (1.5 M NaH_2_PO_4_, 0.1% v/v TSP, 2 mM NaN_3_ in D_2_O, pH7.4), vortexed and centrifuged at 12 000g for 5 min at 4°C. Then, 550 µL of the resulting supernatant was transferred into a 5 mm SampleJet NMR tube for ^1^H-NMR analysis. The ^1^H-NMR spectra were imported into Matlab for preprocessing as reported^24^ using Probabilistic Quotient Normalisation (PQN)^34^ followed by peak picking with the Statistical Recoupling of Variables (SRV) algorithm.^35^ Structural assignment was performed as reviewed^36^ using in-house and publicly available databases. The hippurate peaks at 7.84(d), 7.55(t) and 7.64(t) ppm were integrated manually and summed.

### Metagenomics

Shotgun sequencing of microbial DNA and metagenomics processing workflow for gene richness were performed as published.^9^ Sequences were mapped onto the previously released integrated gene catalogue.^7^ Following the strategy published in Vieira-Silva *et al,* 37 we built a novel set of 20 manually curated gut-specific metabolic modules to map microbial phenylpropanoid metabolism from metagenomic data (online supplemental data 1). Assembly of the module set was based on extensive literature and database review (KEGG,^38^ MetaCyc^39^). Included pathways were restricted to prokaryote metabolism of phenylpropanoids and related substrates. While the scope of the current module sets exceeds microbial benzoate production, it does not claim completeness regarding coverage of microbial phenylpropanoid metabolism. Each module represents a cellular enzymatic process, defined as a set of ortholog groups and delimited by input and output metabolites. Module structure follows the KEGG database syntax. Abundances of customised modules were derived from the ortholog abundance tables using Omixer-RPMV.1.0 (https://github.com/raeslab/omixer-rpm).^40^ Enterotyping of the genus-level abundance microbial profiles with Hellinger transformation was performed based on the Dirichlet multinomial mixtures (DMM) approach implemented in R package DirichletMultinomial, as described.^41^

10.1136/gutjnl-2020-323314.supp1 Supplementary data

### Univariate statistical analysis

Outliers were identified by ROUT test (Q=1%) in GraphPad (Prism). For comparisons between groups, normality was tested using D'Agostino-Pearson omnibus normality test, then one-way analysis of variance, followed by Tukey’s honestly significant difference (HSD) post hoc testing when data were normally distributed; otherwise, groups were compared using the two-tailed Mann-Whitney test (p<0.05 considered to be statistically significant). Data were displayed as mean±SEM throughout. Multiple testing corrections were performed using Storey’s procedure (noted q).^42^ For comparison between more than two groups, a two-tailed Kruskal-Wallis test was applied (with multiple correction using the Benjamini-Hochberg method, noted p adjusted for false discovery rate, pFDR) followed by a joint-rank Dunn test for pairwise comparisons between groups.

### Principal component analysis (PCA)

PCA was performed using MATLAB V.R2014a function ‘ppca’ in order to run a probabilistic PCA to be able to include variables with a minority of missing values.

### Principal coordinates analysis (PCoA)

Unconstrained ordination was performed to visualise interindividual variation in microbiota composition, using Bray-Curtis dissimilarity on the genus-level abundance matrix with the vegan (V.2.4-1) R package.^43^

### Least-squares and stepwise multivariate linear regressions

The cumulative contributions of explanatory variables on excreted hippurate were determined by multivariate linear regressions (with or without stepwise feature selection). Hippurate and the continuous explanatory variables were rank-transformed and model explanatory power was assessed using the Akaike information criterion.

### Orthogonal partial least squares (O-PLS)

Orthogonal partial least squares discriminant analysis (O-PLS-DA) was performed in MATLAB V.R2014a for supervised multivariate analysis as described.^44^ The predictive capability of O-PLS-DA models was evaluated through sevenfold cross-validation^44^ to compute Q^2^_Yhat_ goodness-of-prediction parameters. The empirical significance of the Q^2^_Yhat_ parameter was evaluated by random permutation testing (10 000 iterations).^45^

### K-means clustering

Optimal number of clusters were determined using the elbow, silhouette and gap statistics methods by majority vote from the R (4.0.3) packages factoextra (1.0.7) and cluster (2.1.0). Clustering was performed using the built-in R K-means function with the Hartigan-Wong algorithm using 25 random sets.

### Animal experiments

All animal procedures were authorised by the ethics committee of the University of Paris (Ref: 00486.02). Four groups of six C57BL/6J male mice (Janvier Labs) were fed either control carbohydrate diet (CHD; 10% fat) (D12450Ki, Research diets) or high-fat diet (HFD; 60% fat) (D12492i, Research diets) as illustrated in online supplemental figure 1. The composition of each diet is given in online supplemental data 2. Hippurate (5.55 mM in 0.9% NaCl) was administered subcutaneously for 6 weeks using Alzet minipumps (model 2006, Charles River); control mice were infused with saline. Procedures and assays were as described.^46^ Briefly, glucose tolerance and insulin secretion were assessed using an intraperitoneal glucose tolerance test (IPGTT). Blood samples were collected before injection of glucose (2 g/kg) and sequentially afterwards to determine glycaemia using a glucometer (Roche Diagnostics) and insulin using an ELISA (Mercodia). Pancreas sections were incubated with an insulin-specific antibody (Cat #: C27C9, Ozyme) or an anti-Ki67 antibody (Cat #:ab15580, Abcam) followed by an horseradish peroxidase (HRP)-conjugated secondary antibody (Cat #:1706516, Bio-Rad). Digital images were analysed using Visiopharm Integrator System (Visiopharm), and quantitative analysis was carried out using a positive pixels algorithm (Indica Labs).

10.1136/gutjnl-2020-323314.supp2 Supplementary data

10.1136/gutjnl-2020-323314.supp3 Supplementary data

## Results

### Hippurate is the urine metabolite most strongly associated with faecal microbial gene richness

**Figure 1:**
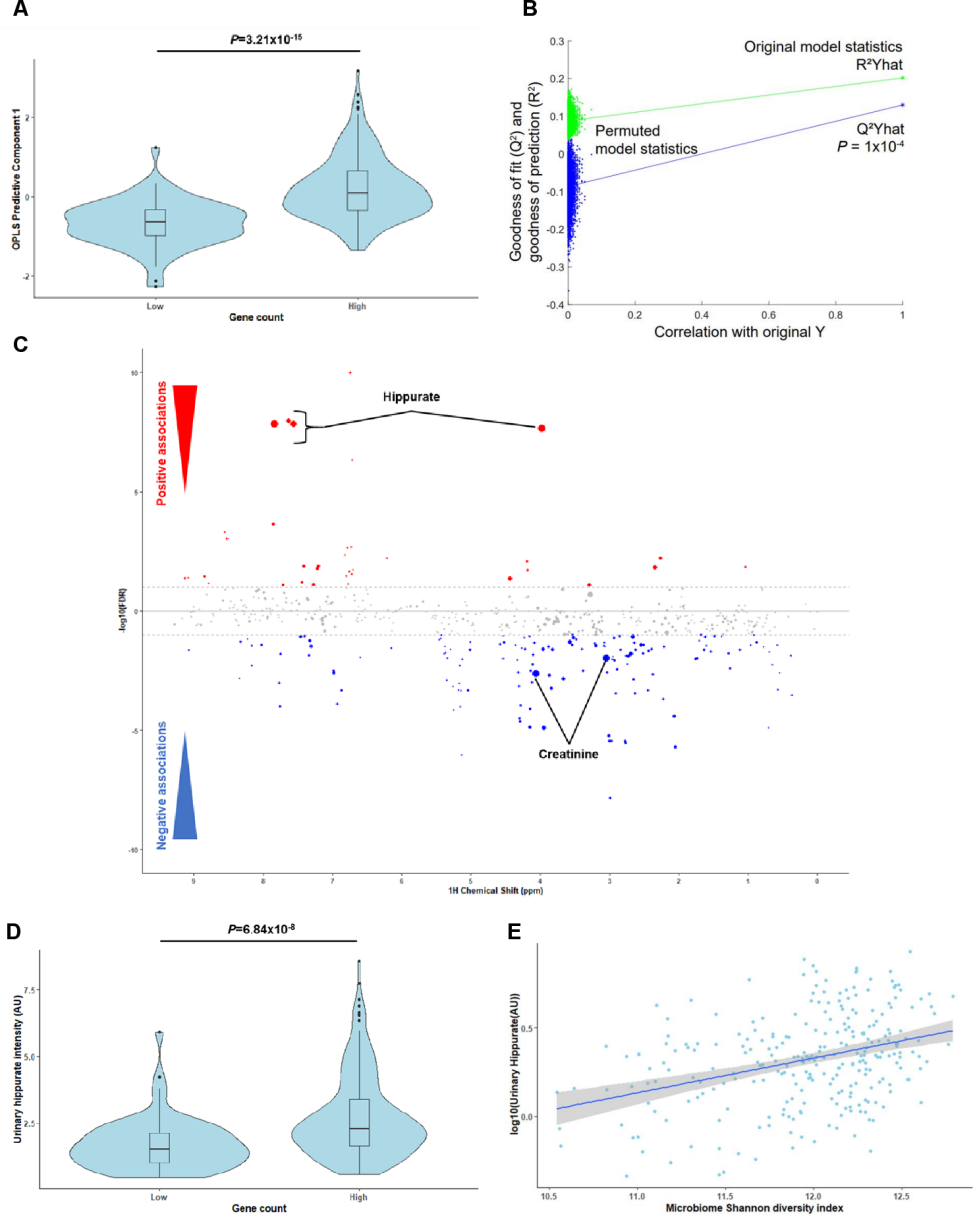
Hippurate is the main metabolite correlated with gene richness and functional redundancy of the gut microbiome. (A) Scores plot (predictive component 1) obtained for an orthogonal partial least squares discriminant analysis (O-PLS-DA) model fitted using urinary ^1^H-NMR spectra to predict microbial gene richness, showing a significant association between high gene richness (over 480 000 gene counts) and ^1^H-NMR spectra (p=3.21×10^-15^ for a significantly non-zero slope using F-test, N=271). (B) Empirical assessment of the significance of O-PLS goodness-of-fit parameter Q^2^_Yhat_ by generating a null distribution with 10 000 random permutations (p=1.00×10^-4^). (C) Manhattan plot highlighting associations between ^1^H-NMR variables and gene count displayed in a pseudo-spectrum layout. A negative value (blue circles) means a negative correlation, while a positive value (red circles) means a positive correlation. Grey circles are clusters with a p value >0.01. Size of circles represents the covariance of the cluster with the gene count. (D) Association between urinary hippurate intensity (area under the curve of the hippurate ^1^H-NMR peaks; AU) and high gene counts (over 480 000; p=6.84×10^-8^ for a significantly non-zero slope using F-test). (E) Linear-regression-based scatterplot showing correlation between urinary hippurate (AU: log-transformed for visualisation purposes) and Shannon microbial diversity index (Spearman’s rho^2^=0.108, p=2.82×10^-8^; N=271).

To identify microbial and host compounds mediating beneficial effects in metabolic health, we profiled the urinary metabolome of the MetaHIT population^9^ using ^1^H-NMR to perform a Metabolome-Wide Association Study (MWAS)^25^ for microbial gene richness.^9^ We first built an O-PLS-DA model based on the ^1^H-NMR spectra to stratify the population by gene richness using our previously published cut-off of 480 000 microbial genes^9^ (figure 1A, p=3.21×10^-15^). A gene counts density plot supported the validity of the stratification (online supplemental figure 2A). The cross-validated model significantly predicted variance associated with gene richness through a permutation test (figure 1B, p=0.1×10^-4^, 10 000 randomisations). Model coefficients for this discrimination revealed hippurate as having the strongest association with high microbial gene counts and creatinine with low gene counts (figure 1C). Individuals with low microbial gene richness present significantly lower urinary hippurate levels than those with high (figure 1D, online supplemental figure 2B; rank-based Spearman’s correlation rho^2^=0.173, p=1.99×10^-9^). These data support the association between hippurate levels, gene richness and Shannon’s diversity index^23^ (figure 1E, rho^2^=0.108, p=2.82×10^-8^).

10.1136/gutjnl-2020-323314.supp4 Supplementary data

Importantly, urinary hippurate levels showed significant negative associations with markers for metabolic impairments such as body mass index (BMI), body weight, the homeostasis model assessment of insulin resistance (HOMA-IR), interleukin-6 (IL-6), insulin and C-peptide (partial Spearman’s correlations, q<0.1, online supplemental figure 2C). Urinary hippurate levels did not associate with either serum glycine (n=269, linear Pearson’s correlation, r=0.06, p=0.30), which is required for hippurate synthesis through conjugation with gut microbial benzoate,^47^ or eGFR (r=−0.10, p=0.11), which could limit hippurate clearance, or to a combined effect of both variables (r=−0.02×10^-2^, p=0.84; online supplemental figure 2D, E, online supplemental table 1). Two representative annotated ^1^H-NMR spectra for individuals with low or high gene counts are shown (online supplemental figure 3, online supplemental table 2). We next used K-means clustering for data-driven stratification of urinary hippurate into ‘high’ and ‘low’ (online supplemental figure 4A). Individuals in the ‘high’ hippurate cluster exhibited higher hippurate levels (p<2.16×10^-16^, online supplemental figure 4B) and gene counts (p=2.00×10^-5^, online supplemental figure 4C). Moreover, insulin resistance (HOMA-IR) was significantly lower in obese subjects (BMI>25) with higher levels of hippurate excretion (p=0.0019, online supplemental figure 4D), which, however, was not significant in lean subjects. We next derived absolute hippurate quantifications using the Bruker IVDr algorithm.^48^ Absolute hippurate values highly correlated with hippurate (AU) (rho^2^=0.76, p<2.2×10^-16^), individuals with high gene counts secreted higher absolute hippurate (p=0.00015) and absolute hippurate significantly correlated with gene counts (rho^2^=0.11, p=7.17×10^-8^; online supplemental figure 5A–C). Using the hippurate stratification in online supplemental figure 4A for absolute hippurate values, we report that the cut-off value for secreted hippurate is 3.94 mM (online supplemental figure 5D). To further validate our results, we normalised our ^1^H-NMR peaks with the corresponding creatinine values. Again, scores obtained from an O-PLS-DA model built on creatinine-corrected ^1^H-NMR peaks significantly associated with gene richness (p=8.12×10^-14^), predicted gene counts (p=1×10^-4^; 10 000 permutations) and creatinine-adjusted hippurate had the strongest covariance with high gene counts (online supplemental figure 6A–C). Moreover, individuals with high gene counts secreted elevated creatinine-adjusted hippurate (p=5.38×10^-9^) and creatinine-adjusted hippurate correlated with gene richness (rho^2^=0.18, p=5.48×10^-13^) and Shannon diversity (rho^2^=0.12, p=6.01×10^-9^, online supplemental figure 6D–F).

10.1136/gutjnl-2020-323314.supp5 Supplementary data

10.1136/gutjnl-2020-323314.supp6 Supplementary data

10.1136/gutjnl-2020-323314.supp7 Supplementary data

10.1136/gutjnl-2020-323314.supp8 Supplementary data

10.1136/gutjnl-2020-323314.supp9 Supplementary data

10.1136/gutjnl-2020-323314.supp10 Supplementary data

### Gut microbiome determinants of hippurate production in the phenylpropanoid pathway

**Figure 2:**
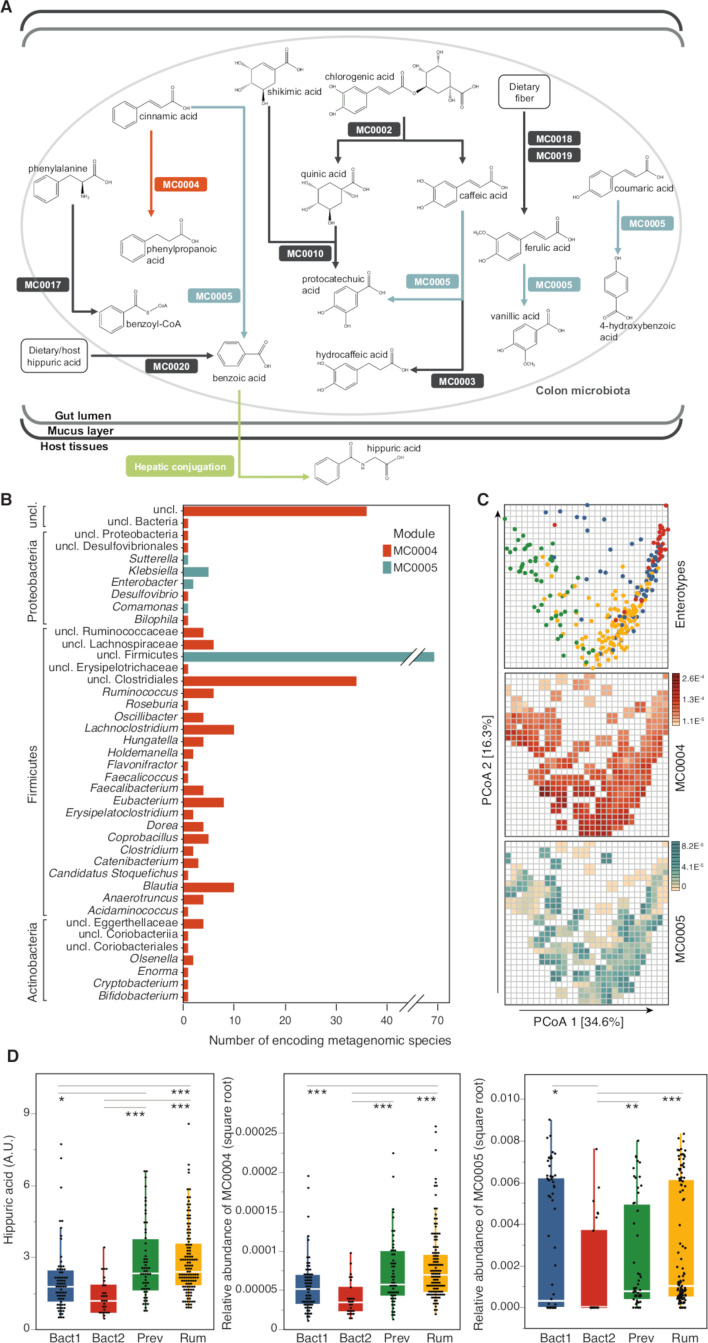
Detection of microbial phenylpropanoid metabolism-related modules in faecal metagenomes of healthy volunteers and their associations with urine hippurate concentrations. (A) Visualisation of gut-specific metabolic modules (GMMs) encoding phenylpropanoid metabolism-related pathways detected in more than 20% of individuals; MC0004 (orange; N=271, Spearman’s rho=0.19, q-value=0.006) and MC0005 (blue; N=271, Spearman’s rho=0.21, q-value=0.006) relative abundances correlate positively with urine hippurate concentrations (online supplemental table 4). (B) Metagenomic species encoding modules MC0004 and MC0005. (C) (Top panel) Faecal microbiomes dissimilarity visualised on the first plane of the genus-level principal coordinates analysis (PCoA, Bray-Curtis dissimilarity), with individual samples coloured according to enterotypes (Bacteroides1 (Bact1), blue; Bacteroides2 (Bact2), red; Prevotella (Prev), green; Ruminococcaceae (Rum), yellow). (Middle and bottom panels) Same genus-level PCoA overlaid with a mesh coloured according to the median abundances of GMMs MC0004 (red) and MC0005 (blue) in samples falling within each cell of the mesh (N=271). MC0005 relative abundance was transformed for clearer visualisation (square root). (D) Distribution of urine hippurate concentrations (N=271, Kruskal-Wallis, χ^2^=41.78, q-value=4.45×10^-9^; (left panel) and MC0004 (N=271, Kruskal-Wallis, χ^2^=40.04, q-value=1.05×10^-8^; (middle panel)) and MC0005 (N=271, Kruskal-Wallis, χ^2^=22.25, q-value=5.79×10^-5^; (right panel)) relative abundances over enterotypes. Significance levels of post hoc Dunn test corrected for multiple testing are indicated (q-value <0.05 (*); <0.01 (**); <0.0001 (***); online supplemental tables 7 and 8). The body of the boxplot represents the first and third quartiles of the distribution, with the median line, and the whiskers extend from the quartiles to the last data point within 1.5×IQR, with outliers beyond.

To characterise microbiome determinants of benzoate production (the microbial precursor of hippurate), we next used faecal metagenomic data. We functionally annotated functions of the Integrated Gene Catalogue (IGC) to KEGG Orthology (KO) groups and found 2733 KEGG modules positively associated with urine hippurate (pFDR <0.05; online supplemental table 3). We then manually curated 20 metabolic modules covering microbial phenylpropanoid metabolism, including benzoate production (referenced in online supplemental data 1). Each module represents an enzymatic reaction, defined as a set of ortholog groups and delimited by input and output metabolites. Proportional abundances of only two modules were correlated with urine hippurate levels (at prevalence (number of subject with the pathway present/total number of subjects)>20%): cinnamate conversion, leading to the production of phenylpropanoate (MC0004; N=271, prevalence=100%, Spearman’s rho=0.19, q-value=0.006) and coumarate degradation, also encoding degradation of cinnamate to benzoate (MC0005; N=271, prevalence=74%, Spearman’s rho=0.21, q-value=0.006; figure 2A; online supplemental figure 7, online supplemental table 4). Interestingly, while MC0004 and MC0005 are competing for cinnamate as a common precursor, they both positively associated with urinary hippurate—despite the fact that the MC0004 metabolism would not result in increased benzoate production. Both modules additionally correlated with microbial gene richness (MC0004; N=271, rho=0.63, q-value=8.35×10^-31^; MC0005; N=271, rho=0.34, q-value=4.48×10^-8^; online supplemental table 5). Metagenomic species encoding MC0004 orthologs were predominantly found in genomes of *Firmicutes*, *Actinobacteria* and, to a lesser extent, *Proteobacteria* (figure 2B; online supplemental tables 6 and 7). Distribution of MC0005 was limited to (unclassified) *Firmicutes* and *Proteobacteria* (figure 2B), with both modules undetected in genera belonging to the predominant *Bacteroidetes* phylum.

10.1136/gutjnl-2020-323314.supp11 Supplementary data

10.1136/gutjnl-2020-323314.supp12 Supplementary data

10.1136/gutjnl-2020-323314.supp13 Supplementary data

10.1136/gutjnl-2020-323314.supp14 Supplementary data

10.1136/gutjnl-2020-323314.supp15 Supplementary data

10.1136/gutjnl-2020-323314.supp16 Supplementary data

10.1136/gutjnl-2020-323314.supp17 Supplementary data

Mapping of relative abundances on an enteroscape (first plane of a normalised genus-level PCoA based on Bray-Curtis dissimilarity^38^; figure 2C) revealed a gradient of module distribution across microbiome community types. Prior research identified a potentially dysbiotic microbiome community characterised by inflammation, low microbial density, gene richness and Bact2 enterotype.^6 49 50^ We applied the same strategy, that is, DMM on genus-level abundance profiles,^41^ to cluster faecal microbiomes into four enterotypes (Ruminococcaceae, Rum; *Bacteroides1*, Bact1; *Bacteroides2*, Bact2; *Prevotella*, Prev). Urinary hippurate (N=271, Kruskal-Wallis, χ^2^=41.78, q-value=4.45×10^-9^), MC0004 (χ^2^=40.04, q-value=1.05×10^-8^) and MC0005 (χ^2^=22.25, q-value=5.79×10^-5^) relative abundances were unevenly distributed over enterotypes, with Bact1 and Bact 2 carriers displaying lower hippurate excretion levels than their Rum and Prev counterparts (figure 2D; online supplemental tables 8 and 9). These results suggest that microbial modules and community structures could potentially (co-)determine the abundance of hippurate in the host.

10.1136/gutjnl-2020-323314.supp18 Supplementary data

### Urine hippurate level associates with improved metabolic health in individuals with diets rich in saturated fats

**Figure 3:**
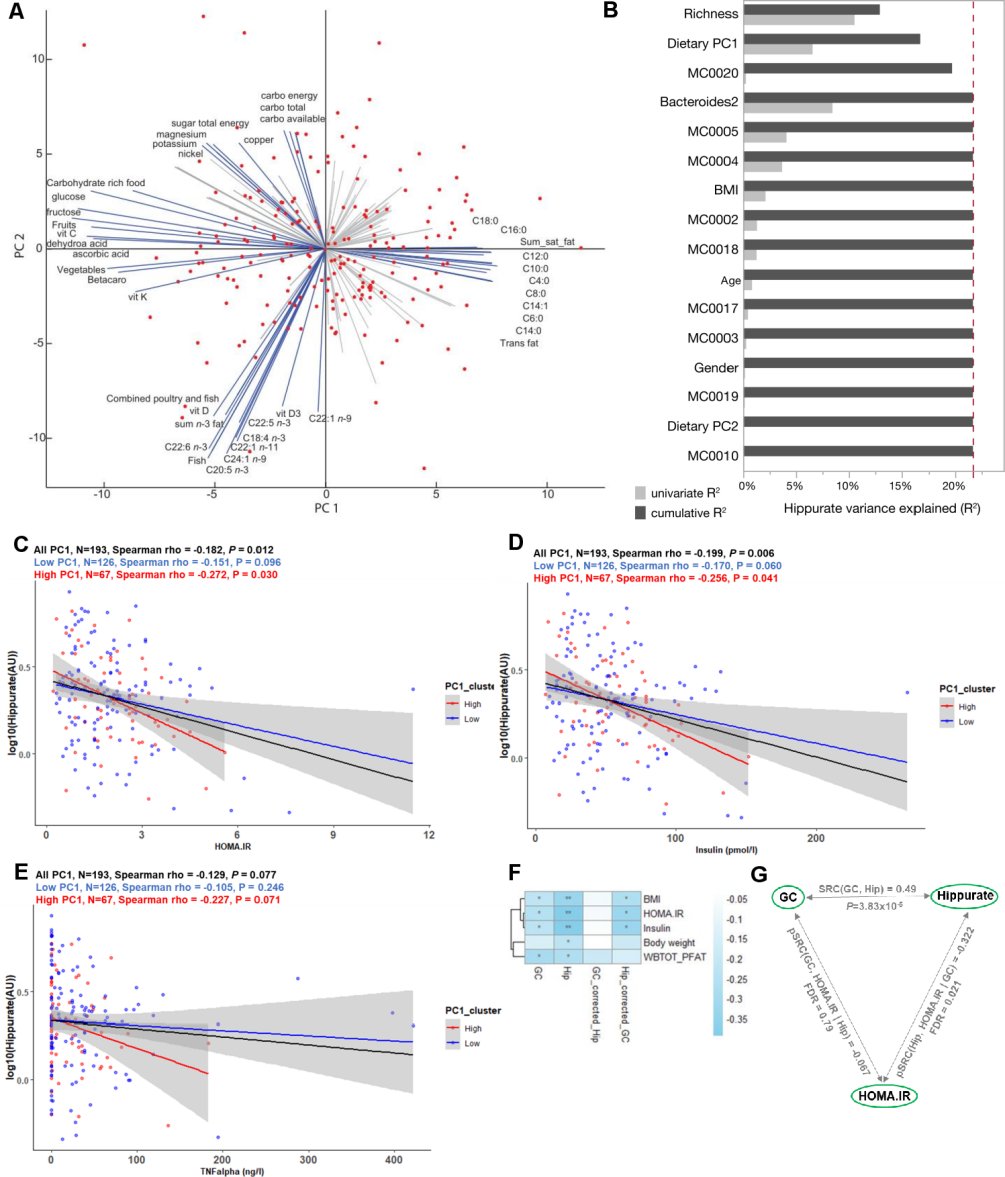
Elevated urine hippurate abundance associates with improved glucose homeostasis only in participants consuming a diet rich in saturated fats and meat. (A) Biplot of the principal component analysis (PCA) of dietary intakes highlights opposite diets along the first two principal components (PCs). The main drivers of each PCs are named and represented by blue arrows. (B) Cumulative contributions of explanatory variables to interindividual variation in hippurate excretion, estimated by stepwise rank-transformed linear regression (sLR; n=193; online supplemental table 11). Explanatory variables included age, gender, body mass index (BMI), Integrated Gene Catalogue (IGC) richness, microbiota phenylpropanoid metabolism modules and diet as dietary principal components. (C–E) Linear-regression-based scatterplots showing the association between urinary hippurate (AU; log-transformed for visualisation purposes) and homeostasis model assessment of insulin resistance (HOMA-IR), plasma insulin and tumour necrosis factor-α (TNFα) for the whole cohort (n=193; black line), for those consuming a diet rich in lipids (high PC1, n=67; red line) and for those consuming a diet rich in vegetables and fruits (low PC1, n=126, blue line). Colour-coded Spearman partial correlations and p values adjusted for age, sex and BMI are depicted above. For full name description of physiological data, see online supplemental table 10. (F) Heatmap depicting Spearman’s correlations of hippurate or microbial gene counts with adiposity bioclinical variables unadjusted or adjusted for hippurate or gene counts as indicated. WBTOT_PFAT, total body fat percentage. **Spearman p<0.01, *Spearman p<0.1 after multiple testing adjustment with the Benjamini-Hochberg method. (G) Schematic illustrating partial Spearman correlations between microbial gene counts (GC) or hippurate (Hip) with HOMA-IR after adjustment for hippurate or gene counts, respectively. The unadjusted Spearman correlation between hippurate and gene counts is shown at the top of the triangle.

We next assessed individual nutritional intake through validated FFQs available for 193 study participants.^18^ A PCA of 133 dietary intake descriptors summarises dietary patterns and loadings highlight four archetypal diets: higher consumption of fruits and vegetables versus high consumption of meat containing saturated fats on the first principal component (PC1) and carbohydrate-rich foods versus fish-containing unsaturated fats on PC2 (figure 3A), a trend that was confirmed at the food ingredient and nutrient level (online supplemental figure 8A, B). We therefore used K-means clustering to stratify the population according to dietary PC1 contrasting between healthy (low-PC1, higher consumption of fruit and vegetables; n=126) and at-risk (high-PC1, higher consumption of saturated lipids and meat; n=67) diets (online supplemental figure 8C). The clinical variables of individuals in the two dietary clusters were not significantly different, unlike the consumption of main dietary items including meat, potatoes and saturated fat (online supplemental table 10).

10.1136/gutjnl-2020-323314.supp19 Supplementary data

10.1136/gutjnl-2020-323314.supp20 Supplementary data

10.1136/gutjnl-2020-323314.supp21 Supplementary data

To summarise the main factors influencing interindividual variation in hippurate excretion, we calculated the cumulative contribution of several covariates using stepwise rank-transformed linear regression (sLR, n=193; figure 3B; online supplemental table 11). Microbial gene richness accounted for 12.95% of the variation in urine hippurate (p=2.76×10^-7^), with dietary PC1 (p=3.78×10^-3^), MC0020 encoding for hippurate dehydrolase (p=8.36×10^-3^), and Bact2 prevalence (p=3.07×10^-2^), respectively, adding an additional 3.76%, 3.02% and 1.97% to the cumulative, non-redundant explanatory power. While gene richness was positively associated with secreted hippurate, all other factors displayed a negative correlation (online supplemental table 11). When replacing dietary PCs with individual food items, the latter did not contribute significantly to hippurate excretion (online supplemental figure 8D, online supplemental table 11).

We next set out to disentangle the interaction between dietary habits, hippurate association and glycaemic control. Indeed, hippurate more strongly associated with lower HOMA-IR in those consuming an at-risk diet (high PC1; rho=−0.272, p=0.03; figure 3C, red) when compared with those with lower lipid intake (low PC1; rho=−0.151, p=0.096; figure 3C, blue) or the whole population (rho=−0.182, p=0.012; figure 3C, black), adjusted for age, sex and BMI. Similar observations were made for circulating insulin and tumour necrosis factor-α (TNFα) (figure 3D, E). Using K-means to stratify for hippurate each dietary cluster (online supplemental figure 8C) revealed that for the subset of 67 individuals consuming an at-risk diet, elevated urinary hippurate associated with improved insulin sensitivity, lower fasting insulin and lower fasting associated adipocyte factor (FIAF)^51^ or C-reactive protein (online supplemental table 11, online supplemental figure 9A–D). Urinary hippurate did not associate with any health benefits in the subsets of participants consuming mostly a fruit and vegetable diet (low PC1, online supplemental table 12) despite having similar levels of hippurate to individuals with high-PC1 (online supplemental figure 9E). Similarly, hippurate levels associated with limited benefits for those consuming a high carbohydrate diet but not a pescetarian diet (online supplemental table 12).

10.1136/gutjnl-2020-323314.supp22 Supplementary data

10.1136/gutjnl-2020-323314.supp23 Supplementary data

To disentangle contributions of hippurate and microbial gene richness to bioclinical variables in subjects consuming a fat-rich diet, we adjusted Spearman’s rank-based correlations. In this subpopulation, hippurate levels significantly correlated with low adiposity and better glycaemic control, similarly to gene richness (figure 3F). However, the associations between gene richness and bioclinical variables collapsed after adjusting for secreted hippurate (rho=−0.067, NS), suggesting it was contributed by the partial correlation between gene richness and hippurate (figure 3G). This finding suggests that not only is hippurate the strongest excreted marker for gene richness, but that it may have a beneficial effect in metabolic disease driven by diets rich in saturated fats.

### Chronic hippurate treatment modulates glucose homeostasis in mice

**Figure 4:**
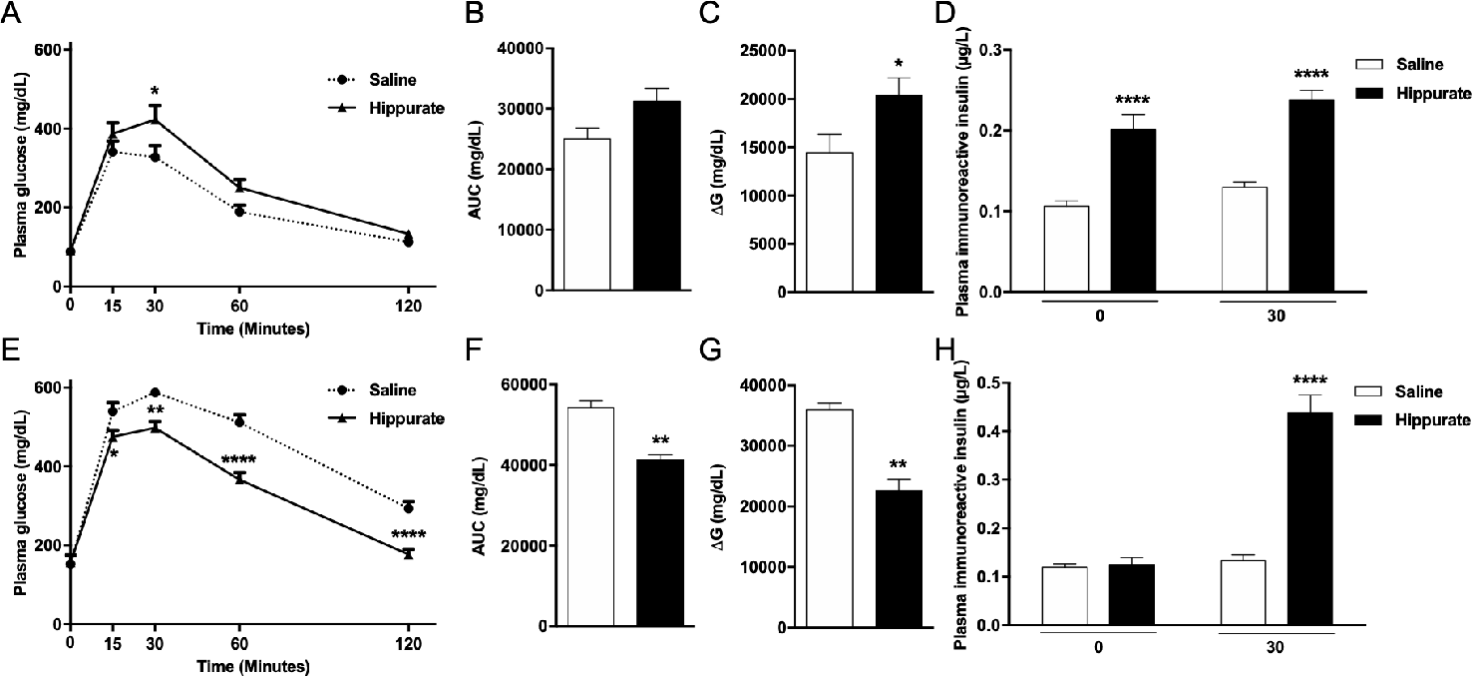
Effects of chronic subcutaneous administration of hippurate on glucose tolerance and insulin secretion in C57BL/6J mice. Mice were fed control chow diet (A–D) or high-fat diet (E–H). The effects of chronic subcutaneous administration of hippurate (5.55 mM) for 42 days were tested on glucose tolerance (A–C, E–G) and glucose-stimulated insulin secretion (D, H). Control mice were treated with saline. Area under the curve (AUC) was calculated as the sum of plasma glucose values during the intraperitoneal glucose tolerance test (IPGTT). ΔG is the AUC over the baseline value integrated over the 120 min of the IPGTT. All glycaemia and insulin measures during the IPGTT are from 6 mice/group. Data were analysed using the unpaired Mann-Whitney test. Results are means±SEM. *p<0.05; **p<0.01; ****p<0.0001, significantly different between mice treated with hippurate and saline-treated controls.

Since hippurate was associated with markers of metabolic health primarily in subjects consuming diets rich in saturated fats, we investigated diet-dependent influences of hippurate on host metabolism in mice. Mice were treated with 5.55 mM hippurate (0.14 mg/kg/day) (online supplemental figure 1). Subcutaneous infusion ensured constant delivery of hippurate, avoiding first-pass metabolism. Hippurate did not affect body weight, BMI or fasting glycaemia (online supplemental figure 10A–F). Hippurate reduced glucose tolerance in the CHD-fed lean mice (figure 4A–C). Glycaemia 30 min after glucose challenge and the ΔG parameter were significantly elevated in hippurate-treated mice than controls. Conversely, hippurate improved glucose tolerance in HFD-fed obese mice, exemplified by a significant reduction in cumulative glycaemia (−23.90%, p<0.005) and ΔG parameter (−37.22%, p<0.005; figure 4E–G). Strongly enhanced insulin release and glucose-induced insulin secretion in hippurate-treated lean mice (figure 4D) suggests a direct effect on insulin secretion. In contrast, hippurate administration in HFD-fed mice enhanced insulin secretion in response to glucose and may account for improved glucose tolerance mediated by hippurate in obese mice. Hippurate significantly increased insulin-positive areas in lean (+294%, p=0.0063) and obese mice (+348%, p=0.0468; online supplemental figure 11A, B). Staining for Ki67-positive nuclei revealed that β-cell proliferation was increased by hippurate (+289.73%, p=0.0152) only in CHD-fed mice (online supplemental figure 11C). Altogether, data from hippurate-treated mice broadly agree with the metabolic improvements observed in the human study: hippurate improves glucose homeostasis under HFD conditions and increases insulin-positive beta cell mass.

10.1136/gutjnl-2020-323314.supp24 Supplementary data

10.1136/gutjnl-2020-323314.supp25 Supplementary data

## Discussion

We integrated metabolomics with metagenomics in 271 middle-aged non-diabetic subjects from the MetaHIT study^9^ and identified urinary hippurate as the metabolite most significantly associated with microbial gene richness. Dissection of the microbiome determinants of hippurate production led to the identification of two metabolic modules associated with urinary concentrations, one of them leading to the synthesis of its precursor benzoate. We then highlighted diet-dependent relationships between microbiota–host co-metabolism of benzoate and hippurate, demonstrating that hippurate is primarily associated with metabolic benefits in individuals consuming a saturated fat-rich diet. Consistent with associations between microbial gene richness and insulin sensitivity reported in MetaHIT^9^ and associations between circulating hippurate and reduced metabolic disease risk,^23 25 26 28^ our findings suggest that elevated levels of hippurate are a marker of metabolic health, primarily in people with habitual diets rich in saturated fats.

Our analyses provide new insights in the microbial background of benzoate production, with two modules being significantly associated with urine hippurate variation. These modules cover the degradation of cinnamate, an intermediate in the metabolism of a broad range of plant secondary metabolites,^52^ present in large quantities of its unmodified form in, for example, berries.^53^ The phenylpropanoid pathway connects a wide range of dietary substrates such as phenylalanine, quinic acid, shikimic acid or chlorogenic acid to benzoate being often a common endpoint. Dietary and microbial intermediates in this pathway are associated with beneficial health outcomes.^23 54^ Both modules were distributed unevenly over the gut ecosystem main bacterial phyla, notably remaining undetected among *Bacteroidetes*. The latter observation was mirrored by their low relative abundances in the dysbiotic Bact2 enterotype, which is characterised by low bacterial cell counts, low microbial gene richness, and respectively low and high relative abundances of butyrate producers and *Bacteroides* spp^55^ compared with Prev or Rum enterotypes. Bact2 prevalence associates with stool moisture content,^49^ inflammation,^6 49^ obesity and insulin resistance.^6^ This community has been described as immature, with its metabolic potential reflecting refrained/prematurely halted successional ecosystem development.^55 56^ Hence, benzoate production could be thought of as an emergent community feature linked to ecosystem maturation into eubiosis. Remarkably, out of the individual modules included in the present analysis, only MC00020 encoding hippurate hydrolase contributed to urine hippurate concentrations beyond dietary principal components and enterotype. This suggests that in non-Bact2 enterotypes, benzoate production largely depends on food intake in agreement with Pallister *et al*.^23^ The negative contribution of hippurate hydrolase to urinary concentrations further suggests higher deconjugation of dietary hippurate and/or retroconversion of detoxified benzoate after excretion in the intestinal tract, as demonstrated for trimethylamine-*N*-oxide.^57^ This microbiome determinism of urinary hippurate levels appears to mirror and antagonise host genetic determinism we identified for benzoate in a rat F2-intercross.^58^

Our finding that hippurate exerts beneficial metabolic effects in the context of high-fat diet was replicated in a mouse model of HFD-induced obesity. Hippurate dramatically improved glucose tolerance in obese HFD-fed mice which may be explained by stimulation of glucose-induced insulin secretion and/or β-cell mass increase in a diet-dependent manner. Although glucose tolerance displays an interaction between diet and response to hippurate in CHD-fed mice, the mechanism of which remaining elusive, the main effect is an increased glucose tolerance in HFD in both mice and humans. Collectively, our preclinical studies suggest that some of the beneficial metabolic effects of hippurate may be mediated through direct action on the pancreas. This is consistent with our work showing an inverse association among hippurate, insulin resistance, hypertension, obesity or liver steatosis,^24–26 28^ and observations that hippurate exerts protective effects in β-cells.^59^ Further experimentation would be required to unequivocally establish this.

## Conclusion

Overall, we identify hippurate as a pivotal microbial–host co-metabolite mediating part of the beneficial metabolic improvements associated with high microbial gene richness in the context of Western-style diets. This work expands previous reports in which hippurate was inversely associated with insulin resistance, steatosis, hypertension and obesity,^24–26 28^ and microbial ecological diversity.^23 28^ Our work provides a simple beneficial marker documenting the diversity of microbial ecosystems and functions, as well as providing health benefits in terms of metabolic control. Our observations support the existence of several microbial–host metabolic states with different responses to diet and health outcomes for the host, further exemplifying the role of the microbiome in human biochemical individuality^60^ and provides avenues in personalised nutrition and stratified medicine.^61 62^

## Data Availability

Data are available upon reasonable request to the corresponding authors
