# Supplementary material for: Human and preclinical studies of the host–gut microbiome co-metabolite hippurate as a marker and mediator of metabolic health
Source: Gut. 2021 May 11;70(11):2105–14. doi: 10.1136/gutjnl-2020-323314 (PMC8515120; doi:10.1136/gutjnl-2020-323314)
Supplement: Supplementary data [file gutjnl-2020-323314supp001.pdf]

BMJ Publishing Group Limited (BMJ) disclaims all liability and responsibility arising from any reliance placed on this supplemental material which has been supplied by the author(s)

Supplementary Data 1

```
MC0001     phenylalanine degradation (cinnamate production)
cpd   [phenylalanine]  [cinnamate, NH3]
K10775
ref   MetaCyc Pathway: benzoate biosynthesis I (CoA-dependent, fl-
oxidative)
///
MC0002     chlorogenate degradation
cpd   [chlorogenate]   [caffeate, quinate]
K06889     K09252
ref   MetaCyc Pathway: chlorogenic acid degradation      PMID: 11412317
///
MC0003     caffeate respiration
cpd   [caffeate]  [hydrocaffeate]
bactNOG04579
ref   PMID: 23315745
///
MC0004     cinnamate conversion
cpd   [cinnamate] [phenylpropanoate]
K10797
ref   KEGG Pathway: phenylalanine metabolism
///
MC0005     coumarate degradation
cpd   [coumarate] [hydroxybenzoate]
cpd   [ferulate]  [vanillate]
cpd   [caffeate]  [protocatechuate]
cpd   [cinnamate] [benzoate]
K01904     bactNOG05057
K18383     K01692     K01715     K01782     K01825     K13767
      K15016     bactNOG19280
K21802     K00141
ref   MetaCyc Pathway: 4-coumarate degradation (anaerobic)     PMID:
22328668
///
MC0006     (hydroxy)benzoate degradation
cpd   [(hydroxy)benzoate]    [3-hydroxypimeloyl-CoA]
K04105,K04107,K04108,K04109  K20458,K04107,K04108,K04109  K04110
      bactNOG00950
K04112,K04113,K04114,K04115  K19515,K19516
K07537
K07538
K07539
ref   MetaCyc Pathway: 4-coumarate degradation (anaerobic)     MetaCyc
Pathway: benzoyl-CoA degradation II (anaerobic)     Kegg module: M00541
///
MC0007     ferulate degradation
cpd   [ferulate]  [vanillin]
K12508
K18383
ref   MetaCyc Pathway: ferulate degradation
///
MC0008     vanillin conversion
cpd   [vanillin, (O2)] [protocatechuate]
K21802     bactNOG00059
K03862,K03863    K15066
ref   MetaCyc Pathway: superpathway of vanillin and vanillate
degradation Kegg Pathway: aminobenzoate degradation
```

```
///
MC0009     cinnamate degradation
cpd   [cinnamate] [benzoyl-CoA]
bactNOG08521
actNOG07134
COG0277
bactNOG05297
ref   MetaCyc Pathway: trans-cinnamoyl-CoA biosynthesis   MetaCyc
Pathway: benzoyl-CoA biosynthesis
///
MC0010     quinate degradation
cpd   [quinate]   [protocatechuate]
cpd   [shikimate] [protocatechuate]
K05887     K05358     COG0169
K03785     K03786     K13832
K09483     K15652
ref   MetaCyc Pathway: quinate degradation I  MetaCyc Pathway: quinate
degradation II   PMID: 12620848
///
MC0011     4-hydroxybenzoate conversion
cpd   [4-hydroxybenzoate, O2]      [protocatechuate]
K00481
ref   Kegg Pathway: benzoate degradation PMID: 12620848   PMID: 14556848
///
MC0012     catechin degradation
cpd   [catechin]  [protocatechuate, phloroglucinol carboxylic acid]
bactNOG14887
ref   PMID: 12608578
///
MC0013     3-hydroxybenzoate conversion
cpd   [3-hydroxybenzoate, O2]      [protocatechuate]
K19065
ref   Kegg Pathway: benzoate degradation
///
MC0014     benzoate conversion
cpd   [benzoate, O2]   [4-hydroxybenzoate]
K07824
ref   Kegg Pathway: benzoate degradation
///
MC0015     benzoate degradation (aerobic)
cpd   [benzoate, O2]   [catechol, CO2]
K05549,K05550,K05784
K05783
ref   Kegg module: M00551    MetaCyc Pathway: benzoate degradation I
(aerobic)
///
MC0016     benzoate degradation (anaerobic)
cpd   [benzoyl-CoA]    [3-hydroxypimeloyl-CoA]
K04112,K04113,K04114,K04115  K19515,K19516
K19066
K07534
K07535
K07536
K04118
ref   MetaCyc Pathway: benzoyl-CoA degradation III (anaerobic) Kegg
module: M00540
///
MC0017     phenylalanine degradation
```

```
cpd   [phenylalanine, 2-oxoglutarate]   [CO2, glutamate, benzoyl-CoA]
K00832     K00812     K00813     K11358     K00817
K04103
K00146     K00129
K01912
K18361
K18355,K18356,K18357,K18358,K18359
ref   MetaCyc Pathway: phenylacetate degradation II (anaerobic)MetaCyc
Pathway: L-phenylalanine degradation II (anaerobic)
///
MC0018     cellulose and hemicellulose degradation (cellulolosome)
cpd   [cellulose, hemicellulose]   [ferulate, polysaccharide,
oligosaccharide]
K01181     K13465
K09252
bactNOG05428
ref   MetaCyc Pathway: cellulose and hemicellulose degradation
(cellulolosome)
///
MC0019     feruloyl esterase (cellulolosome)
cpd   [cellulose, hemicellulose]   [ferulate]
K09252
ref   MetaCyc Pathway: cellulose and hemicellulose degradation
(cellulolosome)  MC0018
///
MC0020     hippurate hydrolase
cpd   [hippurate] [glycine, benzoate]
K01451
ref   PMID: 18665452
///
```
