# Supplementary figures and images for: Human and preclinical studies of the host–gut microbiome co-metabolite hippurate as a marker and mediator of metabolic health
Source: Gut. 2021 May 11;70(11):2105–14. doi: 10.1136/gutjnl-2020-323314 (PMC8515120; doi:10.1136/gutjnl-2020-323314)

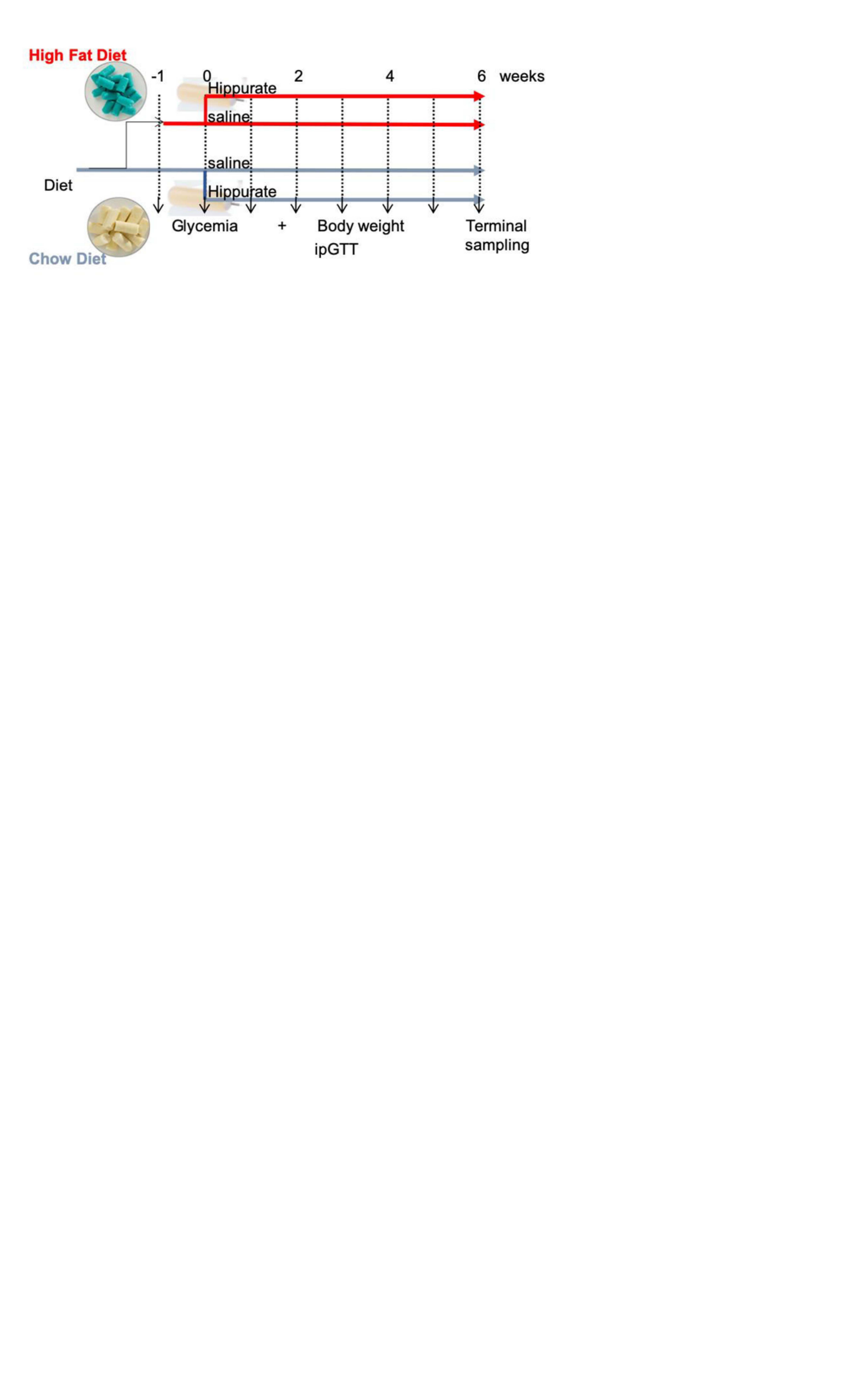

Supplemental Figure 1

Supplement: Supplementary data [file gutjnl-2020-323314supp002.pdf]

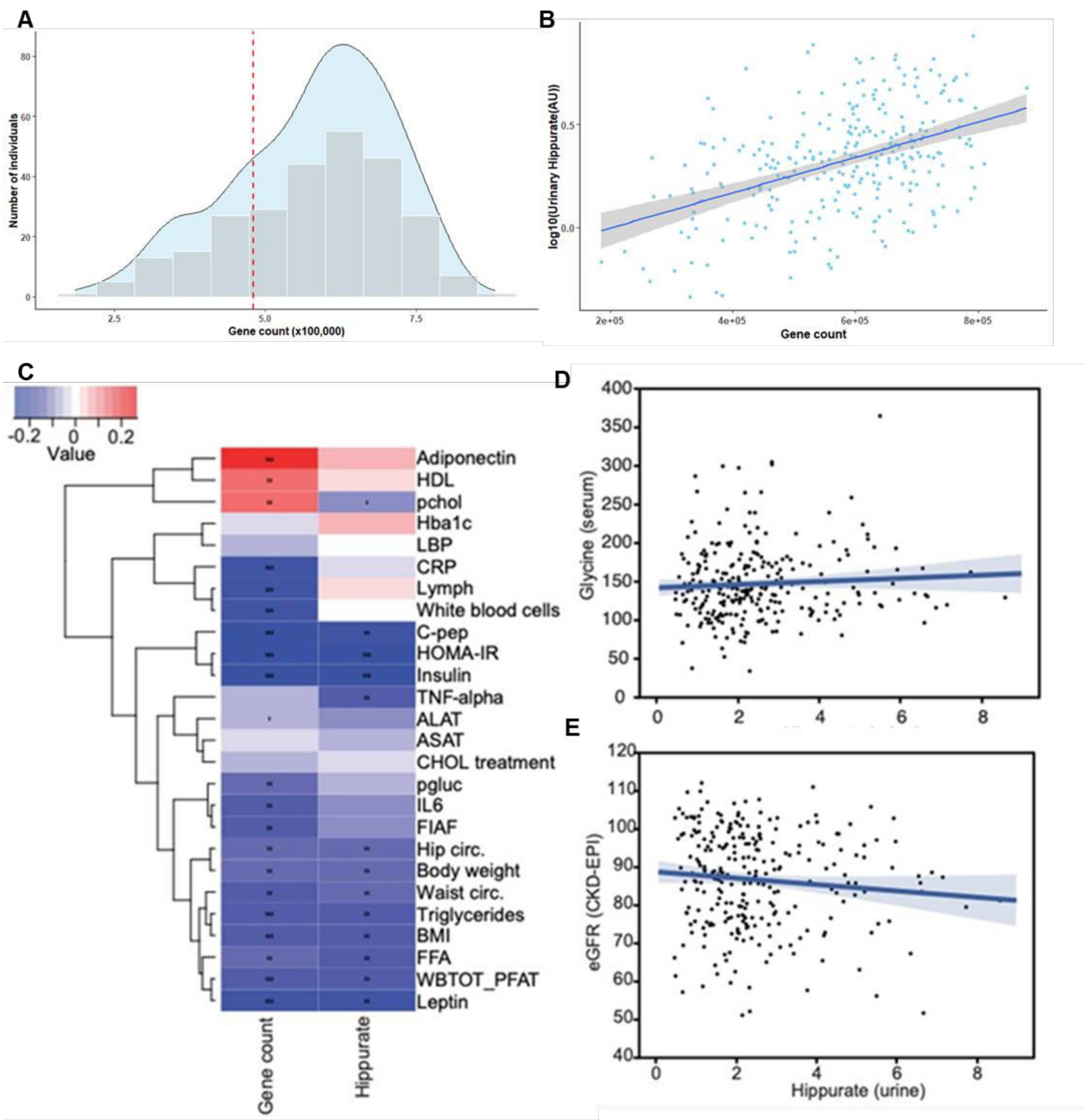

Supplemental Figure 2

Supplement: Supplementary data [file gutjnl-2020-323314supp004.pdf]

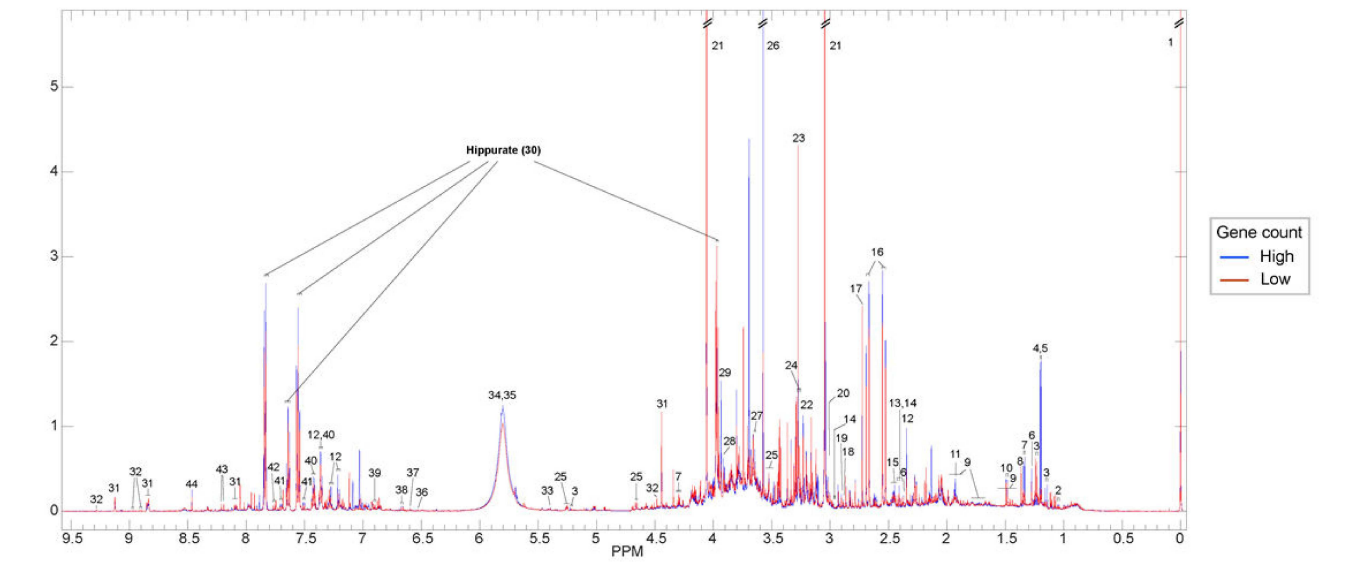

Supplemental Figure 3

Supplement: Supplementary data [file gutjnl-2020-323314supp006.pdf]

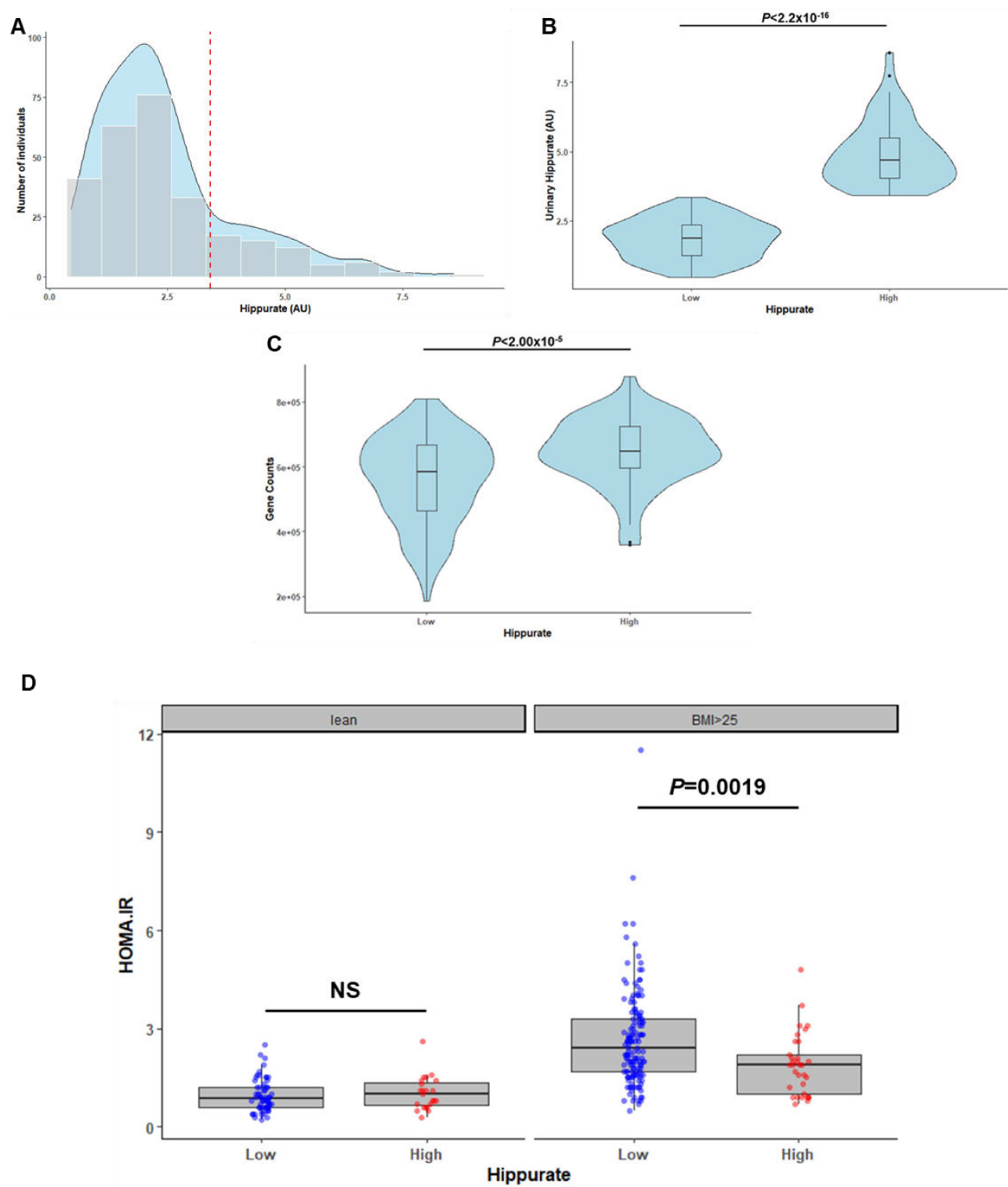

Supplemental Figure 4

Supplement: Supplementary data [file gutjnl-2020-323314supp008.pdf]

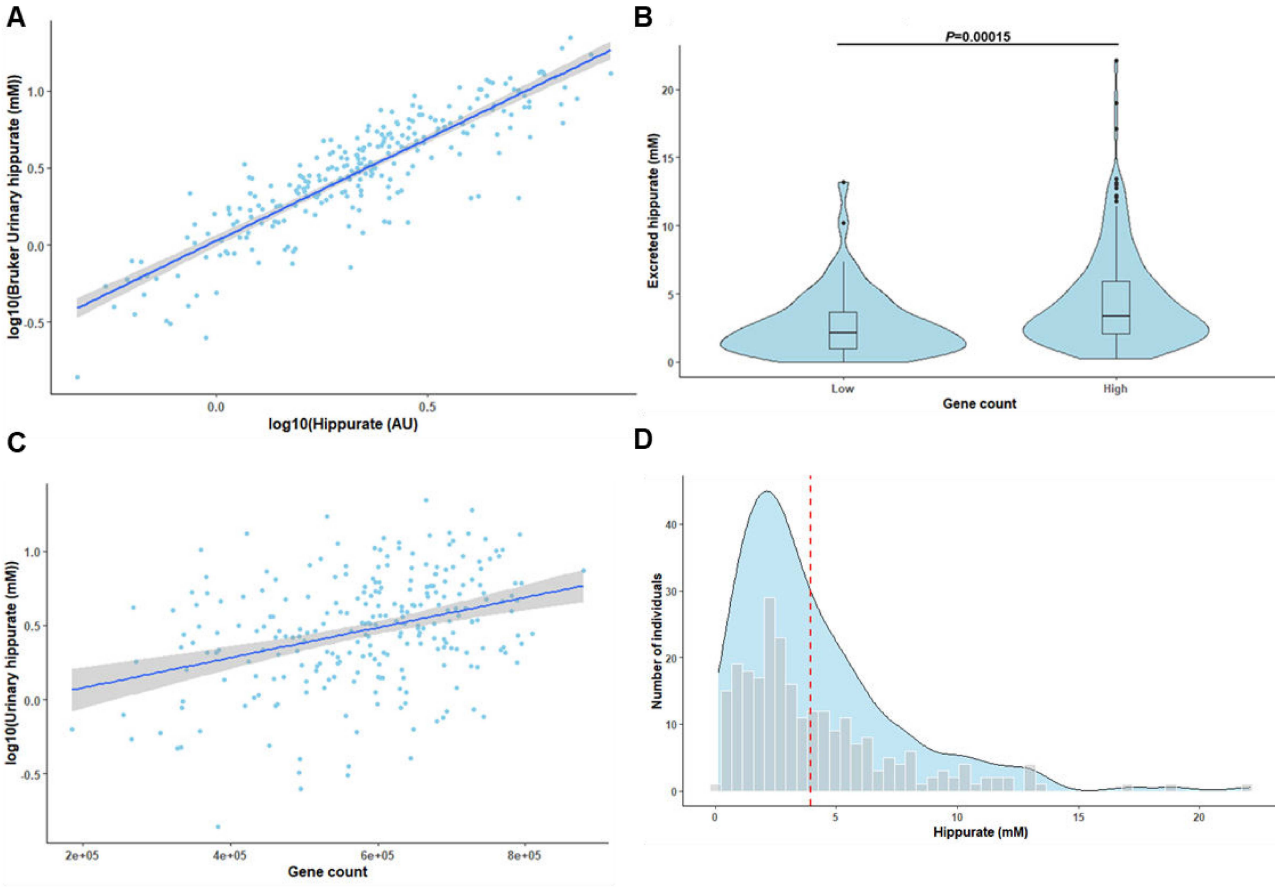

Supplemental Figure 5

Supplement: Supplementary data [file gutjnl-2020-323314supp009.pdf]

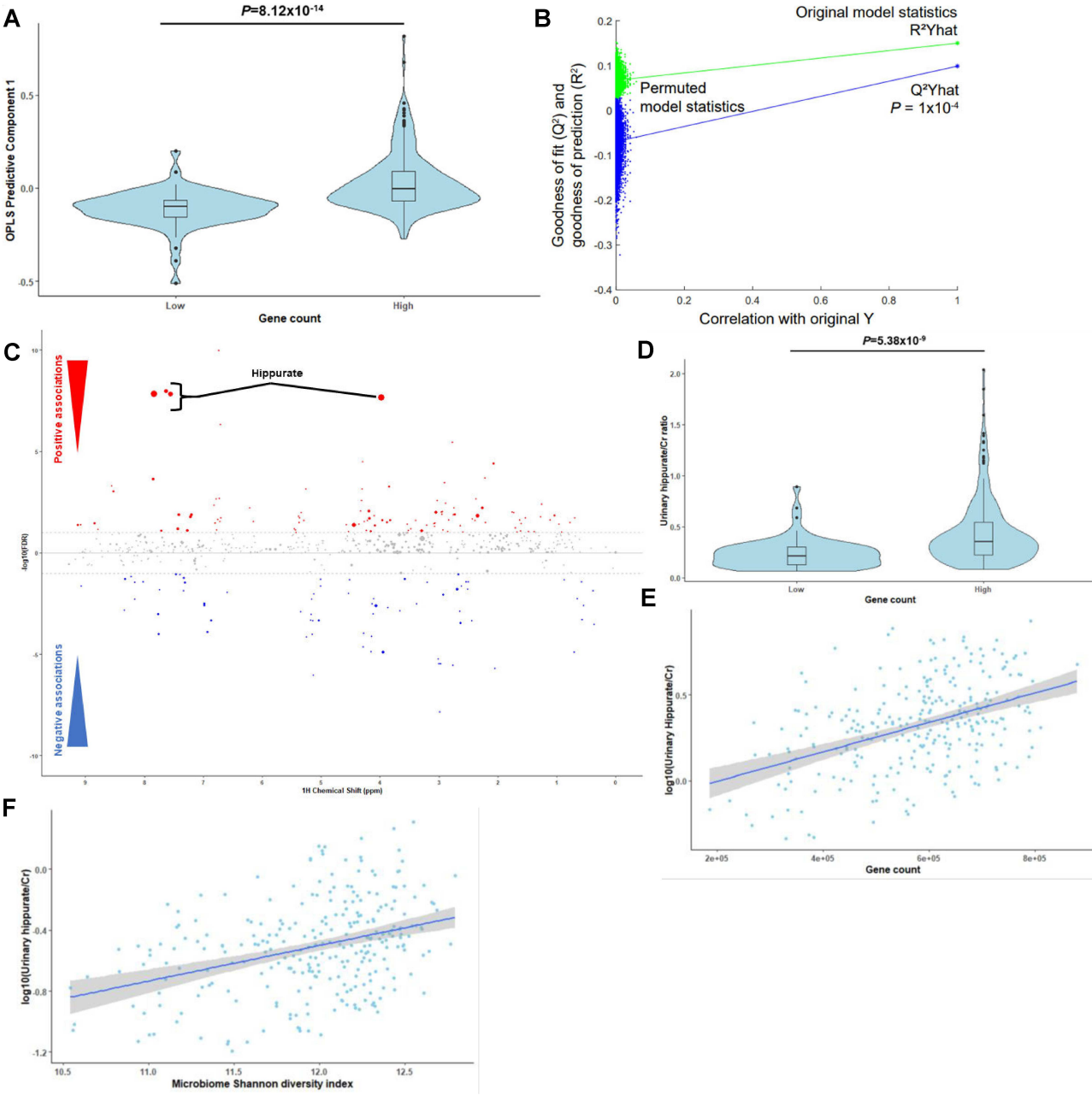

Supplemental Figure 6

Supplement: Supplementary data [file gutjnl-2020-323314supp010.pdf]

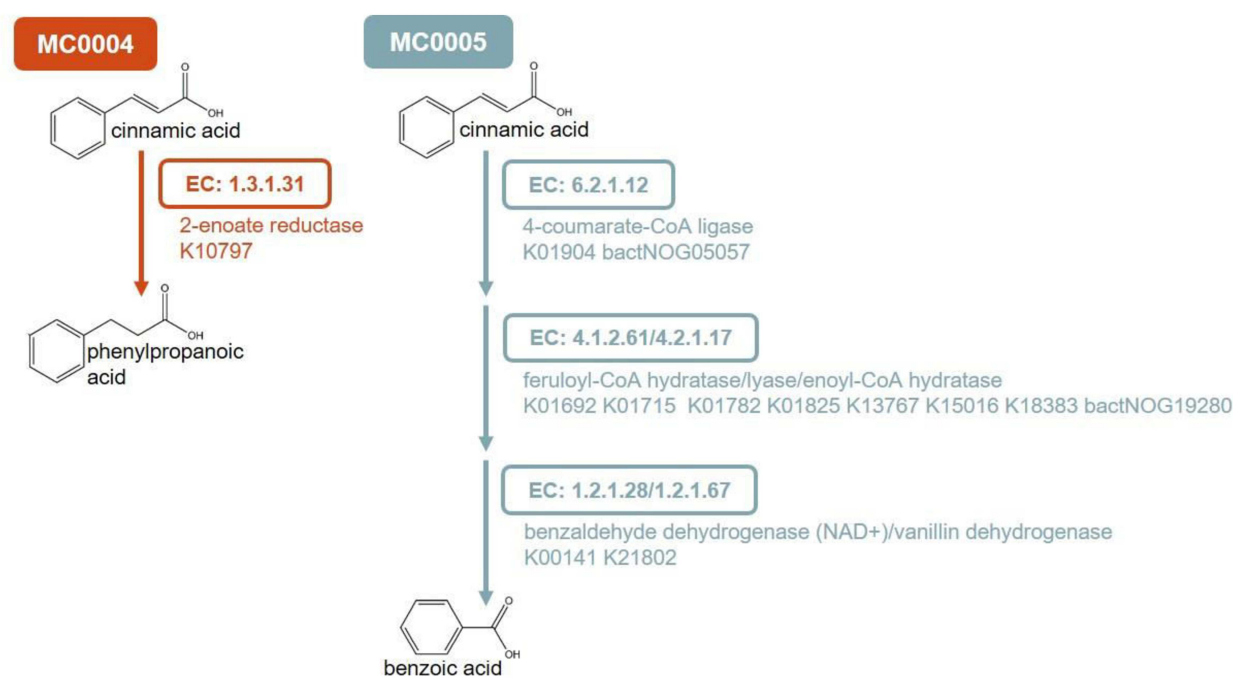

Supplemental Figure 7

Supplement: Supplementary data [file gutjnl-2020-323314supp012.pdf]

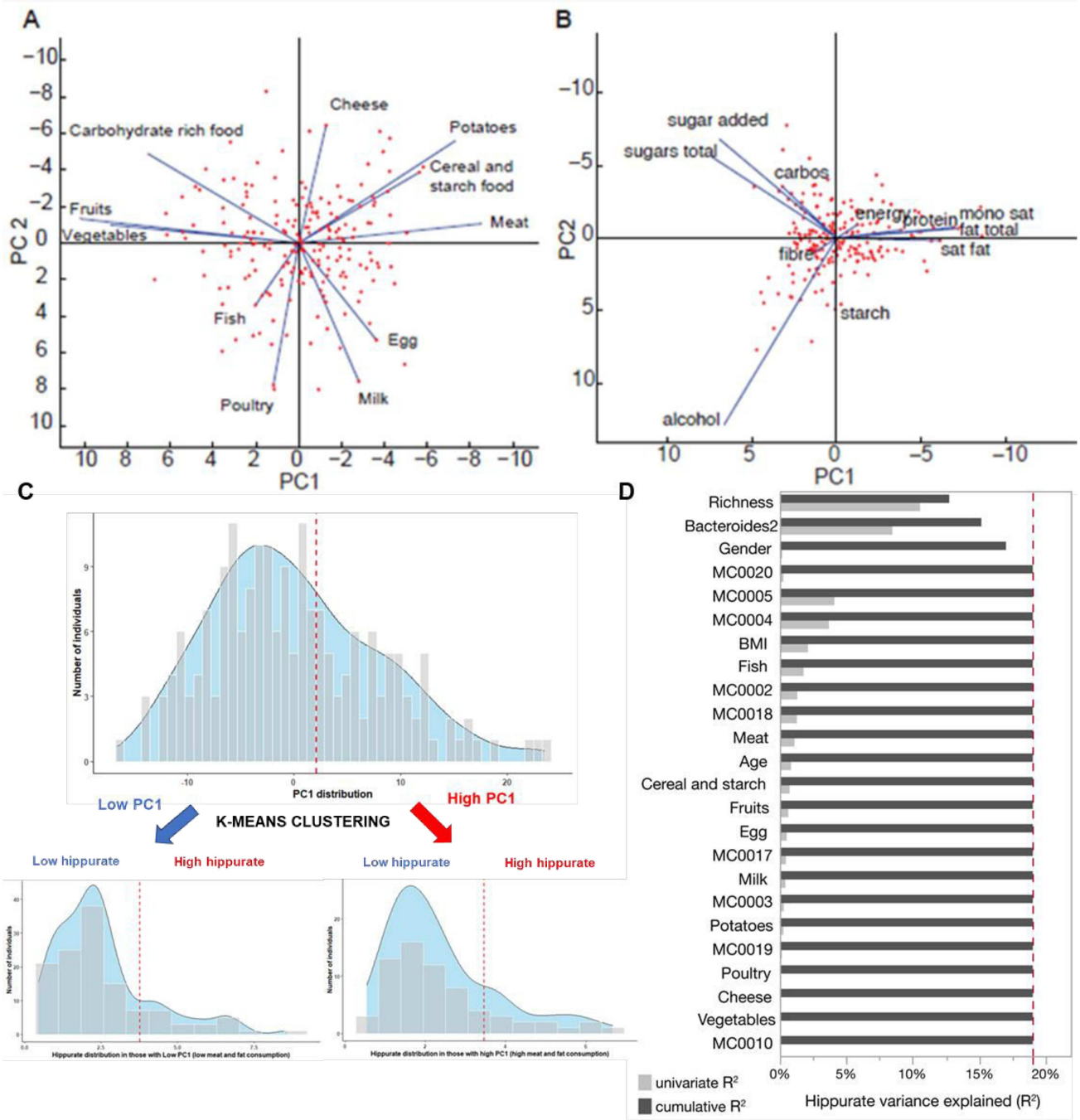

Supplemental Figure 8

Supplement: Supplementary data [file gutjnl-2020-323314supp019.pdf]

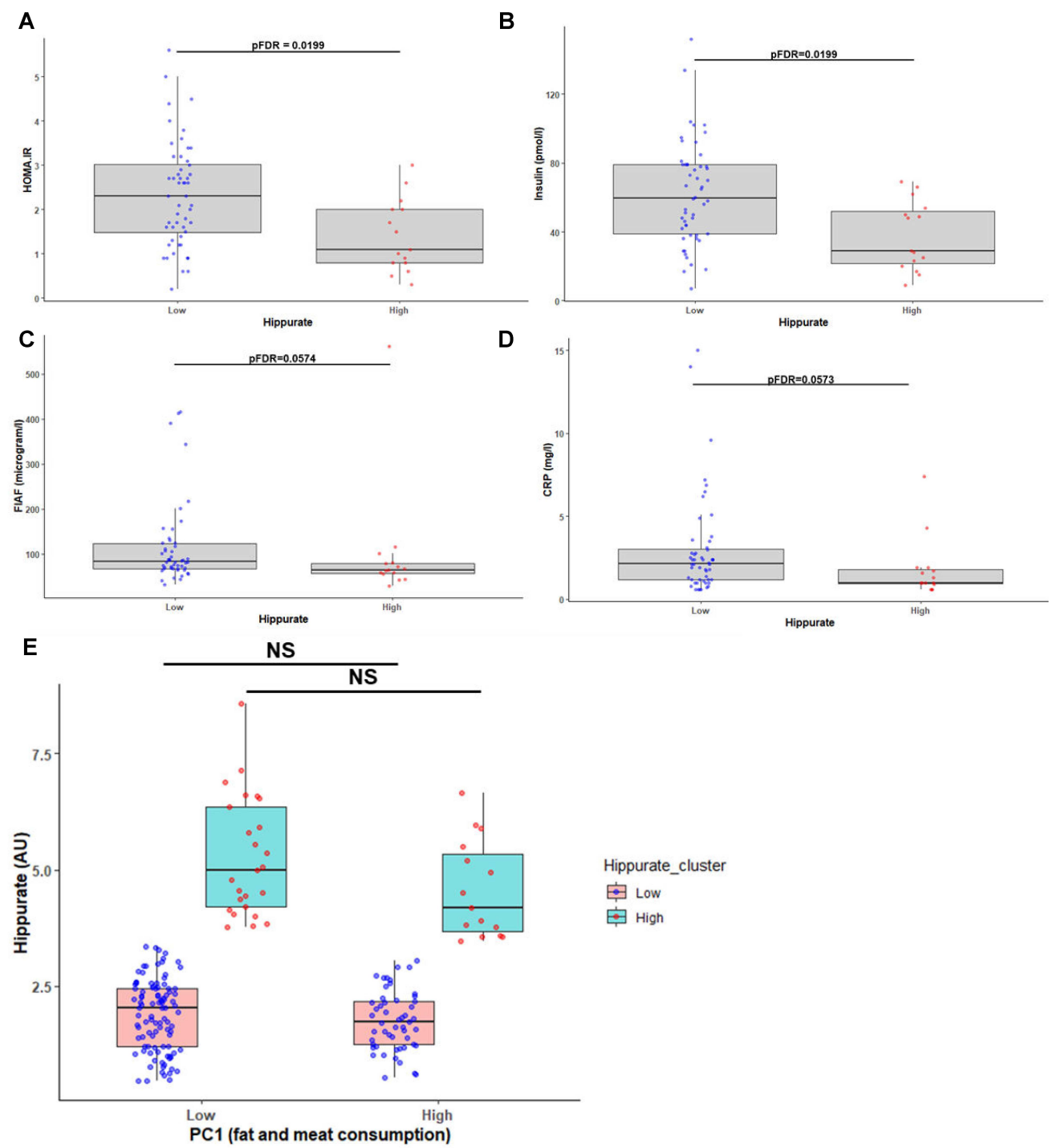

Supplemental Figure 9

Supplement: Supplementary data [file gutjnl-2020-323314supp022.pdf]

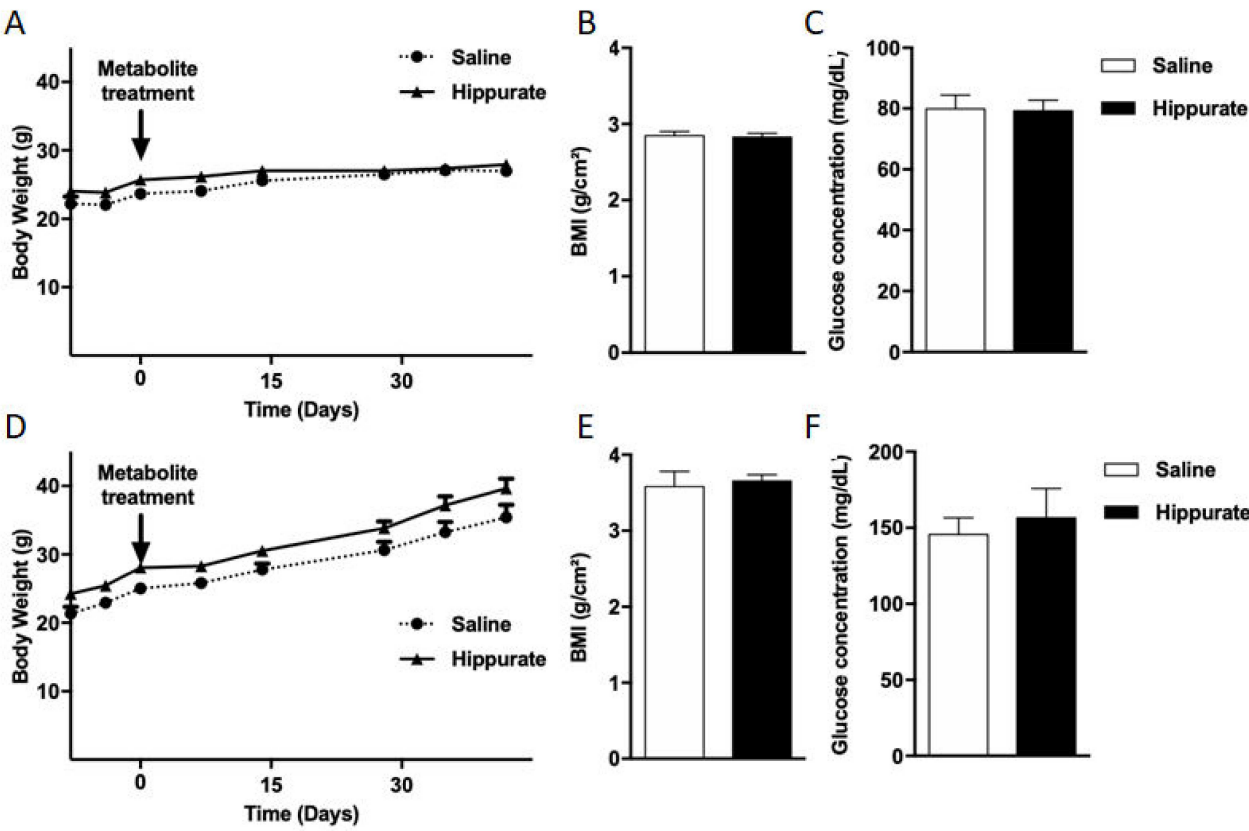

Supplemental Figure 10

Supplement: Supplementary data [file gutjnl-2020-323314supp024.pdf]

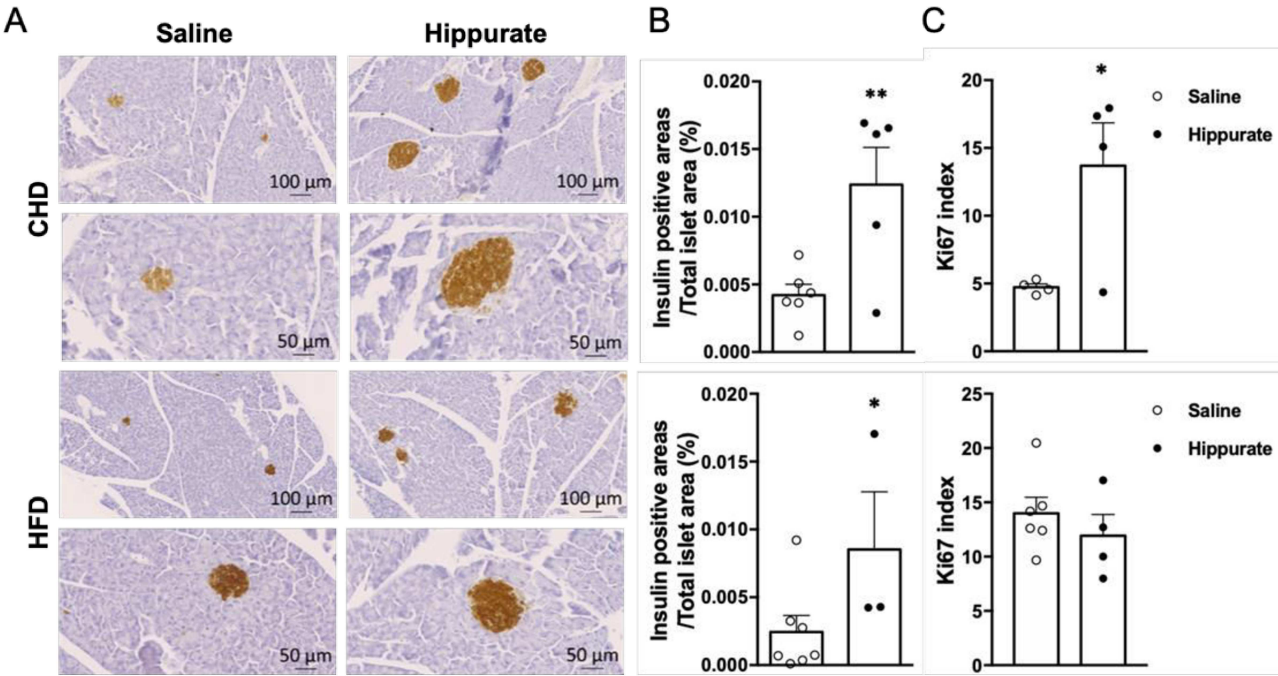

Supplemental Figure 11

Supplement: Supplementary data [file gutjnl-2020-323314supp025.pdf]
