# Supplementary material for: Human and preclinical studies of the host–gut microbiome co-metabolite hippurate as a marker and mediator of metabolic health
Source: Gut. 2021 May 11;70(11):2105–14. doi: 10.1136/gutjnl-2020-323314 (PMC8515120; doi:10.1136/gutjnl-2020-323314)
Supplement: Supplementary data [file gutjnl-2020-323314supp003.pdf]

Supplementary Data 2. Details of the formulation of the control diet (10% fat) (D12450Ki, Research Diets Inc. NJ) and the high fat diet (60% fat) (D12492i, Research Diets Inc. NJ) used to promote obesity in C57BL6J mice.

**Control Diet**	**D12450Ki**
**Caloric Information**	
Protein	**20** % Kcal
Fat	**10** % Kcal
Carbohydrate	**70** % Kcal
Energy density	**3.82** Kcal/g

**Class description**	**Ingredients**	**Grams**
Protein	Casein, Lactic, 30 Mesh	200.00 g
Protein	Cystine, L	3.00 g
Carbohydrate	Starch, Corn	550.00 g
Carbohydrate	Lodex 10	150.00 g
Carbohydrate	Sucrose, Fine Granulated	4.00 g
Fiber	Solka Floc, FCC200	50.00 g
Fat	Soybean Oil, USP	25.00 g
Fat	Lard	20.00 g
Mineral (See below)	S10026B	50.00 g
Vitamin	Choline Bitartrate	2.00 g
Vitamin (See below)	V10001C	1.00 g
Dye	Dye, Blue FD&C #1, Alum. Lake 35-42%	0.03 g
Dye	Dye, Red FD&C #40, Alum. Lake 35-42%	0.03 g
	Total:	1055.05 g

**High Fat Diet**	**D12492i**	
**Caloric Information**		
Protein	**20** % Kcal	
Fat	**60** % Kcal	
Carbohydrate	**20** % Kcal	
Energy density	**5.21** Kcal/g	
**Class description**	**Ingredients**	**Grams**
Protein	Casein, Lactic, 30 Mesh	200.00 g
Protein	Cystine, L	3.00 g
Carbohydrate	Lodex 10	125.00 g
Carbohydrate	Sucrose, Fine Granulated	72.80 g
Fiber	Solka Floc, FCC200	50.00 g
Fat	Lard	245.00 g
Fat	Soybean Oil, USP	25.00 g
Mineral (See below)	S10026B	50.00 g
Vitamin	Choline Bitartrate	2.00 g
Vitamin (See below)	V10001C	1.00 g
Dye	Dye, Blue FD&C #1, Alum. Lake 35-42%	0.05 g
	Total:	773.85 g

Mineral Mix (for 50g)

Mineral Potassium Citrate, Monohydrate	16.50 g
Mineral Calcium Phosphate, Dibasic	13.00 g
Mineral Calcium Carbonate, Light, USP	5.50 g
Mineral Sodium Chloride	2.59 g
Mineral Magnesium Sulfate, Heptahydrate	2.58 g
Mineral Magnesium Oxide, Heavy, DC USP	0.42 g
Mineral Ferric Citrate	0.21 g
Mineral Manganese Carbonate Hydrate	0.12 g
Mineral Zinc Carbonate	0.06 g
Mineral Chromium Potassium Sulfate	0.02 g
Mineral Copper Carbonate	0.01 g
Mineral Ammonium Molybdate Tetrahydrate	0.003 g
Mineral Sodium Fluoride	0.002 g
Mineral Sodium Selenite	0.0005 g
Mineral Potassium Iodate	0.0005 g

Vitamin Mix (for 1g)

Vitamin Vitamin E Acetate, 50%	0.100 g
Vitamin Niacin (a.k.a. B3)	0.030 g

Vitamin Biotin, 1%	0.020 g
Vitamin Pantothenic Acid, d, Calcium (a.k.a. B5)	0.016 g
Vitamin Vitamin D3, 100,000 IU/gm	0.010 g
Vitamin Vitamin B12, 0.1% Mannitol	0.010 g
Vitamin Vitamin A Acetate, 500,000 IU/gm	0.008 g
Vitamin Pyridoxine HCl (a.k.a. B6)	0.007 g
Vitamin Riboflavin (a.k.a. B2)	0.006 g
Vitamin Thiamine HCl (a.k.a. B1)	0.006 g
Vitamin Folic Acid	0.002 g
Vitamin Menadione Sodium Bisulfite	0.0008 g
