# Supplementary material for: Human and preclinical studies of the host–gut microbiome co-metabolite hippurate as a marker and mediator of metabolic health
Source: Gut. 2021 May 11;70(11):2105–14. doi: 10.1136/gutjnl-2020-323314 (PMC8515120; doi:10.1136/gutjnl-2020-323314)
Supplement: Supplementary data [file gutjnl-2020-323314supp005.pdf]

Supplementary Table ST.1. Modelling of urinary hippurate in function of kidney filtration rate and serum glycine levels. Least-squares multivariate linear regression of urinary hippurate levels using glycine serum levels and glomerular filtration rate as explanatory variables (N=269). All variables rank-tranformed. The full model summary statistics are reported labelled with the prefix "Model", followed by the parameters estimates statistics for each explanatory variable.

Y	Model DF	Model Sum of Squares	Model Mean Square	Model R2	Model F ratio	Model P-value	N	Term	Estimate	Std Error	t Ratio	Term P-value
Hippurate	3	20414.1	6804.7	0.01237	1.1065	0.3469	269	Intercept	145.17	14.251101	10.19	8.83E-21
Hippurate	3	20414.1	6804.7	0.01237	1.1065	0.3469	269	serum Glycine	0.02767	0.061965	0.45	0.6556
Hippurate	3	20414.1	6804.7	0.01237	1.1065	0.3469	269	eGFR	-0.09913	0.061867	-1.6	0.1103
Hippurate	3	20414.1	6804.7	0.01237	1.1065	0.3469	269	eGFR * serum G	-0.00016	0.000794	-0.2	0.8387
