# Supplementary material for: Human and preclinical studies of the host–gut microbiome co-metabolite hippurate as a marker and mediator of metabolic health
Source: Gut. 2021 May 11;70(11):2105–14. doi: 10.1136/gutjnl-2020-323314 (PMC8515120; doi:10.1136/gutjnl-2020-323314)
Supplement: Supplementary data [file gutjnl-2020-323314supp007.pdf]

Supplementary Table ST.2. Annotated of 1H NMR peaks reported in in Suppl.Fig.3

Number	Chemical shift (ppm)	Metabolite
1	0.000 (s)	TSP
2	1.041 (d)	L-valine
3	1.172 (d), 1.236 (d), 5.213 (d)	fucose
4	1.194 (d)	methylmalonate
5	1.201 (d)	3-aminoisobutyrate
6	1.274 (s)	3-hydroxyisovalerate
7	1.33 (d), 4.114 (q)	lactate
8	1.369 (s)	2-hydroxyisobutyrate
9	1.453 (m), 1.727 (m), 1.912 (m)	lysine
10	1.485 (d)	alanine
11	1.921 (s)	acetate
12	2.344 (s), 7.287 (t), 7.380 (t)	phenylacetate
13	2.407 (s)	succinate
14	2.407 (t), 3.004 (t)	2-oxoglutarate
15	2.442 (m)	L-glutamine
16	2.538 (d), 2.689 (d)	citrate
17	2.729 (s)	dimethylamine
18	2.889 (s)	TMA
19	2.903 (s)	dimethylglycine
20	3.028 (s)	creatine phosphate
21	3.052 (s), 4.062 (s)	creatinine
22	3.233 (s)	carnitine
23	3.277 (s)	TMAO
24	3.277 (t)	taurine
25	3.54 (dd), 4.659 (d), 5.248 (d)	glucose
26	3.576 (s)	glycine
27	3.67 (d)	L-isoleucine
28	3.936 (s)	glycolate
29	3.959 (s)	creatine
30	3.977 (d), 7.556 (t), 7.632 (t), 7.834 (d)	hippurate
31	4.445 (s), 8.077 (m), 8.845 (m), 9.127 (s)	trigonelline
32	4.486 (s), 8.901 (d), 8.969 (d), 9.279 (s)	1-methylnicotinamide
33	5.398 (s)	allantoin
34	5.774 (s broad)	urea
35	5.774 (s)	cis-aconitate
36	6.527 (s)	fumarate
37	6.595 (s)	trans-aconitate
38	6.66 (d)	N-methyl-2-pyridone-5-carboxamide (2-PY)
39	6.925 (d)	3-hydroxymandelate
40	7.380 (d), 7.419 (t)	N-phenylacetylglycine
41	7.505 (d), 7.696 (d)	3-indoxylsulfate
42	7.753 (d)	4-hydroxybenzoate OR 4-hydroxyhippurate
43	8.191 (s), 8.213 (s)	adenine
44	8.465 (s)	formate
