# Supplementary material for: Human and preclinical studies of the host–gut microbiome co-metabolite hippurate as a marker and mediator of metabolic health
Source: Gut. 2021 May 11;70(11):2105–14. doi: 10.1136/gutjnl-2020-323314 (PMC8515120; doi:10.1136/gutjnl-2020-323314)
Supplement: Supplementary data [file gutjnl-2020-323314supp011.pdf]

Supplementary Table ST.3. Association between microbiota functional potential mapped to KEGG Orthologs (KOs) database and urine hippurate levels.

KEGG KO	Spearman ρ	P	FDR	Description
K02899	0.409792	2.85E-12	1.97E-08	large subunit ribosomal protein L27
K02935	0.399738	1.13E-11	3.91E-08	large subunit ribosomal protein L7/L12
K02952	0.392481	2.90E-11	6.68E-08	small subunit ribosomal protein S13
K02961	0.37576	2.23E-10	2.57E-07	small subunit ribosomal protein S17
K02990	0.378693	1.58E-10	2.57E-07	small subunit ribosomal protein S6
K02994	0.376085	2.15E-10	2.57E-07	small subunit ribosomal protein S8
K02887	0.372484	3.28E-10	3.23E-07	large subunit ribosomal protein L20
K02888	0.365768	7.08E-10	6.11E-07	large subunit ribosomal protein L21
K04078	0.362371	1.04E-09	7.96E-07	chaperonin GroES
K00205	0.356358	1.55E-09	8.33E-07	formylmethanofuran dehydrogenase subunit F EC:1.2.99.5
K02864	0.35799	1.69E-09	8.33E-07	large subunit ribosomal protein L10
K02867	0.358219	1.65E-09	8.33E-07	large subunit ribosomal protein L11
K02892	0.358773	1.55E-09	8.33E-07	large subunit ribosomal protein L23
K06416	0.359451	1.44E-09	8.33E-07	stage V sporulation protein S
K06297	0.356638	1.96E-09	9.02E-07	spore germination protein KC
K02986	0.354268	2.54E-09	1.10E-06	small subunit ribosomal protein S4
K02965	0.353186	2.86E-09	1.16E-06	small subunit ribosomal protein S19
K00171	0.350923	3.65E-09	1.20E-06	pyruvate ferredoxin oxidoreductase, delta subunit EC:1.2.7.1
K02313	0.348287	4.83E-09	1.20E-06	chromosomal replication initiator protein
K02876	0.3482	4.88E-09	1.20E-06	large subunit ribosomal protein L15
K02895	0.352274	3.15E-09	1.20E-06	large subunit ribosomal protein L24
K02946	0.347884	5.05E-09	1.20E-06	small subunit ribosomal protein S10
K02963	0.347876	5.05E-09	1.20E-06	small subunit ribosomal protein S18
K03282	0.348486	4.73E-09	1.20E-06	large conductance mechanosensitive channel
K03332	-0.34981	4.11E-09	1.20E-06	fructan beta-fructosidase EC:3.2.1.80
K03664	0.349707	4.15E-09	1.20E-06	SsrA-binding protein
K07009	0.349659	4.18E-09	1.20E-06	putative glutamine amidotransferase; putative glutamine amidotransferase
K07752	0.346579	4.58E-09	1.20E-06	carboxypeptidase D EC:3.4.17.22
K10685	0.34576	5.01E-09	1.20E-06	ubiquitin-like 1-activating enzyme E1 B EC:6.3.2.19
K02959	0.346906	5.60E-09	1.29E-06	small subunit ribosomal protein S16
K02520	0.346206	6.03E-09	1.31E-06	translation initiation factor IF-3
K14275	0.344012	6.05E-09	1.31E-06	D-xylonate dehydratase
K02890	0.344827	6.97E-09	1.42E-06	large subunit ribosomal protein L22
K03437	0.345017	6.84E-09	1.42E-06	RNA methyltransferase, TrmH family
K01178	0.341943	7.55E-09	1.48E-06	glucoamylase EC:3.2.1.3
K02878	0.343892	7.69E-09	1.48E-06	large subunit ribosomal protein L16
K06283	0.343426	8.08E-09	1.51E-06	putative DeoR family transcriptional regulator, stage III sporulation protein D
K02518	0.343154	8.31E-09	1.51E-06	translation initiation factor IF-1
K02884	0.342707	8.71E-09	1.54E-06	large subunit ribosomal protein L19
K03687	0.34221	9.18E-09	1.58E-06	molecular chaperone GrpE
K06436	0.341398	9.98E-09	1.67E-06	spore coat assemly protein
K08156	-0.34125	1.01E-08	1.67E-06	MFS transporter, DHA1 family, arabinose polymer transporter
K06402	0.340216	1.13E-08	1.81E-06	stage IV sporulation protein FB EC:3.4.24.-

K07694	0.339015	1.28E-08	0.000002	two-component system, NarL family, vancomycin resistance associated response regulator VraR
K00756	0.338039	1.41E-08	2.15E-06	pyrimidine-nucleoside phosphorylase EC:2.4.2.2
K02950	0.337905	1.43E-08	2.15E-06	small subunit ribosomal protein S12
K09140	0.335223	1.54E-08	2.26E-06	pre-rRNA-processing protein TSR3
K01725	0.334955	1.58E-08	2.27E-06	cyanate lyase EC:4.2.1.104
K02468	0.333699	1.80E-08	2.54E-06	DeoR family transcriptional regulator, glucitol operon repressor
K02939	0.333593	2.23E-08	3.07E-06	large subunit ribosomal protein L9
K01090	0.332703	2.43E-08	3.23E-06	protein phosphatase EC:3.1.3.16
K02931	0.332696	2.44E-08	3.23E-06	large subunit ribosomal protein L5
K08178	-0.33034	2.55E-08	3.32E-06	MFS transporter, SHS family, lactate transporter
K03040	0.331573	2.73E-08	3.49E-06	DNA-directed RNA polymerase subunit alpha EC:2.7.7.6
K00170	0.329474	3.37E-08	3.58E-06	pyruvate ferredoxin oxidoreductase, beta subunit EC:1.2.7.1
K02340	0.330193	3.13E-08	3.58E-06	DNA polymerase III subunit delta EC:2.7.7.7
K02967	0.329737	3.28E-08	3.58E-06	small subunit ribosomal protein S2
K02996	0.329567	3.34E-08	3.58E-06	small subunit ribosomal protein S9
K05808	0.330374	3.08E-08	3.58E-06	putative sigma-54 modulation protein
K06386	0.329465	3.37E-08	3.58E-06	stage II sporulation protein Q
K07148	-0.33067	2.99E-08	3.58E-06	uncharacterized protein; uncharacterized protein
K07650	0.331089	2.86E-08	3.58E-06	two-component system, OmpR family, sensor histidine kinase CssS EC:2.7.13.3
K07678	-0.33093	2.91E-08	3.58E-06	two-component system, NarL family, sensor histidine kinase BarA EC:2.7.13.3
K09835	0.327677	3.34E-08	3.58E-06	prolycopene isomerase EC:5.2.1.13
K11212	0.329573	3.33E-08	3.58E-06	LPPG_FO 2-phospho-L-lactate transferase EC:2.7.8.28
K13281	0.3268	3.65E-08	3.82E-06	UV DNA damage endonuclease EC:3.-.-.-
K14058	0.328074	3.87E-08	3.99E-06	tRNA 2-thiocytidine biosynthesis protein TtcA
K01174	0.327581	4.06E-08	4.07E-06	micrococcal nuclease EC:3.1.31.1
K03625	0.327612	4.05E-08	4.07E-06	N utilization substance protein B
K00125	0.324227	4.73E-08	4.67E-06	formate dehydrogenase, beta subunit EC:1.2.1.2
K02871	0.325204	5.14E-08	4.93E-06	large subunit ribosomal protein L13
K06313	0.325212	5.14E-08	4.93E-06	spore germination protein
K06418	0.323256	5.22E-08	4.93E-06	small acid-soluble spore protein A (major alpha-type SASP)
K00655	0.324635	5.43E-08	0.000005	1-acyl-sn-glycerol-3-phosphate acyltransferase EC:2.3.1.51
K14540	0.32473	5.38E-08	0.000005	ribosome biogenesis GTPase A
K07166	0.32426	5.64E-08	5.12E-06	ACT domain-containing protein; ACT domain-containing protein
K02838	0.323702	5.95E-08	5.24E-06	ribosome recycling factor
K02879	0.323576	6.03E-08	5.24E-06	large subunit ribosomal protein L17
K07447	0.323671	5.97E-08	5.24E-06	putative holliday junction resolvase EC:3.1.-.-
K13746	0.321731	6.08E-08	5.24E-06	carboxynorspermidine dehydrogenase; carboxynorspermidine dehydrogenase
K04480	0.321277	6.36E-08	5.42E-06	methanol---5-hydroxybenzimidazolylcobamide Co-methyltransferase EC:2.1.1.90
K06405	0.322775	6.52E-08	5.49E-06	stage V sporulation protein AC
K07577	0.32047	6.89E-08	5.73E-06	putative mRNA 3-end processing factor; putative mRNA 3-end processing factor
K01408	0.321751	7.20E-08	5.78E-06	insulysin EC:3.4.24.56
K03091	0.321753	7.20E-08	5.78E-06	RNA polymerase sporulation-specific sigma factor
K06438	0.321957	7.06E-08	5.78E-06	similar to stage IV sporulation protein

K03076	0.320751	7.94E-08	6.30E-06	preprotein translocase subunit SecY
K02201	0.318763	8.15E-08	6.39E-06	pantetheine-phosphate adenylyltransferase EC:2.7.7.3
K04483	0.31804	8.75E-08	6.56E-06	DNA repair protein RadA
K08972	0.31983	8.68E-08	6.56E-06	putative membrane protein; putative membrane protein
K09145	0.318215	8.60E-08	6.56E-06	hypothetical protein; hypothetical protein
K14120	0.31812	8.68E-08	6.56E-06	energy-converting hydrogenase B subunit K
K01916	0.317606	9.13E-08	6.69E-06	NAD+ synthase EC:6.3.1.5
K02613	0.317416	9.30E-08	6.69E-06	ring-1,2-phenylacetyl-CoA epoxidase subunit PaaE
K03296	-0.31921	9.21E-08	6.69E-06	hydrophobic/amphiphilic exporter-1 (mainly G- bacteria), HAE1 family
K04074	0.319115	9.29E-08	6.69E-06	cell division initiation protein
K00934	0.316765	9.91E-08	7.05E-06	arginine kinase EC:2.7.3.3
K07699	0.318165	1.02E-07	7.17E-06	two-component system, response regulator, stage 0 sporulation protein A
K03705	0.317968	1.04E-07	7.24E-06	heat-inducible transcriptional repressor
K06881	0.31777	1.06E-07	7.25E-06	phosphoesterase RecJ domain-containing protein; phosphoesterase RecJ domain-containing protein
K06950	0.317738	1.06E-07	7.25E-06	uncharacterized protein; uncharacterized protein
K06295	0.317141	1.12E-07	7.60E-06	spore germination protein KA
K02948	0.314992	1.18E-07	7.74E-06	small subunit ribosomal protein S11
K02992	0.316717	1.17E-07	7.74E-06	small subunit ribosomal protein S7
K03784	0.31676	1.17E-07	7.74E-06	purine-nucleoside phosphorylase EC:2.4.2.1
K00728	0.316454	1.20E-07	7.81E-06	dolichyl-phosphate-mannose-protein mannosyltransferase EC:2.4.1.109
K04769	0.316199	1.23E-07	7.93E-06	AbrB family transcriptional regulator, stage V sporulation protein T
K01462	0.315618	1.30E-07	8.23E-06	peptide deformylase EC:3.5.1.88
K09772	0.315614	1.30E-07	8.23E-06	cell division inhibitor SepF
K03978	0.315493	1.31E-07	8.25E-06	GTP-binding protein
K09118	0.31533	1.34E-07	8.30E-06	hypothetical protein; hypothetical protein
K06286	0.314978	1.38E-07	8.51E-06	septation ring formation regulator
K00564	0.314538	1.44E-07	8.64E-06	16S rRNA (guanine1207-N2)-methyltransferase EC:2.1.1.172
K00995	0.3146	1.43E-07	8.64E-06	CDP-diacylglycerol--glycerol-3-phosphate 3-phosphatidyltransferase EC:2.7.8.5
K03231	0.313039	1.42E-07	8.64E-06	elongation factor 1-alpha
K06401	0.312714	1.47E-07	8.74E-06	stage IV sporulation protein FA
K03429	0.313665	1.56E-07	9.14E-06	1,2-diacylglycerol 3-glucosyltransferase EC:2.4.1.157
K05363	0.313672	1.56E-07	9.14E-06	serine/alanine adding enzyme EC:2.3.2.10
K09138	0.311837	1.60E-07	9.26E-06	hypothetical protein; hypothetical protein
K02906	0.313141	1.64E-07	9.45E-06	large subunit ribosomal protein L3
K09976	0.312827	1.69E-07	9.57E-06	hypothetical protein; hypothetical protein
K13280	0.312897	1.68E-07	9.57E-06	signal peptidase, endoplasmic reticulum-type EC:3.4.-.-
K02356	0.31265	1.72E-07	9.58E-06	elongation factor P
K06944	0.311052	1.72E-07	9.58E-06	ribosome biogenesis GTPase EC:3.6.1.-; ribosome biogenesis GTPase EC:3.6.1.-
K02933	0.312052	1.82E-07	0.00001	large subunit ribosomal protein L6
K03629	0.311892	1.85E-07	1.01E-05	DNA replication and repair protein RecF
K02991	0.310106	1.88E-07	1.02E-05	small subunit ribosomal protein S6e
K15238	0.309999	1.90E-07	1.03E-05	2,5-dichloro-2,5-cyclohexadiene-1,4-diol dehydrogenase 2 EC:1.3.1.-
K03243	0.309703	1.96E-07	1.05E-05	translation initiation factor 5B
K07041	0.309317	2.03E-07	1.08E-05	probable rRNA maturation factor; probable rRNA maturation factor

K06960	0.309902	2.23E-07	1.17E-05	ribosomal RNA assembly protein; ribosomal RNA assembly protein
K03057	0.308163	2.27E-07	1.18E-05	transcription elongation factor
K07015	0.309766	2.25E-07	1.18E-05	putative hydrolases of HD superfamily; putative hydrolases of HD superfamily
K04567	0.309556	2.30E-07	1.18E-05	lysyl-tRNA synthetase, class II EC:6.1.1.6
K06421	0.309391	2.33E-07	0.000012	small acid-soluble spore protein D (minor alpha/beta-type SASP)
K03234	0.307576	2.40E-07	0.000012	elongation factor 2
K06878	0.309142	2.39E-07	0.000012	tRNA-binding protein; tRNA-binding protein
K07562	0.30718	2.49E-07	1.24E-05	nonsense-mediated mRNA decay protein 3
K06399	0.308598	2.51E-07	1.25E-05	stage IV sporulation protein B EC:3.4.21.116
K00760	0.308474	2.54E-07	1.25E-05	hypoxanthine phosphoribosyltransferase EC:2.4.2.8
K09762	0.308224	2.60E-07	1.27E-05	hypothetical protein; hypothetical protein
K11144	0.308199	2.61E-07	1.27E-05	primosomal protein DnaI
K02654	0.307795	2.71E-07	0.000013	leader peptidase (prepilin peptidase) / N-methyltransferase EC:3.4.23.43 2.1.1.-
K06398	0.307437	2.80E-07	1.34E-05	stage IV sporulation protein A
K06407	0.307136	2.88E-07	1.37E-05	stage V sporulation protein AE
K02639	0.305513	2.91E-07	1.38E-05	ferredoxin
K09788	-0.30613	3.15E-07	1.48E-05	hypothetical protein; hypothetical protein
K09765	0.306055	3.18E-07	1.48E-05	hypothetical protein; hypothetical protein
K00621	0.303992	3.35E-07	0.000015	glucosamine-phosphate N-acetyltransferase EC:2.3.1.4
K06024	0.305613	3.31E-07	0.000015	segregation and condensation protein B
K06334	0.305647	3.30E-07	0.000015	spore coat protein JC
K06948	0.305494	3.34E-07	0.000015	ribosome biogenesis GTPase EC:3.6.1.-; ribosome biogenesis GTPase EC:3.6.1.-
K07466	0.304277	3.26E-07	0.000015	replication factor A1
K12574	0.305603	3.31E-07	0.000015	ribonuclease J EC:3.1.-.-
K06333	0.305196	3.44E-07	1.53E-05	spore coat protein JB
K05896	0.305004	3.50E-07	1.55E-05	segregation and condensation protein A
K01781	-0.30475	3.58E-07	1.57E-05	mandelate racemase EC:5.1.2.2
K07313	0.304237	3.75E-07	1.64E-05	serine/threonine protein phosphatase 1 EC:3.1.3.16
K11777	-0.30234	3.91E-07	0.000017	HAD superfamily phosphatase; HAD superfamily phosphatase
K02530	0.301229	4.33E-07	1.87E-05	DeoR family transcriptional regulator, lactose phosphotransferase system repressor
K00555	0.301135	4.37E-07	1.87E-05	tRNA (guanine26-N2/guanine27-N2)-dimethyltransferase EC:2.1.1.215 2.1.1.216
K02904	0.302269	4.49E-07	1.91E-05	large subunit ribosomal protein L29
K02885	0.300565	4.60E-07	1.95E-05	large subunit ribosomal protein L19e
K02921	0.30036	4.69E-07	1.98E-05	large subunit ribosomal protein L37Ae
K02956	0.301333	4.89E-07	2.04E-05	small subunit ribosomal protein S15
K01238	-0.30121	4.94E-07	2.05E-05	purine nucleosidase EC:3.2.2.1
K02664	0.29978	4.95E-07	2.05E-05	type IV pilus assembly protein PilO
K00869	0.299668	5.00E-07	2.05E-05	mevalonate kinase EC:2.7.1.36
K07582	0.299533	5.06E-07	2.07E-05	
K11694	0.299289	5.18E-07	0.000021	peptidoglycan pentaglycine glycine transferase (the second and third glycine) EC:2.3.2.17
K03346	0.300419	5.31E-07	2.14E-05	replication initiation and membrane attachment protein
K02984	0.298943	5.34E-07	2.14E-05	small subunit ribosomal protein S3Ae
K08218	-0.30019	5.42E-07	2.16E-05	MFS transporter, PAT family, beta-lactamase induction signal transducer AmpG

K13693	0.298628	5.50E-07	2.18E-05	glucosyl-3-phosphoglycerate synthase EC:2.4.1.-; glucosyl-3-phosphoglycerate synthase EC:2.4.1.-
K01808	0.298557	5.53E-07	2.18E-05	ribose 5-phosphate isomerase B EC:5.3.1.6
K02770	-0.29989	5.56E-07	2.18E-05	PTS system, fructose-specific IIC component
K01190	-0.29977	5.62E-07	0.000022	beta-galactosidase EC:3.2.1.23
K01758	0.29939	5.82E-07	0.000022	cystathionine gamma-lyase EC:4.4.1.1
K03264	0.298058	5.79E-07	0.000022	translation initiation factor 6
K06012	0.299443	5.79E-07	0.000022	spore protease EC:3.4.24.78
K09779	0.299553	5.73E-07	0.000022	hypothetical protein; hypothetical protein
K11600	0.298049	5.80E-07	0.000022	exosome complex component RRP41
K03307	-0.29925	5.90E-07	2.22E-05	solute_Na+ symporter, SSS family
K01596	0.298637	6.23E-07	2.33E-05	phosphoenolpyruvate carboxykinase (GTP) EC:4.1.1.32
K07067	0.297185	6.27E-07	2.33E-05	DNA integrity scanning protein
K11693	0.298607	6.24E-07	2.33E-05	peptidoglycan pentaglycine glycine transferase (the first glycine) EC:2.3.2.16
K01668	-0.29849	6.31E-07	2.33E-05	tyrosine phenol-lyase EC:4.1.99.2
K03545	0.298265	6.44E-07	2.36E-05	trigger factor
K06994	0.297712	6.76E-07	2.47E-05	putative drug exporter of the RND superfamily; putative drug exporter of the RND superfamily
K03660	0.297485	6.90E-07	0.000025	N-glycosylase/DNA lyase EC:3.2.2.- 4.2.99.18
K01167	0.295425	7.35E-07	2.66E-05	ribonuclease T1 EC:3.1.27.3
K14094	0.294965	7.67E-07	2.76E-05	energy-converting hydrogenase A subunit C
K01875	0.295413	8.29E-07	2.97E-05	seryl-tRNA synthetase EC:6.1.1.11
K11781	0.293701	8.59E-07	3.06E-05	FO synthase subunit 2 EC:2.5.1.77
K09803	0.294885	8.69E-07	3.08E-05	hypothetical protein; hypothetical protein
K03263	0.293492	8.75E-07	3.08E-05	translation initiation factor 5A
K02685	0.293366	8.85E-07	0.000031	DNA primase large subunit EC:2.7.7.-
K13688	0.294642	8.88E-07	0.000031	cyclic beta-1,2-glucan synthetase EC:2.4.1.-
K02481	-0.29442	9.05E-07	3.13E-05	two-component system, NtrC family, response regulator; two-component system, NtrC family, response regulator
K06925	0.294408	9.06E-07	3.13E-05	UPF0079 ATP-binding protein; UPF0079 ATP-binding protein
K05873	0.294242	9.20E-07	3.16E-05	adenylate cyclase, class 2 EC:4.6.1.1
K12553	0.292755	9.34E-07	0.000032	penicillin-binding protein 3 EC:3.4.-.-
K02433	0.293781	9.58E-07	3.23E-05	aspartyl-tRNA(Asn)/glutamyl-tRNA (Gln) amidotransferase subunit A EC:6.3.5.6 6.3.5.7
K02877	0.292477	9.58E-07	3.23E-05	large subunit ribosomal protein L15e
K14081	0.292481	9.58E-07	3.23E-05	methanol corrinoid protein
K02023	0.293685	9.66E-07	3.24E-05	multiple sugar transport system ATP-binding protein
K06943	0.292167	9.85E-07	3.28E-05	nucleolar GTP-binding protein
K03265	0.291792	1.02E-06	3.36E-05	peptide chain release factor subunit 1
K06965	0.291824	1.02E-06	3.36E-05	protein pelota
K06387	0.292923	1.03E-06	0.000034	stage II sporulation protein R
K06174	0.291525	1.04E-06	0.000034	ATP-binding cassette, sub-family E, member 1
K07177	0.292777	1.05E-06	0.000034	PDZ domain-containing protein; PDZ domain-containing protein
K02881	0.292537	1.07E-06	3.46E-05	large subunit ribosomal protein L18
K03124	0.291174	1.08E-06	3.47E-05	transcription initiation factor TFIIB
K01489	0.292343	1.09E-06	3.47E-05	cytidine deaminase EC:3.5.4.5
K02930	0.291059	1.09E-06	3.47E-05	large subunit ribosomal protein L4e
K02236	0.290986	1.09E-06	3.48E-05	leader peptidase (prepilin peptidase) / N-methyltransferase EC:3.4.23.43 2.1.1.-
K00036	-0.29201	1.12E-06	3.53E-05	glucose-6-phosphate 1-dehydrogenase EC:1.1.1.49
K02001	-0.29194	1.13E-06	3.53E-05	glycine betaine/proline transport system permease protein

K06961	0.290695	1.12E-06	3.53E-05	ribosomal RNA assembly protein
K11145	0.291149	1.21E-06	3.77E-05	ribonuclease III family protein EC:3.1.26.-; ribonuclease III family protein EC:3.1.26.-
K06346	0.290762	1.25E-06	3.88E-05	spoIIIJ-associated protein
K06874	0.289251	1.28E-06	3.95E-05	zinc finger protein; zinc finger protein
K04487	0.290443	1.28E-06	3.95E-05	cysteine desulfurase EC:2.8.1.7
K02002	-0.28855	1.36E-06	4.13E-05	glycine betaine/proline transport system substrate-binding protein
K03151	0.289878	1.35E-06	4.13E-05	thiamine biosynthesis protein ThiI
K09942	0.288531	1.36E-06	4.13E-05	hypothetical protein; hypothetical protein
K02982	0.289686	1.37E-06	4.15E-05	small subunit ribosomal protein S3
K01560	0.289608	1.38E-06	4.16E-05	2-haloacid dehalogenase EC:3.8.1.2
K03529	0.289555	1.39E-06	4.16E-05	chromosome segregation protein
K01470	-0.28948	1.39E-06	4.16E-05	creatinine amidohydrolase EC:3.5.2.10
K06385	0.289454	1.40E-06	4.16E-05	stage II sporulation protein P
K06153	0.289365	1.41E-06	4.17E-05	undecaprenyl-diphosphatase EC:3.6.1.27
K01993	-0.28904	1.45E-06	4.24E-05	HlyD family secretion protein
K02250	0.287757	1.45E-06	4.24E-05	competence protein ComK
K06397	0.289104	1.44E-06	4.24E-05	stage III sporulation protein AH
K06423	0.287773	1.45E-06	4.24E-05	small acid-soluble spore protein F (minor alpha/beta-type SASP)
K09769	0.288633	1.50E-06	4.33E-05	hypothetical protein; hypothetical protein
K14112	0.287436	1.50E-06	4.33E-05	energy-converting hydrogenase B subunit C
K03238	0.287211	1.53E-06	4.39E-05	translation initiation factor 2 subunit 2
K14126	0.28716	1.53E-06	4.39E-05	F420-non-reducing hydrogenase subunit A EC:1.12.99.-
K06967	0.288233	1.55E-06	0.000044	tRNA (adenine22-N1)-methyltransferase EC:2.1.1.217
K07055	0.287037	1.55E-06	0.000044	tRNA wybutosine-synthesizing protein 2 EC:2.1.1.-
K00972	0.288159	1.56E-06	4.42E-05	UDP-N-acetylglucosamine pyrophosphorylase EC:2.7.7.23
K02866	0.286796	1.58E-06	4.44E-05	large subunit ribosomal protein L10e
K03471	0.288048	1.58E-06	4.44E-05	ribonuclease HIII EC:3.1.26.4
K01286	0.287819	1.61E-06	4.48E-05	D-alanyl-D-alanine carboxypeptidase EC:3.4.16.4
K02014	-0.2878	1.61E-06	4.48E-05	iron complex outermembrane recepter protein
K03216	0.287618	1.64E-06	4.54E-05	tRNA (cytidine/uridine-2'-O-)-methyltransferase EC:2.1.1.207
K07085	-0.28754	1.65E-06	4.55E-05	putative transport protein; putative transport protein
K03217	0.287257	1.69E-06	4.64E-05	preprotein translocase subunit YidC
K00186	0.285946	1.70E-06	4.67E-05	2-oxoisovalerate ferredoxin oxidoreductase, alpha subunit EC:1.2.7.7
K02434	0.2868	1.76E-06	0.000048	aspartyl-tRNA(Asn)/glutamyl-tRNA (Gln) amidotransferase subunit B EC:6.3.5.6 6.3.5.7
K00558	0.286459	1.81E-06	4.91E-05	DNA (cytosine-5-)-methyltransferase EC:2.1.1.37
K02974	0.285198	1.82E-06	4.92E-05	small subunit ribosomal protein S24e
K06394	0.286345	1.83E-06	4.92E-05	stage III sporulation protein AE
K05970	-0.28616	1.86E-06	4.98E-05	sialate O-acetylesterase EC:3.1.1.53
K03627	0.284829	1.88E-06	5.02E-05	putative transcription factor
K02977	0.284674	1.90E-06	5.04E-05	small subunit ribosomal protein S27Ae
K06981	0.284658	1.90E-06	5.04E-05	pantoate kinase EC:2.7.1.169; pantoate kinase EC:2.7.1.169
K08724	0.285925	1.89E-06	5.04E-05	penicillin-binding protein 2B
K02910	0.284594	1.92E-06	5.05E-05	large subunit ribosomal protein L31e
K02769	-0.28554	1.96E-06	5.13E-05	PTS system, fructose-specific IIB component EC:2.7.1.69
K00021	0.284086	0.000002	0.000052	hydroxymethylglutaryl-CoA reductase (NADPH) EC:1.1.1.34
K00939	0.285272	0.000002	0.000052	adenylate kinase EC:2.7.4.3

K07114	-0.28531	0.000002	0.000052	uncharacterized protein; uncharacterized protein
K06343	0.283978	2.02E-06	0.000052	spore coat protein Y
K08286	0.283982	2.02E-06	0.000052	protein-serine/threonine kinase EC:2.7.11.-
K01889	0.285048	2.04E-06	5.23E-05	phenylalanyl-tRNA synthetase alpha chain EC:6.1.1.20
K00091	0.284691	2.10E-06	0.000053	dihydroflavonol-4-reductase EC:1.1.1.219
K03053	0.283612	2.09E-06	0.000053	DNA-directed RNA polymerase subunit H EC:2.7.7.6
K06344	0.283661	2.08E-06	0.000053	spore coat protein Z
K07078	-0.28475	2.09E-06	0.000053	UPF0755 protein; UPF0755 protein
K09787	0.284723	2.10E-06	0.000053	hypothetical protein; hypothetical protein
K06406	0.284455	2.15E-06	5.36E-05	stage V sporulation protein AD
K12589	0.283285	2.14E-06	5.36E-05	exosome complex component RRP42
K14535	0.283058	2.19E-06	5.45E-05	transcription initiation factor TFIID subunit 9 / adenylate kinase EC:2.7.4.3
K07647	-0.28412	2.21E-06	5.48E-05	two-component system, OmpR family, sensor histidine kinase TorS EC:2.7.13.3
K08310	0.28403	2.22E-06	0.000055	dATP pyrophosphohydrolase EC:3.6.1.-
K03839	-0.28393	2.24E-06	5.53E-05	flavodoxin I
K00943	0.283422	2.34E-06	5.75E-05	dTMP kinase EC:2.7.4.9
K03041	0.281971	2.40E-06	5.86E-05	DNA-directed RNA polymerase subunit A' EC:2.7.7.6
K07573	0.281973	2.40E-06	5.86E-05	exosome complex component CSL4
K05714	0.281917	2.41E-06	5.86E-05	2-hydroxy-6-ketonona-2,4-dienedioic acid hydrolase EC:3.7.1.-
K01961	-0.2826	2.51E-06	6.08E-05	acetyl-CoA carboxylase, biotin carboxylase subunit EC:6.4.1.2 6.3.4.14
K02385	0.282426	2.55E-06	6.12E-05	flagellar protein FlbD
K06328	0.281246	2.55E-06	6.12E-05	spore coat protein E
K07742	0.282417	2.55E-06	6.12E-05	hypothetical protein; hypothetical protein
K07583	0.281174	2.57E-06	6.14E-05	tRNA pseudouridine synthase 10 EC:5.4.99.-
K00399	0.281104	2.59E-06	6.15E-05	methyl-coenzyme M reductase alpha subunit EC:2.8.4.1
K01303	-0.28204	2.63E-06	6.22E-05	acylaminoacyl-peptidase EC:3.4.19.1
K03330	0.280849	2.64E-06	6.22E-05	glutamyl-tRNA(Gln) amidotransferase subunit E EC:6.3.5.7
K03572	0.282034	2.63E-06	6.22E-05	DNA mismatch repair protein MutL
K07579	0.280777	2.66E-06	6.22E-05	putative methylase; putative methylase
K08600	0.281936	2.65E-06	6.22E-05	sortase B EC:3.4.22.70
K00527	0.281761	2.69E-06	6.27E-05	ribonucleoside-triphosphate reductase EC:1.17.4.2
K00833	-0.28174	2.70E-06	6.27E-05	adenosylmethionine-8-amino-7-oxononanoate aminotransferase EC:2.6.1.62
K12306	-0.28052	2.72E-06	0.000063	MFS transporter, FLVCR family, MFS-domain-containing protein 7
K05944	0.280448	2.73E-06	6.31E-05	N-acetylglucosaminyldiphosphoundecaprenol EC:2.4.1.187
K14564	0.280336	2.76E-06	6.35E-05	nucleolar protein 56
K08169	-0.28121	2.82E-06	6.47E-05	MFS transporter, DHA2 family, multidrug resistance protein
K00960	0.279981	2.85E-06	0.000065	DNA-directed RNA polymerase EC:2.7.7.6
K14653	0.279884	2.87E-06	6.51E-05	2-amino-5-formylamino-6-ribosylaminopyrimidin-4(3H)-one 5'-monophosphate deformylase EC:3.5.1.102
K03105	0.279909	2.86E-06	6.51E-05	signal recognition particle subunit SRP19
K00033	-0.28061	2.97E-06	6.69E-05	6-phosphogluconate dehydrogenase EC:1.1.1.44
K06393	0.280621	2.96E-06	6.69E-05	stage III sporulation protein AD
K01881	0.280533	2.99E-06	6.71E-05	prolyl-tRNA synthetase EC:6.1.1.15
K06348	0.280325	3.04E-06	0.000068	sporulation inhibitor KapD
K03042	0.278898	3.12E-06	6.94E-05	DNA-directed RNA polymerase subunit A'' EC:2.7.7.6
K04799	0.278929	3.11E-06	6.94E-05	flap endonuclease-1 EC:3.-.-.-

K07533	0.279673	3.21E-06	7.12E-05	foldase protein PrsA EC:5.2.1.8
K00024	-0.27958	3.23E-06	7.13E-05	malate dehydrogenase EC:1.1.1.37
K02668	-0.27962	3.22E-06	7.13E-05	two-component system, NtrC family, sensor histidine kinase PilS EC:2.7.13.3
K03101	0.279549	3.24E-06	7.13E-05	signal peptidase II EC:3.4.23.36
K01048	0.279416	3.28E-06	7.18E-05	lysophospholipase EC:3.1.1.5
K00402	0.278172	3.32E-06	7.24E-05	methyl-coenzyme M reductase gamma subunit EC:2.8.4.1
K09747	0.279239	3.33E-06	7.24E-05	hypothetical protein; hypothetical protein
K06932	0.278057	3.35E-06	7.27E-05	23S rRNA (adenine2503-C2)-methyltransferase EC:2.1.1.192; 23S rRNA (adenine2503-C2)-methyltransferase EC:2.1.1.192
K06863	0.277839	3.41E-06	7.38E-05	5-formaminoimidazole-4-carboxamide-1-(beta)-D-ribofuranosyl 5'-monophosphate synthetase EC:6.3.4.-
K00169	0.278752	3.46E-06	7.47E-05	pyruvate ferredoxin oxidoreductase, alpha subunit EC:1.2.7.1
K03550	0.278601	3.51E-06	7.54E-05	holliday junction DNA helicase RuvA EC:3.6.4.12
K00172	0.278407	3.57E-06	7.64E-05	pyruvate ferredoxin oxidoreductase, gamma subunit EC:1.2.7.1
K00204	0.277176	3.61E-06	7.68E-05	formylmethanofuran dehydrogenase subunit H EC:1.2.99.5
K00974	0.278301	3.60E-06	7.68E-05	tRNA nucleotidyltransferase (CCA-adding enzyme) EC:2.7.7.72 3.1.3.- 3.1.4.-
K02966	0.277092	3.63E-06	7.71E-05	small subunit ribosomal protein S19e
K08384	0.277865	3.73E-06	0.000079	stage V sporulation protein D (sporulation-specific penicillin-binding protein)
K00836	0.277736	3.77E-06	7.93E-05	diaminobutyrate-2-oxoglutarate transaminase EC:2.6.1.76
K02995	0.276655	3.77E-06	7.93E-05	small subunit ribosomal protein S8e
K04070	0.277623	3.80E-06	7.98E-05	putative pyruvate formate lyase activating enzyme EC:1.97.1.4
K09955	-0.27752	3.84E-06	8.03E-05	hypothetical protein; hypothetical protein
K01028	0.276361	3.86E-06	8.05E-05	3-oxoacid CoA-transferase subunit A EC:2.8.3.5
K01641	0.276245	3.90E-06	8.05E-05	hydroxymethylglutaryl-CoA synthase EC:2.3.3.10
K14057	0.276228	3.91E-06	8.05E-05	LysR family transcriptional regulator, regulator of abg operon
K14106	0.276325	3.87E-06	8.05E-05	energy-converting hydrogenase A subunit O
K15045	0.276255	3.90E-06	8.05E-05	radical S-adenosyl methionine domain-containing protein 2
K02912	0.275908	4.01E-06	8.24E-05	large subunit ribosomal protein L32e
K01857	-0.27544	4.17E-06	8.54E-05	3-carboxy-cis,cis-muconate cycloisomerase EC:5.5.1.2
K00134	0.276394	4.21E-06	0.000086	glyceraldehyde 3-phosphate dehydrogenase EC:1.2.1.12
K03466	0.276364	4.22E-06	0.000086	DNA segregation ATPase FtsK/SpoIIIE, S-DNA-T family
K07795	-0.2763	4.24E-06	8.62E-05	putative tricarboxylic transport membrane protein
K01868	0.276232	4.27E-06	8.64E-05	threonyl-tRNA synthetase EC:6.1.1.3
K10702	0.275066	4.30E-06	8.69E-05	2-hydroxy-6-oxohepta-2,4-dienoate hydroxylase EC:3.7.1.-
K08325	-0.27606	4.33E-06	8.69E-05	NADP-dependent alcohol dehydrogenase EC:1.1.-.-
K10747	0.274992	4.33E-06	8.69E-05	DNA ligase 1 EC:6.5.1.1
K01361	0.275721	4.45E-06	0.000088	lactocepin EC:3.4.21.96
K02357	0.275812	4.42E-06	0.000088	elongation factor Ts
K02987	0.274656	4.45E-06	0.000088	small subunit ribosomal protein S4e
K03110	0.275763	4.44E-06	0.000088	fused signal recognition particle receptor
K07707	0.275813	4.42E-06	0.000088	two-component system, AgrA family, response regulator AgrA
K07978	0.274588	4.48E-06	8.83E-05	GntR family transcriptional regulator
K04068	0.27555	4.51E-06	8.88E-05	anaerobic ribonucleoside-triphosphate reductase activating protein EC:1.97.1.4

K07135	0.27428	4.59E-06	0.00009	molybdenum cofactor cytidylyltransferase EC:2.7.7.76; molybdenum cofactor cytidylyltransferase EC:2.7.7.76
K02086	0.27513	4.67E-06	9.11E-05	DNA replication protein
K04795	0.274102	4.66E-06	9.11E-05	fibrillarin-like pre-rRNA processing protein
K00936	-0.27502	4.71E-06	9.17E-05	polyphosphate kinase EC:2.7.4.1
K03679	0.273858	4.76E-06	9.17E-05	exosome complex component RRP4
K04798	0.273888	4.75E-06	9.17E-05	prefoldin beta subunit
K09165	0.27387	4.75E-06	9.17E-05	hypothetical protein; hypothetical protein
K03242	0.273739	4.81E-06	9.21E-05	translation initiation factor 2 subunit 3
K12999	0.274787	4.81E-06	9.21E-05	glucosyltransferase EC:2.4.1.-
K02926	0.2746	4.88E-06	9.33E-05	large subunit ribosomal protein L4
K00319	0.273129	5.05E-06	9.58E-05	methylenetetrahydromethanopterin dehydrogenase EC:1.5.99.9
K03047	0.273162	5.04E-06	9.58E-05	DNA-directed RNA polymerase subunit D EC:2.7.7.6
K15024	0.274183	5.05E-06	9.58E-05	propanediol utilization protein; propanediol utilization protein
K09024	0.274036	5.11E-06	9.66E-05	flavin reductase EC:1.5.1.-
K01922	0.272902	5.15E-06	9.69E-05	phosphopantothenate-cysteine ligase EC:6.3.2.5
K03387	-0.27395	5.15E-06	9.69E-05	alkyl hydroperoxide reductase subunit F EC:1.6.4.-
K11062	0.273848	5.19E-06	9.73E-05	probable enterotoxin D; probable enterotoxin D
K06915	0.273798	5.21E-06	9.75E-05	tRNA 2-selenouridine synthase EC:2.9.1.-; tRNA 2-selenouridine synthase EC:2.9.1.-
K01928	0.273683	5.26E-06	9.81E-05	UDP-N-acetylmuramoyl-L-alanyl-D-glutamate--2,6-diaminopimelate ligase EC:6.3.2.13
K08722	0.273492	5.34E-06	9.91E-05	5'-nucleotidase EC:3.1.3.5
K10834	0.273515	5.33E-06	9.91E-05	heme-transporting ATPase EC:3.6.3.41
K12820	0.272388	5.37E-06	9.94E-05	pre-mRNA-splicing factor ATP-dependent RNA helicase DHX15/PRP43 EC:3.6.4.13
K01890	0.273317	5.42E-06	0.0001	phenylalanyl-tRNA synthetase beta chain EC:6.1.1.20
K13487	0.272179	5.47E-06	0.000101	methyl-accepting chemotaxis protein WspA
K00401	0.271815	5.63E-06	0.000103	methyl-coenzyme M reductase beta subunit EC:2.8.4.1
K00951	0.272773	5.66E-06	0.000104	GTP pyrophosphokinase EC:2.7.6.5
K01785	-0.27169	5.69E-06	0.000104	aldose 1-epimerase EC:5.1.3.3
K11665	0.271669	5.70E-06	0.000104	DNA helicase INO80 EC:3.6.4.12
K01449	0.272625	5.73E-06	0.000104	N-acetylmuramoyl-L-alanine amidase EC:3.5.1.28
K03585	-0.2725	5.79E-06	0.000105	membrane fusion protein
K00187	0.270948	6.05E-06	0.000109	2-oxoisovalerate ferredoxin oxidoreductase, beta subunit EC:1.2.7.7
K03931	-0.27172	6.17E-06	0.000111	putative isomerase
K10725	0.27074	6.15E-06	0.000111	archaeal cell division control protein 6
K05586	-0.27075	6.15E-06	0.000111	bidirectional NiFe hydrogenase diaphorase subunit EC:1.6.5.3
K00326	0.270575	6.24E-06	0.000112	cytochrome-b5 reductase EC:1.6.2.2
K09758	-0.27123	6.42E-06	0.000114	aspartate 4-decarboxylase EC:4.1.1.12
K12292	0.270172	6.45E-06	0.000115	ATP-binding cassette, subfamily C, bacterial, competence factor transporting protein EC:3.4.22.-
K04802	0.269967	6.56E-06	0.000116	proliferating cell nuclear antigen
K08167	0.269985	6.55E-06	0.000116	MFS transporter, DHA2 family, methyl viologen resistance protein SmvA
K06390	0.270878	6.60E-06	0.000117	stage III sporulation protein AA
K02601	0.270801	6.64E-06	0.000117	transcriptional antiterminator NusG
K02005	-0.27067	6.72E-06	0.000118	HlyD family secretion protein
K09720	0.269647	6.73E-06	0.000118	hypothetical protein; hypothetical protein
K02338	0.270544	6.78E-06	0.000119	DNA polymerase III subunit beta EC:2.7.7.7

K02069	-0.27048	6.82E-06	0.000119	putative ABC transport system permease protein
K07142	0.269331	6.90E-06	0.00012	UPF0148 protein; UPF0148 protein
K05715	0.269063	7.06E-06	0.000122	2-phosphoglycerate kinase EC:2.7.2.-
K01463	0.269718	7.25E-06	0.000125	dihydropyrimidinase EC:3.5.2.2
K01783	0.269739	7.24E-06	0.000125	ribulose-phosphate 3-epimerase EC:5.1.3.1
K03624	0.269645	7.29E-06	0.000126	transcription elongation factor GreA
K02566	0.269544	7.35E-06	0.000126	NagD protein
K02652	0.269429	7.42E-06	0.000126	type IV pilus assembly protein PilB
K03595	0.269374	7.45E-06	0.000126	GTP-binding protein Era
K03798	0.269405	7.43E-06	0.000126	cell division protease FtsH EC:3.4.24.-
K06412	0.269379	7.45E-06	0.000126	stage V sporulation protein G
K14109	0.268462	7.41E-06	0.000126	energy-converting hydrogenase A subunit R
K00798	-0.26932	7.48E-06	0.000127	cob(I)alamin adenosyltransferase EC:2.5.1.17
K02238	0.269261	7.52E-06	0.000127	competence protein ComEC
K03433	0.268194	7.57E-06	0.000128	proteasome beta subunit EC:3.4.25.1
K06378	0.269081	7.63E-06	0.000128	stage II sporulation protein AA (anti-sigma F factor antagonist)
K00672	0.267942	7.73E-06	0.00013	formylmethanofuran--tetrahydromethanopterin N-formyltransferase EC:2.3.1.101
K03700	0.26887	7.76E-06	0.00013	recombination protein U
K07072	0.267864	7.78E-06	0.00013	UPF0755 protein; UPF0755 protein
K01959	0.267742	7.86E-06	0.00013	pyruvate carboxylase subunit A EC:6.4.1.1
K07002	0.267773	7.84E-06	0.00013	glutamine amidotransferase; glutamine amidotransferase
K07719	-0.26872	7.85E-06	0.00013	two-component system, response regulator YcbB
K10819	-0.26871	7.86E-06	0.00013	histidine kinase
K03501	0.268645	7.90E-06	0.00013	16S rRNA (guanine527-N7)-methyltransferase EC:2.1.1.170
K09812	0.268515	7.98E-06	0.000131	cell division transport system ATP-binding protein
K06113	-0.26847	8.01E-06	0.000131	arabinan endo-1,5-alpha-L-arabinosidase EC:3.2.1.99
K11621	0.26834	8.10E-06	0.000132	lia operon protein LiaG
K01622	0.267329	8.12E-06	0.000133	fructose 1,6-bisphosphate aldolase/phosphatase EC:4.1.2.13 3.1.3.11; fructose 1,6-bisphosphate aldolase/phosphatase EC:4.1.2.13 3.1.3.11
K04758	0.268216	8.18E-06	0.000133	ferrous iron transport protein A
K07584	0.268207	8.18E-06	0.000133	hypothetical protein; hypothetical protein
K06409	0.268173	8.20E-06	0.000133	stage V sporulation protein B
K13490	0.267171	8.23E-06	0.000133	two-component system, chemotaxis family, sensor histidine kinase and response regulator WspE
K03470	0.268047	8.29E-06	0.000134	ribonuclease HII EC:3.1.26.4
K07404	-0.26798	8.33E-06	0.000134	6-phosphogluconolactonase EC:3.1.1.31
K01880	0.267792	8.46E-06	0.000136	glycyl-tRNA synthetase EC:6.1.1.14
K01960	-0.26769	8.53E-06	0.000136	pyruvate carboxylase subunit B EC:6.4.1.1
K02683	0.26672	8.53E-06	0.000136	DNA primase EC:2.7.7.-
K03696	0.26769	8.53E-06	0.000136	ATP-dependent Clp protease ATP-binding subunit ClpC
K07463	0.266725	8.53E-06	0.000136	archaea-specific RecJ-like exonuclease; archaea-specific RecJ-like exonuclease
K01539	0.266627	8.60E-06	0.000136	sodium/potassium-transporting ATPase subunit alpha EC:3.6.3.9
K13039	0.26659	8.62E-06	0.000137	sulfopyruvate decarboxylase subunit beta EC:4.1.1.79; sulfopyruvate decarboxylase subunit beta EC:4.1.1.79
K03232	0.266469	8.71E-06	0.000138	elongation factor 1-beta
K04488	0.267376	8.74E-06	0.000138	nitrogen fixation protein NifU and related proteins
K11749	0.267342	8.77E-06	0.000138	regulator of sigma E protease EC:3.4.24.-

K07124	0.267145	8.91E-06	0.00014	5-hydroxyisourate hydrolase EC:3.5.2.17; 5-hydroxyisourate hydrolase EC:3.5.2.17
K05995	0.266842	9.12E-06	0.000143	dipeptidase E EC:3.4.13.21
K07260	0.266799	9.15E-06	0.000143	D-alanyl-D-alanine carboxypeptidase EC:3.4.16.4
K07134	0.265814	9.18E-06	0.000143	molybdenum cofactor cytidylyltransferase EC:2.7.7.76; molybdenum cofactor cytidylyltransferase EC:2.7.7.76
K03044	0.265697	9.27E-06	0.000144	DNA-directed RNA polymerase subunit B' EC:2.7.7.6
K07387	-0.26664	9.27E-06	0.000144	putative metalloprotease EC:3.4.24.-; putative metalloprotease EC:3.4.24.-
K07558	0.265637	9.31E-06	0.000144	tRNA nucleotidyltransferase (CCA-adding enzyme) EC:2.7.7.72; tRNA nucleotidyltransferase (CCA-adding enzyme) EC:2.7.7.72
K07007	0.266484	9.39E-06	0.000145	glutamine amidotransferase; glutamine amidotransferase
K03287	-0.26613	9.65E-06	0.000148	outer membrane factor, OMF family
K03697	0.266178	9.62E-06	0.000148	ATP-dependent Clp protease ATP-binding subunit ClpE
K07089	0.266128	9.65E-06	0.000148	lipopolysaccharide export system permease protein; lipopolysaccharide export system permease protein
K11293	0.266126	9.66E-06	0.000148	protein HIRA/HIR1
K06969	0.266091	9.68E-06	0.000148	23S rRNA (cytosine1962-C5)-methyltransferase EC:2.1.1.191
K06862	0.265102	9.72E-06	0.000148	energy-converting hydrogenase B subunit Q
K03051	0.264972	9.82E-06	0.000149	DNA-directed RNA polymerase subunit F EC:2.7.7.6
K01187	-0.26588	9.85E-06	0.000149	alpha-glucosidase EC:3.2.1.20
K06426	0.264875	9.90E-06	0.00015	small acid-soluble spore protein I (minor)
K11260	0.264721	0.00001	0.000151	formylmethanofuran dehydrogenase subunit G EC:1.2.99.5
K07170	-0.2654	1.02E-05	0.000154	GAF domain-containing protein; GAF domain-containing protein
K11630	0.265296	1.03E-05	0.000155	two-component system, OmpR family, bacitracin resistance response regulator BceR
K00927	0.265179	1.04E-05	0.000156	phosphoglycerate kinase EC:2.7.2.3
K14105	0.264039	1.06E-05	0.000158	energy-converting hydrogenase A subunit N
K02283	0.264775	1.07E-05	0.00016	pilus assembly protein CpaF
K03657	0.264802	1.07E-05	0.00016	DNA helicase II / ATP-dependent DNA helicase PcrA EC:3.6.4.12
K01554	0.2645	0.000011	0.000163	fumarylacetoacetase EC:3.7.1.2
K03702	0.264487	0.000011	0.000163	excinuclease ABC subunit B
K03832	-0.26447	0.000011	0.000163	periplasmic protein TonB
K06383	0.264393	0.000011	0.000164	stage II sporulation protein GA (sporulation sigma-E factor processing peptidase) EC:3.4.23.-
K02322	0.263193	1.13E-05	0.000167	DNA polymerase II large subunit EC:2.7.7.7
K02484	-0.26398	1.14E-05	0.000168	two-component system, OmpR family, sensor kinase EC:2.7.13.3; two-component system, OmpR family, sensor kinase EC:2.7.13.3
K00783	0.263924	1.15E-05	0.000169	23S rRNA (pseudouridine1915-N3)-methyltransferase EC:2.1.1.177
K03715	0.263887	1.15E-05	0.000169	1,2-diacylglycerol 3-beta-galactosyltransferase EC:2.4.1.46
K07713	-0.26385	1.16E-05	0.000169	two-component system, NtrC family, response regulator HydG
K01876	0.26375	1.17E-05	0.00017	aspartyl-tRNA synthetase EC:6.1.1.12
K06305	0.262604	1.19E-05	0.000173	spore germination protein Q
K07159	0.262593	1.19E-05	0.000173	UPF0271 protein; UPF0271 protein
K02968	0.263399	0.000012	0.000174	small subunit ribosomal protein S20
K00203	0.262347	1.21E-05	0.000175	formylmethanofuran dehydrogenase subunit D EC:1.2.99.5

K01134	-0.2632	1.22E-05	0.000176	arylsulfatase A EC:3.1.6.8
K01482	0.263051	1.23E-05	0.000177	dimethylargininase EC:3.5.3.18
K07099	0.262965	1.24E-05	0.000178	N-acetylmuramic acid 6-phosphate etherase EC:4.2.1.126; N-acetylmuramic acid 6-phosphate etherase EC:4.2.1.126
K13598	-0.26296	1.24E-05	0.000178	two-component system, NtrC family, nitrogen regulation sensor histidine kinase NtrY EC:2.7.13.3
K07778	0.262735	1.26E-05	0.000181	two-component system, NarL family, sensor histidine kinase DesK EC:2.7.13.3
K03166	0.261733	1.27E-05	0.000182	DNA topoisomerase VI subunit A EC:5.99.1.3
K06996	-0.2616	1.28E-05	0.000183	phospholipase/carboxylesterase; phospholipase/carboxylesterase
K06970	-0.26153	0.000013	0.000184	23S rRNA (adenine1618-N6)-methyltransferase EC:2.1.1.181
K06392	0.262386	0.000013	0.000184	stage III sporulation protein AC
K01409	0.262255	0.000013	0.000186	O-sialoglycoprotein endopeptidase EC:3.4.24.57
K02883	0.261303	1.32E-05	0.000186	large subunit ribosomal protein L18e
K01342	0.262054	1.33E-05	0.000187	subtilisin EC:3.4.21.62
K11616	-0.26203	1.33E-05	0.000187	malate_Na+ symporter
K04107	-0.26118	1.33E-05	0.000187	4-hydroxybenzoyl-CoA reductase subunit gamma EC:1.3.7.9
K14086	0.261096	1.34E-05	0.000187	ech hydrogenase subunit A
K02825	0.261923	1.34E-05	0.000188	pyrimidine operon attenuation protein / uracil phosphoribosyltransferase EC:2.4.2.9
K07060	0.261015	1.35E-05	0.000188	UPF0271 protein; UPF0271 protein
K07799	-0.26195	1.34E-05	0.000188	putative multidrug efflux transporter MdtA
K02874	0.261854	1.35E-05	0.000188	large subunit ribosomal protein L14
K14093	0.260926	1.35E-05	0.000188	energy-converting hydrogenase A subunit B
K00648	-0.26171	1.37E-05	0.000189	3-oxoacyl-acyl-carrier-protein synthase III EC:2.3.1.180
K00400	0.26077	1.37E-05	0.00019	methyl coenzyme M reductase system, component A2; methyl coenzyme M reductase system, component A2
K13489	0.260716	1.38E-05	0.00019	chemotaxis-related protein WspD
K01635	0.261499	1.39E-05	0.000191	tagatose 1,6-diphosphate aldolase EC:4.1.2.40
K01425	-0.26147	0.000014	0.000191	glutaminase EC:3.5.1.2
K05366	0.261332	0.000014	0.000193	penicillin-binding protein 1A EC:2.4.1.- 3.4.-.-
K00196	0.260402	1.41E-05	0.000193	carbon-monoxide dehydrogenase iron sulfur subunit
K14123	0.260299	1.42E-05	0.000194	energy-converting hydrogenase B subunit N
K00942	0.2608	1.47E-05	0.0002	guanylate kinase EC:2.7.4.8
K01130	-0.26077	1.47E-05	0.0002	arylsulfatase EC:3.1.6.1
K08971	0.259689	0.000015	0.000203	putative membrane protein; putative membrane protein
K00847	-0.2605	0.000015	0.000204	fructokinase EC:2.7.1.4
K02889	0.259553	0.000015	0.000204	large subunit ribosomal protein L21e
K01626	0.260233	1.53E-05	0.000207	3-deoxy-7-phosphoheptulonate synthase EC:2.5.1.54
K03237	0.259172	1.55E-05	0.00021	translation initiation factor 2 subunit 1
K07094	0.258986	1.58E-05	0.000212	putative glycerol-1-phosphate prenyltransferase EC:2.5.1.-
K07791	-0.25979	1.59E-05	0.000213	anaerobic C4-dicarboxylate transporter DcuA
K07793	-0.25967	0.000016	0.000214	putative tricarboxylic transport membrane protein
K10852	-0.25948	1.62E-05	0.000217	isopenicillin-N N-acyltransferase EC:2.3.1.164
K00578	0.258558	1.63E-05	0.000217	tetrahydromethanopterin S-methyltransferase subunit B EC:2.1.1.86
K02013	-0.2594	1.63E-05	0.000217	iron complex transport system ATP-binding protein EC:3.6.3.34
K14121	0.258555	1.63E-05	0.000217	energy-converting hydrogenase B subunit L
K00586	0.258375	1.65E-05	0.000219	diphthine synthase EC:2.1.1.98

K01994	0.258352	1.66E-05	0.000219	LuxR family transcriptional regulator, transcriptional regulator of spore coat protein
K03588	0.259269	1.65E-05	0.000219	cell division protein FtsW
K07030	0.259205	1.66E-05	0.000219	D-glycero-alpha-D-manno-heptose-7-phosphate kinase EC:2.7.1.168; D-glycero-alpha-D-manno-heptose-7-phosphate kinase EC:2.7.1.168
K09119	0.258118	1.69E-05	0.000222	hypothetical protein; hypothetical protein
K02896	0.258111	1.69E-05	0.000222	large subunit ribosomal protein L24e
K04112	0.258934	0.000017	0.000222	benzoyl-CoA reductase subunit EC:1.3.7.8
K01991	-0.25881	0.000017	0.000224	polysaccharide export outer membrane protein
K02242	0.258797	1.71E-05	0.000224	competence protein ComFC
K01057	-0.25868	1.73E-05	0.000225	6-phosphogluconolactonase EC:3.1.1.31
K02834	0.25857	1.74E-05	0.000227	ribosome-binding factor A
K00579	0.257499	1.77E-05	0.00023	tetrahydromethanopterin S-methyltransferase subunit C EC:2.1.1.86
K08259	0.258224	1.79E-05	0.000232	lysostaphin EC:3.4.24.75
K07575	0.257124	1.82E-05	0.000236	PUA domain protein; PUA domain protein
K14297	0.257049	1.83E-05	0.000237	nuclear pore complex protein Nup98-Nup96
K00200	0.256997	1.84E-05	0.000238	formylmethanofuran dehydrogenase subunit A EC:1.2.99.5
K07244	0.25692	1.85E-05	0.000238	mgtE-like transporter; mgtE-like transporter
K14128	0.256911	1.85E-05	0.000238	F420-non-reducing hydrogenase subunit G EC:1.12.99.-
K00697	-0.25759	1.88E-05	0.000241	trehalose 6-phosphate synthase EC:2.4.1.15
K09153	0.25756	1.88E-05	0.000241	hypothetical protein; hypothetical protein
K01867	0.257445	0.000019	0.000242	tryptophanyl-tRNA synthetase EC:6.1.1.2
K02441	0.25746	0.000019	0.000242	GlpG protein
K08979	0.256538	0.000019	0.000243	putative membrane protein; putative membrane protein
K02979	0.256456	1.92E-05	0.000244	small subunit ribosomal protein S28e
K09704	-0.2573	1.92E-05	0.000244	hypothetical protein; hypothetical protein
K14155	-0.25724	1.93E-05	0.000244	cystathione beta-lyase EC:4.4.1.8
K02469	0.257202	1.93E-05	0.000245	DNA gyrase subunit A EC:5.99.1.3
K01552	0.257143	1.94E-05	0.000245	myosin ATPase EC:3.6.4.1
K11780	0.256285	1.95E-05	0.000245	FO synthase subunit 1 EC:2.5.1.77
K00571	0.25681	0.00002	0.000249	site-specific DNA-methyltransferase (adenine-specific) EC:2.1.1.72
K01574	0.255968	0.00002	0.000249	acetoacetate decarboxylase EC:4.1.1.4
K06041	-0.25679	0.00002	0.000249	arabinose-5-phosphate isomerase EC:5.3.1.13
K13950	0.256854	1.99E-05	0.000249	para-aminobenzoate synthetase EC:2.6.1.85
K14054	0.255946	0.00002	0.000249	protein MpaA
K00004	0.256666	2.02E-05	0.000251	(R,R)-butanediol dehydrogenase / diacetyl reductase EC:1.1.1.4 1.1.1.303
K01492	-0.25653	2.04E-05	0.000251	phosphoribosylaminoimidazolecarboxamide formyltransferase EC:2.1.2.3
K02929	0.255741	2.03E-05	0.000251	large subunit ribosomal protein L44e
K03581	0.256565	2.03E-05	0.000251	exodeoxyribonuclease V alpha subunit EC:3.1.11.5
K06045	0.255708	2.03E-05	0.000251	squalene-hopene cyclase EC:5.4.99.17 4.2.1.129
K07574	0.25655	2.03E-05	0.000251	RNA-binding protein
K02000	-0.25626	2.08E-05	0.000256	glycine betaine/proline transport system ATP-binding protein EC:3.6.3.32
K02495	0.256251	2.08E-05	0.000256	oxygen-independent coproporphyrinogen III oxidase EC:1.3.99.22
K06140	0.255425	2.08E-05	0.000256	regulator of nucleoside diphosphate kinase
K03502	0.256227	2.08E-05	0.000256	DNA polymerase V
K08602	0.256181	0.000021	0.000256	oligoendopeptidase F EC:3.4.24.-

K07561	0.255296	0.000021	0.000257	diphthamide synthase subunit DPH2
K00584	0.25522	2.11E-05	0.000258	tetrahydromethanopterin S-methyltransferase subunit H EC:2.1.1.86
K01308	0.255944	2.13E-05	0.000259	g-D-glutamyl-meso-diaminopimelate peptidase EC:3.4.19.11
K07033	0.255932	2.13E-05	0.000259	inner membrane protein; inner membrane protein
K14441	0.255929	2.13E-05	0.000259	ribosomal protein S12 methylthiotransferase EC:2.-.-.-
K07564	0.254984	2.15E-05	0.000261	
K02916	0.254908	2.16E-05	0.000262	large subunit ribosomal protein L35
K07581	0.254584	2.22E-05	0.000268	hypothetical protein; hypothetical protein
K01478	0.255357	2.23E-05	0.000268	arginine deiminase EC:3.5.3.6
K01592	0.254549	2.22E-05	0.000268	tyrosine decarboxylase EC:4.1.1.25
K03438	0.255289	2.24E-05	0.000269	16S rRNA (cytosine1402-N4)-methyltransferase EC:2.1.1.199
K09722	0.254391	2.25E-05	0.00027	4-phosphopantoate---beta-alanine ligase EC:6.3.2.36; 4-phosphopantoate---beta-alanine ligase EC:6.3.2.36
K10826	0.254381	2.25E-05	0.00027	Fe3+-transporting ATPase EC:3.6.3.30
K07284	0.255141	2.26E-05	0.00027	sortase A EC:3.4.22.70
K07178	0.254237	2.28E-05	0.000272	RIO kinase 1 EC:2.7.11.1
K07042	0.254974	0.000023	0.000273	probable rRNA maturation factor
K08783	0.254094	0.000023	0.000274	extracellular matrix protein 14 EC:3.4.17.-
K08974	0.25475	2.33E-05	0.000277	putative membrane protein; putative membrane protein
K11441	-0.25385	2.35E-05	0.000278	dehydrogluconokinase EC:2.7.1.13
K00809	0.253809	2.35E-05	0.000278	deoxyhypusine synthase EC:2.5.1.46
K14111	0.253569	0.000024	0.000283	energy-converting hydrogenase B subunit B
K00577	0.253475	2.42E-05	0.000284	tetrahydromethanopterin S-methyltransferase subunit A EC:2.1.1.86
K07103	0.253443	2.42E-05	0.000285	N-acetylmuramic acid 6-phosphate etherase EC:4.2.1.126; N-acetylmuramic acid 6-phosphate etherase EC:4.2.1.126
K04789	0.253342	2.44E-05	0.000286	mycobactin peptide synthetase MbtE
K00097	-0.25406	2.46E-05	0.000287	4-hydroxythreonine-4-phosphate dehydrogenase EC:1.1.1.262
K02479	-0.25403	2.46E-05	0.000287	two-component system, NarL family, response regulator; two-component system, NarL family, response regulator
K07775	0.254029	2.46E-05	0.000287	two-component system, OmpR family, response regulator ResD
K09163	0.254091	2.45E-05	0.000287	hypothetical protein; hypothetical protein
K14097	0.253259	2.46E-05	0.000287	energy-converting hydrogenase A subunit F
K01621	0.253825	0.000025	0.000291	phosphoketolase EC:4.1.2.9
K13275	0.253733	2.52E-05	0.000292	major intracellular serine protease EC:3.4.21.-
K03789	0.253671	2.53E-05	0.000293	ribosomal-protein-alanine N-acetyltransferase EC:2.3.1.128
K07238	0.253628	2.54E-05	0.000293	zinc transporter, ZIP family
K02793	-0.25355	2.55E-05	0.000295	PTS system, mannose-specific IIA component EC:2.7.1.69
K09497	0.252596	2.58E-05	0.000298	T-complex protein 1 subunit epsilon
K00616	-0.25334	0.000026	0.000298	transaldolase EC:2.2.1.2
K06379	0.253305	0.000026	0.000299	stage II sporulation protein AB (anti-sigma F factor) EC:2.7.11.1
K02962	0.252441	2.61E-05	0.0003	small subunit ribosomal protein S17e
K08735	0.252293	2.64E-05	0.000303	DNA mismatch repair protein MSH2
K01225	0.252909	2.68E-05	0.000306	cellulose 1,4-beta-cellobiosidase EC:3.2.1.91
K06384	0.252894	2.68E-05	0.000306	stage II sporulation protein M
K03439	0.252758	0.000027	0.000308	tRNA (guanine-N7-)-methyltransferase EC:2.1.1.33
K04796	0.251934	2.72E-05	0.000308	small nuclear ribonucleoprotein

K14103	0.251947	2.71E-05	0.000308	energy-converting hydrogenase A subunit L
K09730	0.251639	2.78E-05	0.000315	hypothetical protein; hypothetical protein
K00773	0.252359	0.000028	0.000316	queuine tRNA-ribosyltransferase EC:2.4.2.29
K02503	0.252222	2.82E-05	0.000318	Hit-like protein involved in cell-cycle regulation
K02527	-0.25221	2.82E-05	0.000318	3-deoxy-D-manno-octulosonic-acid transferase EC:2.4.99.12 2.4.99.13 2.4.99.14 2.4.99.15
K00603	-0.25211	2.85E-05	0.00032	glutamate formiminotransferase EC:2.1.2.5
K09696	0.252095	2.85E-05	0.00032	sodium transport system permease protein
K03795	0.251219	2.87E-05	0.000322	sirohydrochlorin cobaltochelatase EC:4.99.1.3
K04043	0.251995	2.87E-05	0.000322	molecular chaperone DnaK
K09495	0.251186	2.87E-05	0.000322	T-complex protein 1 subunit gamma
K13292	0.251942	2.88E-05	0.000322	phosphatidylglycerol_prolipoprotein diacylglycerol transferase EC:2.-.-.-
K13730	0.25173	2.93E-05	0.000326	internalin A
K06212	0.251699	2.93E-05	0.000327	formate transporter
K00580	0.250893	2.94E-05	0.000327	tetrahydromethanopterin S-methyltransferase subunit D EC:2.1.1.86
K03626	0.250873	2.94E-05	0.000327	nascent polypeptide-associated complex subunit alpha
K09733	0.250828	2.95E-05	0.000327	hypothetical protein; hypothetical protein
K11695	0.250828	2.95E-05	0.000327	peptidoglycan pentaglycine glycine transferase (the fourth and fifth glycine) EC:2.3.2.18
K07572	0.250772	2.97E-05	0.000328	putative nucleotide binding protein; putative nucleotide binding protein
K07570	0.251415	0.00003	0.00033	general stress protein 13
K00768	-0.25136	0.00003	0.000331	nicotinate-nucleotide--dimethylbenzimidazole phosphoribosyltransferase EC:2.4.2.21
K05955	0.250569	3.01E-05	0.000331	protein farnesyltransferase/geranylgeranyltransferase type-1 subunit alpha EC:2.5.1.58 2.5.1.59
K03763	0.251202	3.05E-05	0.000333	DNA polymerase III subunit alpha, Gram-positive type EC:2.7.7.7
K05929	0.250433	3.04E-05	0.000333	phosphoethanolamine N-methyltransferase EC:2.1.1.103
K07254	0.250433	3.04E-05	0.000333	tRNA (cytidine56-2'-O)-methyltransferase EC:2.1.1.206
K04797	0.250367	3.06E-05	0.000334	prefoldin alpha subunit
K03167	0.250319	3.07E-05	0.000335	DNA topoisomerase VI subunit B EC:5.99.1.3
K14652	-0.25107	3.08E-05	0.000335	3,4-dihydroxy 2-butanone 4-phosphate synthase / GTP cyclohydrolase II EC:4.1.99.12 3.5.4.25
K01493	0.250903	3.11E-05	0.000338	dCMP deaminase EC:3.5.4.12
K02662	0.250932	0.000031	0.000338	type IV pilus assembly protein PilM
K14095	0.250116	3.12E-05	0.000338	energy-converting hydrogenase A subunit D
K00459	-0.24989	3.17E-05	0.000343	nitronate monooxygenase EC:1.13.12.16
K02988	0.250505	0.000032	0.000346	small subunit ribosomal protein S5
K13282	0.249728	0.000032	0.000346	cyanophycinase EC:3.4.15.6
K03708	0.250456	3.22E-05	0.000347	transcriptional regulator CtsR
K14104	0.249634	3.23E-05	0.000347	energy-converting hydrogenase A subunit M
K04094	0.25037	3.24E-05	0.000348	methylenetetrahydrofolate--tRNA-(uracil-5-)-methyltransferase EC:2.1.1.74
K06223	0.250095	0.000033	0.000354	DNA adenine methylase EC:2.1.1.72
K07077	-0.24917	3.35E-05	0.000358	UPF0755 protein; UPF0755 protein
K14099	0.249132	3.36E-05	0.000359	energy-converting hydrogenase A subunit H
K03686	0.249826	3.37E-05	0.00036	molecular chaperone DnaJ
K05962	-0.24973	0.000034	0.000362	protein-histidine pros-kinase EC:2.7.13.1
K09123	0.24954	3.45E-05	0.000366	hypothetical protein; hypothetical protein
K12874	0.248797	3.44E-05	0.000366	intron-binding protein aquarius

K05949	0.248652	3.48E-05	0.000369	NAD+---dinitrogen-reductase ADP-D-ribosyltransferase EC:2.4.2.37
K03070	0.249389	3.49E-05	0.000369	preprotein translocase subunit SecA
K01599	-0.24929	3.51E-05	0.000371	uroporphyrinogen decarboxylase EC:4.1.1.37
K03622	0.248382	3.55E-05	0.000375	archaea-specific DNA-binding protein
K07333	0.248319	3.57E-05	0.000376	archaeal flagellar protein FlaJ
K05967	0.248989	3.59E-05	0.000378	hypothetical protein; hypothetical protein
K02913	0.248903	3.61E-05	0.00038	large subunit ribosomal protein L33
K03434	0.248008	3.65E-05	0.000382	N-acetylglucosaminylphosphatidylinositol deacetylase EC:3.5.1.89
K01282	0.248005	3.65E-05	0.000382	dipeptidyl aminopeptidase EC:3.4.14.-
K01575	0.248722	3.66E-05	0.000383	acetolactate decarboxylase EC:4.1.1.5
K01553	0.247913	3.68E-05	0.000384	myosin ATPase EC:3.6.4.1
K01580	-0.24848	3.73E-05	0.000389	glutamate decarboxylase EC:4.1.1.15
K02978	0.247689	3.74E-05	0.000389	small subunit ribosomal protein S27e
K01639	-0.24756	3.78E-05	0.000391	N-acetylneuraminate lyase EC:4.1.3.3
K03430	-0.2483	3.78E-05	0.000391	2-aminoethylphosphonate-pyruvate transaminase EC:2.6.1.37
K13993	0.248324	3.77E-05	0.000391	HSP20 family protein
K06939	0.248275	3.78E-05	0.000392	23S rRNA (adenine2503-C2)-methyltransferase EC:2.1.1.192; 23S rRNA (adenine2503-C2)-methyltransferase EC:2.1.1.192
K03432	0.247199	3.88E-05	0.000401	proteasome alpha subunit EC:3.4.25.1
K00981	0.247888	0.000039	0.000402	phosphatidate cytidylyltransferase EC:2.7.7.41
K13613	0.247087	3.91E-05	0.000403	polyketide synthase PksM
K01284	-0.24781	3.92E-05	0.000403	peptidyl-dipeptidase Dcp EC:3.4.15.5
K00358	-0.24771	3.95E-05	0.000405	NADH oxidase EC:1.6.-.-
K03150	-0.2477	3.95E-05	0.000405	thiamine biosynthesis ThiH
K00525	-0.24768	3.96E-05	0.000405	ribonucleoside-diphosphate reductase alpha chain EC:1.17.4.1
K03421	0.246817	3.99E-05	0.000408	methyl-coenzyme M reductase subunit C
K01811	-0.24739	4.04E-05	0.000412	alpha-D-xyloside xylohydrolase EC:3.2.1.177
K14101	0.246387	4.12E-05	0.00042	energy-converting hydrogenase A subunit J
K02653	0.247003	4.16E-05	0.000423	type IV pilus assembly protein PilC
K15118	0.246247	4.16E-05	0.000423	solute carrier family 25, member 38
K09181	-0.24691	4.18E-05	0.000425	hypothetical protein
K14115	0.246102	0.000042	0.000426	energy-converting hydrogenase B subunit F
K01338	0.246428	4.34E-05	0.000439	ATP-dependent Lon protease EC:3.4.21.53
K03136	0.245622	4.36E-05	0.00044	transcription initiation factor TFIIE subunit alpha
K03322	-0.24634	4.36E-05	0.00044	manganese transport protein
K01892	0.246274	4.38E-05	0.000441	histidyl-tRNA synthetase EC:6.1.1.21
K09951	0.246269	4.39E-05	0.000441	CRISPR-associated protein Cas2
K09723	0.245497	0.000044	0.000442	hypothetical protein; hypothetical protein
K03977	0.24606	4.45E-05	0.000447	GTP-binding protein
K03556	0.245901	0.000045	0.000451	LuxR family transcriptional regulator, maltose regulon positive regulatory protein
K03537	0.244875	0.000046	0.00046	ribonuclease P/MRP protein subunit POP5 EC:3.1.26.5
K14118	0.244891	0.000046	0.00046	energy-converting hydrogenase B subunit I
K02470	0.245488	4.64E-05	0.000463	DNA gyrase subunit B EC:5.99.1.3
K13940	0.24547	4.65E-05	0.000463	dihydroneopterin aldolase / 2-amino-4-hydroxy-6-hydroxymethyldihydropteridine diphosphokinase EC:4.1.2.25 2.7.6.3
K03473	-0.24541	4.67E-05	0.000464	erythronate-4-phosphate dehydrogenase EC:1.1.1.290
K03403	0.244606	0.000047	0.000467	magnesium chelatase subunit H EC:6.6.1.1

K11931	0.245234	4.73E-05	0.000469	biofilm PGA synthesis lipoprotein PgaB EC:3.-.-.-
K10112	0.245132	4.77E-05	0.000472	maltose/maltodextrin transport system ATP-binding protein
K03286	-0.24511	4.77E-05	0.000472	OmpA-OmpF porin, OOP family
K07736	0.245087	4.78E-05	0.000472	CarD family transcriptional regulator; CarD family transcriptional regulator
K05306	-0.24503	0.000048	0.000474	phosphonoacetaldehyde hydrolase EC:3.11.1.1
K03536	0.244939	4.83E-05	0.000476	ribonuclease P protein component EC:3.1.26.5
K13678	0.244748	0.000049	0.000482	monoglucosyldiacylglycerol glycosyltransferase EC:2.4.1.-
K06391	0.244677	4.93E-05	0.000484	stage III sporulation protein AB
K01627	-0.24464	4.94E-05	0.000484	2-dehydro-3-deoxyphosphooctonate aldolase (KDO 8-P synthase) EC:2.5.1.55
K11752	-0.24461	4.95E-05	0.000485	diaminohydroxyphosphoribosylaminopyrimidine deaminase / 5-amino-6-(5-phosphoribosylamino)uracil reductase EC:3.5.4.26 1.1.1.193
K01686	-0.2445	0.00005	0.000487	mannonate dehydratase EC:4.2.1.8
K07816	0.244516	4.98E-05	0.000487	putative GTP pyrophosphokinase EC:2.7.6.5
K05813	0.244422	5.02E-05	0.000489	sn-glycerol 3-phosphate transport system substrate-binding protein
K04116	0.243544	5.08E-05	0.000495	cyclohexanecarboxylate-CoA ligase EC:6.2.1.-
K02508	-0.24422	0.000051	0.000495	AraC family transcriptional regulator, 4-hydroxyphenylacetate 3-monooxygenase operon regulatory protein
K07473	0.244189	0.000051	0.000496	DNA-damage-inducible protein J
K01358	0.244071	5.15E-05	0.000499	ATP-dependent Clp protease, protease subunit EC:3.4.21.92
K01153	0.244012	5.17E-05	0.000501	type I restriction enzyme, R subunit EC:3.1.21.3
K01751	-0.24392	0.000052	0.000502	diaminopropionate ammonia-lyase EC:4.3.1.15
K09939	-0.24393	0.000052	0.000502	hypothetical protein; hypothetical protein
K14340	0.24316	5.23E-05	0.000504	mannosyltransferase EC:2.4.1.-; mannosyltransferase EC:2.4.1.-
K02233	-0.24362	5.32E-05	0.000512	adenosylcobinamide-GDP ribazoletransferase EC:2.7.8.26
K05364	0.24352	5.36E-05	0.000515	peptidoglycan glycosyltransferase EC:2.4.1.129
K07586	0.243387	0.000054	0.000519	hypothetical protein; hypothetical protein
K01241	-0.24323	5.47E-05	0.000524	AMP nucleosidase EC:3.2.2.4
K10960	0.242446	0.000055	0.000527	geranylgeranyl reductase EC:1.3.1.83
K01213	-0.24306	5.54E-05	0.00053	galacturan 1,4-alpha-galacturonidase EC:3.2.1.67
K06201	-0.24301	5.56E-05	0.000531	copper homeostasis protein
K07035	0.242799	5.64E-05	0.000538	inner membrane protein; inner membrane protein
K03517	-0.24266	0.000057	0.000542	quinolinate synthase EC:2.5.1.72
K07306	-0.24265	0.000057	0.000542	anaerobic dimethyl sulfoxide reductase subunit A EC:1.8.5.3
K03056	0.241919	5.72E-05	0.000543	DNA-directed RNA polymerase subunit L EC:2.7.7.6
K03045	0.241873	5.74E-05	0.000544	DNA-directed RNA polymerase subunit B" EC:2.7.7.6
K03606	-0.24245	5.79E-05	0.000548	putative colanic acid biosysnthesis UDP-glucose lipid carrier transferase
K02925	0.241687	5.82E-05	0.00055	large subunit ribosomal protein L3e
K01356	0.242351	5.83E-05	0.00055	repressor LexA EC:3.4.21.88
K01870	0.242274	5.86E-05	0.000553	isoleucyl-tRNA synthetase EC:6.1.1.5
K00554	0.242202	0.000059	0.000553	tRNA (guanine37-N1)-methyltransferase EC:2.1.1.228
K01089	-0.24221	5.89E-05	0.000553	imidazoleglycerol-phosphate dehydratase / histidinol-phosphatase EC:4.2.1.19 3.1.3.15
K07478	0.242253	5.87E-05	0.000553	putative ATPase
K12308	-0.2422	0.000059	0.000553	beta-galactosidase EC:3.2.1.23

K01001	0.241464	5.91E-05	0.000554	UDP-N-acetylglucosamine--dolichyl-phosphate N-acetylglucosaminephosphotransferase EC:2.7.8.15
K01750	0.24208	5.94E-05	0.000556	ornithine cyclodeaminase EC:4.3.1.12
K05937	-0.24136	5.96E-05	0.000557	hypothetical protein; hypothetical protein
K12340	-0.24197	5.99E-05	0.000559	outer membrane channel protein
K03106	0.241924	6.01E-05	0.00056	signal recognition particle subunit SRP54
K06976	0.241804	6.06E-05	0.000564	pantoate kinase EC:2.7.1.169; pantoate kinase EC:2.7.1.169
K03120	0.241019	0.000061	0.000568	transcription initiation factor TFIID TATA-box-binding protein
K06194	-0.24154	6.18E-05	0.000572	lipoprotein NlpD
K08096	0.240896	6.16E-05	0.000572	GTP cyclohydrolase IIa EC:3.5.4.29
K13051	-0.24154	6.18E-05	0.000572	beta-aspartyl-peptidase (threonine type) EC:3.4.19.5
K10875	0.241429	6.23E-05	0.000576	DNA repair and recombination protein RAD54 and RAD54-like protein EC:3.6.4.-
K02017	-0.24128	0.000063	0.000581	molybdate transport system ATP-binding protein EC:3.6.3.29
K07117	0.240566	6.31E-05	0.000582	5-hydroxyisourate hydrolase EC:3.5.2.17; 5-hydroxyisourate hydrolase EC:3.5.2.17
K06975	-0.24101	6.42E-05	0.00059	pantoate kinase EC:2.7.1.169; pantoate kinase EC:2.7.1.169
K14194	0.240327	6.42E-05	0.00059	serine-aspartate repeat-containing protein C/D/E
K07585	0.24021	6.48E-05	0.000595	hypothetical protein; hypothetical protein
K08153	-0.24075	6.54E-05	0.000599	MFS transporter, DHA1 family, multidrug resistance protein
K09974	0.240707	6.56E-05	0.000601	hypothetical protein; hypothetical protein
K06168	0.240684	6.57E-05	0.000601	bifunctional enzyme involved in thiolation and methylation of tRNA
K07165	-0.24061	0.000066	0.000603	transmembrane sensor
K03428	0.239873	6.64E-05	0.000605	magnesium-protoporphyrin O-methyltransferase EC:2.1.1.11
K03236	0.239814	6.67E-05	0.000607	translation initiation factor 1A
K01433	-0.24042	0.000067	0.000609	formyltetrahydrofolate deformylase EC:3.5.1.10
K02954	0.240382	6.71E-05	0.00061	small subunit ribosomal protein S14
K09697	0.240347	6.73E-05	0.00061	sodium transport system ATP-binding protein
K03058	0.239687	6.73E-05	0.00061	DNA-directed RNA polymerase subunit N EC:2.7.7.6
K01092	-0.24023	6.79E-05	0.000614	myo-inositol-1(or 4)-monophosphatase EC:3.1.3.25
K07388	0.239531	0.000068	0.000615	hydrogenase expression/formation protein; hydrogenase expression/formation protein
K01730	-0.24007	6.87E-05	0.000619	oligogalacturonide lyase EC:4.2.2.6
K05825	0.240069	6.87E-05	0.000619	2-aminoadipate transaminase EC:2.6.1.-
K02279	0.239993	0.000069	0.000621	pilus assembly protein CpaB
K03270	-0.23996	6.92E-05	0.000621	3-deoxy-D-manno-octulosonate 8-phosphate phosphatase (KDO 8-P phosphatase) EC:3.1.3.45
K07796	-0.23997	6.92E-05	0.000621	Cu(I)/Ag(I) efflux system outer membrane protein CusC
K00948	0.239863	6.97E-05	0.000625	ribose-phosphate pyrophosphokinase EC:2.7.6.1
K10615	0.239803	0.00007	0.000627	E3 ubiquitin-protein ligase HERC4 EC:6.3.2.19
K12373	-0.23974	7.03E-05	0.000629	hexosaminidase EC:3.2.1.52
K07569	0.23865	7.25E-05	0.000647	RNA-binding protein
K13812	0.238581	7.28E-05	0.00065	bifunctional enzyme Fae/Hps EC:4.3.-.- 4.1.2.43
K00966	-0.23922	0.000073	0.00065	mannose-1-phosphate guanylyltransferase EC:2.7.7.13
K15051	0.239188	7.31E-05	0.00065	DNA-entry nuclease; DNA-entry nuclease
K01935	-0.23912	7.34E-05	0.000652	dethiobiotin synthetase EC:6.3.3.3
K02170	0.239117	7.35E-05	0.000652	pimelyl-acyl-carrier protein methyl ester esterase EC:3.1.1.85

K10254	0.239039	7.39E-05	0.000655	myosin-crossreactive antigen
K09809	0.238956	7.43E-05	0.000658	CDP-glycerol glycerophosphotransferase EC:2.7.8.12
K07728	0.238261	7.45E-05	0.000659	putative transcriptional regulator; putative transcriptional regulator
K09775	0.238811	0.000075	0.000663	hypothetical protein; hypothetical protein
K01070	-0.23808	7.55E-05	0.000666	S-formylglutathione hydrolase EC:3.1.2.12
K01209	-0.23854	7.65E-05	0.000673	alpha-N-arabinofuranosidase EC:3.2.1.55
K06949	0.238555	7.65E-05	0.000673	ribosome biogenesis GTPase EC:3.6.1.-
K02074	-0.23852	7.66E-05	0.000673	zinc/manganese transport system ATP-binding protein
K01170	0.237722	7.75E-05	0.000679	tRNA-intron endonuclease, archaea type EC:3.1.27.9
K09789	-0.23835	7.76E-05	0.000679	hypothetical protein; hypothetical protein
K07144	0.237638	0.000078	0.000682	UPF0176 protein; UPF0176 protein
K13963	0.238229	7.83E-05	0.000684	serpin B
K03168	0.238138	7.88E-05	0.000687	DNA topoisomerase I EC:5.99.1.2
K07644	0.238084	0.000079	0.000689	two-component system, OmpR family, heavy metal sensor histidine kinase CusS EC:2.7.13.3
K06296	0.237273	0.00008	0.000696	spore germination protein KB
K02908	0.236848	8.25E-05	0.000717	large subunit ribosomal protein L30e
K03734	-0.23736	8.32E-05	0.000723	thiamine biosynthesis lipoprotein
K07040	0.237297	8.36E-05	0.000725	uncharacterized protein; uncharacterized protein
K07712	-0.23719	8.42E-05	0.00073	two-component system, NtrC family, nitrogen regulation response regulator GlnG
K09003	0.236476	8.47E-05	0.000732	hypothetical protein; hypothetical protein
K03574	0.237058	0.000085	0.000733	7,8-dihydro-8-oxoguanine triphosphatase EC:3.6.1.-
K07717	-0.23707	0.000085	0.000733	two-component system, sensor histidine kinase YcbA EC:2.7.13.3
K09739	0.23645	8.48E-05	0.000733	hypothetical protein; hypothetical protein
K00540	-0.23699	8.54E-05	0.000735	nicotinamide N-methyltransferase EC:2.1.1.1
K08770	0.236337	8.55E-05	0.000735	ubiquitin C
K00806	0.236827	8.64E-05	0.000742	undecaprenyl diphosphate synthase EC:2.5.1.31
K02343	0.236765	8.68E-05	0.000744	DNA polymerase III subunit gamma/tau EC:2.7.7.7
K06284	0.236741	0.000087	0.000745	transcriptional pleiotropic regulator of transition state genes
K06936	0.23589	8.83E-05	0.000755	23S rRNA (adenine2503-C2)-methyltransferase EC:2.1.1.192; 23S rRNA (adenine2503-C2)-methyltransferase EC:2.1.1.192
K01873	0.23639	8.91E-05	0.000761	valyl-tRNA synthetase EC:6.1.1.9
K02922	0.235726	8.93E-05	0.000762	large subunit ribosomal protein L37e
K06989	-0.2363	8.97E-05	0.000764	aspartate dehydrogenase EC:1.4.1.21
K02794	-0.23623	0.00009	0.000765	PTS system, mannose-specific IIB component EC:2.7.1.69
K07732	0.23561	0.00009	0.000765	riboflavin kinase, archaea type EC:2.7.1.161
K01456	0.235625	0.00009	0.000765	peptide-N4-(N-acetyl-beta-glucosaminyl)asparagine amidase EC:3.5.1.52
K00979	-0.23614	9.07E-05	0.000769	3-deoxy-manno-octulosonate cytidylyltransferase (CMP-KDO synthetase) EC:2.7.7.38
K03552	0.235159	0.000093	0.000787	holliday junction resolvase, archaea type
K08591	0.235752	9.32E-05	0.000787	glycerol-3-phosphate acyltransferase PlsY EC:2.3.1.15
K13643	0.235762	9.31E-05	0.000787	Rrf2 family transcriptional regulator, iron-sulfur cluster assembly transcription factor
K07321	0.235688	9.36E-05	0.00079	CO dehydrogenase maturation factor
K07560	0.235571	9.44E-05	0.000795	D-tyrosyl-tRNA(Tyr) deacylase EC:3.1.-.-
K04800	0.234903	9.47E-05	0.000797	replication factor C large subunit
K02886	0.235505	9.48E-05	0.000797	large subunit ribosomal protein L2
K00925	0.23542	9.54E-05	0.000798	acetate kinase EC:2.7.2.1

K01278	-0.23541	9.54E-05	0.000798	dipeptidyl-peptidase 4 EC:3.4.14.5
K03050	0.23479	9.54E-05	0.000798	DNA-directed RNA polymerase subunit E" EC:2.7.7.6
K09777	0.235456	9.51E-05	0.000798	hypothetical protein; hypothetical protein
K00583	0.234588	9.68E-05	0.000804	tetrahydromethanopterin S-methyltransferase subunit G EC:2.1.1.86
K02068	-0.2352	9.69E-05	0.000804	putative ABC transport system ATP-binding protein
K02863	0.235234	9.66E-05	0.000804	large subunit ribosomal protein L1
K09142	0.234584	9.68E-05	0.000804	hypothetical protein; hypothetical protein
K09154	0.23466	9.63E-05	0.000804	hypothetical protein; hypothetical protein
K11440	0.234578	9.69E-05	0.000804	choline dehydrogenase EC:1.1.1.1
K12600	-0.23519	0.000097	0.000804	superkiller protein 3
K09128	0.235031	0.000098	0.000811	hypothetical protein; hypothetical protein
K09968	0.235047	9.79E-05	0.000811	hypothetical protein; hypothetical protein
K08070	0.234805	0.0001	0.000823	2-alkenal reductase EC:1.3.1.74
K07580	0.234123	0.0001	0.000826	hypothetical protein; hypothetical protein
K01815	-0.23469	0.0001	0.000828	4-deoxy-L-threo-5-hexosulose-uronate ketol-isomerase EC:5.3.1.17
K04484	0.233902	0.000102	0.000836	DNA repair protein RadB
K14124	0.233901	0.000102	0.000836	energy-converting hydrogenase B subunit O
K00443	0.233829	0.000102	0.000838	coenzyme F420 hydrogenase gamma subunit EC:1.12.98.1
K01043	-0.23444	0.000102	0.000838	carboxylesterase 1 EC:3.1.1.1
K03561	-0.23443	0.000102	0.000838	biopolymer transport protein ExbB
K03090	0.234331	0.000103	0.000843	RNA polymerase sigma-B factor
K11442	0.233632	0.000104	0.000847	putative uridylyltransferase EC:2.7.7.-; putative uridylyltransferase EC:2.7.7.-
K12950	0.233521	0.000104	0.000852	cation-transporting P-type ATPase C EC:3.6.3.-
K09800	-0.23402	0.000105	0.000858	hypothetical protein; hypothetical protein
K00581	0.2333	0.000106	0.000864	tetrahydromethanopterin S-methyltransferase subunit E EC:2.1.1.86
K04042	0.233842	0.000106	0.000866	bifunctional UDP-N-acetylglucosamine pyrophosphorylase / Glucosamine-1-phosphate N-acetyltransferase EC:2.7.7.23 2.3.1.157
K07576	0.233838	0.000107	0.000866	metallo-beta-lactamase family protein; metallo-beta-lactamase family protein
K11294	-0.23367	0.000108	0.000875	nucleolin
K11927	-0.23305	0.000108	0.000875	ATP-dependent RNA helicase RhlE EC:3.6.4.13
K01436	-0.23361	0.000108	0.000876	amidohydrolase EC:3.5.1.-; amidohydrolase EC:3.5.1.-
K05985	0.233608	0.000108	0.000876	ribonuclease M5 EC:3.1.26.8
K01256	-0.23358	0.000108	0.000876	aminopeptidase N EC:3.4.11.2
K05595	-0.23354	0.000109	0.000878	multiple antibiotic resistance protein
K02003	0.233427	0.00011	0.000884	putative ABC transport system ATP-binding protein
K01188	-0.23338	0.00011	0.000886	beta-glucosidase EC:3.2.1.21
K01746	-0.23333	0.00011	0.000887	formiminotetrahydrofolate cyclodeaminase EC:4.3.1.4
K07726	0.233344	0.00011	0.000887	putative transcriptional regulator; putative transcriptional regulator
K00106	0.232673	0.000111	0.000889	xanthine dehydrogenase/oxidase EC:1.17.1.4 1.17.3.2
K06894	-0.23323	0.000111	0.000891	L-lysine exporter family protein LysE/ArgO; L-lysine exporter family protein LysE/ArgO
K12511	0.233212	0.000111	0.000891	tight adherence protein C
K03558	-0.23303	0.000113	0.0009	membrane protein required for colicin V production
K02875	0.232428	0.000113	0.0009	large subunit ribosomal protein L14e
K03383	-0.23238	0.000113	0.000902	cyanuric acid amidohydrolase EC:3.5.2.15
K00440	0.232249	0.000114	0.000907	coenzyme F420 hydrogenase alpha subunit EC:1.12.98.1

K01547	-0.23284	0.000114	0.000907	K+-transporting ATPase ATPase B chain EC:3.6.3.12
K05305	-0.23288	0.000114	0.000907	fucokinase EC:2.7.1.52
K12510	0.232835	0.000114	0.000907	tight adherence protein B
K03444	-0.23277	0.000115	0.000909	MFS transporter, SP family, sugar porter, other
K08676	-0.23278	0.000115	0.000909	tricorn protease EC:3.4.21.-
K02319	0.232143	0.000115	0.00091	DNA polymerase I EC:2.7.7.7
K02600	0.232677	0.000115	0.000912	N utilization substance protein A
K07258	0.232687	0.000115	0.000912	D-alanyl-D-alanine carboxypeptidase (penicillin-binding protein 5/6) EC:3.4.16.4
K07557	0.2319	0.000117	0.000922	archaeosine tRNA-ribosyltransferase EC:2.4.2.-; archaeosine tRNA-ribosyltransferase EC:2.4.2.-
K03409	0.232286	0.000119	0.000934	chemotaxis protein CheX
K06411	0.232302	0.000118	0.000934	dipicolinate synthase subunit B
K13612	0.231618	0.000119	0.000937	polyketide synthase PksL
K08303	0.232154	0.00012	0.00094	putative protease EC:3.4.-.-; putative protease EC:3.4.-.-
K10110	0.232108	0.00012	0.000941	maltose/maltodextrin transport system permease protein
K11610	0.231511	0.00012	0.000941	beta-ketoacyl ACP reductase EC:1.1.1.100
K00551	0.232064	0.00012	0.000942	phosphatidylethanolamine N-methyltransferase EC:2.1.1.17
K01623	-0.23208	0.00012	0.000942	fructose-bisphosphate aldolase, class I EC:4.1.2.13
K02231	-0.2319	0.000122	0.000951	adenosylcobinamide kinase / adenosylcobinamide-phosphate guanylyltransferase EC:2.7.1.156 2.7.7.62
K06413	0.231637	0.000124	0.000968	stage V sporulation protein K
K01990	0.231545	0.000125	0.000972	ABC-2 type transport system ATP-binding protein
K04085	-0.23154	0.000125	0.000972	tRNA 2-thiouridine synthesizing protein A EC:2.8.1.-
K00442	0.230922	0.000125	0.000973	coenzyme F420 hydrogenase delta subunit
K00362	0.231344	0.000127	0.000983	nitrite reductase (NAD(P)H) large subunit EC:1.7.1.4
K00845	-0.23124	0.000127	0.000989	glucokinase EC:2.7.1.2
K14761	0.231144	0.000128	0.000994	ribosome-associated protein
K13491	0.230508	0.000129	0.000997	two-component system, chemotaxis family, response regulator WspF EC:3.1.1.61
K09710	0.231047	0.000129	0.000999	ribosome-associated protein
K03540	0.23043	0.000129	0.001	ribonuclease P protein subunit RPR2 EC:3.1.26.5
K02796	-0.23038	0.00013	0.001002	PTS system, mannose-specific IID component
K00803	0.230861	0.000131	0.001008	alkyldihydroxyacetonephosphate synthase EC:2.5.1.26
K02548	-0.23085	0.000131	0.001008	1,4-dihydroxy-2-naphthoate octaprenyltransferase EC:2.5.1.74 2.5.1.-
K03394	-0.23062	0.000133	0.001022	precorrin-2/cobalt-factor-2 C20-methyltransferase EC:2.1.1.130 2.1.1.151
K07679	-0.23055	0.000134	0.001026	two-component system, NarL family, sensor histidine kinase EvgS EC:2.7.13.3
K03969	0.23053	0.000134	0.001027	phage shock protein A
K02323	0.229849	0.000135	0.001033	DNA polymerase II small subunit EC:2.7.7.7
K03650	0.230389	0.000135	0.001034	tRNA modification GTPase EC:3.6.-.-
K04047	-0.23036	0.000135	0.001034	starvation-inducible DNA-binding protein
K07991	0.229768	0.000136	0.001034	archaeal preflagellin peptidase FlaK EC:3.4.23.52
K09735	0.229798	0.000135	0.001034	hypothetical protein; hypothetical protein
K02860	0.230314	0.000136	0.001035	16S rRNA processing protein RimM
K00975	0.230027	0.000139	0.001054	glucose-1-phosphate adenylyltransferase EC:2.7.7.27
K06020	-0.22984	0.00014	0.001065	sulfate-transporting ATPase EC:3.6.3.25
K14170	0.229866	0.00014	0.001065	chorismate mutase / prephenate dehydratase EC:5.4.99.5 4.2.1.51
K03059	0.229281	0.00014	0.001065	DNA-directed RNA polymerase subunit P EC:2.7.7.6
K02964	0.229214	0.000141	0.001067	small subunit ribosomal protein S18e

K06023	0.229757	0.000141	0.001068	HPr kinase/phosphorylase EC:2.7.11.- 2.7.4.-
K02536	-0.2291	0.000142	0.001073	UDP-3-O-3-hydroxymyristoyl glucosamine N-acyltransferase EC:2.3.1.191
K09595	0.228936	0.000144	0.001084	minor histocompatibility antigen H13 EC:3.4.23.-
K08097	0.22891	0.000144	0.001085	phosphosulfolactate synthase EC:4.4.1.19
K02491	0.22888	0.000144	0.001086	two-component system, sporulation sensor kinase A EC:2.7.13.3
K06400	0.229436	0.000144	0.001086	site-specific DNA recombinase
K09811	0.229406	0.000145	0.001087	cell division transport system permease protein
K07082	0.229241	0.000146	0.001095	UPF0755 protein; UPF0755 protein
K12234	0.228689	0.000146	0.001095	coenzyme F420-0_L-glutamate ligase / coenzyme F420-1_gamma-L-glutamate ligase EC:6.3.2.31 6.3.2.34
K12428	0.228683	0.000146	0.001095	fatty acid CoA ligase FadD32
K13942	0.228693	0.000146	0.001095	5,10-methenyltetrahydromethanopterin hydrogenase EC:1.12.98.2
K14096	0.228544	0.000147	0.001103	energy-converting hydrogenase A subunit E
K01401	0.228506	0.000148	0.001105	aureolysin EC:3.4.24.29
K01046	0.229055	0.000148	0.001105	triacylglycerol lipase EC:3.1.1.3
K12823	0.228458	0.000148	0.001106	ATP-dependent RNA helicase DDX5/DBP2 EC:3.6.4.13
K14102	0.22834	0.00015	0.001114	energy-converting hydrogenase A subunit K
K07106	-0.22885	0.00015	0.001116	N-acetylmuramic acid 6-phosphate etherase EC:4.2.1.126
K07158	0.228128	0.000152	0.001128	UPF0271 protein; UPF0271 protein
K06992	0.227808	0.000155	0.001151	putative drug exporter of the RND superfamily; putative drug exporter of the RND superfamily
K01006	0.228343	0.000155	0.001152	pyruvate,orthophosphate dikinase EC:2.7.9.1
K01424	-0.22822	0.000157	0.001161	L-asparaginase EC:3.5.1.1
K06982	0.227542	0.000158	0.001169	pantoate kinase EC:2.7.1.169
K09687	0.228005	0.000159	0.001175	antibiotic transport system ATP-binding protein
K01972	0.227979	0.000159	0.001176	DNA ligase (NAD+) EC:6.5.1.2
K00425	-0.22772	0.000162	0.001196	cytochrome d ubiquinol oxidase subunit I EC:1.10.3.-
K06237	0.227057	0.000163	0.001203	collagen, type IV, alpha
K00842	0.227587	0.000164	0.001204	aminotransferase EC:2.6.1.-
K01180	0.227564	0.000164	0.001204	endo-1,3(4)-beta-glucanase EC:3.2.1.6
K01874	0.227036	0.00017	0.001247	methionyl-tRNA synthetase EC:6.1.1.10
K03049	0.226426	0.00017	0.001251	DNA-directed RNA polymerase subunit E' EC:2.7.7.6
K12394	0.226122	0.000174	0.001275	AP-1 complex subunit sigma 1/2
K00096	0.226463	0.000176	0.001292	glycerol-1-phosphate dehydrogenase NAD(P) EC:1.1.1.261
K06875	0.225843	0.000177	0.001297	programmed cell death protein 5
K07162	0.226366	0.000178	0.001298	transmembrane sensor; transmembrane sensor
K01546	-0.22633	0.000178	0.001299	K+-transporting ATPase ATPase A chain EC:3.6.3.12
K02688	-0.22615	0.00018	0.001311	transcriptional regulator, propionate catabolism operon regulatory protein
K03760	-0.22616	0.00018	0.001311	phosphoethanolamine transferase
K09764	-0.22616	0.00018	0.001311	hypothetical protein; hypothetical protein
K06298	0.226104	0.000181	0.001314	germination protein M
K00894	0.225329	0.000184	0.001332	ethanolamine kinase EC:2.7.1.82
K14110	0.225343	0.000184	0.001332	energy-converting hydrogenase B subunit A
K01846	0.225782	0.000185	0.001338	methylaspartate mutase EC:5.4.99.1
K01966	-0.22573	0.000185	0.001341	propionyl-CoA carboxylase beta chain EC:6.4.1.3
K05716	0.225121	0.000186	0.001347	cyclic 2,3-diphosphoglycerate synthetase EC:4.6.1.-
K00426	-0.22562	0.000187	0.001349	cytochrome d ubiquinol oxidase subunit II EC:1.10.3.-
K01885	0.225532	0.000188	0.001355	glutamyl-tRNA synthetase EC:6.1.1.17

K01109	0.224764	0.000191	0.001375	inositol polyphosphate-4-phosphatase EC:3.1.3.66
K03892	0.225235	0.000192	0.00138	ArsR family transcriptional regulator
K03048	0.225193	0.000192	0.001382	DNA-directed RNA polymerase subunit delta
K07739	0.224638	0.000192	0.001383	elongator complex protein 3 EC:2.3.1.48
K03487	0.224616	0.000193	0.001383	LacI family transcriptional regulator, asc operon repressor
K00141	0.224565	0.000193	0.001387	benzaldehyde dehydrogenase (NAD) EC:1.2.1.28
K00278	-0.22507	0.000194	0.001388	L-aspartate oxidase EC:1.4.3.16
K11632	-0.22449	0.000194	0.001391	bacitracin transport system permease protein
K06173	0.224977	0.000195	0.001394	tRNA pseudouridine38-40 synthase EC:5.4.99.12
K00634	-0.22492	0.000196	0.001395	phosphate butyryltransferase EC:2.3.1.19
K01787	-0.22491	0.000196	0.001395	N-acylglucosamine 2-epimerase EC:5.1.3.8
K09724	0.224394	0.000196	0.001395	hypothetical protein; hypothetical protein
K07123	0.224172	0.000199	0.001412	5-hydroxyisourate hydrolase EC:3.5.2.17; 5-hydroxyisourate hydrolase EC:3.5.2.17
K09790	-0.22471	0.000199	0.001412	hypothetical protein; hypothetical protein
K05827	0.224078	0.0002	0.00142	alpha-aminoadipate--LysW ligase LysX EC:6.3.2.-
K01828	-0.22445	0.000202	0.001432	protein disulfide-isomerase EC:5.3.4.1
K10606	0.223921	0.000202	0.001432	E3 ubiquitin-protein ligase FANCL EC:6.3.2.19
K04835	0.223697	0.000205	0.001451	methylaspartate ammonia-lyase EC:4.3.1.2
K06199	-0.22422	0.000205	0.001451	CrcB protein
K04563	0.22363	0.000206	0.001456	cyclin-dependent kinase EC:2.7.11.22
K00201	0.223543	0.000207	0.001463	formylmethanofuran dehydrogenase subunit B EC:1.2.99.5
K01598	0.223463	0.000208	0.001469	phosphopantothenoylcysteine decarboxylase EC:4.1.1.36
K03422	0.223379	0.00021	0.001476	methyl-coenzyme M reductase subunit D
K02684	0.22332	0.00021	0.00148	DNA primase small subunit EC:2.7.7.-
K01745	-0.22382	0.000211	0.001481	histidine ammonia-lyase EC:4.3.1.3
K03569	0.223801	0.000211	0.001481	rod shape-determining protein MreB and related proteins
K00298	0.223251	0.000211	0.001483	N5-(carboxyethyl)ornithine synthase EC:1.5.1.24
K03149	-0.22367	0.000213	0.001491	thiamine biosynthesis ThiG
K02188	-0.22363	0.000213	0.001494	cobalt-precorrin-5B (C1)-methyltransferase EC:2.1.1.195
K00333	0.22302	0.000215	0.0015	NADH-quinone oxidoreductase subunit D EC:1.6.5.3
K06187	0.223555	0.000214	0.0015	recombination protein RecR
K02341	0.223516	0.000215	0.001501	DNA polymerase III subunit delta' EC:2.7.7.7
K10212	0.222738	0.000219	0.001525	glycosyl-4,4'-diaponeurosporenoate acyltransferase EC:2.3.1.-; glycosyl-4,4'-diaponeurosporenoate acyltransferase EC:2.3.1.-
K04801	0.222296	0.000225	0.00157	replication factor C small subunit
K00860	-0.22269	0.000227	0.00158	adenylylsulfate kinase EC:2.7.1.25
K07250	0.222655	0.000228	0.001581	4-aminobutyrate aminotransferase / (S)-3-amino-2-methylpropionate transaminase EC:2.6.1.19 2.6.1.22
K09810	-0.22266	0.000228	0.001581	lipoprotein-releasing system ATP-binding protein EC:3.6.3.-
K01271	-0.22262	0.000228	0.001583	Xaa-Pro dipeptidase EC:3.4.13.9
K13985	-0.22254	0.000229	0.001589	N-acyl-phosphatidylethanolamine-hydrolyzing phospholipase D EC:3.1.4.54
K06914	0.221948	0.000231	0.001597	tRNA 2-selenouridine synthase EC:2.9.1.-; tRNA 2-selenouridine synthase EC:2.9.1.-
K06911	-0.22244	0.000231	0.001597	alpha-ketoglutarate-dependent 2,4-dichlorophenoxyacetate dioxygenase EC:1.14.11.-; alpha-ketoglutarate-dependent 2,4-dichlorophenoxyacetate dioxygenase EC:1.14.11.-
K02795	-0.22233	0.000233	0.001607	PTS system, mannose-specific IIC component
K02835	0.222263	0.000234	0.001613	peptide chain release factor 1
K03827	-0.22218	0.000235	0.00162	putative acetyltransferase EC:2.3.1.-

K02624	0.222122	0.000236	0.001621	IclR family transcriptional regulator, pca regulon regulatory protein
K06382	0.222149	0.000235	0.001621	stage II sporulation protein E EC:3.1.3.16
K05542	0.221628	0.000236	0.001621	tRNA-dihydrouridine synthase 1 EC:1.3.1.88
K06410	0.22201	0.000238	0.00163	dipicolinate synthase subunit A
K08744	0.221511	0.000237	0.00163	cardiolipin synthase EC:2.7.8.-
K09738	0.221417	0.000239	0.001638	hypothetical protein; hypothetical protein
K03548	0.221902	0.000239	0.001639	putative permease
K02059	-0.22124	0.000242	0.001654	putative sulfate transport system ATP-binding protein
K00813	0.221183	0.000243	0.001657	aspartate aminotransferase EC:2.6.1.1
K06913	0.221198	0.000242	0.001657	tRNA 2-selenouridine synthase EC:2.9.1.-; tRNA 2-selenouridine synthase EC:2.9.1.-
K03621	0.221455	0.000246	0.001679	glycerol-3-phosphate acyltransferase PlsX EC:2.3.1.15
K07259	-0.22145	0.000247	0.001679	D-alanyl-D-alanine carboxypeptidase / D-alanyl-D-alanine-endopeptidase (penicillin-binding protein 4) EC:3.4.16.4 3.4.21.-
K14059	0.221443	0.000247	0.001679	integrase
K11719	-0.22133	0.000249	0.00169	lipopolysaccharide export system protein LptC
K02674	0.221272	0.000249	0.001693	type IV pilus assembly protein PilY1
K07223	-0.22129	0.000249	0.001693	putative iron-dependent peroxidase; putative iron-dependent peroxidase
K06979	0.221179	0.000251	0.001702	pantoate kinase EC:2.7.1.169; pantoate kinase EC:2.7.1.169
K03703	0.220996	0.000254	0.001721	excinuclease ABC subunit C
K11537	-0.22093	0.000255	0.001726	MFS transporter, NHS family, xanthosine permease
K03285	-0.2209	0.000256	0.001728	general bacterial porin, GBP family
K03596	0.220784	0.000258	0.00174	GTP-binding protein LepA
K06631	0.220252	0.000258	0.001742	polo-like kinase 1 EC:2.7.11.21
K09011	-0.22069	0.000259	0.001747	D-citramalate synthase EC:2.3.1.182
K03975	-0.22066	0.00026	0.001749	membrane-associated protein
K00275	-0.22054	0.000262	0.001762	pyridoxamine 5'-phosphate oxidase EC:1.4.3.5
K02817	-0.22035	0.000265	0.00178	PTS system, trehalose-specific IIA component EC:2.7.1.69
K13652	-0.22035	0.000265	0.00178	AraC family transcriptional regulator; AraC family transcriptional regulator
K07744	0.219826	0.000266	0.001781	hypothetical protein; hypothetical protein
K08298	0.220293	0.000266	0.001783	crotonobetainyl-CoA_carnitine CoA-transferase EC:2.8.3.-
K15255	-0.22024	0.000267	0.001787	ATP-dependent DNA helicase PIF1 EC:3.6.4.12
K06374	0.220208	0.000268	0.001789	spore maturation protein B
K14122	0.219674	0.000268	0.001793	energy-converting hydrogenase B subunit M
K02547	0.220022	0.000271	0.001808	methicillin resistance protein
K00510	0.219392	0.000273	0.001821	heme oxygenase EC:1.14.99.3
K09741	0.219399	0.000273	0.001821	hypothetical protein; hypothetical protein
K05905	-0.2198	0.000275	0.001829	protein-disulfide reductase EC:1.8.1.8
K14098	0.219267	0.000276	0.001832	energy-converting hydrogenase A subunit G
K02970	0.219549	0.000279	0.001854	small subunit ribosomal protein S21
K04373	0.219059	0.000279	0.001854	p90 ribosomal S6 kinase EC:2.7.11.1
K06442	0.219438	0.000281	0.001866	23S rRNA (cytidine1920-2'-O)/16S rRNA (cytidine1409-2'-O)-methyltransferase EC:2.1.1.226 2.1.1.227
K01507	0.219395	0.000282	0.001869	inorganic pyrophosphatase EC:3.6.1.1
K01516	0.219364	0.000283	0.001871	
K14838	0.21886	0.000283	0.001871	nucleolar protein 15
K04075	0.219294	0.000284	0.001874	tRNA(Ile)-lysidine synthase EC:6.3.4.19
K12164	0.218821	0.000284	0.001874	ubiquitin-like modifier-activating enzyme 5
K01866	0.219092	0.000288	0.001897	tyrosyl-tRNA synthetase EC:6.1.1.1

K02915	0.218517	0.000289	0.001907	large subunit ribosomal protein L34e
K14807	0.218455	0.000291	0.001913	ATP-dependent RNA helicase DDX51/DBP6 EC:3.6.4.13
K00156	-0.21886	0.000292	0.00192	pyruvate dehydrogenase (quinone) EC:1.2.5.1
K00074	0.218764	0.000294	0.001927	3-hydroxybutyryl-CoA dehydrogenase EC:1.1.1.157
K06034	0.218304	0.000294	0.001927	sulfopyruvate decarboxylase subunit alpha EC:4.1.1.79; sulfopyruvate decarboxylase subunit alpha EC:4.1.1.79
K09727	0.218294	0.000294	0.001927	hypothetical protein; hypothetical protein
K01423	-0.21869	0.000295	0.001933	L-asparaginase EC:3.5.1.1
K01548	-0.2187	0.000295	0.001933	K+-transporting ATPase ATPase C chain EC:3.6.3.12
K02065	-0.21868	0.000296	0.001933	putative ABC transport system ATP-binding protein
K00652	-0.21858	0.000298	0.001943	8-amino-7-oxononanoate synthase EC:2.3.1.47
K14117	0.21806	0.000298	0.001946	energy-converting hydrogenase B subunit H
K13486	0.218026	0.000299	0.001949	chemotaxis protein methyltransferase WspC
K13002	-0.21834	0.000302	0.001969	glycosyltransferase EC:2.4.1.-
K06984	0.217827	0.000303	0.001971	aspartyl protease family protein; aspartyl protease family protein
K14125	0.217768	0.000304	0.001977	energy-converting hydrogenase B subunit P
K12668	0.217658	0.000306	0.001989	oligosaccharyltransferase complex subunit epsilon
K10627	0.217516	0.000309	0.002006	E3 ubiquitin-protein ligase RAD18 EC:6.3.2.19
K07498	0.217836	0.000312	0.002024	putative transposase; putative transposase
K01869	0.217815	0.000313	0.002025	leucyl-tRNA synthetase EC:6.1.1.4
K13277	0.217771	0.000314	0.002029	minor extracellular protease Epr EC:3.4.21.-
K00607	-0.21766	0.000316	0.002042	aspartate carbamoyltransferase EC:2.1.3.2
K01054	0.217062	0.000318	0.002057	acylglycerol lipase EC:3.1.1.23
K11392	0.217404	0.000321	0.002072	16S rRNA (cytosine1407-C5)-methyltransferase EC:2.1.1.178
K03805	-0.21691	0.000322	0.002073	thiol_disulfide interchange protein DsbG
K03495	0.216871	0.000322	0.002076	tRNA uridine 5-carboxymethylaminomethyl modification enzyme
K01685	-0.21732	0.000323	0.002077	altronate hydrolase EC:4.2.1.7
K08717	-0.21716	0.000326	0.002095	urea transporter
K13925	0.217171	0.000326	0.002095	plasmin and fibronectin-binding protein A
K10773	0.217059	0.000328	0.002107	endonuclease III EC:4.2.99.18
K00259	-0.2169	0.000332	0.002127	alanine dehydrogenase EC:1.4.1.1
K07456	0.216837	0.000333	0.002134	DNA mismatch repair protein MutS2
K12508	0.216356	0.000333	0.002134	feruloyl-CoA synthase EC:6.2.1.34
K08314	0.216299	0.000335	0.00214	fructose-6-phosphate aldolase 2 EC:4.1.2.-
K02056	0.216672	0.000337	0.00215	simple sugar transport system ATP-binding protein EC:3.6.3.17
K00767	-0.21657	0.000339	0.002162	nicotinate-nucleotide pyrophosphorylase (carboxylating) EC:2.4.2.19
K08068	0.21608	0.00034	0.002164	N-acetylglucosamine-6-phosphate 2-epimerase and phosphatase EC:5.1.3.-
K02563	0.216438	0.000342	0.002177	UDP-N-acetylglucosamine--N-acetylmuramyl-(pentapeptide) pyrophosphoryl-undecaprenol N-acetylglucosamine transferase EC:2.4.1.227
K03539	0.215923	0.000343	0.002181	ribonuclease P/MRP protein subunit RPP1 EC:3.1.26.5
K07443	-0.21638	0.000343	0.002181	methylated-DNA-protein-cysteine methyltransferase related protein
K06211	0.216315	0.000345	0.002188	HipB family transcriptional regulator, involved in the regulation of NAD biosynthesis
K11934	-0.21631	0.000345	0.002188	outer membrane protein X
K01002	-0.21625	0.000346	0.002194	phosphoglycerol transferase EC:2.7.8.20
K09924	-0.21577	0.000346	0.002194	hypothetical protein; hypothetical protein

K02993	0.215743	0.000347	0.002196	small subunit ribosomal protein S7e
K07226	0.215727	0.000347	0.002196	hypothetical protein; hypothetical protein
K13572	0.216138	0.000349	0.002201	proteasome accessory factor B
K00937	-0.21601	0.000351	0.002217	polyphosphate kinase EC:2.7.4.1
K01133	-0.21599	0.000352	0.002218	choline-sulfatase EC:3.1.6.6
K13049	0.215978	0.000352	0.002218	carboxypeptidase PM20D1 EC:3.4.17.-
K14446	0.215467	0.000353	0.002223	crotonyl-CoA carboxylase/reductase EC:1.3.1.85
K12960	0.215907	0.000354	0.002224	5-methylthioadenosine/S-adenosylhomocysteine deaminase EC:3.5.4.31 3.5.4.28
K02471	-0.21585	0.000355	0.00223	putative ATP-binding cassette transporter
K08794	0.215382	0.000355	0.00223	calcium/calmodulin-dependent protein kinase I EC:2.7.11.17
K00658	-0.21529	0.000357	0.002241	2-oxoglutarate dehydrogenase E2 component (dihydrolipoamide succinyltransferase) EC:2.3.1.61
K02538	-0.21549	0.000363	0.002276	activator of the mannose operon, transcriptional antiterminator
K03771	-0.21545	0.000364	0.002279	peptidyl-prolyl cis-trans isomerase SurA EC:5.2.1.8
K08173	-0.21545	0.000364	0.002279	MFS transporter, MHS family, metabolite_H+ symporter
K14092	0.214962	0.000365	0.002281	energy-converting hydrogenase A subunit A
K01114	-0.21538	0.000366	0.002284	phospholipase C EC:3.1.4.3
K00627	0.215325	0.000367	0.00229	pyruvate dehydrogenase E2 component (dihydrolipoamide acetyltransferase) EC:2.3.1.12
K02894	0.214839	0.000368	0.002293	large subunit ribosomal protein L23e
K03086	0.215153	0.000371	0.002312	RNA polymerase primary sigma factor
K02049	0.215136	0.000372	0.002312	NitT/TauT family transport system ATP-binding protein
K06959	0.215107	0.000372	0.002314	uncharacterized protein; uncharacterized protein
K11065	-0.21505	0.000374	0.00232	thiol peroxidase, atypical 2-Cys peroxiredoxin EC:1.11.1.15
K03698	0.214919	0.000377	0.002338	CMP-binding protein
K00032	0.214418	0.000378	0.002343	phosphogluconate 2-dehydrogenase EC:1.1.1.43
K01141	-0.21472	0.000382	0.002364	exodeoxyribonuclease I EC:3.1.11.1
K06419	0.214243	0.000382	0.002366	small acid-soluble spore protein B (major beta-type SASP)
K02429	-0.21464	0.000384	0.002372	MFS transporter, FHS family, L-fucose permease
K03300	-0.21454	0.000386	0.002384	citrate-Mg2+_H+ or citrate-Ca2+_H+ symporter, CitMHS family
K10918	-0.2145	0.000387	0.002389	LysR family transcriptional regulator, transcriptional activator AphB
K00240	-0.21448	0.000388	0.00239	succinate dehydrogenase iron-sulfur subunit EC:1.3.99.1
K00677	-0.21418	0.000395	0.002434	UDP-N-acetylglucosamine acyltransferase EC:2.3.1.129
K07107	-0.21407	0.000398	0.002447	acyl-CoA thioester hydrolase EC:3.1.2.-
K13201	0.213623	0.000398	0.002447	nucleolysin TIA-1/TIAR
K01843	-0.21399	0.0004	0.002456	lysine 2,3-aminomutase EC:5.4.3.2
K02869	0.213384	0.000404	0.00248	large subunit ribosomal protein L12
K01804	-0.21372	0.000407	0.002496	L-arabinose isomerase EC:5.3.1.4
K01392	0.213139	0.000411	0.002515	thimet oligopeptidase EC:3.4.24.15
K07198	0.212964	0.000415	0.002541	5'-AMP-activated protein kinase, catalytic alpha subunit EC:2.7.11.11
K00202	0.213372	0.000416	0.002544	formylmethanofuran dehydrogenase subunit C EC:1.2.99.5
K01269	0.213349	0.000417	0.002546	aminopeptidase EC:3.4.11.-
K04031	-0.21331	0.000418	0.002549	ethanolamine utilization protein EutS
K01932	0.213268	0.000419	0.002554	phosphoribosylformylglycinamidine cyclo-ligase EC:6.3.3.1
K00281	-0.21325	0.000419	0.002555	glycine dehydrogenase EC:1.4.4.2

K14116	0.212476	0.000428	0.002608	energy-converting hydrogenase B subunit G
K03077	-0.21286	0.00043	0.002614	L-ribulose-5-phosphate 4-epimerase EC:5.1.3.4
K02015	-0.21282	0.000431	0.002619	iron complex transport system permease protein
K02067	-0.21274	0.000433	0.00263	putative ABC transport system substrate-binding protein
K01138	-0.21261	0.000437	0.00265	uncharacterized sulfatase EC:3.1.6.-; uncharacterized sulfatase EC:3.1.6.-
K07738	0.212557	0.000438	0.002656	transcriptional repressor NrdR
K09482	0.211986	0.000442	0.002677	glutamyl-tRNA(Gln) amidotransferase subunit D EC:6.3.5.7
K03055	0.211952	0.000443	0.002678	DNA-directed RNA polymerase subunit K EC:2.7.7.6
K07512	0.21196	0.000443	0.002678	mitochondrial trans-2-enoyl-CoA reductase EC:1.3.1.38
K09141	0.211801	0.000447	0.002699	hypothetical protein; hypothetical protein
K01722	0.211813	0.000447	0.002699	hydroperoxide dehydratase EC:4.2.1.92
K02169	-0.21219	0.000449	0.002704	malonyl-CoA O-methyltransferase EC:2.1.1.197
K08991	0.211545	0.000455	0.002739	crossover junction endonuclease MUS81 EC:3.1.22.-
K07652	0.211931	0.000456	0.002744	two-component system, OmpR family, sensor histidine kinase VicK EC:2.7.13.3
K02909	0.211909	0.000457	0.002746	large subunit ribosomal protein L31
K10896	0.21143	0.000458	0.002752	fanconi anemia group M protein
K03801	-0.21141	0.000459	0.002752	lipoyl(octanoyl) transferase EC:2.3.1.181
K05823	0.211271	0.000463	0.002775	N-acetyldiaminopimelate deacetylase EC:3.5.1.47
K04773	-0.21167	0.000463	0.002777	protease IV EC:3.4.21.-
K06017	0.211534	0.000468	0.002799	endopolyphosphatase EC:3.6.1.10
K00384	0.211443	0.00047	0.002813	thioredoxin reductase (NADPH) EC:1.8.1.9
K01711	-0.21137	0.000472	0.002823	GDPmannose 4,6-dehydratase EC:4.2.1.47
K06987	0.2112	0.000477	0.002851	aspartate dehydrogenase EC:1.4.1.21; aspartate dehydrogenase EC:1.4.1.21
K03306	-0.21109	0.000481	0.002868	inorganic phosphate transporter, PiT family
K02537	0.210647	0.000481	0.00287	mitotic spindle assembly checkpoint protein MAD2
K02232	-0.21098	0.000484	0.002881	adenosylcobyric acid synthase EC:6.3.5.10
K08138	-0.21099	0.000484	0.002881	MFS transporter, SP family, xylose_H+ symportor
K01261	0.210418	0.000488	0.002904	glutamyl aminopeptidase EC:3.4.11.7
K09013	0.210658	0.000494	0.002935	Fe-S cluster assembly ATP-binding protein
K01192	-0.21048	0.0005	0.002966	beta-mannosidase EC:3.2.1.25
K14826	0.210009	0.000501	0.002973	FK506-binding nuclear protein EC:5.2.1.8
K02927	0.210325	0.000505	0.002989	large subunit ribosomal protein L40e
K01155	0.21025	0.000507	0.003001	type II restriction enzyme EC:3.1.21.4
K07735	-0.21023	0.000507	0.003002	putative transcriptional regulator
K03269	-0.21019	0.000509	0.003007	UDP-2,3-diacylglucosamine hydrolase EC:3.6.1.54
K01597	0.209709	0.000511	0.003016	diphosphomevalonate decarboxylase EC:4.1.1.33
K10109	0.210066	0.000513	0.003025	maltose/maltodextrin transport system permease protein
K05340	-0.20992	0.000517	0.00305	glucose uptake protein
K06133	0.209874	0.000519	0.003057	4'-phosphopantetheinyl transferase EC:2.7.8.-
K02774	-0.2098	0.000521	0.003067	PTS system, galactitol-specific IIB component EC:2.7.1.69
K08065	0.209379	0.000522	0.003067	nuclear transcription Y subunit beta
K00029	-0.20974	0.000524	0.003076	malate dehydrogenase (oxaloacetate-decarboxylating)(NADP+) EC:1.1.1.40
K07059	0.209662	0.000526	0.003087	UPF0271 protein; UPF0271 protein
K05936	-0.20963	0.000527	0.00309	precorrin-4 C11-methyltransferase EC:2.1.1.133
K12613	0.209158	0.000529	0.003099	mRNA-decapping enzyme subunit 2 EC:3.-.-.-
K13953	0.209552	0.00053	0.003101	alcohol dehydrogenase, propanol-preferring EC:1.1.1.1
K01289	0.209121	0.00053	0.003101	carboxypeptidase EC:3.4.16.-
K02066	-0.20943	0.000533	0.003118	putative ABC transport system permease protein

K03756	-0.20939	0.000535	0.003121	putrescine_ornithine antiporter
K13798	0.208984	0.000535	0.003121	DNA-directed RNA polymerase subunit B EC:2.7.7.6; DNA-directed RNA polymerase subunit B EC:2.7.7.6
K02050	0.209237	0.00054	0.003149	NitT/TauT family transport system permease protein
K14844	0.208806	0.000541	0.00315	pumilio homology domain family member 6
K04706	0.20876	0.000542	0.003157	E3 SUMO-protein ligase PIAS1 EC:6.3.2.-
K02337	0.209045	0.000547	0.003179	DNA polymerase III subunit alpha EC:2.7.7.7
K13821	-0.20903	0.000547	0.003179	proline dehydrogenase / delta 1-pyrroline-5-carboxylate dehydrogenase EC:1.5.99.8 1.5.1.12
K03559	-0.20888	0.000552	0.003206	biopolymer transport protein ExbD
K13243	0.208776	0.000556	0.003224	c-di-GMP-specific phosphodiesterase EC:3.1.4.52
K11527	-0.20875	0.000557	0.003226	two-component system, unclassified family, sensor histidine kinase and response regulator EC:2.7.13.3; two-component system, unclassified family, sensor histidine kinase and response regulator EC:2.7.13.3
K06861	-0.20871	0.000558	0.003233	lipopolysaccharide export system ATP-binding protein EC:3.6.3.-
K08191	-0.20869	0.000559	0.003233	MFS transporter, ACS family, hexuronate transporter
K03355	0.208227	0.000561	0.003242	anaphase-promoting complex subunit 8
K03367	0.208591	0.000562	0.003248	D-alanine--poly(phosphoribitol) ligase subunit 1 EC:6.1.1.13
K14458	0.20812	0.000565	0.003259	2-acylglycerol O-acyltransferase 1 EC:2.3.1.22
K09685	0.208483	0.000566	0.003265	purine operon repressor; purine operon repressor
K01824	0.208054	0.000567	0.003267	cholestenol delta-isomerase EC:5.3.3.5
K08083	0.208392	0.000569	0.003278	two-component system, LytT family, response regulator AlgR
K06179	0.208231	0.000575	0.003308	23S rRNA pseudouridine955/2504/2580 synthase EC:5.4.99.24
K11596	0.20775	0.000578	0.003321	argonaute
K01712	-0.20812	0.000579	0.003322	urocanate hydratase EC:4.2.1.49
K14337	0.207728	0.000579	0.003322	alpha-1,6-mannosyltransferase EC:2.4.1.-
K14810	0.207681	0.00058	0.003327	ATP-dependent RNA helicase DDX56/DBP9 EC:3.6.4.13
K00150	0.207564	0.000585	0.003349	glyceraldehyde-3-phosphate dehydrogenase (NAD(P)) EC:1.2.1.59
K06143	-0.20792	0.000586	0.003352	inner membrane protein
K05665	0.207533	0.000586	0.003352	ATP-binding cassette, subfamily C (CFTR/MRP), member 1
K04076	0.207905	0.000587	0.003353	Lon-like ATP-dependent protease EC:3.4.21.-
K08293	0.207493	0.000587	0.003353	mitogen-activated protein kinase EC:2.7.11.24
K11367	0.207412	0.00059	0.003367	chromodomain-helicase-DNA-binding protein 1 EC:3.6.4.12
K00153	0.207373	0.000592	0.003372	S-(hydroxymethyl)mycothiol dehydrogenase EC:1.1.1.306
K08194	-0.20765	0.000596	0.003396	MFS transporter, ACS family, D-galactonate transporter
K01678	0.20761	0.000598	0.003401	fumarate hydratase subunit beta EC:4.2.1.2
K00257	-0.20752	0.000601	0.003415	alanine dehydrogenase EC:1.4.1.1
K04108	0.207527	0.000601	0.003415	4-hydroxybenzoyl-CoA reductase subunit alpha EC:1.3.7.9
K07544	0.207094	0.000602	0.003417	benzylsuccinate CoA-transferase BbsF subunit EC:2.8.3.15
K07516	0.206997	0.000606	0.003433	3-hydroxyacyl-CoA dehydrogenase EC:1.1.1.35
K08680	0.207402	0.000605	0.003433	2-succinyl-6-hydroxy-2,4-cyclohexadiene-1-carboxylate synthase EC:4.2.99.20
K01073	0.207186	0.000614	0.003474	acyl-CoA hydrolase EC:3.1.2.20
K14306	0.206756	0.000615	0.003479	nuclear pore complex protein Nup62
K09889	-0.20713	0.000616	0.003481	ribosome-associated protein
K02193	-0.207	0.000621	0.003505	heme exporter protein A EC:3.6.3.41

K03725	0.206583	0.000622	0.003508	archaea-specific helicase EC:3.6.1.-; archaea-specific helicase EC:3.6.1.-
K02456	-0.20684	0.000627	0.003534	general secretion pathway protein G
K05879	-0.20677	0.000629	0.003547	dihydroxyacetone kinase, C-terminal domain EC:2.7.1.-
K01491	0.206733	0.000631	0.003553	methylenetetrahydrofolate dehydrogenase (NADP+) / methenyltetrahydrofolate cyclohydrolase EC:1.5.1.5 3.5.4.9
K01440	-0.20632	0.000632	0.003553	nicotinamidase EC:3.5.1.19
K14119	0.206264	0.000634	0.003564	energy-converting hydrogenase B subunit J
K14777	0.206241	0.000635	0.003566	ATP-dependent RNA helicase DDX47/RRP3 EC:3.6.4.13
K14100	0.206185	0.000637	0.003575	energy-converting hydrogenase A subunit I
K07011	-0.20643	0.000643	0.003603	putative hydrolases of HD superfamily; putative hydrolases of HD superfamily
K12735	0.206035	0.000643	0.003603	peptidyl-prolyl cis-trans isomerase-like 4 EC:5.2.1.8
K05668	0.206012	0.000644	0.003605	ATP-binding cassette, subfamily C (CFTR/MRP), member 5
K14188	0.205991	0.000645	0.003607	D-alanine--poly(phosphoribitol) ligase subunit 2 EC:6.1.1.13
K02016	-0.20636	0.000646	0.003609	iron complex transport system substrate-binding protein
K07792	-0.20634	0.000646	0.00361	anaerobic C4-dicarboxylate transporter DcuB
K03326	-0.20632	0.000647	0.003611	C4-dicarboxylate transporter, DcuC family
K13110	0.205919	0.000648	0.003611	microfibrillar-associated protein 1
K00819	-0.20623	0.000651	0.003626	ornithine--oxo-acid transaminase EC:2.6.1.13
K10711	-0.20621	0.000652	0.003628	GntR family transcriptional regulator, frlABCD operon transcriptional regulator
K07129	0.206019	0.000659	0.003665	arylformamidase EC:3.5.1.9; arylformamidase EC:3.5.1.9
K11247	0.205633	0.000659	0.003665	endophilin-A
K02190	-0.206	0.00066	0.003665	sirohydrochlorin cobaltochelatase EC:4.99.1.3
K05352	0.205574	0.000662	0.003672	ribitol-5-phosphate 2-dehydrogenase EC:1.1.1.137
K00243	-0.20584	0.000667	0.003697	hypothetical protein; hypothetical protein
K09713	0.205438	0.000667	0.003697	hypothetical protein; hypothetical protein
K01573	-0.20576	0.00067	0.003705	oxaloacetate decarboxylase, gamma subunit EC:4.1.1.3
K12862	0.205377	0.00067	0.003705	pleiotropic regulator 1
K07723	0.205319	0.000672	0.003715	CopG family transcriptional regulator
K08159	0.205504	0.000681	0.003758	MFS transporter, DHA1 family, L-arabinose/isopropyl-beta-D-thiogalactopyranoside export protein
K13482	0.205008	0.000685	0.003781	xanthine dehydrogenase large subunit EC:1.17.1.4
K14113	0.204857	0.000692	0.003814	energy-converting hydrogenase B subunit D
K01183	-0.20521	0.000693	0.003818	chitinase EC:3.2.1.14
K00320	0.204768	0.000696	0.003829	coenzyme F420-dependent N5,N10-methenyltetrahydromethanopterin reductase EC:1.5.99.11
K08568	0.204707	0.000698	0.00384	cathepsin X EC:3.4.18.1
K12604	0.204598	0.000703	0.003863	CCR4-NOT transcription complex subunit 1
K00051	0.204568	0.000704	0.003867	malate dehydrogenase (NADP+) EC:1.1.1.82
K11884	0.204475	0.000708	0.003886	RNA-binding protein PNO1
K09490	0.204459	0.000709	0.003887	heat shock 70kDa protein 5
K10827	0.2044	0.000712	0.003898	polyamine-transporting ATPase EC:3.6.3.31
K08281	0.204736	0.000713	0.003902	nicotinamidase/pyrazinamidase EC:3.5.1.19 3.5.1.-
K09913	0.204359	0.000713	0.003902	hypothetical protein; hypothetical protein
K07111	0.204303	0.000716	0.003912	UPF0716 protein FxsA; UPF0716 protein FxsA
K01121	0.204665	0.000717	0.003913	2',3'-cyclic-nucleotide 3'-phosphodiesterase EC:3.1.4.37
K02485	0.204261	0.000718	0.003913	two-component system, unclassified family, response regulator
K14818	0.204262	0.000718	0.003913	ribosome assembly protein SQT1

K03218	0.20447	0.000725	0.003951	23S rRNA (guanosine2251-2'-O)-methyltransferase EC:2.1.1.185
K07264	-0.20444	0.000726	0.003955	4-amino-4-deoxy-L-arabinose transferase EC:2.4.2.43
K00969	0.204281	0.000734	0.00399	nicotinate-nucleotide adenylyltransferase EC:2.7.7.18
K03092	-0.20424	0.000735	0.003993	RNA polymerase sigma-54 factor
K07588	-0.20425	0.000735	0.003993	LAO/AO transport system kinase EC:2.7.-.-
K03654	-0.20416	0.000739	0.004007	ATP-dependent DNA helicase RecQ EC:3.6.4.12
K05670	0.203797	0.000738	0.004007	ATP-binding cassette, subfamily C (CFTR/MRP), member 13
K14317	0.203737	0.000741	0.004016	nuclear pore complex protein Nup214
K03321	-0.20371	0.000742	0.004019	sulfate permease, SulP family
K00239	-0.20407	0.000743	0.004021	succinate dehydrogenase flavoprotein subunit EC:1.3.99.1
K07137	0.20394	0.000749	0.004046	molybdenum cofactor cytidylyltransferase EC:2.7.7.76; molybdenum cofactor cytidylyltransferase EC:2.7.7.76
K10394	0.203565	0.000749	0.004046	kinesin family member 3/17
K07787	-0.20378	0.000757	0.004083	Cu(I)/Ag(I) efflux system membrane protein CusA
K00904	0.203321	0.00076	0.004088	deoxyguanosine kinase EC:2.7.1.113
K02042	0.203689	0.000761	0.004088	phosphonate transport system permease protein
K02651	-0.20367	0.000762	0.004088	pilus assembly protein Flp/PilA
K04516	-0.20369	0.000761	0.004088	chorismate mutase EC:5.4.99.5
K07497	0.203667	0.000762	0.004088	putative transposase; putative transposase
K07862	-0.20373	0.000759	0.004088	serine/threonine transporter
K03846	0.20333	0.00076	0.004088	alpha-1,2-mannosyltransferase EC:2.4.1.259 2.4.1.261
K02824	0.203647	0.000762	0.00409	uracil permease
K03531	0.20354	0.000767	0.004113	cell division protein FtsZ
K08479	-0.20337	0.000775	0.004152	two-component system, OmpR family, clock-associated histidine kinase SasA EC:2.7.13.3
K07706	0.203278	0.00078	0.004173	two-component system, AgrA family, sensor histidine kinase AgrC EC:2.7.13.-
K14408	0.202776	0.000786	0.004203	cleavage stimulation factor subunit 3
K11894	0.202721	0.000789	0.004214	type VI secretion system protein ImpI
K02006	0.202971	0.000794	0.004242	cobalt/nickel transport system ATP-binding protein
K07684	-0.20292	0.000797	0.004247	two-component system, NarL family, nitrate/nitrite response regulator NarL
K04073	-0.20257	0.000796	0.004247	acetaldehyde dehydrogenase EC:1.2.1.10
K07730	0.202388	0.000805	0.004288	putative transcriptional regulator; putative transcriptional regulator
K07335	0.202737	0.000806	0.004289	basic membrane protein A and related proteins
K02677	0.202216	0.000814	0.004326	classical protein kinase C EC:2.7.11.13
K01995	0.202227	0.000831	0.004417	branched-chain amino acid transport system ATP-binding protein
K06138	-0.20211	0.000837	0.004444	pyrroloquinoline quinone biosynthesis protein D
K03775	-0.20192	0.000847	0.004493	FKBP-type peptidyl-prolyl cis-trans isomerase SlyD EC:5.2.1.8
K15032	0.201506	0.000849	0.004503	mTERF domain-containing protein, mitochondrial
K00865	-0.20175	0.000856	0.004532	glycerate kinase EC:2.7.1.31
K13507	0.201302	0.00086	0.004553	glycerol-3-phosphate O-acyltransferase / dihydroxyacetone phosphate acyltransferase EC:2.3.1.15 2.3.1.42
K07248	-0.20165	0.000861	0.004553	lactaldehyde dehydrogenase / glycolaldehyde dehydrogenase EC:1.2.1.22 1.2.1.21
K00805	0.20151	0.000868	0.004587	heptaprenyl diphosphate synthase EC:2.5.1.30
K07392	0.201296	0.000879	0.004643	AAA family ATPase
K10761	0.201199	0.000884	0.004663	tRNA(His) guanylyltransferase EC:2.7.7.79

K11826	0.200844	0.000884	0.004663	AP-2 complex subunit mu-1
K05776	-0.20118	0.000885	0.004664	molybdate transport system ATP-binding protein
K10545	0.201143	0.000887	0.004672	D-xylose transport system ATP-binding protein EC:3.6.3.17
K06123	0.200661	0.000894	0.004704	1-acylglycerone phosphate reductase EC:1.1.1.101
K03843	0.200494	0.000903	0.004749	alpha-1,3/alpha-1,6-mannosyltransferase EC:2.4.1.132 2.4.1.257
K01744	-0.20075	0.000909	0.004768	aspartate ammonia-lyase EC:4.3.1.1
K03462	0.200405	0.000908	0.004768	nicotinamide phosphoribosyltransferase EC:2.4.2.12
K05928	0.200388	0.000909	0.004768	tocopherol O-methyltransferase EC:2.1.1.95
K12591	0.200364	0.00091	0.004772	exosome complex exonuclease RRP6 EC:3.1.13.-
K12835	0.200317	0.000913	0.004782	ATP-dependent RNA helicase DDX42 EC:3.6.4.13
K03748	0.200561	0.000919	0.004809	SanA protein
K14559	0.200191	0.00092	0.004811	U3 small nucleolar RNA-associated protein MPP10
K06942	0.200462	0.000924	0.004827	nucleolar GTP-binding protein; nucleolar GTP-binding protein
K07084	-0.20047	0.000924	0.004827	putative transport protein; putative transport protein
K06396	0.200444	0.000925	0.004828	stage III sporulation protein AG
K03107	0.199914	0.000935	0.004877	signal recognition particle subunit SRP68
K06896	-0.20024	0.000937	0.004879	nucleoid-associated protein; nucleoid-associated protein
K06177	-0.20022	0.000938	0.004881	tRNA pseudouridine32 synthase / 23S rRNA pseudouridine746 synthase EC:5.4.99.28 5.4.99.29
K02358	0.200007	0.00095	0.004941	elongation factor Tu
K00763	0.199932	0.000954	0.00496	nicotinate phosphoribosyltransferase EC:2.4.2.11
K00912	-0.19989	0.000956	0.004967	tetraacyldisaccharide 4'-kinase EC:2.7.1.130
K14114	0.199515	0.000958	0.004973	energy-converting hydrogenase B subunit E
K03981	-0.19938	0.000966	0.005008	thiol_disulfide interchange protein DsbC EC:5.3.4.1
K11681	0.199384	0.000966	0.005008	helicase SWR1 EC:3.6.4.12
K01847	-0.19967	0.000969	0.005019	methylmalonyl-CoA mutase EC:5.4.99.2
K02944	0.199087	0.000983	0.005088	large subunit ribosomal protein LX
K06183	0.19933	0.000989	0.005115	16S rRNA pseudouridine516 synthase EC:5.4.99.19
K08688	-0.1987	0.001006	0.005199	creatinase EC:3.5.3.3
K01068	0.198607	0.001012	0.005225	palmitoyl-CoA hydrolase EC:3.1.2.2
K07824	0.198517	0.001017	0.005245	benzoate 4-monooxygenase EC:1.14.13.12
K14843	0.198524	0.001017	0.005245	pescadillo
K02893	0.19849	0.001019	0.00525	large subunit ribosomal protein L23Ae
K06155	-0.19881	0.00102	0.005253	Gnt-I system high-affinity gluconate transporter
K14108	0.198452	0.001021	0.005254	energy-converting hydrogenase A subunit Q
K02022	-0.19844	0.001022	0.005255	HlyD family secretion protein
K02775	-0.19864	0.001031	0.005293	PTS system, galactitol-specific IIC component
K13734	0.19859	0.001034	0.005306	fibronectin-binding protein 1
K01206	-0.19856	0.001035	0.00531	alpha-L-fucosidase EC:3.2.1.51
K03742	0.198385	0.001046	0.005363	competence/damage-inducible protein CinA
K07262	-0.19796	0.001052	0.005383	D-alanyl-D-alanine endopeptidase (penicillin-binding protein 7) EC:3.4.21.-
K01365	0.197965	0.001052	0.005383	cathepsin L EC:3.4.22.15
K03386	-0.19822	0.001057	0.005403	peroxiredoxin (alkyl hydroperoxide reductase subunit C) EC:1.11.1.15
K05546	0.197822	0.001061	0.005419	alpha 1,3-glucosidase EC:3.2.1.84
K01839	0.198136	0.001062	0.005423	phosphopentomutase EC:5.4.2.7
K12761	0.197751	0.001065	0.005435	carbon catabolite-derepressing protein kinase EC:2.7.11.1
K00158	0.19773	0.001066	0.005437	pyruvate oxidase EC:1.2.3.3
K06338	0.197655	0.001071	0.005458	spore coat protein SA

K14746	0.197628	0.001073	0.005462	(S)-1-phenylethanol dehydrogenase EC:1.1.1.311
K03544	0.197937	0.001075	0.005467	ATP-dependent Clp protease ATP-binding subunit ClpX
K08675	0.197437	0.001085	0.005514	Lon-like ATP-dependent protease EC:3.4.21.-
K12392	0.197433	0.001086	0.005514	AP-1 complex subunit beta-1
K01201	-0.19771	0.001089	0.00552	glucosylceramidase EC:3.2.1.45
K09721	0.197383	0.001089	0.00552	hypothetical protein; hypothetical protein
K02209	0.19738	0.001089	0.00552	minichromosome maintenance protein 5 (cell division control protein 46)
K08961	-0.19768	0.001091	0.005525	chondroitin-sulfate-ABC endolyase/exolyase EC:4.2.2.20 4.2.2.21; chondroitin-sulfate-ABC endolyase/exolyase EC:4.2.2.20 4.2.2.21
K01363	0.197331	0.001092	0.005528	cathepsin B EC:3.4.22.1
K01904	0.197231	0.001099	0.005553	4-coumarate--CoA ligase EC:6.2.1.12
K03551	0.197561	0.001099	0.005553	holliday junction DNA helicase RuvB EC:3.6.4.12
K08832	0.197164	0.001103	0.005565	serine/threonine-protein kinase SRPK3 EC:2.7.11.1
K14771	0.197156	0.001104	0.005565	U3 small nucleolar RNA-associated protein 19
K13125	0.197165	0.001103	0.005565	nitric oxide synthase-interacting protein
K13210	0.197123	0.001106	0.005572	far upstream element-binding protein
K07798	-0.19734	0.001114	0.005607	Cu(I)/Ag(I) efflux system membrane protein CusB
K13107	-0.19692	0.001119	0.00563	RNA-binding motif protein, X-linked 2
K03604	0.197214	0.001122	0.005639	LacI family transcriptional regulator, purine nucleotide synthesis repressor
K01684	-0.19714	0.001127	0.005654	galactonate dehydratase EC:4.2.1.6
K10406	0.196822	0.001126	0.005654	kinesin family member C2/C3
K14791	0.196804	0.001127	0.005654	periodic tryptophan protein 1
K15016	0.196698	0.001134	0.005684	3-hydroxybutyryl-CoA dehydratase / 3-hydroxyacyl-CoA dehydrogenase EC:4.2.1.55 1.1.1.35; 3-hydroxybutyryl-CoA dehydratase / 3-hydroxyacyl-CoA dehydrogenase EC:4.2.1.55 1.1.1.35
K01102	0.196689	0.001135	0.005684	pyruvate dehydrogenase phosphatase EC:3.1.3.43
K03147	-0.197	0.001136	0.005687	thiamine biosynthesis protein ThiC
K06236	0.196958	0.001139	0.005696	collagen, type I/II/III/V/XI, alpha
K01677	0.196909	0.001142	0.005709	fumarate hydratase subunit alpha EC:4.2.1.2
K01035	0.196519	0.001146	0.005725	acetate CoA-transferase beta subunit EC:2.8.3.8
K09744	0.19649	0.001148	0.005731	hypothetical protein; hypothetical protein
K06941	0.196785	0.00115	0.005738	23S rRNA (adenine2503-C2)-methyltransferase EC:2.1.1.192
K00647	-0.19676	0.001152	0.005743	3-oxoacyl-acyl-carrier-protein synthase I EC:2.3.1.41
K02371	-0.19659	0.001164	0.005795	enoyl-acyl carrier protein reductase II EC:1.3.1.-
K01479	0.196564	0.001166	0.005801	formiminoglutamase EC:3.5.3.8
K04759	0.19654	0.001167	0.005805	ferrous iron transport protein B
K08297	0.196508	0.001169	0.005811	crotonobetainyl-CoA dehydrogenase EC:1.3.99.-
K14629	0.196168	0.00117	0.005812	enoyl reductase
K02499	0.196468	0.001172	0.005817	tetrapyrrole methylase family protein / MazG family protein
K04518	-0.1964	0.001177	0.005838	prephenate dehydratase EC:4.2.1.51
K03575	-0.19623	0.001189	0.005891	A/G-specific adenine glycosylase EC:3.2.2.-
K07203	0.19579	0.001197	0.005927	FKBP12-rapamycin complex-associated protein
K03061	0.195708	0.001203	0.005951	26S proteasome regulatory subunit T1
K14692	0.19566	0.001206	0.005964	solute carrier family 30 (zinc transporter), member 5
K08054	0.195587	0.001211	0.005986	calnexin
K05520	-0.19589	0.001212	0.005987	protease I EC:3.2.-.-
K00311	0.195539	0.001215	0.00599	electron-transferring-flavoprotein dehydrogenase EC:1.5.5.1

K01738	0.195861	0.001215	0.00599	cysteine synthase A EC:2.5.1.47
K00924	0.195823	0.001217	0.005999	acetate kinase EC:2.7.2.1
K02444	-0.19577	0.001221	0.006012	DeoR family transcriptional regulator, glycerol-3-phosphate regulon repressor
K00687	0.195341	0.001229	0.006041	penicillin-binding protein 2B EC:2.3.2.-
K00946	-0.19568	0.001228	0.006041	thiamine-monophosphate kinase EC:2.7.4.16
K01296	0.195324	0.00123	0.006041	carboxypeptidase M EC:3.4.17.12
K11594	0.195323	0.00123	0.006041	ATP-dependent RNA helicase EC:3.6.4.13
K04024	-0.19553	0.001238	0.006072	ethanolamine utilization protein EutJ
K04517	0.195532	0.001238	0.006072	prephenate dehydrogenase EC:1.3.1.12
K00246	-0.19551	0.00124	0.006074	fumarate reductase subunit C
K12828	0.195182	0.001241	0.006074	splicing factor 3B subunit 1
K02028	0.195459	0.001244	0.006082	polar amino acid transport system ATP-binding protein EC:3.6.3.21
K03065	0.195135	0.001244	0.006082	26S proteasome regulatory subunit T5
K01077	-0.19539	0.001249	0.006103	alkaline phosphatase EC:3.1.3.1
K02328	0.194991	0.001255	0.006125	DNA polymerase delta subunit 2
K00539	-0.19492	0.00126	0.006145	nicotinamide N-methyltransferase EC:2.1.1.1
K12837	0.194914	0.00126	0.006145	splicing factor U2AF 65 kDa subunit
K09717	0.194882	0.001263	0.006146	hypothetical protein; hypothetical protein
K11105	-0.19521	0.001262	0.006146	cell volume regulation protein A
K01900	0.194875	0.001263	0.006146	succinyl-CoA synthetase beta subunit EC:6.2.1.4 6.2.1.5
K01518	0.195163	0.001265	0.006147	bis(5'-nucleosidyl)-tetraphosphatase EC:3.6.1.17
K02227	-0.19515	0.001267	0.006147	adenosylcobinamide-phosphate synthase EC:6.3.1.10
K08773	-0.19513	0.001268	0.006147	RalA-binding protein 1
K12617	-0.19482	0.001268	0.006147	DNA topoisomerase 2-associated protein PAT1
K03845	0.194812	0.001268	0.006147	alpha-1,3-mannosyltransferase EC:2.4.1.258
K02517	-0.1951	0.00127	0.006152	lipid A biosynthesis lauroyl acyltransferase EC:2.3.1.-
K07977	0.194751	0.001273	0.00616	Arf/Sar family, other
K07227	-0.19467	0.001279	0.006185	hypothetical protein
K02210	0.194628	0.001282	0.006196	minichromosome maintenance protein 7 (cell division control protein 47)
K12854	0.194569	0.001286	0.006214	pre-mRNA-splicing helicase BRR2 EC:3.6.4.13
K06999	-0.19484	0.00129	0.006226	phospholipase/carboxylesterase; phospholipase/carboxylesterase
K00794	-0.19481	0.001292	0.006231	6,7-dimethyl-8-ribityllumazine synthase EC:2.5.1.78
K00604	0.194788	0.001294	0.006236	methionyl-tRNA formyltransferase EC:2.1.2.9
K08641	-0.19477	0.001295	0.006239	D-alanyl-D-alanine dipeptidase EC:3.4.13.22
K00210	-0.19474	0.001297	0.006246	prephenate dehydrogenase EC:1.3.1.12
K02057	0.194609	0.001307	0.006284	simple sugar transport system permease protein
K02327	0.1943	0.001307	0.006284	DNA polymerase delta subunit 1 EC:2.7.7.7
K05362	0.194535	0.001313	0.006306	UDP-N-acetylmuramoyl-L-alanyl-D-glutamate-L-lysine ligase EC:6.3.2.7
K01421	0.194384	0.001324	0.006358	putative membrane protein
K00532	0.194349	0.001327	0.006367	ferredoxin hydrogenase EC:1.12.7.2
K02937	0.194023	0.001328	0.006367	large subunit ribosomal protein L7e
K00814	0.193937	0.001335	0.006395	alanine transaminase EC:2.6.1.2
K01431	0.193581	0.001363	0.006521	beta-ureidopropionase EC:3.5.1.6
K02331	0.193591	0.001362	0.006521	DNA polymerase phi subunit EC:2.7.7.7
K04651	0.193778	0.001372	0.006559	hydrogenase nickel incorporation protein HypA/HybF
K13806	0.193443	0.001374	0.006565	sn1-specific diacylglycerol lipase EC:3.1.1.-
K14445	-0.1937	0.001379	0.006581	solute carrier family 13 (sodium-dependent dicarboxylate transporter), member 2/3/5

K03379	0.193339	0.001383	0.006595	cyclohexanone monooxygenase EC:1.14.13.22
K03534	-0.19355	0.00139	0.006628	L-rhamnose mutarotase EC:5.1.3.-
K05592	-0.19345	0.001398	0.006661	ATP-dependent RNA helicase DeaD EC:3.6.4.13
K00588	0.193398	0.001403	0.006678	caffeoyl-CoA O-methyltransferase EC:2.1.1.104
K13601	0.192937	0.001415	0.006734	bacteriochlorophyll C8 methyltransferase EC:2.1.1.-
K00729	0.192884	0.00142	0.00675	dolichyl-phosphate beta-glucosyltransferase EC:2.4.1.117
K02435	0.193169	0.001421	0.006753	aspartyl-tRNA(Asn)/glutamyl-tRNA (Gln) amidotransferase subunit C EC:6.3.5.6 6.3.5.7
K03205	0.193155	0.001423	0.006754	type IV secretion system protein VirD4
K03538	0.192835	0.001424	0.006755	ribonuclease P protein subunit POP4 EC:3.1.26.5
K02882	0.192805	0.001426	0.006757	large subunit ribosomal protein L18Ae
K07813	0.193118	0.001426	0.006757	accessory gene regulator B
K10268	0.192765	0.00143	0.006769	F-box and leucine-rich repeat protein 2/20
K07814	0.192987	0.001437	0.006797	putative two-component system response regulator; putative two-component system response regulator
K01860	0.192671	0.001438	0.006797	chloromuconate cycloisomerase EC:5.5.1.7
K07115	-0.1929	0.001444	0.006823	acyl-homoserine-lactone acylase EC:3.5.1.97; acyl-homoserine-lactone acylase EC:3.5.1.97
K05868	0.192545	0.001448	0.006837	cyclin B
K14846	0.192463	0.001455	0.006865	ribosome production factor 1
K01814	-0.1927	0.001461	0.006888	phosphoribosylformimino-5-aminoimidazole carboxamide ribotide isomerase EC:5.3.1.16
K09707	0.192566	0.001472	0.006936	hypothetical protein; hypothetical protein
K02523	-0.19249	0.001478	0.006956	octaprenyl-diphosphate synthase EC:2.5.1.90
K13041	-0.1922	0.001477	0.006956	two-component system, LuxR family, response regulator TtrR
K01281	0.192451	0.001482	0.006962	X-Pro dipeptidyl-peptidase EC:3.4.14.11
K03722	0.19246	0.001481	0.006962	ATP-dependent DNA helicase DinG EC:3.6.4.12
K08257	-0.19244	0.001482	0.006962	mannan endo-1,6-alpha-mannosidase EC:3.2.1.101
K01056	0.192409	0.001485	0.00697	peptidyl-tRNA hydrolase, PTH1 family EC:3.1.1.29
K02726	0.192091	0.001487	0.006973	20S proteasome subunit alpha 2 EC:3.4.25.1
K00309	0.192052	0.00149	0.006984	pyrimidodiazepine synthase EC:1.5.4.1
K00037	0.192233	0.001501	0.007027	3-alpha-hydroxysteroid dehydrogenase EC:1.1.1.50
K01253	0.191894	0.001504	0.007039	microsomal epoxide hydrolase EC:3.3.2.9
K06669	0.191757	0.001516	0.00709	structural maintenance of chromosome 3 (chondroitin sulfate proteoglycan 6)
K11022	-0.19196	0.001524	0.007122	structural toxin protein (hemagglutinin/hemolysin) RtxA
K00793	-0.19184	0.001535	0.007168	riboflavin synthase EC:2.5.1.9
K00950	-0.19182	0.001537	0.00717	2-amino-4-hydroxy-6-hydroxymethyldihydropteridine diphosphokinase EC:2.7.6.3
K03305	-0.19181	0.001537	0.00717	proton-dependent oligopeptide transporter, POT family
K03088	-0.19163	0.001554	0.007226	RNA polymerase sigma-70 factor, ECF subfamily
K03609	0.191638	0.001553	0.007226	septum site-determining protein MinD
K10797	0.191642	0.001553	0.007226	2-enoate reductase EC:1.3.1.31
K10737	0.191347	0.001552	0.007226	minichromosome maintenance protein 8
K00853	-0.19156	0.00156	0.007252	L-ribulokinase EC:2.7.1.16
K01136	-0.19149	0.001566	0.007277	iduronate 2-sulfatase EC:3.1.6.13
K02377	-0.19147	0.001568	0.007278	GDP-L-fucose synthase EC:1.1.1.271
K12278	0.191085	0.001576	0.007312	MSHA biogenesis protein MshG
K14205	-0.19118	0.001595	0.007393	phosphatidylglycerol lysyltransferase EC:2.3.2.3
K02224	-0.19107	0.001604	0.007428	cobyrinic acid a,c-diamide synthase EC:6.3.5.9 6.3.5.11
K03567	-0.19108	0.001604	0.007428	glycine cleavage system transcriptional repressor
K00945	0.190888	0.001621	0.007498	cytidylate kinase EC:2.7.4.14

K02596	0.190592	0.001622	0.007498	nitrogen fixation protein NifX
K08365	0.190741	0.001635	0.007549	MerR family transcriptional regulator, mercuric resistance operon regulatory protein
K13787	0.190745	0.001635	0.007549	geranylgeranyl diphosphate synthase, type I EC:2.5.1.1 2.5.1.10 2.5.1.29
K05692	0.190422	0.001638	0.007557	actin beta/gamma 1
K01207	0.190448	0.001663	0.007667	beta-N-acetylhexosaminidase EC:3.2.1.52
K02240	0.190395	0.001668	0.007685	competence protein ComFA
K09533	0.190039	0.001674	0.00771	DnaJ homolog subfamily C member 13
K03666	0.190309	0.001676	0.007713	host factor-I protein
K07012	0.19028	0.001679	0.00772	putative hydrolases of HD superfamily; putative hydrolases of HD superfamily
K09553	0.189905	0.001687	0.007754	stress-induced-phosphoprotein 1
K08955	0.189742	0.001703	0.007822	ATP-dependent metalloprotease EC:3.4.24.-
K12614	0.18949	0.001728	0.007931	ATP-dependent RNA helicase DDX6/DHH1 EC:3.6.4.13
K13488	0.189449	0.001732	0.007944	chemotaxis-related protein WspB
K09825	0.189716	0.001734	0.007947	Fur family transcriptional regulator, peroxide stress response regulator
K02997	0.189408	0.001736	0.007952	small subunit ribosomal protein S9e
K10858	0.189394	0.001737	0.007953	DNA mismatch repair protein PMS2
K14571	0.189273	0.00175	0.008003	ribosome biogenesis ATPase
K12396	0.189151	0.001762	0.008054	AP-3 complex subunit delta-1
K00777	0.189403	0.001765	0.008063	beta-galactoside alpha-2,6-sialyltransferase (sialyltransferase 1) EC:2.4.99.1
K03634	-0.18934	0.001771	0.008087	outer membrane lipoprotein carrier protein
K00382	-0.18929	0.001777	0.008106	dihydrolipoamide dehydrogenase EC:1.8.1.4
K01803	0.189261	0.001779	0.008112	triosephosphate isomerase (TIM) EC:5.3.1.1
K00290	-0.18923	0.001783	0.008123	saccharopine dehydrogenase (NAD+, L-lysine forming) EC:1.5.1.7
K09761	0.189135	0.001792	0.00816	16S rRNA (uracil1498-N3)-methyltransferase EC:2.1.1.193
K00956	-0.1891	0.001796	0.008167	sulfate adenylyltransferase subunit 1 EC:2.7.7.4
K14803	0.188814	0.001796	0.008167	protein phosphatase 2C homolog 2/3 EC:3.1.3.16
K01000	0.188985	0.001807	0.008208	phospho-N-acetylmuramoyl-pentapeptide-transferase EC:2.7.8.13
K14805	0.188708	0.001807	0.008208	ATP-dependent RNA helicase DDX24/MAK5 EC:3.6.4.13
K07010	0.188947	0.001811	0.008221	putative glutamine amidotransferase; putative glutamine amidotransferase
K09748	0.188917	0.001814	0.008229	ribosome maturation factor RimP
K07563	0.188603	0.001818	0.00824	
K15190	0.188587	0.00182	0.008242	7SK snRNA methylphosphate capping enzyme EC:2.1.1.-
K04086	0.188544	0.001824	0.008256	ATP-dependent Clp protease ATP-binding subunit ClpL
K07127	0.188515	0.001827	0.00826	5-hydroxyisourate hydrolase EC:3.5.2.17
K05765	0.188513	0.001827	0.00826	cofilin
K13013	-0.18874	0.001832	0.008278	O-antigen biosynthesis protein WbqV
K03733	-0.18872	0.001835	0.008285	integrase/recombinase XerC
K14837	0.188279	0.001852	0.008355	nucleolar protein 12
K01930	0.188241	0.001856	0.008368	folylpolyglutamate synthase EC:6.3.2.17
K07705	0.188382	0.00187	0.008427	two-component system, LytT family, response regulator LytT
K02907	0.188319	0.001877	0.008434	large subunit ribosomal protein L30
K03385	-0.18831	0.001878	0.008434	cytochrome c-552 EC:1.7.2.2
K05802	-0.18832	0.001877	0.008434	potassium efflux system protein KefA
K05847	-0.1883	0.001879	0.008434	osmoprotectant transport system ATP-binding protein

K07161	0.188338	0.001875	0.008434	transmembrane sensor; transmembrane sensor
K00508	-0.18806	0.001875	0.008434	linoleoyl-CoA desaturase EC:1.14.19.3
K00818	-0.18825	0.001884	0.008442	acetylornithine aminotransferase EC:2.6.1.11
K01997	0.188248	0.001885	0.008442	branched-chain amino acid transport system permease protein
K13694	-0.18825	0.001885	0.008442	lipoprotein Spr
K01534	0.188208	0.001889	0.00845	Cd2+/Zn2+-exporting ATPase EC:3.6.3.3 3.6.3.5
K02870	0.187937	0.001888	0.00845	large subunit ribosomal protein L12e
K03943	0.187861	0.001896	0.008477	NADH dehydrogenase (ubiquinone) flavoprotein 2 EC:1.6.5.3 1.6.99.3
K14753	0.187853	0.001897	0.008477	guanine nucleotide-binding protein subunit beta-2-like 1 protein
K06897	-0.18811	0.001899	0.008479	nucleoid-associated protein; nucleoid-associated protein
K02478	-0.18805	0.001906	0.008506	two-component system, LytT family, sensor kinase EC:2.7.13.3; two-component system, LytT family, sensor kinase EC:2.7.13.3
K02044	0.187965	0.001915	0.00854	phosphonate transport system substrate-binding protein
K00557	0.187914	0.001921	0.008548	tRNA (uracil-5-)-methyltransferase EC:2.1.1.35
K03709	0.187918	0.00192	0.008548	DtxR family transcriptional regulator, Mn-dependent transcriptional regulator
K10838	-0.18765	0.00192	0.008548	xeroderma pigmentosum group C-complementing protein
K03455	-0.18789	0.001923	0.008554	monovalent cation_H+ antiporter-2, CPA2 family
K02856	-0.18785	0.001927	0.008558	L-rhamnose-H+ transport protein; L-rhamnose-H+ transport protein
K06063	0.187572	0.001928	0.008558	SNW domain-containing protein 1
K12585	0.187578	0.001927	0.008558	exosome complex exonuclease DIS3/RRP44 EC:3.1.13.-
K03060	0.187831	0.00193	0.00856	DNA-directed RNA polymerase subunit omega EC:2.7.7.6
K00528	0.187789	0.001934	0.008575	ferredoxin--NADP+ reductase EC:1.18.1.2
K11320	0.187447	0.001942	0.008603	E1A-binding protein p400 EC:3.6.4.-
K09516	-0.18765	0.001949	0.008631	all-trans-retinol 13,14-reductase EC:1.3.99.23
K09820	0.187324	0.001955	0.008652	manganese/iron transport system ATP-binding protein
K14138	0.187547	0.001961	0.008671	carbon monoxide dehydrogenase / acetyl-CoA synthase subunit alpha EC:1.2.7.4 1.2.99.2 2.3.1.169
K07003	0.187486	0.001968	0.008695	glutamine amidotransferase; glutamine amidotransferase
K00341	-0.18745	0.001971	0.008705	NADH-quinone oxidoreductase subunit L EC:1.6.5.3
K11357	-0.18744	0.001972	0.008705	two-component system, cell cycle sensor histidine kinase DivJ EC:2.7.13.3
K09486	0.187084	0.001982	0.008742	hypoxia up-regulated 1
K12479	0.187072	0.001983	0.008743	vacuolar protein sorting-associated protein 45
K10783	0.187291	0.001989	0.008758	trans-2-enoyl-CoA reductase (NAD+) EC:1.3.1.44
K03164	0.187019	0.001989	0.008758	DNA topoisomerase II EC:5.99.1.3
K04478	0.186989	0.001993	0.008767	monofunctional glycosyltransferase EC:2.4.1.-
K01775	0.187172	0.002003	0.0088	alanine racemase EC:5.1.1.1
K12107	0.187181	0.002002	0.0088	cag pathogenicity island protein 22
K06147	0.186964	0.002026	0.008898	ATP-binding cassette, subfamily B, bacterial
K02647	0.186905	0.002033	0.008901	carbohydrate diacid regulator
K03046	0.186937	0.002029	0.008901	DNA-directed RNA polymerase subunit beta' EC:2.7.7.6
K03491	-0.18692	0.002031	0.008901	lichenan operon transcriptional antiterminator
K03925	0.186902	0.002033	0.008901	MraZ protein
K04020	-0.18663	0.002033	0.008901	phosphotransacetylase
K03145	0.186612	0.002036	0.008905	transcription elongation factor S-II
K07029	0.186457	0.002054	0.008978	D-glycero-alpha-D-manno-heptose-7-phosphate kinase EC:2.7.1.168; D-glycero-alpha-D-manno-heptose-7-phosphate kinase EC:2.7.1.168

K07720	-0.18662	0.002065	0.009017	two-component system, response regulator YesN
K10610	0.186359	0.002065	0.009017	DNA damage-binding protein 1
K00062	-0.18659	0.002069	0.009027	D-arabinose 1-dehydrogenase EC:1.1.1.116
K06022	-0.18657	0.002071	0.009031	molybdate-transporting ATPase EC:3.6.3.29
K00067	-0.18651	0.002078	0.009056	dTDP-4-dehydrorhamnose reductase EC:1.1.1.133
K03814	-0.18637	0.002094	0.009122	monofunctional biosynthetic peptidoglycan transglycosylase EC:2.4.1.-
K13040	-0.18608	0.002098	0.009131	two-component system, LuxR family, sensor histidine kinase TtrS EC:2.7.13.3
K03718	-0.18631	0.002102	0.009143	Lrp/AsnC family transcriptional regulator, regulator for asnA, asnC and gidA
K01528	0.185846	0.002126	0.009241	dynamin GTPase EC:3.6.5.5
K08818	0.185834	0.002127	0.009241	cell division cycle 2-like EC:2.7.11.22
K12700	0.18607	0.002131	0.00925	non-specific ribonclеoside hydrolase EC:3.2.-.-
K00748	-0.18562	0.002153	0.009328	lipid-A-disaccharide synthase EC:2.4.1.182
K00757	-0.18589	0.002153	0.009328	uridine phosphorylase EC:2.4.2.3
K01883	0.185904	0.002151	0.009328	cysteinyl-tRNA synthetase EC:6.1.1.16
K04763	-0.18582	0.002161	0.00935	integrase/recombinase XerD
K13530	-0.18582	0.00216	0.00935	AraC family transcriptional regulator, regulatory protein of adaptative response / methylphosphotriester-DNA alkyltransferase methyltransferase EC:2.1.1.-
K12495	0.185548	0.002162	0.00935	IQ motif and SEC7 domain-containing protein
K00830	0.185482	0.00217	0.009378	alanine-glyoxylate transaminase / serine-glyoxylate transaminase / serine-pyruvate transaminase EC:2.6.1.44 2.6.1.45 2.6.1.51
K05390	0.185473	0.002171	0.009378	potassium large conductance calcium-activated channel subfamily M alpha, invertebrate
K00034	0.185654	0.002181	0.009407	glucose 1-dehydrogenase EC:1.1.1.47
K00938	0.185384	0.002182	0.009407	phosphomevalonate kinase EC:2.7.4.2
K06928	0.185658	0.00218	0.009407	nucleoside-triphosphatase THEP1 EC:3.6.1.15; nucleoside-triphosphatase THEP1 EC:3.6.1.15
K11530	0.185369	0.002184	0.009409	autoinducer 2-degrading protein
K07701	0.185335	0.002188	0.009421	two-component system, CitB family, sensor histidine kinase DcuS EC:2.7.13.3
K13103	0.18531	0.002191	0.009429	tuftelin-interacting protein 11
K10846	0.185248	0.002198	0.009456	DNA excision repair protein ERCC-5
K09690	0.185484	0.002202	0.009463	lipopolysaccharide transport system permease protein
K01571	0.185455	0.002205	0.009473	oxaloacetate decarboxylase, alpha subunit EC:4.1.1.3
K02836	0.185359	0.002217	0.009518	peptide chain release factor 2
K03677	-0.18533	0.002221	0.009527	
K08316	0.185319	0.002222	0.009527	16S rRNA (guanine966-N2)-methyltransferase EC:2.1.1.171
K14715	0.184965	0.002234	0.009571	solute carrier family 39 (zinc transporter), member 9
K11644	0.184844	0.002249	0.00963	paired amphipathic helix protein Sin3a
K05540	0.185034	0.002258	0.009662	tRNA-dihydrouridine synthase B EC:1.-.-.-
K07217	-0.185	0.002262	0.009673	Mn-containing catalase; Mn-containing catalase
K03390	0.184938	0.00227	0.00969	heterodisulfide reductase subunit C EC:1.8.98.1
K05787	0.184689	0.002269	0.00969	DNA-binding protein HU-alpha
K10356	0.184686	0.002269	0.00969	myosin I
K00611	0.18492	0.002272	0.009693	ornithine carbamoyltransferase EC:2.1.3.3
K01027	0.184626	0.002277	0.009703	3-oxoacid CoA-transferase EC:2.8.3.5
K05722	0.184621	0.002277	0.009703	FYVE, RhoGEF and PH domain containing 3
K12829	0.184446	0.0023	0.009793	splicing factor 3B subunit 2

K14387	0.184419	0.002303	0.009802	solute carrier family 5 (high affinity choline transporter), member 7
K02029	0.18466	0.002305	0.009804	polar amino acid transport system permease protein
K05968	-0.18464	0.002307	0.009808	sialate O-acetylesterase EC:3.1.1.53
K00353	-0.18451	0.002325	0.009875	NADPH2 dehydrogenase EC:1.6.99.1
K04566	0.184498	0.002326	0.009875	lysyl-tRNA synthetase, class I EC:6.1.1.6
K08289	-0.18449	0.002327	0.009875	phosphoribosylglycinamide formyltransferase 2 EC:2.1.2.2
K10956	0.18418	0.002334	0.009897	protein transport protein SEC61 subunit alpha
K12400	0.184156	0.002337	0.009904	AP-4 complex subunit epsilon-1
K12524	-0.18437	0.002342	0.00992	bifunctional aspartokinase / homoserine dehydrogenase 1 EC:2.7.2.4 1.1.1.3
K03280	0.184055	0.00235	0.009948	UDP-N-acetylglucosamine_(glucosyl)LPS alpha-1,2-N-acetylglucosaminyltransferase EC:2.4.1.56
K03599	-0.18417	0.002369	0.010017	RNA polymerase-associated protein
K05685	0.184163	0.00237	0.010017	macrolide transport system ATP-binding/permease protein EC:3.6.3.-
K11928	0.184153	0.002371	0.010017	sodium/proline symporter
K05914	0.183798	0.002385	0.010067	nitric oxide dioxygenase EC:1.14.12.17
K06180	0.184009	0.00239	0.010085	23S rRNA pseudouridine1911/1915/1917 synthase EC:5.4.99.23
K01796	0.183917	0.002402	0.01013	alpha-methylacyl-CoA racemase EC:5.1.99.4
K07108	0.183636	0.002406	0.01014	UPF0716 protein FxsA; UPF0716 protein FxsA
K02282	0.183855	0.00241	0.010152	pilus assembly protein CpaE
K02519	0.183741	0.002426	0.010205	translation initiation factor IF-2
K02924	0.183489	0.002426	0.010205	large subunit ribosomal protein L39e
K03113	-0.18371	0.002431	0.010218	translation initiation factor 1
K00703	0.183366	0.002477	0.0104	starch synthase EC:2.4.1.21
K07755	0.183116	0.002477	0.0104	arsenite methyltransferase EC:2.1.1.137
K03936	0.183081	0.002482	0.010414	NADH dehydrogenase (ubiquinone) Fe-S protein 3 EC:1.6.5.3 1.6.99.3
K14684	0.183058	0.002485	0.010421	solute carrier family 25 (mitochondrial phosphate transporter), member 23/24/25/41
K09133	-0.18328	0.002488	0.010429	hypothetical protein; hypothetical protein
K04523	0.182926	0.002503	0.010485	ubiquilin
K10680	0.18291	0.002505	0.010487	N-ethylmaleimide reductase EC:1.-.-.-
K09766	0.183128	0.002509	0.010497	hypothetical protein; hypothetical protein
K14940	0.182872	0.002511	0.010497	gamma-F420-2_alpha-L-glutamate ligase EC:6.3.2.32
K05961	0.182853	0.002513	0.010502	deoxynucleoside kinase EC:2.7.1.145
K01899	0.182833	0.002516	0.010507	succinyl-CoA synthetase alpha subunit EC:6.2.1.4 6.2.1.5
K01196	0.182812	0.002519	0.010513	glycogen debranching enzyme EC:2.4.1.25 3.2.1.33
K00053	-0.18289	0.002542	0.010597	ketol-acid reductoisomerase EC:1.1.1.86
K11422	0.182649	0.002542	0.010597	histone-lysine N-methyltransferase SETD1 EC:2.1.1.43
K02799	-0.18281	0.002553	0.010637	PTS system, mannitol-specific IIB component EC:2.7.1.69
K09693	0.182686	0.002571	0.010706	teichoic acid transport system ATP-binding protein EC:3.6.3.40
K05820	0.182664	0.002575	0.010713	MFS transporter, PPP family, 3-phenylpropionic acid transporter
K00548	-0.18264	0.002578	0.010722	5-methyltetrahydrofolate--homocysteine methyltransferase EC:2.1.1.13
K03032	0.182341	0.002586	0.010746	26S proteasome regulatory subunit N2
K09488	0.182322	0.002588	0.010751	TNF receptor-associated protein 1
K13274	0.18253	0.002594	0.010766	cell wall-associated protease EC:3.4.21.-
K03465	0.182506	0.002597	0.010768	thymidylate synthase (FAD) EC:2.1.1.148

K03500	0.182494	0.002599	0.010768	16S rRNA (cytosine967-C5)-methyltransferase EC:2.1.1.176
K06076	-0.1825	0.002598	0.010768	long-chain fatty acid transport protein
K08066	0.182188	0.002608	0.010798	nuclear transcription factor Y, gamma
K02372	-0.18241	0.00261	0.010803	3R-hydroxymyristoyl ACP dehydrase EC:4.2.1.-
K07460	0.182383	0.002615	0.010815	putative endonuclease
K07590	0.182059	0.002626	0.01085	large subunit ribosomal protein L7A
K10815	0.18206	0.002626	0.01085	hydrogen cyanide synthase HcnB EC:1.4.99.5
K07316	0.182276	0.00263	0.010859	adenine-specific DNA-methyltransferase EC:2.1.1.72
K12815	0.182019	0.002632	0.01086	pre-mRNA-splicing factor ATP-dependent RNA helicase PRP16 EC:3.6.4.13
K13677	0.18197	0.002639	0.010883	1,2-diacylglycerol-3-glucose alpha-1,2-glucosyltransferase EC:2.4.1.208
K08857	0.181951	0.002642	0.010888	NIMA (never in mitosis gene a)-related kinase EC:2.7.11.1
K01706	-0.18217	0.002646	0.010898	glucarate dehydratase EC:4.2.1.40
K00661	-0.18214	0.00265	0.010906	maltose O-acetyltransferase EC:2.3.1.79
K07110	0.181806	0.002663	0.01095	UPF0716 protein FxsA; UPF0716 protein FxsA
K04464	0.181813	0.002662	0.01095	mitogen-activated protein kinase 7 EC:2.7.11.24
K00882	0.181974	0.002674	0.010988	1-phosphofructokinase EC:2.7.1.56
K08853	0.181698	0.002679	0.011002	AP2-associated kinase EC:2.7.11.1
K04093	0.181622	0.002691	0.011042	chorismate mutase EC:5.4.99.5
K03030	0.181586	0.002696	0.011057	26S proteasome regulatory subunit N11
K14797	0.181553	0.002701	0.011071	essential nuclear protein 1
K07311	-0.18171	0.002713	0.011116	Tat-targeted selenate reductase subunit YnfG
K03649	0.18147	0.002749	0.011256	TDG/mug DNA glycosylase family protein EC:3.2.2.-
K01921	0.181431	0.002755	0.011272	D-alanine-D-alanine ligase EC:6.3.2.4
K15112	0.181182	0.002756	0.011272	solute carrier family 25 (mitochondrial uncoupling protein), member 27
K03724	0.181315	0.002773	0.011325	ATP-dependent helicase Lhr and Lhr-like helicase EC:3.6.4.-
K14665	-0.18132	0.002772	0.011325	amidohydrolase EC:3.5.1.-
K01110	0.181057	0.002776	0.011327	phosphatidylinositol-3,4,5-trisphosphate 3-phosphatase EC:3.1.3.67
K02953	0.181049	0.002777	0.011327	small subunit ribosomal protein S13e
K14676	0.181042	0.002778	0.011327	lysophospholipid hydrolase EC:3.1.1.5
K04034	-0.18115	0.002798	0.011401	anaerobic magnesium-protoporphyrin IX monomethyl ester cyclase EC:4.-.-.-
K01051	-0.18111	0.002804	0.011418	pectinesterase EC:3.1.1.11
K02180	0.180825	0.002811	0.011442	cell cycle arrest protein BUB3
K02854	0.180986	0.002823	0.011483	AraC family transcriptional regulator, L-rhamnose operon transcriptional activator RhaR
K01620	-0.18095	0.002829	0.011499	threonine aldolase EC:4.1.2.5
K05822	0.180696	0.002831	0.011503	tetrahydrodipicolinate N-acetyltransferase EC:2.3.1.89
K10873	0.180566	0.002851	0.011578	DNA repair and recombination protein RAD52
K07051	0.180514	0.00286	0.011605	tRNA wybutosine-synthesizing protein 2 EC:2.1.1.-; tRNA wybutosine-synthesizing protein 2 EC:2.1.1.-
K02147	0.180416	0.002875	0.011661	V-type H+-transporting ATPase subunit B EC:3.6.3.14
K04788	0.180398	0.002878	0.011666	mycobactin phenyloxazoline synthetase
K05869	0.180233	0.002904	0.011765	calcium/calmodulin-dependent protein kinase IV EC:2.7.11.17
K02150	0.180214	0.002907	0.01177	V-type H+-transporting ATPase subunit E EC:3.6.3.14
K12682	-0.18043	0.002909	0.011771	tracheal colonization factor
K11593	0.180189	0.002911	0.011772	eukaryotic translation initiation factor 2C
K10954	0.180166	0.002915	0.011773	zona occludens toxin

K03942	0.180167	0.002914	0.011773	NADH dehydrogenase (ubiquinone) flavoprotein 1 EC:1.6.5.3 1.6.99.3
K11614	0.180386	0.002917	0.011775	two-component system, CitB family, sensor histidine kinase MalK EC:2.7.13.3
K00064	-0.18014	0.002919	0.011776	D-threo-aldose 1-dehydrogenase EC:1.1.1.122
K00343	-0.18033	0.002925	0.011776	NADH-quinone oxidoreductase subunit N EC:1.6.5.3
K03754	0.180104	0.002925	0.011776	translation initiation factor eIF-2B subunit beta
K02184	0.180098	0.002926	0.011776	formin 2
K05284	0.180119	0.002922	0.011776	phosphatidylinositol glycan, class M EC:2.4.1.-
K01179	0.180319	0.002928	0.011776	endoglucanase EC:3.2.1.4
K01452	0.180288	0.002933	0.011776	chitin deacetylase EC:3.5.1.41
K02981	0.180066	0.002931	0.011776	small subunit ribosomal protein S2e
K08825	0.180059	0.002932	0.011776	dual-specificity tyrosine-(Y)-phosphorylation regulated kinase EC:2.7.12.1
K04045	0.180255	0.002938	0.01179	molecular chaperone HscC
K06441	-0.18001	0.002939	0.01179	ferredoxin hydrogenase gamma subunit EC:1.12.7.2
K01673	-0.18023	0.002942	0.011791	carbonic anhydrase EC:4.2.1.1
K06207	0.180223	0.002943	0.011791	GTP-binding protein
K07375	0.179926	0.002953	0.011825	tubulin beta
K01887	0.180102	0.002962	0.011848	arginyl-tRNA synthetase EC:6.1.1.19
K07019	-0.18011	0.002962	0.011848	putative hydrolases of HD superfamily; putative hydrolases of HD superfamily
K01099	0.179836	0.002968	0.011862	phosphatidylinositol-bisphosphatase EC:3.1.3.36
K14537	0.179753	0.002981	0.01191	nuclear GTP-binding protein
K06628	0.179722	0.002986	0.011923	cell division control protein 45
K12571	0.17968	0.002993	0.011944	PAB-dependent poly(A)-specific ribonuclease subunit 2 EC:3.1.13.4
K01069	0.17976	0.003018	0.011995	hydroxyacylglutathione hydrolase EC:3.1.2.6
K06281	-0.1798	0.003011	0.011995	hydrogenase large subunit EC:1.12.99.6
K09498	0.179538	0.003017	0.011995	T-complex protein 1 subunit zeta
K12819	0.179567	0.003012	0.011995	pre-mRNA-processing factor SLU7
K14539	0.179537	0.003017	0.011995	large subunit GTPase 1 EC:3.6.1.-
K14788	0.179527	0.003018	0.011995	ribosome biogenesis protein ENP2
K14792	0.179539	0.003016	0.011995	rRNA biogenesis protein RRP5
K02911	0.179731	0.003023	0.012003	large subunit ribosomal protein L32
K03668	-0.17973	0.003024	0.012003	heat shock protein HslJ
K00346	-0.17964	0.003038	0.01204	Na+-transporting NADH_ubiquinone oxidoreductase subunit A EC:1.6.5.-
K02564	-0.17965	0.003037	0.01204	glucosamine-6-phosphate deaminase EC:3.5.99.6
K14555	0.179419	0.003036	0.01204	U3 small nucleolar RNA-associated protein 13
K00649	0.179385	0.003042	0.012047	glyceronephosphate O-acyltransferase EC:2.3.1.42
K02955	0.179319	0.003053	0.012084	small subunit ribosomal protein S14e
K01805	-0.17953	0.003056	0.012084	xylose isomerase EC:5.3.1.5
K12506	0.179527	0.003056	0.012084	2-C-methyl-D-erythritol 4-phosphate cytidylyltransferase / 2-C-methyl-D-erythritol 2,4-cyclodiphosphate synthase EC:2.7.7.60 4.6.1.12
K00380	-0.17949	0.003062	0.012098	sulfite reductase (NADPH) flavoprotein alpha-component EC:1.8.1.2
K07344	-0.17917	0.003077	0.012153	type IV secretion system protein TrbL
K02072	0.179251	0.003103	0.012238	D-methionine transport system permease protein
K07076	-0.17926	0.003102	0.012238	UPF0755 protein; UPF0755 protein
K09650	-0.17918	0.003115	0.012265	rhomboid-like protein EC:3.4.21.105
K02732	0.178956	0.003114	0.012265	20S proteasome subunit beta 6 EC:3.4.25.1

K14997	0.178955	0.003114	0.012265	solute carrier family 38 (sodium-coupled neutral amino acid transporter), member 11
K06079	-0.1791	0.003127	0.012299	copper homeostasis protein (lipoprotein)
K13998	0.178869	0.003129	0.012299	dihydrofolate reductase / thymidylate synthase EC:1.5.1.3 2.1.1.45
K00846	0.178888	0.003125	0.012299	ketohexokinase EC:2.7.1.3
K01844	0.179066	0.003134	0.012306	beta-lysine 5,6-aminomutase EC:5.4.3.3
K02071	0.179064	0.003134	0.012306	D-methionine transport system ATP-binding protein
K07660	0.179044	0.003137	0.012313	two-component system, OmpR family, response regulator PhoP
K03976	0.178987	0.003147	0.012344	putative transcription regulator
K09384	0.178909	0.00316	0.012389	hypothetical protein; hypothetical protein
K03197	-0.17861	0.003173	0.012424	type IV secretion system protein VirB2
K03257	0.178613	0.003172	0.012424	translation initiation factor 4A
K03923	-0.17858	0.003178	0.012435	modulator of drug activity B
K07322	-0.17879	0.003181	0.01244	regulator of cell morphogenesis and NO signaling
K03618	-0.17878	0.003183	0.012442	hydrogenase-1 operon protein HyaF
K02868	0.178512	0.00319	0.012462	large subunit ribosomal protein L11e
K03497	0.178704	0.003196	0.012477	chromosome partitioning protein, ParB family
K02101	0.178414	0.003207	0.012514	arabinose operon protein AraL
K03685	0.178626	0.003209	0.012516	ribonuclease III EC:3.1.26.3
K06148	0.178598	0.003214	0.012528	ATP-binding cassette, subfamily C, bacterial
K02619	-0.17854	0.003223	0.012533	4-amino-4-deoxychorismate lyase EC:4.1.3.38
K02989	0.178327	0.003222	0.012533	small subunit ribosomal protein S5e
K05236	0.178333	0.003221	0.012533	coatomer protein complex, subunit alpha (xenin)
K10405	0.178323	0.003223	0.012533	kinesin family member C1
K14781	0.178314	0.003224	0.012533	ATP-dependent RNA helicase DHR2 EC:3.6.4.13
K04646	0.178279	0.00323	0.012549	clathrin heavy chain
K01557	-0.17825	0.003236	0.012549	acylpyruvate hydrolase EC:3.7.1.5
K01925	0.178479	0.003235	0.012549	UDP-N-acetylmuramoylalanine--D-glutamate ligase EC:6.3.2.9
K12830	0.178257	0.003234	0.012549	splicing factor 3B subunit 3
K00861	0.178203	0.003244	0.012573	riboflavin kinase EC:2.7.1.26
K02957	0.178166	0.00325	0.012591	small subunit ribosomal protein S15Ae
K01665	-0.17835	0.003257	0.012609	para-aminobenzoate synthetase component I EC:2.6.1.85
K01093	-0.17809	0.003263	0.012626	4-phytase / acid phosphatase EC:3.1.3.26 3.1.3.2
K05853	0.178078	0.003266	0.01263	Ca2+ transporting ATPase, sarcoplasmic/endoplasmic reticulum EC:3.6.3.8
K00761	0.178249	0.003275	0.012651	uracil phosphoribosyltransferase EC:2.4.2.9
K02938	0.17803	0.003275	0.012651	large subunit ribosomal protein L8e
K09580	0.177848	0.003307	0.012767	protein disulfide-isomerase A1 EC:5.3.4.1
K03601	0.177992	0.003321	0.012814	exodeoxyribonuclease VII large subunit EC:3.1.11.6
K03038	0.177747	0.003325	0.012823	26S proteasome regulatory subunit N8
K00817	-0.17793	0.003333	0.012845	histidinol-phosphate aminotransferase EC:2.6.1.9
K10848	0.177669	0.003339	0.012862	DNA excision repair protein ERCC-4 EC:3.1.-.-
K15146	0.177574	0.003356	0.012921	mediator of RNA polymerase II transcription subunit 4
K03594	-0.17775	0.003364	0.012938	bacterioferritin
K10977	0.177531	0.003364	0.012938	methanogen homocitrate synthase EC:2.3.3.14 2.3.3.-
K00563	0.177737	0.003367	0.01294	23S rRNA (guanine745-N1)-methyltransferase EC:2.1.1.187
K10746	0.177491	0.003371	0.012951	exonuclease 1 EC:3.1.-.-
K06931	0.177432	0.003382	0.012975	23S rRNA (adenine2503-C2)-methyltransferase EC:2.1.1.192; 23S rRNA (adenine2503-C2)-methyltransferase EC:2.1.1.192

K07289	-0.17766	0.00338	0.012975	AsmA protein
K12483	0.177425	0.003383	0.012975	EH domain-containing protein 1
K04066	0.177607	0.00339	0.012992	primosomal protein N' (replication factor Y) (superfamily II helicase) EC:3.6.4.-
K06920	-0.1776	0.003391	0.012992	queuosine biosynthesis protein QueC
K02880	0.177342	0.003399	0.013012	large subunit ribosomal protein L17e
K06202	-0.17754	0.003403	0.013023	CyaY protein
K02199	-0.17739	0.003429	0.013105	cytochrome c biogenesis protein CcmG, thiol_disulfide interchange protein DsbE
K09726	0.177187	0.003427	0.013105	hypothetical protein; hypothetical protein
K12876	0.177159	0.003432	0.013105	RNA-binding protein 8A
K14574	0.177166	0.003431	0.013105	ribosome maturation protein SDO1
K01918	-0.17731	0.003445	0.013147	pantoate--beta-alanine ligase EC:6.3.2.1
K03027	0.177067	0.003449	0.013155	DNA-directed RNA polymerases I and III subunit RPAC1
K11251	0.177048	0.003453	0.013162	histone H2A
K01443	-0.17721	0.003463	0.013177	N-acetylglucosamine-6-phosphate deacetylase EC:3.5.1.25
K03587	-0.17723	0.00346	0.013177	cell division protein FtsI (penicillin-binding protein 3) EC:2.4.1.129
K04499	0.177005	0.003461	0.013177	RuvB-like protein 1 (pontin 52)
K08059	0.176983	0.003465	0.013179	interferon, gamma-inducible protein 30
K03893	0.177107	0.003483	0.013238	arsenical pump membrane protein
K14778	0.176793	0.003501	0.0133	ATP-dependent RNA helicase DDX49/DBP8 EC:3.6.4.13
K07338	0.176776	0.003504	0.013305	hypothetical protein; hypothetical protein
K00926	0.176885	0.003524	0.013374	carbamate kinase EC:2.7.2.2
K00254	0.17664	0.00353	0.013388	dihydroorotate dehydrogenase EC:1.3.5.2
K07359	0.176601	0.003537	0.013401	calcium/calmodulin-dependent protein kinase kinase EC:2.7.11.17
K02873	0.176603	0.003537	0.013401	large subunit ribosomal protein L13e
K10708	-0.17679	0.003543	0.013405	fructoselysine 6-phosphate deglycase EC:3.5.-.-
K09487	0.176568	0.003543	0.013405	heat shock protein 90kDa beta
K14299	0.176565	0.003544	0.013405	nucleoporin SEH1
K02897	-0.17663	0.003572	0.013496	large subunit ribosomal protein L25
K14982	0.176635	0.003572	0.013496	two-component system, OmpR family, sensor histidine kinase CiaH EC:2.7.13.3
K13111	0.176346	0.003586	0.013541	WD40 repeat-containing protein SMU1
K07976	0.176323	0.00359	0.013543	Rab family, other
K12311	0.176324	0.00359	0.013543	lysosomal alpha-mannosidase EC:3.2.1.24
K05571	0.176525	0.003593	0.013545	multicomponent Na+_H+ antiporter subunit G
K00218	-0.1765	0.003597	0.013549	protochlorophyllide reductase EC:1.3.1.33
K03933	0.176499	0.003598	0.013549	chitin-binding protein
K09510	0.176234	0.003608	0.013579	DnaJ homolog subfamily B member 4
K06988	0.176127	0.003628	0.013605	aspartate dehydrogenase EC:1.4.1.21; aspartate dehydrogenase EC:1.4.1.21
K08723	0.17618	0.003618	0.013605	5'-nucleotidase EC:3.1.3.5
K08981	-0.17638	0.003621	0.013605	putative membrane protein; putative membrane protein
K14987	-0.17616	0.003622	0.013605	two-component system, LuxR family, response regulator FixJ
K03283	0.176157	0.003623	0.013605	heat shock 70kDa protein 1/8
K14538	0.176142	0.003625	0.013605	nuclear GTP-binding protein
K15113	0.176136	0.003627	0.013605	solute carrier family 25 (mitochondrial iron transporter), member 28/37
K00790	0.176191	0.003657	0.013706	UDP-N-acetylglucosamine 1-carboxyvinyltransferase EC:2.5.1.7

K03301	0.175966	0.00366	0.013708	ATP_ADP antiporter, AAA family
K02773	-0.17613	0.00367	0.013739	PTS system, galactitol-specific IIA component EC:2.7.1.69
K14005	0.175826	0.003687	0.013796	protein transport protein SEC31
K14774	0.175797	0.003693	0.01381	U3 small nucleolar RNA-associated protein 25
K00188	0.175741	0.003704	0.013844	2-oxoisovalerate ferredoxin oxidoreductase, delta subunit EC:1.2.7.7
K02567	-0.17589	0.003718	0.013886	periplasmic nitrate reductase NapA EC:1.7.99.4
K12812	0.1756	0.003732	0.013918	ATP-dependent RNA helicase UAP56/SUB2 EC:3.6.4.13
K08734	0.175591	0.003734	0.013918	DNA mismatch repair protein MLH1
K11540	0.175592	0.003734	0.013918	carbamoyl-phosphate synthase / aspartate carbamoyltransferase / dihydroorotase EC:6.3.5.5 2.1.3.2 3.5.2.3
K14772	0.175603	0.003732	0.013918	U3 small nucleolar RNA-associated protein 20
K02734	0.175564	0.00374	0.013931	20S proteasome subunit beta 4 EC:3.4.25.1
K11618	0.175748	0.003745	0.013943	two-component system, NarL family, response regulator LiaR
K14758	0.175478	0.003757	0.01398	casein kinase I homolog HRR25 EC:2.7.11.1
K03953	0.175398	0.003773	0.014032	NADH dehydrogenase (ubiquinone) 1 alpha subcomplex 9 EC:1.6.5.3 1.6.99.3
K09962	0.175342	0.003784	0.014067	hypothetical protein; hypothetical protein
K01230	0.175246	0.003804	0.01412	mannosyl-oligosaccharide alpha-1,2-mannosidase EC:3.2.1.113
K02940	0.175248	0.003803	0.01412	large subunit ribosomal protein L9e
K12845	0.175241	0.003805	0.01412	U4/U6 small nuclear ribonucleoprotein SNU13
K01318	0.175188	0.003815	0.014148	glutamyl endopeptidase EC:3.4.21.19
K10752	0.175184	0.003816	0.014148	histone-binding protein RBBP4
K01823	0.175366	0.003822	0.01416	isopentenyl-diphosphate delta-isomerase EC:5.3.3.2
K11824	0.175035	0.003847	0.014246	AP-2 complex subunit alpha
K03741	-0.17522	0.003851	0.014253	arsenate reductase EC:1.20.4.1
K14808	0.175001	0.003854	0.014256	ATP-dependent RNA helicase DDX54/DBP10 EC:3.6.4.13
K11654	0.174954	0.003863	0.014277	SWI/SNF-related matrix-associated actin-dependent regulator of chromatin subfamily A member 5 EC:3.6.4.-
K07889	0.174956	0.003863	0.014277	Ras-related protein Rab-5C
K14558	0.17491	0.003872	0.014303	periodic tryptophan protein 2
K11322	0.174872	0.00388	0.014324	enhancer of polycomb-like protein
K07251	0.174973	0.003902	0.014397	thiamine kinase EC:2.7.1.89
K06062	0.174699	0.003916	0.014441	histone acetyltransferase EC:2.3.1.48
K03094	0.174663	0.003924	0.014461	S-phase kinase-associated protein 1
K13754	0.174627	0.003931	0.014481	solute carrier family 24 (sodium/potassium/calcium exchanger), member 6
K08272	0.174513	0.003955	0.014561	calcium binding protein 39
K10756	0.174467	0.003965	0.014589	replication factor C subunit 3/5
K03235	0.17445	0.003969	0.014595	elongation factor 3
K12453	-0.17443	0.003974	0.014606	CDP-paratose synthetase EC:1.-.-.-
K01958	-0.17454	0.003993	0.014662	pyruvate carboxylase EC:6.4.1.1
K13786	0.174333	0.003993	0.014662	cob(II)yrinic acid a,c-diamide reductase EC:1.16.8.1
K01117	0.174297	0.004001	0.014683	sphingomyelin phosphodiesterase EC:3.1.4.12
K01937	0.174381	0.004026	0.014768	CTP synthase EC:6.3.4.2
K06997	0.174343	0.004035	0.014791	phospholipase/carboxylesterase; phospholipase/carboxylesterase
K14554	0.174128	0.004037	0.014792	U3 small nucleolar RNA-associated protein 21
K12863	0.174107	0.004041	0.0148	protein CWC15
K01998	0.174255	0.004053	0.014836	branched-chain amino acid transport system permease protein

K13431	0.174012	0.004062	0.014859	signal recognition particle receptor subunit alpha
K02998	0.173917	0.004082	0.014926	small subunit ribosomal protein SAe
K08874	0.173886	0.004089	0.014943	transformation/transcription domain-associated protein
K08322	-0.17405	0.004097	0.014956	starvation sensing protein RspB EC:1.1.1.-
K14827	0.173859	0.004095	0.014956	pre-rRNA-processing protein IPI1
K11253	0.173835	0.0041	0.01496	histone H3
K03178	0.173817	0.004104	0.014966	ubiquitin-activating enzyme E1 EC:6.3.2.19
K05384	0.173765	0.004115	0.014999	bilin biosynthesis protein
K13642	0.173755	0.004118	0.015	CRP/FNR family transcriptional regulator, transcriptional activator FtrB
K06674	0.173632	0.004145	0.01509	structural maintenance of chromosome 2
K02041	0.17382	0.004148	0.015092	phosphonate transport system ATP-binding protein EC:3.6.3.28
K07053	0.173768	0.004159	0.015126	tRNA wybutosine-synthesizing protein 2 EC:2.1.1.-; tRNA wybutosine-synthesizing protein 2 EC:2.1.1.-
K09499	0.173555	0.004161	0.015127	T-complex protein 1 subunit eta
K01999	0.17362	0.004191	0.015228	branched-chain amino acid transport system substrate-binding protein
K12855	0.173265	0.004226	0.015345	pre-mRNA-processing factor 6
K11254	0.173237	0.004232	0.01536	histone H4
K03002	0.173191	0.004242	0.015389	DNA-directed RNA polymerase I subunit RPA2 EC:2.7.7.6
K00332	0.173143	0.004253	0.015421	NADH-quinone oxidoreductase subunit C EC:1.6.5.3
K02025	0.173252	0.004273	0.015463	multiple sugar transport system permease protein
K03200	-0.17304	0.004276	0.015463	type IV secretion system protein VirB5
K05570	0.173031	0.004279	0.015463	multicomponent Na+_H+ antiporter subunit F
K11494	0.173251	0.004274	0.015463	RCC1 and BTB domain-containing protein
K12344	-0.17324	0.004275	0.015463	3-oxo-5-alpha-steroid 4-dehydrogenase 2 EC:1.3.99.5
K11434	0.173039	0.004277	0.015463	protein arginine N-methyltransferase 1 EC:2.1.1.-
K05587	0.172991	0.004288	0.015488	bidirectional NiFe hydrogenase diaphorase subunit EC:1.6.5.3
K03490	0.173157	0.004295	0.015505	AraC family transcriptional regulator, cel operon repressor
K01263	-0.17284	0.004323	0.015588	cytosol alanyl aminopeptidase EC:3.4.11.14
K06515	0.17283	0.004324	0.015588	solute carrier family 44 (choline transporter-like protein), member 1
K08489	0.172834	0.004323	0.015588	syntaxin 16
K03021	0.172804	0.00433	0.0156	DNA-directed RNA polymerase III subunit RPC2 EC:2.7.7.6
K02021	0.172933	0.004346	0.015648	putative ABC transport system ATP-binding protein
K06966	-0.17289	0.004355	0.015672	tRNA (adenine22-N1)-methyltransferase EC:2.1.1.217; tRNA (adenine22-N1)-methyltransferase EC:2.1.1.217
K00898	0.172673	0.00436	0.015684	pyruvate dehydrogenase kinase EC:2.7.11.2
K06067	0.172486	0.004403	0.015831	histone deacetylase 1/2 EC:3.5.1.98
K04782	0.172475	0.004406	0.015832	isochorismate pyruvate lyase EC:4.2.99.21
K01537	0.17263	0.004415	0.015857	Ca2+-transporting ATPase EC:3.6.3.8
K01996	0.172555	0.004433	0.015912	branched-chain amino acid transport system ATP-binding protein
K02493	0.172497	0.004446	0.015952	release factor glutamine methyltransferase EC:2.1.1.-
K05349	-0.17248	0.00445	0.015957	beta-glucosidase EC:3.2.1.21
K00230	-0.17238	0.004473	0.016023	menaquinone-dependent protoporphyrinogen oxidase EC:1.3.5.3
K11853	0.172189	0.004473	0.016023	ubiquitin carboxyl-terminal hydrolase 34 EC:3.1.2.15
K05850	0.17216	0.004479	0.016034	Ca2+ transporting ATPase, plasma membrane EC:3.6.3.8
K10949	0.172155	0.004481	0.016034	ER lumen protein retaining receptor
K00249	0.172096	0.004494	0.016067	acyl-CoA dehydrogenase EC:1.3.99.3
K00006	0.172102	0.004493	0.016067	glycerol-3-phosphate dehydrogenase (NAD+) EC:1.1.1.8

K03573	0.17215	0.004527	0.016177	DNA mismatch repair protein MutH
K08831	0.17192	0.004536	0.016199	serine/threonine-protein kinase SRPK2 EC:2.7.11.1
K11131	0.171885	0.004545	0.016221	H/ACA ribonucleoprotein complex subunit 4 EC:5.4.99.-
K14802	0.171774	0.004571	0.016308	phospholipid-transporting ATPase EC:3.6.3.1
K14550	0.171719	0.004584	0.016346	U3 small nucleolar RNA-associated protein 10
K14455	0.171695	0.00459	0.016359	aspartate aminotransferase, mitchondrial EC:2.6.1.1
K12879	0.171672	0.004596	0.016369	THO complex subunit 2
K03874	0.17165	0.004601	0.01638	other hect domain ubiquitin protein ligase E3 EC:6.3.2.19
K07277	-0.17182	0.004607	0.016391	outer membrane protein
K03842	0.171599	0.004613	0.016407	beta-1,4-mannosyltransferase EC:2.4.1.142
K01194	0.171702	0.004635	0.016458	alpha,alpha-trehalase EC:3.2.1.28
K07462	0.171713	0.004632	0.016458	single-stranded-DNA-specific exonuclease EC:3.1.-.-
K11104	0.171701	0.004635	0.016458	melibiose permease
K00591	0.171441	0.004652	0.016492	hexaprenyldihydroxybenzoate methyltransferase EC:2.1.1.114
K01820	-0.17164	0.00465	0.016492	4-oxalocrotonate tautomerase EC:5.3.2.-
K12618	0.17145	0.00465	0.016492	5'-3' exoribonuclease 1 EC:3.1.13.-
K00208	-0.1716	0.004659	0.016502	enoyl-acyl-carrier protein reductase I EC:1.3.1.9
K11662	0.171416	0.004658	0.016502	actin-related protein 6
K04083	0.171576	0.004665	0.016515	molecular chaperone Hsp33
K00666	-0.17155	0.004671	0.016517	fatty-acyl-CoA synthase EC:6.2.1.-; fatty-acyl-CoA synthase EC:6.2.1.-
K04095	0.171562	0.004669	0.016517	cell filamentation protein
K09005	0.171542	0.004674	0.016519	hypothetical protein; hypothetical protein
K12869	0.17128	0.004691	0.016573	crooked neck
K10413	0.171204	0.00471	0.01663	dynein heavy chain 1, cytosolic
K03275	0.171353	0.00472	0.016648	UDP-glucose_(glucosyl)LPS alpha-1,3-glucosyltransferase EC:2.4.1.-
K07642	0.171354	0.00472	0.016648	two-component system, OmpR family, sensor histidine kinase BaeS EC:2.7.13.3
K11273	0.171126	0.004729	0.016673	chromosome transmission fidelity protein 1 EC:3.6.4.13
K12520	-0.17111	0.004733	0.016678	minor pilin subunit PapE
K11121	0.171072	0.004743	0.016696	NAD-dependent histone deacetylase SIR2 EC:3.5.1.-
K14767	0.17107	0.004743	0.016696	U3 small nucleolar RNA-associated protein 3
K13025	0.171004	0.004759	0.016745	ATP-dependent RNA helicase EC:3.6.4.13
K00963	0.171058	0.004793	0.016846	UTP--glucose-1-phosphate uridylyltransferase EC:2.7.7.9
K14833	0.17087	0.004793	0.016846	nucleolar complex protein 2
K01788	-0.17085	0.004797	0.016853	N-acylglucosamine-6-phosphate 2-epimerase EC:5.1.3.9
K13892	-0.17101	0.004804	0.016868	glutathione transport system ATP-binding protein
K00662	0.170967	0.004815	0.016899	aminoglycoside N3'-acetyltransferase EC:2.3.1.81
K02212	0.170761	0.00482	0.016907	minichromosome maintenance protein 4 (cell division control protein 54)
K05563	0.170741	0.004825	0.016916	multicomponent K+_H+ antiporter subunit F
K04029	-0.17068	0.00484	0.01696	ethanolamine utilization protein EutP
K14565	0.170618	0.004856	0.017008	nucleolar protein 58
K11380	0.170602	0.00486	0.017013	NuA3 HAT complex component NTO1
K08254	0.170562	0.00487	0.01704	glucan endo-1,3-alpha-glucosidase EC:3.2.1.59
K08864	0.170551	0.004873	0.017041	tousled-like kinase EC:2.7.11.1
K05569	0.170708	0.004881	0.017059	multicomponent Na+_H+ antiporter subunit E
K12864	0.170471	0.004894	0.017096	beta-catenin-like protein 1
K01585	-0.17064	0.004897	0.017098	arginine decarboxylase EC:4.1.1.19
K12736	0.170428	0.004905	0.017117	peptidylprolyl isomerase domain and WD repeat-containing protein 1 EC:5.2.1.8

K05931	0.170251	0.00495	0.017266	histone-arginine methyltransferase CARM1 EC:2.1.1.125
K03883	0.170237	0.004953	0.01727	NADH-ubiquinone oxidoreductase chain 5 EC:1.6.5.3
K07467	-0.17035	0.004973	0.017328	phage replication initiation protein
K07263	-0.17022	0.005006	0.017434	zinc protease EC:3.4.24.-
K06324	0.169977	0.005021	0.017478	spore coat protein A
K00997	0.170132	0.005029	0.017483	holo-acyl-carrier protein synthase EC:2.7.8.7
K03673	-0.17012	0.005032	0.017483	thiol_disulfide interchange protein DsbA
K08264	0.169933	0.005032	0.017483	heterodisulfide reductase subunit D EC:1.8.98.1
K14983	0.16996	0.005025	0.017483	two-component system, OmpR family, response regulator CiaR
K14006	0.169906	0.00504	0.017499	protein transport protein SEC23
K06218	0.170055	0.005049	0.017522	RelE protein
K09953	0.169745	0.005082	0.017628	lipid A 3-O-deacylase
K00955	0.169915	0.005085	0.017631	bifunctional enzyme CysN/CysC EC:2.7.7.4 2.7.1.25
K00962	0.169822	0.00511	0.017698	polyribonucleotide nucleotidyltransferase EC:2.7.7.8
K06641	0.169646	0.005108	0.017698	serine/threonine-protein kinase Chk2 EC:2.7.11.1
K07151	0.16958	0.005126	0.017744	dolichyl-diphosphooligosaccharide--protein glycosyltransferase EC:2.4.1.119
K12191	0.169568	0.005129	0.017746	charged multivesicular body protein 2A
K00888	0.169527	0.00514	0.017775	phosphatidylinositol 4-kinase EC:2.7.1.67
K12391	0.169484	0.005151	0.017806	AP-1 complex subunit gamma-1
K11584	0.169459	0.005158	0.01782	protein phosphatase 2 (formerly 2A), regulatory subunit B'
K04041	-0.16959	0.00517	0.017854	fructose-1,6-bisphosphatase III EC:3.1.3.11
K03644	-0.16954	0.005185	0.017883	lipoic acid synthetase EC:2.8.1.8
K11373	0.16937	0.005182	0.017883	elongator complex protein 1
K12811	0.169352	0.005187	0.017883	ATP-dependent RNA helicase DDX46/PRP5 EC:3.6.4.13
K12940	-0.16946	0.005206	0.017934	aminobenzoyl-glutamate utilization protein A
K14292	0.169288	0.005204	0.017934	trimethylguanosine synthase EC:2.1.1.-
K03389	0.169409	0.00522	0.017971	heterodisulfide reductase subunit B EC:1.8.98.1
K05606	-0.16937	0.00523	0.017997	methylmalonyl-CoA/ethylmalonyl-CoA epimerase EC:5.1.99.1
K10415	0.169124	0.005248	0.01805	dynein intermediate chain, cytosolic
K05567	0.169289	0.005252	0.018055	multicomponent Na+_H+ antiporter subunit C
K01807	-0.16925	0.005264	0.018078	ribose 5-phosphate isomerase A EC:5.3.1.6
K14801	0.169074	0.005262	0.018078	pre-rRNA-processing protein TSR4
K00983	-0.16923	0.005268	0.018084	N-acylneuraminate cytidylyltransferase EC:2.7.7.43
K02985	0.168926	0.005302	0.018191	small subunit ribosomal protein S3e
K07697	0.168883	0.005314	0.018222	two-component system, sporulation sensor kinase B EC:2.7.13.3
K14560	0.168837	0.005326	0.018257	U3 small nucleolar ribonucleoprotein protein IMP3
K11688	-0.16898	0.005335	0.018277	C4-dicarboxylate-binding protein DctP
K14780	0.168779	0.005343	0.018294	ATP-dependent RNA helicase DHX37/DHR1 EC:3.6.4.13
K01759	-0.16893	0.00535	0.01831	lactoylglutathione lyase EC:4.4.1.5
K03043	0.168673	0.005372	0.018376	DNA-directed RNA polymerase subunit beta EC:2.7.7.6
K14950	0.168636	0.005382	0.018402	cation-transporting ATPase 13A1 EC:3.6.3.-
K00328	0.168583	0.005397	0.018433	inositol-polyphosphate multikinase EC:2.7.1.151
K03254	0.168583	0.005397	0.018433	translation initiation factor 3 subunit A
K00996	-0.16848	0.005425	0.018493	undecaprenyl-phosphate galactose phosphotransferase EC:2.7.8.6
K07441	0.168676	0.005421	0.018493	beta-1,4-N-acetylglucosaminyltransferase EC:2.4.1.141
K00026	0.168481	0.005425	0.018493	malate dehydrogenase EC:1.1.1.37
K04482	0.168473	0.005428	0.018493	DNA repair protein RAD51

K13172	0.168497	0.005421	0.018493	serine/arginine repetitive matrix protein 2
K03097	0.168426	0.005441	0.018528	casein kinase II subunit alpha EC:2.7.11.1
K13102	0.168366	0.005457	0.018576	DNA/RNA-binding protein KIN17
K00338	-0.16853	0.00546	0.018577	NADH-quinone oxidoreductase subunit I EC:1.6.5.3
K03198	-0.16825	0.00549	0.01866	type IV secretion system protein VirB3
K03456	0.168251	0.00549	0.01866	protein phosphatase 2 (formerly 2A), regulatory subunit A
K03279	0.168407	0.005496	0.01867	UDP-glucose_(galactosyl)LPS alpha-1,2-glucosyltransferase EC:2.4.1.58
K01531	0.168376	0.005504	0.01869	Mg2+-importing ATPase EC:3.6.3.2
K12818	0.168128	0.005525	0.018749	ATP-dependent RNA helicase DHX8/PRP22 EC:3.6.4.13
K11267	0.16812	0.005527	0.018749	sister chromatid cohesion protein PDS5
K13797	0.168094	0.005535	0.018756	DNA-directed RNA polymerase subunit beta-beta' EC:2.7.7.6
K07374	0.168096	0.005534	0.018756	tubulin alpha
K10213	-0.16823	0.005544	0.01878	ribosylpyrimidine nucleosidase EC:3.2.2.8
K02999	0.168028	0.005553	0.018801	DNA-directed RNA polymerase I subunit RPA1 EC:2.7.7.6
K08092	-0.16812	0.005578	0.018874	3-dehydro-L-gulonate 2-dehydrogenase EC:1.1.1.130
K14414	-0.16808	0.005587	0.018894	transcriptional regulatory protein RtcR
K12670	0.167903	0.005589	0.018894	oligosaccharyltransferase complex subunit beta
K11332	0.16786	0.005602	0.018927	two-component system, OmpR family, response regulator NblR
K11184	0.168001	0.005611	0.01895	catabolite repression HPr-like protein
K14209	0.167808	0.005617	0.018959	solute carrier family 36 (proton-coupled amino acid transporter)
K14403	0.167783	0.005624	0.018974	cleavage and polyadenylation specificity factor subunit 3 EC:3.1.27.-
K06940	0.167877	0.005647	0.019043	23S rRNA (adenine2503-C2)-methyltransferase EC:2.1.1.192; 23S rRNA (adenine2503-C2)-methyltransferase EC:2.1.1.192
K01072	0.167681	0.005653	0.019046	ubiquitin thiolesterase EC:3.1.2.15
K11238	0.167685	0.005652	0.019046	cytokinesis protein
K03740	0.167813	0.005665	0.019077	D-alanine transfer protein
K07664	-0.16777	0.005679	0.019113	two-component system, OmpR family, response regulator BaeR
K03063	0.167552	0.005691	0.019144	26S proteasome regulatory subunit T3
K00010	-0.16771	0.005696	0.019153	myo-inositol 2-dehydrogenase EC:1.1.1.18
K07759	0.167523	0.005699	0.019154	poly(ADP-ribose) glycohydrolase EC:3.2.1.143
K12447	0.167445	0.005722	0.019222	UDP-sugar pyrophosphorylase EC:2.7.7.64
K15013	0.167386	0.00574	0.01927	long-chain-fatty-acid--CoA ligase ACSBG EC:6.2.1.3
K03483	-0.16741	0.005784	0.019335	mannitol operon transcriptional antiterminator
K05337	0.167442	0.005774	0.019335	ferredoxin
K06190	-0.16744	0.005775	0.019335	intracellular septation protein
K06446	0.167408	0.005784	0.019335	acyl-CoA dehydrogenase EC:1.3.99.-
K06607	0.167254	0.005778	0.019335	myo-inositol catabolism protein IolS EC:1.1.1.-
K08309	0.167424	0.005779	0.019335	soluble lytic murein transglycosylase EC:3.2.1.-
K00582	0.167238	0.005783	0.019335	tetrahydromethanopterin S-methyltransferase subunit F EC:2.1.1.86
K02934	0.167273	0.005773	0.019335	large subunit ribosomal protein L6e
K12402	0.167253	0.005779	0.019335	AP-4 complex subunit mu-1
K00600	0.167389	0.005789	0.019335	glycine hydroxymethyltransferase EC:2.1.2.1
K01687	-0.16739	0.00579	0.019335	dihydroxy-acid dehydratase EC:4.2.1.9
K11531	-0.16737	0.005796	0.019347	lsr operon transcriptional repressor
K00692	0.167151	0.005809	0.019382	levansucrase EC:2.4.1.10
K14290	0.167096	0.005825	0.019426	exportin-1

K06184	0.167008	0.005852	0.019505	ATP-binding cassette, subfamily F, member 1
K09691	0.167151	0.00586	0.019519	lipopolysaccharide transport system ATP-binding protein
K08860	0.166974	0.005862	0.019519	eukaryotic translation initiation factor 2-alpha kinase EC:2.7.11.1
K11252	0.166965	0.005865	0.019519	histone H2B
K06198	0.166955	0.005868	0.01952	competence protein CoiA
K02026	0.167102	0.005875	0.019526	multiple sugar transport system permease protein
K09489	0.16693	0.005875	0.019526	heat shock 70kDa protein 4
K14842	0.166885	0.005889	0.019562	ribosome biogenesis protein NSA2
K02730	0.166874	0.005892	0.019564	20S proteasome subunit alpha 1 EC:3.4.25.1
K10598	0.166865	0.005895	0.019564	peptidyl-prolyl cis-trans isomerase-like 2 EC:5.2.1.8
K13511	0.166824	0.005907	0.019595	monolysocardiolipin acyltransferase EC:2.3.1.-
K02737	0.166811	0.005911	0.019599	20S proteasome subunit beta 5 EC:3.4.25.1
K03515	0.166768	0.005924	0.019632	DNA repair protein REV1 EC:2.7.7.-
K14768	0.166752	0.005929	0.019639	U3 small nucleolar RNA-associated protein 7
K00432	-0.16686	0.005948	0.019692	glutathione peroxidase EC:1.11.1.9
K11296	0.166676	0.005952	0.019695	high mobility group protein B3
K12256	0.166668	0.005954	0.019695	putrescine aminotransferase EC:2.6.1.-
K11093	0.166635	0.005964	0.019719	U1 small nuclear ribonucleoprotein 70kDa
K03584	0.166782	0.005971	0.019729	DNA repair protein RecO (recombination protein O)
K03037	0.166606	0.005973	0.019729	26S proteasome regulatory subunit N7
K11474	-0.16657	0.005983	0.019742	GntR family transcriptional regulator, glc operon transcriptional activator
K12393	0.166576	0.005982	0.019742	AP-1 complex subunit mu
K14404	0.166421	0.00603	0.019888	cleavage and polyadenylation specificity factor subunit 4
K07025	-0.16656	0.006038	0.019896	putative hydrolase of the HAD superfamily; putative hydrolase of the HAD superfamily
K14861	0.166397	0.006037	0.019896	nucleolar pre-ribosomal-associated protein 1
K02901	0.166376	0.006044	0.019904	large subunit ribosomal protein L27e
K13148	0.166278	0.006074	0.019995	integrator complex subunit 11 EC:3.1.27.-
K06909	0.166427	0.00608	0.020005	alpha-ketoglutarate-dependent 2,4-dichlorophenoxyacetate dioxygenase EC:1.14.11.-; alpha-ketoglutarate-dependent 2,4-dichlorophenoxyacetate dioxygenase EC:1.14.11.-
K11374	0.166229	0.006089	0.020026	elongator complex protein 2
K03619	-0.16621	0.006095	0.020034	hydrogenase-1 operon protein HyaE
K01810	0.166328	0.006111	0.020077	glucose-6-phosphate isomerase EC:5.3.1.9
K02008	0.166284	0.006124	0.020112	cobalt/nickel transport system permease protein
K10770	0.166068	0.006139	0.020152	alkylated DNA repair protein alkB homolog 8 EC:1.14.11.- 2.1.1.229
K01654	-0.16621	0.006146	0.020163	N-acetylneuraminate synthase EC:2.5.1.56
K02669	0.166039	0.006148	0.020163	twitching motility protein PilT
K12554	0.16616	0.006163	0.020193	alanine adding enzyme EC:2.3.2.-
K00698	0.165991	0.006164	0.020193	chitin synthase EC:2.4.1.16
K13181	0.16597	0.00617	0.020205	ATP-dependent RNA helicase DDX27 EC:3.6.4.13
K06036	-0.16604	0.0062	0.020283	variant-surface-glycoprotein phospholipase C EC:4.6.1.14
K12883	0.165879	0.006199	0.020283	nuclear cap-binding protein subunit 2
K00713	0.165801	0.006223	0.020351	UDP-glucose_(glucosyl)LPS alpha-1,2-glucosyltransferase EC:2.4.1.-
K03233	0.165787	0.006228	0.020355	elongation factor 1-gamma
K03427	0.165937	0.006233	0.020362	type I restriction enzyme M protein EC:2.1.1.72
K10742	-0.16587	0.006255	0.020409	DNA replication ATP-dependent helicase Dna2 EC:3.6.4.12
K14127	0.165698	0.006256	0.020409	F420-non-reducing hydrogenase iron-sulfur subunit D EC:1.12.99.-

K13100	0.165705	0.006254	0.020409	pre-mRNA-splicing factor CWC22
K05343	0.165804	0.006275	0.020461	maltose alpha-D-glucosyltransferase EC:5.4.99.16
K03658	0.165778	0.006283	0.020478	DNA helicase IV EC:3.6.4.12
K15105	0.165599	0.006288	0.020483	solute carrier family 25 (mitochondrial aspartate/glutamate transporter), member 12/13
K01044	0.165709	0.006305	0.020521	carboxylesterase 1 EC:3.1.1.1
K03631	0.165713	0.006304	0.020521	DNA repair protein RecN (Recombination protein N)
K02146	0.165508	0.006317	0.020549	V-type H+-transporting ATPase subunit AC39 EC:3.6.3.14
K00844	-0.16557	0.006348	0.020641	hexokinase EC:2.7.1.1
K11166	0.16538	0.006358	0.020663	dehydrogenase/reductase SDR family member 7B EC:1.1.-.-
K01277	-0.16553	0.006363	0.020671	dipeptidyl-peptidase III EC:3.4.14.4
K04392	0.165352	0.006367	0.020673	Ras-related C3 botulinum toxin substrate 1
K07776	0.165436	0.006393	0.020743	two-component system, OmpR family, response regulator RegX3
K09793	-0.16543	0.006395	0.020743	hypothetical protein; hypothetical protein
K07673	-0.16538	0.006409	0.020781	two-component system, NarL family, nitrate/nitrite sensor histidine kinase NarX EC:2.7.13.3
K05516	-0.16535	0.00642	0.020806	curved DNA-binding protein
K11541	0.165162	0.006429	0.020824	carbamoyl-phosphate synthase / aspartate carbamoyltransferase EC:6.3.5.5 2.1.3.2
K10754	0.165125	0.006441	0.020853	replication factor C subunit 1
K10703	0.165066	0.00646	0.020896	3-hydroxy acyl-CoA dehydratase EC:4.2.1.-
K14569	0.165072	0.006458	0.020896	ribosome biogenesis protein BMS1
K14335	0.165005	0.00648	0.020949	alpha-1,6-mannosyltransferase EC:2.4.1.-
K05956	0.164997	0.006482	0.020949	geranylgeranyl transferase type-2 subunit beta EC:2.5.1.60
K00876	0.165127	0.006493	0.020974	uridine kinase EC:2.7.1.48
K10365	0.164943	0.0065	0.020988	capping protein (actin filament) muscle Z-line, beta
K03087	0.165079	0.006509	0.020994	RNA polymerase nonessential primary-like sigma factor
K07874	0.164915	0.00651	0.020994	Ras-related protein Rab-1A
K14832	0.16491	0.006511	0.020994	ribosome biogenesis protein MAK21
K05991	0.16487	0.006524	0.021015	bacterial leucyl aminopeptidase EC:3.4.11.10
K00902	0.164862	0.006527	0.021015	dolichol kinase EC:2.7.1.108
K10738	0.164877	0.006522	0.021015	minichromosome maintenance protein 9
K12243	0.164981	0.006541	0.02105	AraC family transcriptional regulator, transcriptional activator of the genes for pyochelin and ferripyochelin receptors
K06636	0.164732	0.00657	0.021133	structural maintenance of chromosome 1
K05801	-0.16487	0.006577	0.021146	DnaJ like chaperone protein
K04371	0.164614	0.006609	0.021241	extracellular signal-regulated kinase 1/2 EC:2.7.11.24
K03568	0.164758	0.006615	0.021244	TldD protein
K11176	0.164753	0.006616	0.021244	IMP cyclohydrolase EC:3.5.4.10
K08867	0.164566	0.006625	0.021262	WNK lysine deficient protein kinase EC:2.7.11.1
K01157	0.164638	0.006655	0.021346	deoxyribonuclease II EC:3.1.22.1
K11712	-0.16453	0.006691	0.021453	two-component system, LuxR family, response regulator DctR
K02738	0.164323	0.006707	0.021494	20S proteasome subunit beta 1 EC:3.4.25.1
K13480	-0.16429	0.006717	0.021496	xanthine dehydrogenase iron-sulfur-binding subunit
K09496	0.164298	0.006716	0.021496	T-complex protein 1 subunit delta
K10592	0.164303	0.006714	0.021496	E3 ubiquitin-protein ligase HUWE1 EC:6.3.2.19
K00484	0.164315	0.006764	0.021636	4-hydroxyphenylacetate-3-monooxygenase small chain EC:1.14.14.9
K14806	0.164114	0.006778	0.021672	ATP-dependent RNA helicase DDX31/DBP7 EC:3.6.4.13
K03611	-0.1641	0.006784	0.021681	disulfide bond formation protein DsbB
K07221	-0.16415	0.006821	0.021769	phosphate-selective porin OprO and OprP

K03251	0.163986	0.006822	0.021769	translation initiation factor 3 subunit D
K11088	0.164001	0.006817	0.021769	small nuclear ribonucleoprotein D3
K12814	0.163979	0.006824	0.021769	pre-mRNA-splicing factor ATP-dependent RNA helicase-like protein PRP2 EC:3.6.4.13
K01205	-0.16409	0.00684	0.021785	alpha-N-acetylglucosaminidase EC:3.2.1.50
K02194	-0.16409	0.006841	0.021785	heme exporter protein B
K06408	0.164076	0.006845	0.021785	stage V sporulation protein AF
K13378	-0.16411	0.006833	0.021785	NADH-quinone oxidoreductase subunit C/D EC:1.6.5.3
K12619	0.163927	0.006842	0.021785	5'-3' exoribonuclease 2 EC:3.1.13.-
K03592	0.164067	0.006848	0.021786	PmbA protein
K00389	-0.1639	0.006852	0.021788	putative membrane protein
K05898	-0.16388	0.00686	0.021792	3-oxosteroid 1-dehydrogenase EC:1.3.99.4
K03010	0.16388	0.006858	0.021792	DNA-directed RNA polymerase II subunit RPB2 EC:2.7.7.6
K03118	-0.16396	0.006883	0.021857	sec-independent protein translocase protein TatC
K02154	0.163789	0.00689	0.021867	V-type H+-transporting ATPase subunit I EC:3.6.3.14
K06697	0.163735	0.006909	0.021917	proteasome activator subunit 2 (PA28 beta)
K09971	-0.16387	0.006916	0.02193	general L-amino acid transport system permease protein
K01633	-0.16386	0.006919	0.02193	dihydroneopterin aldolase EC:4.1.2.25
K07407	-0.16385	0.006923	0.021932	alpha-galactosidase EC:3.2.1.22
K00823	0.163815	0.006935	0.021944	4-aminobutyrate aminotransferase EC:2.6.1.19
K01676	-0.16382	0.006934	0.021944	fumarate hydratase, class I EC:4.2.1.2
K15085	0.163655	0.006936	0.021944	solute carrier family 25, member 42
K14429	0.163545	0.006975	0.022046	solute carrier family 12 (potassium/chloride transporters), member 9
K05864	0.163554	0.006972	0.022046	peptidyl-prolyl isomerase D EC:5.2.1.8
K09688	-0.16344	0.007011	0.022149	capsular polysaccharide transport system permease protein
K00788	-0.16358	0.007018	0.022161	thiamine-phosphate pyrophosphorylase EC:2.5.1.3
K01280	0.163314	0.007056	0.022256	tripeptidyl-peptidase II EC:3.4.14.10
K07692	0.163454	0.007062	0.022256	two-component system, NarL family, response regulator DegU
K11837	0.163292	0.007064	0.022256	ubiquitin carboxyl-terminal hydrolase 6/32 EC:3.1.2.15
K14835	0.163293	0.007064	0.022256	ribosomal RNA methyltransferase Nop2 EC:2.1.1.-
K15104	0.163315	0.007056	0.022256	solute carrier family 25 (mitochondrial oxoglutarate transporter), member 11
K03781	-0.16341	0.007077	0.022288	catalase EC:1.11.1.6
K08576	0.163216	0.007091	0.022321	calpain-7 EC:3.4.22.-
K02729	0.163145	0.007116	0.022391	20S proteasome subunit alpha 5 EC:3.4.25.1
K09420	0.163083	0.007139	0.02245	myb proto-oncogene protein
K11835	0.162984	0.007174	0.022552	ubiquitin carboxyl-terminal hydrolase 4/11/15 EC:3.1.2.15
K07649	0.163055	0.007204	0.022634	two-component system, OmpR family, sensor histidine kinase TctE EC:2.7.13.3
K15119	0.162841	0.007226	0.022692	solute carrier family 25, member 39/40
K09952	0.162887	0.007264	0.022803	hypothetical protein; hypothetical protein
K00241	-0.16287	0.007271	0.022815	succinate dehydrogenase cytochrome b556 subunit
K01951	-0.16285	0.007278	0.022824	GMP synthase (glutamine-hydrolysing) EC:6.3.5.2
K08884	0.162842	0.007281	0.022824	serine/threonine protein kinase, bacterial EC:2.7.11.1; serine/threonine protein kinase, bacterial EC:2.7.11.1
K01299	0.162813	0.007291	0.022846	carboxypeptidase Taq EC:3.4.17.19
K08320	0.16265	0.007295	0.022848	CTP pyrophosphohydrolase EC:3.6.1.-
K08604	0.162615	0.007308	0.022878	vibriolysin EC:3.4.24.25
K00143	0.162557	0.007329	0.022907	L-aminoadipate-semialdehyde dehydrogenase EC:1.2.1.31
K05663	0.162548	0.007332	0.022907	mitochondrial ABC transporter ATM

K03165	0.162571	0.007324	0.022907	DNA topoisomerase III EC:5.99.1.2
K13722	0.162551	0.007331	0.022907	tricorn protease interacting factor F2/3 EC:3.4.11.-
K14775	0.162544	0.007334	0.022907	ribosome biogenesis protein UTP30
K02455	0.162528	0.00734	0.022916	general secretion pathway protein F
K11136	0.162507	0.007347	0.022929	regulator of telomere elongation helicase 1 EC:3.6.4.12
K00529	0.162431	0.007375	0.023004	ferredoxin--NAD+ reductase EC:1.18.1.3
K11338	0.162423	0.007378	0.023004	RuvB-like protein 2 EC:3.6.4.12
K00035	0.162412	0.007382	0.023006	D-galactose 1-dehydrogenase EC:1.1.1.48
K04082	-0.16255	0.007386	0.023007	molecular chaperone HscB
K04648	0.162367	0.007399	0.023038	dynactin 1
K11838	0.162342	0.007408	0.023055	ubiquitin carboxyl-terminal hydrolase 7 EC:3.1.2.15
K03710	-0.16237	0.007452	0.023183	GntR family transcriptional regulator; GntR family transcriptional regulator
K00903	-0.1623	0.007479	0.023245	protein-tyrosine kinase EC:2.7.10.-
K02932	0.162159	0.007476	0.023245	large subunit ribosomal protein L5e
K15115	0.162095	0.0075	0.023299	solute carrier family 25 (mitochondrial folate transporter), member 32
K04460	0.162038	0.007521	0.023355	protein phosphatase 5 EC:3.1.3.16
K00052	-0.16209	0.007556	0.023443	3-isopropylmalate dehydrogenase EC:1.1.1.85
K14824	0.161953	0.007553	0.023443	ribosome biogenesis protein ERB1
K13654	0.162022	0.007583	0.023516	GntR family transcriptional regulator, colanic acid and biofilm gene transcriptional regulator
K14786	0.161857	0.00759	0.023525	protein KRI1
K00235	0.161836	0.007597	0.023538	succinate dehydrogenase (ubiquinone) iron-sulfur subunit EC:1.3.5.1
K13837	0.161741	0.007633	0.023639	thrombospondin-related anonymous protein
K07126	0.161878	0.007638	0.023642	5-hydroxyisourate hydrolase EC:3.5.2.17; 5-hydroxyisourate hydrolase EC:3.5.2.17
K14333	0.161699	0.007649	0.023668	2,3-dihydroxybenzoate decarboxylase EC:4.1.1.46
K13483	0.161661	0.007664	0.023701	xanthine dehydrogenase YagT iron-sulfur-binding subunit
K12523	-0.16164	0.007673	0.02372	minor pilin subunit PapK
K00754	-0.16177	0.00768	0.023722	pyrimidine-nucleoside phosphorylase EC:2.4.2.2
K01212	-0.16177	0.007677	0.023722	levanase EC:3.2.1.65
K11827	0.161587	0.007692	0.023746	AP-2 complex subunit sigma-1
K03678	0.16154	0.00771	0.023791	exosome complex component RRP45
K03423	0.161672	0.007716	0.0238	TatD DNase family protein EC:3.1.21.-
K04412	0.161489	0.00773	0.023831	serine/threonine kinase 3 EC:2.7.11.5
K00375	0.161596	0.007745	0.023867	GntR family transcriptional regulator / MocR family aminotransferase; GntR family transcriptional regulator / MocR family aminotransferase
K09774	-0.16159	0.007748	0.023867	lipopolysaccharide export system protein LptA
K14536	0.161423	0.007755	0.023877	ribosome assembly protein 1 EC:3.6.5.-
K14402	0.161254	0.00782	0.024067	cleavage and polyadenylation specificity factor subunit 2
K01761	0.161387	0.007826	0.024073	methionine-gamma-lyase EC:4.4.1.11
K14779	0.161218	0.007834	0.024089	ATP-dependent RNA helicase DDX52/ROK1 EC:3.6.4.13
K00282	0.16132	0.007851	0.024121	glycine dehydrogenase subunit 1 EC:1.4.4.2
K00953	0.161165	0.007855	0.024121	FAD synthetase EC:2.7.7.2
K11393	0.161165	0.007855	0.024121	methyltransferase EC:2.1.1.-
K13941	0.161154	0.007859	0.024122	2-amino-4-hydroxy-6-hydroxymethyldihydropteridine diphosphokinase / dihydropteroate synthase EC:2.7.6.3 2.5.1.15
K11915	0.161119	0.007873	0.024154	serine/threonine protein phosphatase Stp1 EC:3.1.3.16
K00993	0.161094	0.007883	0.024173	ethanolaminephosphotransferase EC:2.7.8.1
K05834	-0.16122	0.00789	0.024184	homoserine/homoserine lactone efflux protein

K02426	-0.16118	0.007906	0.02422	cysteine desulfuration protein SufE
K03605	0.161173	0.007909	0.02422	hydrogenase 1 maturation protease EC:3.4.24.-
K07287	-0.16115	0.007918	0.024227	lipoprotein-34
K12816	0.16101	0.007915	0.024227	pre-mRNA-processing factor 17
K09935	0.161139	0.007922	0.024229	hypothetical protein; hypothetical protein
K01893	0.161015	0.007971	0.024367	asparaginyl-tRNA synthetase EC:6.1.1.22
K00297	-0.16088	0.008026	0.024497	methylenetetrahydrofolate reductase (NADPH) EC:1.5.1.20
K06027	0.160868	0.008028	0.024497	vesicle-fusing ATPase EC:3.6.4.6
K08998	0.160875	0.008026	0.024497	hypothetical protein; hypothetical protein
K02898	0.160734	0.008025	0.024497	large subunit ribosomal protein L26e
K12836	0.160718	0.008031	0.024497	splicing factor U2AF 35 kDa subunit
K06269	0.160683	0.008045	0.024528	protein phosphatase 1, catalytic subunit EC:3.1.3.16
K03787	-0.16081	0.00805	0.024534	5'-nucleotidase EC:3.1.3.5
K02872	0.160657	0.008055	0.024538	large subunit ribosomal protein L13Ae
K00045	-0.16071	0.008092	0.02464	mannitol 2-dehydrogenase EC:1.1.1.67
K01829	0.160694	0.008098	0.024646	protein disulfide-isomerase EC:5.3.4.1
K03579	0.160616	0.008129	0.02473	ATP-dependent helicase HrpB EC:3.6.4.13
K03431	0.160564	0.008149	0.024782	phosphoglucosamine mutase EC:5.4.2.10
K09970	-0.16053	0.008164	0.024814	general L-amino acid transport system permease protein
K01176	0.160508	0.008172	0.024829	alpha-amylase EC:3.2.1.1
K05367	-0.16049	0.008178	0.024836	penicillin-binding protein 1C EC:2.4.1.-
K05934	-0.16045	0.008195	0.024876	precorrin-3B C17-methyltransferase EC:2.1.1.131
K02843	-0.16036	0.008233	0.024982	heptosyltransferase II EC:2.4.-.-
K01154	0.160325	0.008246	0.02501	type I restriction enzyme, S subunit EC:3.1.21.3
K13421	0.160115	0.008273	0.025082	uridine monophosphate synthetase EC:2.4.2.10 4.1.1.23
K14785	0.159984	0.008327	0.025233	ESF2/ABP1 family protein
K02958	0.159916	0.008355	0.025307	small subunit ribosomal protein S15e
K00653	0.159852	0.008381	0.025376	histone acetyltransferase EC:2.3.1.48
K07727	0.159943	0.008402	0.025427	putative transcriptional regulator; putative transcriptional regulator
K07464	0.159924	0.00841	0.02543	putative RecB family exonuclease; putative RecB family exonuclease
K12827	0.159782	0.00841	0.02543	splicing factor 3A subunit 3
K11366	0.159752	0.008423	0.025456	ubiquitin carboxyl-terminal hydrolase 22/27/51 EC:3.1.2.15
K14787	0.15968	0.008453	0.025535	multiple RNA-binding domain-containing protein 1
K13115	0.159614	0.00848	0.025608	coiled-coil domain-containing protein 130
K09494	0.159596	0.008488	0.025619	T-complex protein 1 subunit beta
K03103	-0.15958	0.008495	0.025631	multiple inositol-polyphosphate phosphatase EC:3.1.3.62
K13889	-0.15968	0.00851	0.025659	glutathione transport system substrate-binding protein
K09694	-0.15954	0.008512	0.025659	lipooligosaccharide transport system permease protein
K03260	0.15951	0.008523	0.025682	translation initiation factor 4G
K00288	0.159485	0.008534	0.025703	methylenetetrahydrofolate dehydrogenase (NADP+) / methenyltetrahydrofolate cyclohydrolase / formyltetrahydrofolate synthetase EC:1.5.1.5 3.5.4.9 6.3.4.3
K02218	0.159396	0.008571	0.025804	casein kinase 1 EC:2.7.11.1
K01184	0.15934	0.008595	0.025848	polygalacturonase EC:3.2.1.15
K03253	0.159334	0.008597	0.025848	translation initiation factor 3 subunit B
K09991	0.159347	0.008592	0.025848	hypothetical protein; hypothetical protein
K10817	0.159309	0.008608	0.025862	erythronolide synthase EC:2.3.1.94
K10258	0.159306	0.008609	0.025862	enoyl reductase EC:1.3.1.-

K07095	0.159349	0.00865	0.025972	N-acetylmuramic acid 6-phosphate etherase EC:4.2.1.126; N-acetylmuramic acid 6-phosphate etherase EC:4.2.1.126
K02502	0.159205	0.008711	0.026144	ATP phosphoribosyltransferase regulatory subunit
K14857	0.159033	0.008726	0.026177	AdoMet-dependent rRNA methyltransferase SPB1 EC:2.1.1.-
K08850	0.158987	0.008745	0.026225	aurora kinase, other EC:2.7.11.1
K08819	0.158969	0.008753	0.026236	cyclin-dependent kinase 12/13 EC:2.7.11.22 2.7.11.23
K04564	-0.15908	0.008764	0.026257	superoxide dismutase, Fe-Mn family EC:1.15.1.1
K10762	0.15891	0.008778	0.026289	putative replication protein
K10395	0.158902	0.008782	0.026289	kinesin family member 4/7/21/27
K01842	0.158858	0.008801	0.026333	lysine 2,3-aminomutase EC:5.4.3.2
K03885	-0.15892	0.008833	0.026419	NADH dehydrogenase EC:1.6.99.3
K09922	-0.15879	0.008888	0.026571	hypothetical protein; hypothetical protein
K01198	-0.15871	0.008924	0.026637	xylan 1,4-beta-xylosidase EC:3.2.1.37
K06030	0.158576	0.008923	0.026637	mitofusin EC:3.6.5.-
K14855	0.158571	0.008925	0.026637	ribosome assembly protein 4
K03256	0.158575	0.008924	0.026637	tRNA (adenine-N(1)-)-methyltransferase non-catalytic subunit
K01522	0.158546	0.008936	0.026658	bis(5'-adenosyl)-triphosphatase EC:3.6.1.29
K11191	-0.1585	0.008955	0.026703	PTS system, N-acetylmuramic acid-specific IIB component EC:2.7.1.69
K02350	0.158478	0.008966	0.026723	DNA polymerase zeta subunit EC:2.7.7.7
K08683	0.158392	0.009004	0.026824	3-hydroxyacyl-CoA dehydrogenase / 3-hydroxy-2-methylbutyryl-CoA dehydrogenase EC:1.1.1.35 1.1.1.178
K06689	0.158333	0.00903	0.026891	ubiquitin-conjugating enzyme E2 D/E EC:6.3.2.19
K11806	0.158254	0.009064	0.026982	WD repeat and SOF domain-containing protein 1
K13177	0.158226	0.009077	0.027008	ATP-dependent RNA helicase DDX1 EC:3.6.4.13
K00641	0.158338	0.009087	0.027025	homoserine O-acetyltransferase EC:2.3.1.31
K03313	-0.15828	0.009113	0.027093	Na+_H+ antiporter, NhaA family
K03210	0.158185	0.009155	0.027204	preprotein translocase subunit YajC
K10578	0.158034	0.009162	0.027215	ubiquitin-conjugating enzyme E2 J1 EC:6.3.2.19
K01858	-0.15813	0.00918	0.027237	myo-inositol-1-phosphate synthase EC:5.5.1.4
K02612	-0.15812	0.009182	0.027237	ring-1,2-phenylacetyl-CoA epoxidase subunit PaaD
K04072	0.158133	0.009178	0.027237	acetaldehyde dehydrogenase / alcohol dehydrogenase EC:1.2.1.10 1.1.1.1
K02814	-0.1579	0.009222	0.027344	PTS system, sorbose-specific IIC component
K02945	0.157994	0.00924	0.027378	small subunit ribosomal protein S1
K14213	0.157858	0.009241	0.027378	Xaa-Pro dipeptidase EC:3.4.13.9
K07273	0.157944	0.009262	0.027429	lysozyme
K14644	0.157783	0.009275	0.027455	subtilase-type proteinase RRT12 EC:3.4.21.-
K14688	0.157747	0.009291	0.027492	solute carrier family 30 (zinc transporter), member 1
K01593	-0.15772	0.009302	0.027511	aromatic-L-amino-acid decarboxylase EC:4.1.1.28
K03773	-0.15771	0.009308	0.027516	FKBP-type peptidyl-prolyl cis-trans isomerase FklB EC:5.2.1.8
K01150	-0.15782	0.009319	0.027537	deoxyribonuclease I EC:3.1.21.1
K10872	0.157663	0.009329	0.027556	meiotic recombination protein DMC1
K06282	-0.15776	0.009345	0.027591	hydrogenase small subunit EC:1.12.99.6
K08835	0.157535	0.009388	0.027706	oxidative-stress responsive protein 1 EC:2.7.11.1
K11904	-0.15757	0.009433	0.027817	type VI secretion system secreted protein VgrG
K04354	0.157443	0.00943	0.027817	protein phosphatase 2 (formerly 2A), regulatory subunit B
K09792	0.157485	0.009471	0.027903	hypothetical protein; hypothetical protein
K10609	0.15736	0.009468	0.027903	cullin 4
K01664	0.157453	0.009485	0.027934	para-aminobenzoate synthetase component II EC:2.6.1.85

K05919	0.157444	0.00949	0.027934	superoxide reductase EC:1.15.1.2
K14556	0.157295	0.009498	0.027936	U3 small nucleolar RNA-associated protein 12
K14563	0.157294	0.009498	0.027936	rRNA 2'-O-methyltransferase fibrillarin EC:2.1.1.-
K00355	0.157413	0.009504	0.027941	NAD(P)H dehydrogenase (quinone) EC:1.6.5.2
K12581	0.157054	0.00961	0.02824	CCR4-NOT transcription complex subunit 7/8
K06186	-0.15716	0.009621	0.028262	small protein A
K03130	0.156942	0.009662	0.028369	transcription initiation factor TFIID subunit 5
K06685	0.156895	0.009684	0.028421	maintenance of ploidy protein MOB1 (MPS1 binder 1)
K01715	0.156983	0.009703	0.028466	3-hydroxybutyryl-CoA dehydratase EC:4.2.1.55
K01582	0.156972	0.009708	0.02847	lysine decarboxylase EC:4.1.1.18
K02941	0.156786	0.009735	0.028535	large subunit ribosomal protein LP0
K13216	0.156698	0.009777	0.028646	nuclear inhibitor of protein phosphatase 1 EC:3.1.4.-
K00734	0.156684	0.009783	0.028653	galactosylxylosylprotein 3-beta-galactosyltransferase EC:2.4.1.134
K09157	0.156783	0.009798	0.028682	hypothetical protein; hypothetical protein
K00914	0.156618	0.009815	0.02872	phosphatidylinositol 3-kinase EC:2.7.1.137
K08057	0.156558	0.009843	0.028791	calreticulin
K06978	-0.15666	0.009857	0.028808	pantoate kinase EC:2.7.1.169; pantoate kinase EC:2.7.1.169
K02542	0.156529	0.009857	0.028808	minichromosome maintenance protein 6
K09819	0.156487	0.009877	0.028847	manganese/iron transport system permease protein
K14319	0.156482	0.009879	0.028847	Ran GTPase-activating protein 1
K02197	-0.15656	0.009902	0.02889	cytochrome c-type biogenesis protein CcmE
K07668	0.156563	0.009902	0.02889	two-component system, OmpR family, response regulator VicR
K09500	0.156346	0.009945	0.029002	T-complex protein 1 subunit theta
K00122	0.156336	0.01001	0.029177	formate dehydrogenase EC:1.2.1.2
K13887	0.156204	0.010013	0.029177	coronin-1C
K11720	-0.1563	0.010026	0.029202	lipopolysaccharide export system permease protein
K00057	0.156279	0.010038	0.029212	glycerol-3-phosphate dehydrogenase (NAD(P)+) EC:1.1.1.94
K05543	0.156154	0.010037	0.029212	tRNA-dihydrouridine synthase 2 EC:1.3.1.91
K12188	0.156101	0.010063	0.029272	ESCRT-II complex subunit VPS22
K01163	-0.15617	0.010091	0.029342	hypothetical protein; hypothetical protein
K12737	0.156022	0.010101	0.02936	peptidyl-prolyl cis-trans isomerase SDCCAG10 EC:5.2.1.8
K05360	0.155946	0.010138	0.029455	protein-disulfide reductase (glutathione) EC:1.8.4.2
K05755	0.155916	0.010153	0.029484	actin related protein 2/3 complex, subunit 4
K01347	0.155982	0.010182	0.029558	IgA-specific serine endopeptidase EC:3.4.21.72
K00197	0.155891	0.010227	0.029664	acetyl-CoA decarbonylase/synthase complex subunit gamma EC:2.1.1.-
K01912	-0.15589	0.010229	0.029664	phenylacetate-CoA ligase EC:6.2.1.30
K02960	0.155755	0.010232	0.029664	small subunit ribosomal protein S16e
K03578	0.155818	0.010262	0.02974	ATP-dependent helicase HrpA EC:3.6.4.13
K00565	0.155679	0.010269	0.029748	mRNA (guanine-N7-)-methyltransferase EC:2.1.1.56
K14799	0.155607	0.010305	0.029839	pre-rRNA-processing protein TSR1
K03018	0.155587	0.010315	0.029856	DNA-directed RNA polymerase III subunit RPC1 EC:2.7.7.6
K13891	-0.15556	0.010392	0.030067	glutathione transport system permease protein
K03527	-0.15553	0.010404	0.030089	4-hydroxy-3-methylbut-2-enyl diphosphate reductase EC:1.17.1.2
K10914	-0.15548	0.010432	0.030132	CRP/FNR family transcriptional regulator, cyclic AMP receptor protein
K04382	0.155353	0.010432	0.030132	protein phosphatase 2 (formerly 2A), catalytic subunit EC:3.1.3.16

K15102	0.155365	0.010426	0.030132	solute carrier family 25 (mitochondrial phosphate transporter), member 3
K05308	0.155461	0.01044	0.03014	gluconate dehydratase EC:4.2.1.39
K14848	0.155281	0.010468	0.030209	ribosome assembly protein RRB1
K00135	0.155314	0.010513	0.030328	succinate-semialdehyde dehydrogenase (NADP+) EC:1.2.1.16
K00339	-0.15529	0.010524	0.030346	NADH-quinone oxidoreductase subunit J EC:1.6.5.3
K06877	0.155235	0.010553	0.030416	DEAD/DEAH box helicase domain-containing protein; DEAD/DEAH box helicase domain-containing protein
K01267	0.155167	0.010587	0.030502	aspartyl aminopeptidase EC:3.4.11.21
K00263	0.155006	0.010607	0.030547	leucine dehydrogenase EC:1.4.1.9
K03973	-0.15512	0.010614	0.030553	phage shock protein C
K11202	-0.15498	0.010619	0.030557	PTS system, fructose-specific IIB-like component EC:2.7.1.69
K01216	-0.15496	0.01063	0.030574	licheninase EC:3.2.1.73
K00520	-0.15506	0.010641	0.030593	mercuric reductase EC:1.16.1.1
K12572	0.154922	0.01065	0.030605	PAB-dependent poly(A)-specific ribonuclease subunit 3
K01530	0.154855	0.010684	0.030692	phospholipid-translocating ATPase EC:3.6.3.1
K09201	0.154844	0.010689	0.030694	transcription factor YY
K15237	-0.15479	0.010715	0.030754	2,5-dichloro-2,5-cyclohexadiene-1,4-diol dehydrogenase 1 EC:1.3.1.-
K12997	0.154879	0.010734	0.030784	rhamnosyltransferase EC:2.4.1.-
K10743	0.154761	0.010732	0.030784	ribonuclease H2 subunit A EC:3.1.26.4
K14327	0.154804	0.010772	0.030881	regulator of nonsense transcripts 2
K02453	0.154785	0.010782	0.030897	general secretion pathway protein D
K00671	0.154553	0.010839	0.031048	glycylpeptide N-tetradecanoyltransferase EC:2.3.1.97
K13024	0.154533	0.01085	0.031064	inositol hexakisphosphate/diphosphoinositol-pentakisphosphate kinase EC:2.7.4.21 2.7.4.24
K00771	0.154425	0.010906	0.031212	protein xylosyltransferase EC:2.4.2.26
K08866	0.154383	0.010928	0.031262	serine/threonine-protein kinase TTK/MPS1 EC:2.7.12.1
K15201	0.154245	0.011	0.031456	general transcription factor 3C polypeptide 3 (transcription factor C subunit 4)
K08222	-0.15435	0.01101	0.031472	MFS transporter, YQGE family, putative transporter
K06196	-0.15432	0.011024	0.031497	cytochrome c-type biogenesis protein
K00016	0.154253	0.011059	0.031571	L-lactate dehydrogenase EC:1.1.1.27
K03013	0.154134	0.011059	0.031571	DNA-directed RNA polymerases I, II, and III subunit RPABC1
K13519	0.154071	0.011092	0.031653	lysophospholipid acyltransferase EC:2.3.1.51 2.3.1.23 2.3.1.-
K07794	-0.15417	0.011105	0.031676	putative tricarboxylic transport membrane protein
K03341	0.154006	0.011127	0.031725	O-phospho-L-seryl-tRNASEC:L-selenocysteinyl-tRNA synthase EC:2.9.1.2
K14863	0.153985	0.011138	0.031744	ribosome biogenesis protein YTM1
K03095	0.153929	0.011167	0.031815	SprT-like protein
K02183	0.153898	0.011184	0.031849	calmodulin
K02182	0.153906	0.011242	0.031963	crotonobetaine/carnitine-CoA ligase EC:6.2.1.-
K03408	0.15393	0.01123	0.031963	purine-binding chemotaxis protein CheW
K05350	0.153918	0.011236	0.031963	beta-glucosidase EC:3.2.1.21
K12867	0.153791	0.011241	0.031963	pre-mRNA-splicing factor SYF1
K00264	0.153586	0.011351	0.032247	glutamate synthase (NADPH/NADH) EC:1.4.1.13 1.4.1.14
K12852	0.153585	0.011352	0.032247	116 kDa U5 small nuclear ribonucleoprotein component
K03204	-0.15353	0.011383	0.032323	type IV secretion system protein VirB9
K10353	0.153427	0.011437	0.032463	deoxyadenosine kinase EC:2.7.1.76
K03972	0.153521	0.01145	0.032485	phage shock protein E

K10251	0.153362	0.011473	0.032537	beta-keto reductase EC:1.1.1.-
K03695	0.153454	0.011486	0.032562	ATP-dependent Clp protease ATP-binding subunit ClpB
K07494	0.153381	0.011526	0.032661	putative transposase; putative transposase
K07213	0.153358	0.011538	0.032669	copper chaperone
K02144	0.153245	0.011537	0.032669	V-type H+-transporting ATPase 54 kD subunit EC:3.6.3.14
K03723	0.153199	0.011625	0.032903	transcription-repair coupling factor (superfamily II helicase) EC:3.6.4.-
K12556	0.153056	0.011641	0.032933	penicillin-binding protein 2X EC:2.3.2.-
K05989	-0.15316	0.011647	0.032937	alpha-L-rhamnosidase EC:3.2.1.40
K03420	0.153149	0.011653	0.032941	proteasome regulatory subunit
K01940	-0.15308	0.011693	0.032987	argininosuccinate synthase EC:6.3.4.5
K03746	-0.15296	0.011692	0.032987	DNA-binding protein H-NS
K06883	0.153109	0.011675	0.032987	hemoglobin; hemoglobin
K14376	0.152983	0.011681	0.032987	poly(A) polymerase EC:2.7.7.19
K14454	0.152968	0.011689	0.032987	aspartate aminotransferase, cytoplasmic EC:2.6.1.1
K08856	0.152937	0.011707	0.033011	serine/threonine kinase 16 EC:2.7.11.1
K07559	0.15295	0.011763	0.033157	putative RNA 2'-phosphotransferase EC:2.7.1.-
K00921	0.15281	0.011777	0.033183	1-phosphatidylinositol-3-phosphate 5-kinase EC:2.7.1.150
K13252	0.152875	0.011804	0.033246	putrescine carbamoyltransferase EC:2.1.3.6
K14265	0.152729	0.011822	0.033282	tryptophan aminotransferase EC:2.6.1.27
K00954	0.152795	0.011849	0.033345	pantetheine-phosphate adenylyltransferase EC:2.7.7.3
K07648	-0.15278	0.011859	0.03335	two-component system, OmpR family, aerobic respiration control sensor histidine kinase ArcB EC:2.7.13.3
K13121	0.152661	0.011861	0.03335	protein FRA10AC1
K01101	0.15273	0.011886	0.033407	4-nitrophenyl phosphatase EC:3.1.3.41
K00347	-0.15269	0.011909	0.033459	Na+-transporting NADH_ubiquinone oxidoreductase subunit B EC:1.6.5.-
K01060	-0.15267	0.011922	0.033468	cephalosporin-C deacetylase EC:3.1.1.41
K06049	0.152554	0.011921	0.033468	magnesium chelatase accessory protein
K13106	0.152533	0.011933	0.033484	pre-mRNA-splicing factor CWC26
K00857	0.152584	0.011968	0.033569	thymidine kinase EC:2.7.1.21
K08730	0.152433	0.011989	0.033615	phosphatidylserine synthase 2 EC:2.7.8.29
K09684	0.15249	0.012021	0.03369	purine catabolism regulatory protein
K00374	-0.15242	0.012061	0.033789	nitrate reductase 1, gamma subunit EC:1.7.99.4
K06125	0.152258	0.012089	0.033854	4-hydroxybenzoate hexaprenyltransferase EC:2.5.1.-
K06381	0.152337	0.012108	0.033879	stage II sporulation protein D
K03115	0.152225	0.012108	0.033879	casein kinase II subunit beta
K00901	0.152324	0.012115	0.033884	diacylglycerol kinase EC:2.7.1.107
K03294	-0.15232	0.012119	0.033884	basic amino acid/polyamine antiporter, APA family
K14553	0.152149	0.012151	0.033947	U3 small nucleolar RNA-associated protein 18
K14815	0.152148	0.012152	0.033947	mRNA turnover protein 4
K14567	0.152074	0.012194	0.034052	U3 small nucleolar RNA-associated protein 14
K02760	-0.1521	0.012242	0.034172	PTS system, cellobiose-specific IIB component EC:2.7.1.69
K01523	0.152056	0.012269	0.034232	phosphoribosyl-ATP pyrophosphohydrolase EC:3.6.1.31
K03022	0.151924	0.012281	0.034238	DNA-directed RNA polymerase III subunit RPC8
K12304	0.151924	0.012281	0.034238	soluble calcium-activated nucleotidase 1 EC:3.6.1.6
K09002	0.151937	0.012337	0.034382	hypothetical protein; hypothetical protein
K05524	-0.15192	0.01235	0.034402	ferredoxin
K00027	0.151893	0.012363	0.034412	malate dehydrogenase (oxaloacetate-decarboxylating) EC:1.1.1.38
K07074	0.151894	0.012362	0.034412	UPF0755 protein; UPF0755 protein
K03154	-0.15186	0.012383	0.03444	sulfur carrier protein

K07214	-0.15186	0.01238	0.03444	enterochelin esterase and related enzymes
K10899	0.151729	0.012394	0.034457	ATP-dependent DNA helicase Q1 EC:3.6.4.12
K02476	0.151817	0.012407	0.034473	two-component system, CitB family, sensor kinase EC:2.7.13.3; two-component system, CitB family, sensor kinase EC:2.7.13.3
K12847	0.151702	0.01241	0.034473	U4/U6.U5 tri-snRNP-associated protein 2
K00940	-0.15179	0.012423	0.034497	nucleoside-diphosphate kinase EC:2.7.4.6
K00421	-0.15176	0.012442	0.034535	polyphenol oxidase EC:1.10.3.1
K04656	-0.15173	0.012455	0.034557	hydrogenase maturation protein HypF
K03474	-0.15168	0.012485	0.034598	pyridoxine 5-phosphate synthase EC:2.6.99.2
K05996	-0.15157	0.012485	0.034598	carboxypeptidase T EC:3.4.17.18
K10230	0.151576	0.012483	0.034598	sorbitol/mannitol transport system ATP-binding protein
K10400	0.151525	0.012513	0.034663	kinesin family member 15
K13095	0.15148	0.01254	0.034723	splicing factor 1
K03608	0.151443	0.012627	0.034934	cell division topological specificity factor
K10107	-0.15134	0.012623	0.034934	capsular polysaccharide transport system permease protein
K11718	0.151306	0.012643	0.034966	UDP-glucose_glycoprotein glucosyltransferase EC:2.4.1.-
K14082	-0.15125	0.012676	0.035043	methylcobamide_CoM methyltransferase EC:2.1.1.-
K12237	-0.15123	0.012687	0.03506	nonribosomal peptide synthetase VibF
K08986	0.151313	0.012703	0.035091	putative membrane protein
K01305	-0.15128	0.012722	0.035128	beta-aspartyl-dipeptidase (metallo-type) EC:3.4.19.-
K01034	0.151273	0.012727	0.035129	acetate CoA-transferase alpha subunit EC:2.8.3.8
K08827	0.151151	0.012736	0.035137	serine/threonine-protein kinase PRP4 EC:2.7.11.1
K00970	-0.15121	0.012766	0.035197	poly(A) polymerase EC:2.7.7.19
K07657	-0.15121	0.012767	0.035197	two-component system, OmpR family, phosphate regulon response regulator PhoB
K06639	0.150997	0.012828	0.035349	cell division cycle 14 EC:3.1.3.48
K07774	0.15105	0.01286	0.035425	two-component system, OmpR family, response regulator TctD
K00624	0.150931	0.012867	0.03543	carnitine O-acetyltransferase EC:2.3.1.7
K07183	0.150935	0.01293	0.035588	response regulator NasT
K05819	-0.1508	0.012947	0.03562	MFS transporter, AAHS family, 3-hydroxyphenylpropionic acid transporter
K13525	0.15086	0.012975	0.035685	transitional endoplasmic reticulum ATPase
K10878	0.150627	0.013052	0.03588	meiotic recombination protein SPO11
K09125	-0.15071	0.013065	0.035888	hypothetical protein; hypothetical protein
K10597	0.150613	0.01306	0.035888	ubiquitin conjugation factor E4 B EC:6.3.2.19
K04755	-0.15057	0.013088	0.035937	ferredoxin, 2Fe-2S
K10530	0.150531	0.013111	0.035985	lactate oxidase EC:1.13.12.-
K03363	0.150519	0.013118	0.035992	cell division cycle 20, cofactor of APC complex
K11684	0.15048	0.013142	0.036042	bromodomain-containing factor 1
K01144	0.150429	0.013239	0.036293	exodeoxyribonuclease V EC:3.1.11.5
K04772	-0.15037	0.013277	0.036355	serine protease DegQ EC:3.4.21.-
K09251	0.15038	0.013268	0.036355	putrescine aminotransferase EC:2.6.1.82
K05674	0.150268	0.013273	0.036355	ATP-binding cassette, subfamily C (CFTR/MRP), member 10
K07341	0.150325	0.013303	0.036411	death on curing protein
K08486	0.150135	0.013355	0.03654	syntaxin 1B/2/3
K01386	0.150086	0.013386	0.03661	microbial collagenase EC:3.4.24.3
K09749	0.150154	0.013409	0.036659	hypothetical protein; hypothetical protein
K00680	-0.15013	0.013426	0.036666	gamma-glutamyltranspeptidase EC:2.3.2.2
K01865	0.15013	0.013424	0.036666	tyrosyl-tRNA synthetase EC:6.1.1.1

K13043	-0.15012	0.013428	0.036666	N-succinyl-L-ornithine transcarbamylase EC:2.1.3.11
K03716	0.150098	0.013444	0.036695	spore photoproduct lyase EC:4.1.99.14
K01637	-0.14995	0.013473	0.03676	isocitrate lyase EC:4.1.3.1
K08515	0.149934	0.013481	0.036768	vesicle-associated membrane protein 7
K07342	0.149863	0.013526	0.036863	protein transport protein SEC61 subunit gamma and related proteins
K03028	0.149862	0.013527	0.036863	26S proteasome regulatory subunit N1
K11690	-0.14995	0.013536	0.036864	C4-dicarboxylate transporter, DctM subunit
K12844	0.149845	0.013537	0.036864	U4/U6 small nuclear ribonucleoprotein PRP31
K00895	-0.1499	0.013566	0.036908	pyrophosphate--fructose-6-phosphate 1-phosphotransferase EC:2.7.1.90
K01197	-0.1499	0.013567	0.036908	hyaluronoglucosaminidase EC:3.2.1.35
K06276	0.149793	0.01357	0.036908	3-phosphoinositide dependent protein kinase-1 EC:2.7.11.1
K03603	0.149743	0.013602	0.036981	GntR family transcriptional regulator, negative regulator for fad regulon and positive regulator of fabA
K05642	0.14968	0.013642	0.037074	ATP-binding cassette, subfamily A (ABC1), member 2
K00124	-0.14971	0.013686	0.037181	formate dehydrogenase, beta subunit
K03426	-0.14968	0.01371	0.037232	NAD+ diphosphatase EC:3.6.1.22
K02324	0.149482	0.013768	0.037361	DNA polymerase epsilon subunit 1 EC:2.7.7.7
K03163	0.149481	0.013769	0.037361	DNA topoisomerase I EC:5.99.1.2
K05804	0.149542	0.013795	0.037417	right origin-binding protein
K11124	0.149363	0.013844	0.037536	protein SMG6 EC:3.1.-.-
K10979	0.149311	0.013878	0.037612	DNA end-binding protein Ku
K02569	-0.14927	0.013902	0.037663	cytochrome c-type protein NapC
K14794	0.149187	0.013957	0.037799	ribosomal RNA-processing protein 12
K10010	0.149161	0.014041	0.038009	cystine transport system ATP-binding protein EC:3.6.3.-
K12825	0.149049	0.014048	0.038013	splicing factor 3A subunit 1
K02540	0.149031	0.014059	0.038014	minichromosome maintenance protein 2
K11087	0.149038	0.014055	0.038014	small nuclear ribonucleoprotein D1
K00128	0.149108	0.014075	0.038043	aldehyde dehydrogenase (NAD+) EC:1.2.1.3
K10245	0.14893	0.014125	0.038162	fatty acid elongase 2 EC:2.3.1.-
K07133	0.14902	0.014133	0.038168	molybdenum cofactor cytidylyltransferase EC:2.7.7.76; molybdenum cofactor cytidylyltransferase EC:2.7.7.76
K04498	0.148897	0.014147	0.038191	E1A/CREB-binding protein EC:2.3.1.48
K09474	-0.14878	0.014223	0.038381	acid phosphatase (class A) EC:3.1.3.2
K03844	0.14865	0.014309	0.0386	alpha-1,2-mannosyltransferase EC:2.4.1.-
K00992	-0.14866	0.01437	0.038748	ethanolaminephosphotransferase EC:2.7.8.1
K03340	-0.14845	0.01451	0.039112	diaminopimelate dehydrogenase EC:1.4.1.16
K02761	-0.14831	0.014536	0.039166	PTS system, cellobiose-specific IIC component
K00043	0.148362	0.014568	0.039215	4-hydroxybutyrate dehydrogenase EC:1.1.1.61
K03458	-0.14836	0.014571	0.039215	nucleobase_cation symporter-2, NCS2 family
K14776	0.148264	0.014567	0.039215	ATP-dependent RNA helicase DDX10/DBP4 EC:3.6.4.13
K01173	-0.14825	0.014643	0.039392	endonuclease G, mitochondrial
K11303	0.148052	0.01471	0.039553	histone acetyltransferase 1 EC:2.3.1.48
K13721	0.148046	0.014714	0.039553	aminopeptidase 2 EC:3.4.11.-
K03617	0.148132	0.014722	0.03956	electron transport complex protein RnfA
K05739	0.148	0.014746	0.039607	hypothetical protein
K11412	0.147965	0.014769	0.039654	NAD-dependent deacetylase sirtuin 2 EC:3.5.1.-
K02951	0.147931	0.014793	0.039702	small subunit ribosomal protein S12e
K10126	-0.14792	0.014799	0.039704	two-component system, NtrC family, C4-dicarboxylate transport response regulator DctD
K02808	-0.14801	0.014807	0.039709	PTS system, sucrose-specific IIA component EC:2.7.1.69

K01579	-0.14795	0.014843	0.039791	aspartate 1-decarboxylase EC:4.1.1.11
K04705	0.147845	0.014851	0.039797	signal transducing adaptor molecule
K09686	0.147922	0.014865	0.039819	antibiotic transport system permease protein
K14645	0.147793	0.014887	0.039861	serine protease EC:3.4.21.-; serine protease EC:3.4.21.-
K14856	0.147777	0.014898	0.039876	protein SDA1
K07501	0.147764	0.014907	0.039884	hypothetical protein; hypothetical protein
K02739	0.147744	0.01492	0.03989	20S proteasome subunit beta 2 EC:3.4.25.1
K12859	0.147746	0.014919	0.03989	U5 snRNP protein, DIM1 family
K12849	0.1477	0.014951	0.039955	pre-mRNA-splicing factor 38A
K02405	0.14775	0.014983	0.040025	RNA polymerase sigma factor for flagellar operon FliA
K02320	0.147591	0.015026	0.040095	DNA polymerase alpha subunit A EC:2.7.7.7
K03849	0.147592	0.015025	0.040095	alpha-1,3-glucosyltransferase EC:2.4.1.265
K15216	0.147591	0.015026	0.040095	RNA polymerase I-specific transcription initiation factor RRN3
K10389	0.14754	0.015061	0.040173	tubulin gamma
K05394	-0.14758	0.0151	0.040247	atrazine chlorohydrolase EC:3.8.1.8
K11520	-0.14758	0.015101	0.040247	two-component system, OmpR family, manganese sensing sensor histidine kinase EC:2.7.13.3
K03721	-0.14754	0.015127	0.040302	transcriptional regulator of aroF, aroG, tyrA and aromatic amino acid transport
K00226	-0.1475	0.015152	0.040354	dihydroorotate dehydrogenase (fumarate) EC:1.3.98.1
K14411	0.147326	0.01521	0.040491	RNA-binding protein Musashi
K04030	-0.14737	0.015244	0.040552	ethanolamine utilization protein EutQ
K02155	0.147283	0.01524	0.040552	V-type H+-transporting ATPase 16kDa proteolipid subunit EC:3.6.3.14
K01917	-0.14734	0.015265	0.04059	glutathionylspermidine synthase EC:6.3.1.8
K01774	0.147172	0.015318	0.040716	alanine racemase EC:5.1.1.1
K02837	-0.14726	0.015325	0.04072	peptide chain release factor 3
K07016	0.14724	0.015337	0.040735	putative hydrolases of HD superfamily; putative hydrolases of HD superfamily
K00303	0.147213	0.015356	0.040771	sarcosine oxidase, subunit beta EC:1.5.3.1
K13614	0.147103	0.015367	0.040783	polyketide synthase PksN
K14831	0.147076	0.015385	0.040817	protein MAK16
K04027	-0.14715	0.0154	0.040839	ethanolamine utilization protein EutM
K02486	0.147042	0.015409	0.040848	two-component system, unclassified family, sensor kinase EC:2.7.13.3; two-component system, unclassified family, sensor kinase EC:2.7.13.3
K00120	0.14707	0.015457	0.04095	S-(hydroxymethyl)glutathione dehydrogenase / alcohol dehydrogenase EC:1.1.1.284 1.1.1.1
K07145	-0.14707	0.015459	0.04095	UPF0176 protein; UPF0176 protein
K11204	0.146944	0.015479	0.040986	glutamate--cysteine ligase catalytic subunit EC:6.3.2.2
K13207	0.146904	0.015507	0.041045	CUG-BP- and ETR3-like factor
K09988	0.146955	0.015538	0.041112	hypothetical protein; hypothetical protein
K05805	-0.14672	0.015636	0.041353	CreA protein
K06895	-0.1468	0.015648	0.04137	L-lysine exporter family protein LysE/ArgO
K08227	-0.14674	0.01569	0.041451	MFS transporter, LPLT family, lysophospholipid transporter
K06312	0.146651	0.015688	0.041451	spore germination protein
K07001	-0.1467	0.015721	0.041517	NTE family protein; NTE family protein
K02200	-0.14669	0.015728	0.041519	cytochrome c-type biogenesis protein CcmH
K09689	-0.14656	0.015754	0.041571	capsular polysaccharide transport system ATP-binding protein
K05979	0.146631	0.015769	0.041595	2-phosphosulfolactate phosphatase EC:3.1.3.71
K00982	-0.1465	0.015861	0.041805	glutamate-ammonia-ligase adenylyltransferase EC:2.7.7.42

K03852	0.146417	0.015857	0.041805	sulfoacetaldehyde acetyltransferase EC:2.3.3.15
K14007	0.146269	0.015964	0.042062	protein transport protein SEC24
K02428	-0.14635	0.015973	0.042069	dITP/XTP pyrophosphatase EC:3.6.1.19
K00359	0.146311	0.016001	0.042115	NADH oxidase EC:1.6.-.-
K07219	-0.14631	0.016003	0.042115	putative molybdopterin biosynthesis protein; putative molybdopterin biosynthesis protein
K14552	0.146169	0.016037	0.04219	NET1-associated nuclear protein 1 (U3 small nucleolar RNA-associated protein 17)
K06630	0.146158	0.016045	0.042194	tyrosine 3-monooxygenase/tryptophan 5-monooxygenase activation protein
K06953	0.146134	0.016063	0.042224	tRNA(Met) cytidine acetyltransferase EC:2.3.1.193; tRNA(Met) cytidine acetyltransferase EC:2.3.1.193
K05883	-0.14609	0.016095	0.042293	(R)-2-hydroxyacid dehydrogenase EC:1.1.1.272
K15223	0.146056	0.01612	0.042343	upstream activation factor subunit UAF30
K01084	0.145989	0.016169	0.042455	glucose-6-phosphatase EC:3.1.3.9
K09377	0.145933	0.016211	0.042549	cysteine and glycine-rich protein
K06142	-0.14586	0.016329	0.042826	outer membrane protein
K11021	0.145865	0.016328	0.042826	insecticidal toxin complex protein TccC
K03246	0.145613	0.016449	0.043124	translation initiation factor 3 subunit I
K03757	-0.14558	0.016471	0.043165	cadaverine_lysine antiporter
K10548	0.145659	0.016482	0.043177	putative multiple sugar transport system ATP-binding protein
K09903	0.145615	0.016514	0.043247	uridylate kinase EC:2.7.4.22
K00590	0.145522	0.016584	0.043413	site-specific DNA-methyltransferase (cytosine-N4-specific) EC:2.1.1.113
K08776	0.145423	0.016591	0.043414	puromycin-sensitive aminopeptidase EC:3.4.11.-
K03620	-0.1455	0.016599	0.043419	Ni/Fe-hydrogenase 1 B-type cytochrome subunit
K12521	-0.1453	0.016687	0.043632	minor pilin subunit PapF
K15109	0.145257	0.016717	0.043694	solute carrier family 25 (mitochondrial carnitine/acylcarnitine transporter), member 20/29
K01693	0.145275	0.016771	0.043818	imidazoleglycerol-phosphate dehydratase EC:4.2.1.19
K09858	-0.14525	0.016786	0.043842	SEC-C motif domain protein; SEC-C motif domain protein
K01696	-0.14521	0.016821	0.043877	tryptophan synthase beta chain EC:4.2.1.20
K02009	-0.1452	0.016825	0.043877	cobalt transport protein
K11358	0.14522	0.016812	0.043877	aspartate aminotransferase EC:2.6.1.1
K11788	0.145114	0.016825	0.043877	phosphoribosylamine--glycine ligase / phosphoribosylformylglycinamidine cyclo-ligase EC:6.3.4.13 6.3.3.1
K06307	0.145081	0.01685	0.043927	spore germination protein
K14568	0.145059	0.016868	0.043955	essential for mitotic growth 1
K11099	0.144989	0.016921	0.044078	small nuclear ribonucleoprotein G
K02217	-0.14507	0.016928	0.044079	ferritin EC:1.16.3.1
K12245	0.144832	0.017042	0.044359	bifunctional glycosyltransferase PgtA EC:2.4.1.- 2.4.1.69
K14016	0.144798	0.017068	0.04441	ubiquitin fusion degradation protein 1
K14347	0.144768	0.017091	0.044454	solute carrier family 10 (sodium/bile acid cotransporter), member 7
K08323	-0.14477	0.017153	0.044599	starvation sensing protein RspA
K07257	-0.14462	0.017205	0.044684	spore coat polysaccharide biosynthesis protein SpsF
K03255	0.144629	0.017199	0.044684	protein TIF31
K14269	0.144637	0.017193	0.044684	glutarate semialdehyde dehydrogenase EC:1.2.1.20
K01202	0.144654	0.017247	0.044758	galactosylceramidase EC:3.2.1.46
K04760	-0.14466	0.017241	0.044758	transcription elongation factor GreB
K10229	-0.14455	0.017258	0.044769	sorbitol/mannitol transport system permease protein
K04756	-0.14454	0.017269	0.044782	alkyl hydroperoxide reductase subunit D

K03688	0.14461	0.017281	0.044789	ubiquinone biosynthesis protein
K14596	0.144518	0.017285	0.044789	zeaxanthin glucosyltransferase EC:2.4.1.276
K06015	0.144456	0.017402	0.045058	N-acyl-D-amino-acid deacylase EC:3.5.1.81
K12272	0.144371	0.017401	0.045058	signal recognition particle receptor subunit beta
K02145	0.144342	0.017423	0.045096	V-type H+-transporting ATPase subunit A EC:3.6.3.14
K10844	0.144168	0.017629	0.045611	DNA excision repair protein ERCC-2 EC:3.6.4.12
K00538	0.144003	0.017692	0.045757	formate acetyltransferase activating enzyme EC:1.97.1.4
K00011	0.143962	0.017724	0.045824	aldehyde reductase EC:1.1.1.21
K07790	0.143948	0.017735	0.045836	putative membrane protein PagO
K10535	-0.14389	0.017785	0.045947	hydroxylamine oxidase EC:1.7.3.4
K11129	0.14386	0.017806	0.045983	H/ACA ribonucleoprotein complex subunit 2
K00916	0.143825	0.017835	0.04604	CTD kinase subunit alpha EC:2.7.11.22 2.7.11.23
K13026	0.143729	0.017911	0.046221	
K08255	0.143718	0.01792	0.046227	CoA-disulfide reductase EC:1.8.1.14
K01848	-0.14378	0.017941	0.046262	methylmalonyl-CoA mutase, N-terminal domain EC:5.4.99.2
K00342	-0.14374	0.017971	0.046296	NADH-quinone oxidoreductase subunit M EC:1.6.5.3
K07220	-0.14375	0.017962	0.046296	hypothetical protein; hypothetical protein
K13922	-0.14374	0.017974	0.046296	propionaldehyde dehydrogenase
K03072	0.143689	0.018011	0.046363	preprotein translocase subunit SecD
K12877	0.143603	0.018013	0.046363	protein mago nashi
K07812	-0.14365	0.018046	0.04643	trimethylamine-N-oxide reductase (cytochrome c) 2 EC:1.7.2.3
K01214	0.143352	0.018217	0.046843	isoamylase EC:3.2.1.68
K06995	-0.14335	0.01822	0.046843	phospholipase/carboxylesterase; phospholipase/carboxylesterase
K09493	0.143306	0.018255	0.046916	T-complex protein 1 subunit alpha
K00189	0.143293	0.018266	0.046925	2-oxoisovalerate ferredoxin oxidoreductase, gamma subunit EC:1.2.7.7
K03066	0.143266	0.018288	0.046965	26S proteasome regulatory subunit T6
K14864	0.143173	0.018364	0.047142	tRNA (cytidine32/guanosine34-2'-O)-methyltransferase EC:2.1.1.205
K09158	-0.1429	0.018655	0.047873	hypothetical protein; hypothetical protein
K07171	0.142807	0.018667	0.047886	mRNA interferase EC:3.1.-.-; mRNA interferase EC:3.1.-.-
K00689	-0.14286	0.018688	0.047921	dextransucrase EC:2.4.1.5
K01525	-0.14281	0.018734	0.048022	bis(5'-nucleosyl)-tetraphosphatase (symmetrical) EC:3.6.1.41
K13007	-0.14274	0.018792	0.048153	Fuc2NAc and GlcNAc transferase EC:2.4.1.-
K03694	-0.14269	0.018837	0.04825	ATP-dependent Clp protease ATP-binding subunit ClpA
K11751	-0.14267	0.018851	0.048267	5'-nucleotidase / UDP-sugar diphosphatase EC:3.1.3.5 3.6.1.45
K10575	0.142575	0.018862	0.048278	ubiquitin-conjugating enzyme E2 G1 EC:6.3.2.19
K00194	0.142622	0.01889	0.048314	acetyl-CoA decarbonylase/synthase complex subunit delta
K13048	0.142547	0.018886	0.048314	carboxypeptidase Ss1 EC:3.4.17.-
K01629	-0.14261	0.018901	0.048323	rhamnulose-1-phosphate aldolase EC:4.1.2.19
K04728	0.142503	0.018923	0.048363	ataxia telangiectasia mutated family protein EC:2.7.11.1
K10595	0.14249	0.018934	0.048372	E3 ubiquitin-protein ligase HERC2 EC:6.3.2.19
K02321	0.142467	0.018954	0.048405	DNA polymerase alpha subunit B
K04691	0.142529	0.018969	0.048425	serine protease DegS EC:3.4.21.-
K00435	0.142495	0.018998	0.048464	peroxiredoxin EC:1.11.1.-
K07265	-0.14241	0.019003	0.048464	capsular polysaccharide export protein
K03004	0.142406	0.019005	0.048464	DNA-directed RNA polymerase I subunit RPA43
K00356	0.142452	0.019034	0.048521	NADH dehydrogenase EC:1.6.99.3

K00284	0.142398	0.01908	0.048619	glutamate synthase (ferredoxin) EC:1.4.7.1
K00831	0.142275	0.019185	0.048868	phosphoserine aminotransferase EC:2.6.1.52
K01719	-0.14222	0.019232	0.04897	uroporphyrinogen-III synthase EC:4.2.1.75
K10355	0.142126	0.019244	0.048984	actin, other eukaryote
K03312	-0.14209	0.019345	0.049221	glutamate_Na+ symporter, ESS family
K00639	-0.14206	0.019372	0.049244	glycine C-acetyltransferase EC:2.3.1.29
K03610	0.142053	0.019375	0.049244	septum site-determining protein MinC
K07469	-0.14205	0.019375	0.049244	aldehyde dehydrogenase (FAD-independent) EC:1.2.99.7
K00003	0.142014	0.019408	0.04931	homoserine dehydrogenase EC:1.1.1.3
K07118	-0.14199	0.019428	0.049342	5-hydroxyisourate hydrolase EC:3.5.2.17; 5-hydroxyisourate hydrolase EC:3.5.2.17
K06675	0.141869	0.019466	0.04942	structural maintenance of chromosome 4
K00726	0.14183	0.019499	0.049487	alpha-1,3-mannosyl-glycoprotein beta-1,2-N-acetylglucosaminyltransferase EC:2.4.1.101
K14168	0.141801	0.019524	0.049532	cytoplasmic tRNA 2-thiolation protein 1 EC:2.7.7.-
K01265	0.14185	0.01955	0.049579	methionyl aminopeptidase EC:3.4.11.18
K03847	0.14174	0.019577	0.04963	alpha-1,6-mannosyltransferase EC:2.4.1.260
K00207	0.141731	0.019585	0.049632	dihydropyrimidine dehydrogenase (NADP+) EC:1.3.1.2
K00253	0.141684	0.019626	0.0497	isovaleryl-CoA dehydrogenase EC:1.3.8.4
K06317	0.141691	0.01962	0.0497	inhibitor of the pro-sigma K processing machinery
K10841	0.14174	0.019646	0.049731	DNA excision repair protein ERCC-6
K08321	-0.14172	0.019663	0.049755	putative autoinducer-2 (AI-2) aldolase EC:4.1.2.-
K00366	-0.14168	0.019696	0.049802	ferredoxin-nitrite reductase EC:1.7.7.1
K13500	0.141686	0.019693	0.049802	chondroitin synthase EC:2.4.1.175 2.4.1.226
K08304	-0.14164	0.019733	0.049877	membrane-bound lytic murein transglycosylase A EC:3.2.1.-
K05807	-0.14159	0.01978	0.049979	putative lipoprotein
K14742	0.141571	0.019793	0.049993	hypothetical protease EC:3.4.-.-
K10108	0.14155	0.019811	0.050021	maltose/maltodextrin transport system substrate-binding protein
K03830	-0.14145	0.019898	0.050202	putative acetyltransferase EC:2.3.1.-
K12349	0.141383	0.01989	0.050202	neutral ceramidase EC:3.5.1.23
K13069	0.141442	0.019907	0.050207	diguanylate cyclase EC:2.7.7.65
K11031	-0.14136	0.019914	0.050207	thiol-activated cytolysin
K00105	0.141316	0.01995	0.050278	alpha-glycerophosphate oxidase EC:1.1.3.21
K13019	0.141217	0.020037	0.05048	UDP-GlcNAc3NAcA epimerase EC:5.1.3.23
K09612	0.141233	0.020092	0.050599	alkaline phosphatase isozyme conversion protein EC:3.4.11.-
K00046	-0.14102	0.020284	0.051065	gluconate 5-dehydrogenase EC:1.1.1.69
K00184	-0.141	0.020303	0.051094	molybdopterin oxidoreductase, iron-sulfur binding subunit EC:1.2.7.-
K01495	-0.14097	0.020329	0.051126	GTP cyclohydrolase I EC:3.5.4.16
K07675	0.140965	0.020331	0.051126	two-component system, NarL family, sensor histidine kinase UhpB EC:2.7.13.3
K00863	0.140951	0.020343	0.051139	dihydroxyacetone kinase EC:2.7.1.29
K01067	0.140918	0.020373	0.051193	acetyl-CoA hydrolase EC:3.1.2.1
K03600	-0.14091	0.020379	0.051193	stringent starvation protein B
K12398	0.140787	0.020422	0.051282	AP-3 complex subunit mu
K06677	0.140734	0.02047	0.051384	condensin complex subunit 1
K14570	0.140631	0.020564	0.0516	RNA exonuclease 1 EC:3.1.-.-
K09861	-0.14068	0.020586	0.051637	hypothetical protein; hypothetical protein
K05788	-0.14055	0.020705	0.051917	integration host factor subunit beta
K12209	0.140339	0.020831	0.052191	intracellular multiplication protein IcmE

K04441	0.140332	0.020837	0.052191	p38 MAP kinase EC:2.7.11.24
K09571	0.140339	0.02083	0.052191	FK506-binding protein 4/5 EC:5.2.1.8
K01565	0.140314	0.020853	0.052212	N-sulfoglucosamine sulfohydrolase EC:3.10.1.1
K12860	0.140261	0.020903	0.052317	pre-mRNA-splicing factor CDC5/CEF1
K11979	0.14024	0.020921	0.052345	E3 ubiquitin-protein ligase UBR7 EC:6.3.2.19
K02483	0.140264	0.020968	0.052443	two-component system, OmpR family, response regulator; two-component system, OmpR family, response regulator
K02004	-0.14024	0.020988	0.052475	putative ABC transport system permease protein
K02655	0.14013	0.021023	0.052542	type IV pilus assembly protein PilE
K03681	0.140105	0.021047	0.052583	exosome complex component RRP40
K00336	0.140156	0.021068	0.052616	NADH-quinone oxidoreductase subunit G EC:1.6.5.3
K00283	0.140063	0.021153	0.052806	glycine dehydrogenase subunit 2 EC:1.4.4.2
K11094	0.139984	0.021159	0.052806	U2 small nuclear ribonucleoprotein B''
K07877	0.139958	0.021183	0.052847	Ras-related protein Rab-2A
K11658	0.139925	0.021214	0.052904	bromodomain adjacent to zinc finger domain protein 1B
K00752	0.139889	0.021247	0.052969	hyaluronan synthase EC:2.4.1.212
K00693	0.139846	0.021288	0.05305	glycogen(starch) synthase EC:2.4.1.11
K05311	0.139766	0.021431	0.053387	central glycolytic genes regulator
K07319	0.139741	0.021454	0.053425	putative adenine-specific DNA-methyltransferase EC:2.1.1.72
K09815	0.139608	0.021579	0.053719	zinc transport system substrate-binding protein
K02657	-0.13952	0.021592	0.05373	twitching motility two-component system response regulator PilG
K02234	0.139562	0.021623	0.053788	cobalamin biosynthesis protein CobW
K03851	-0.13941	0.021699	0.05394	taurine-pyruvate aminotransferase EC:2.6.1.77
K00360	-0.13942	0.021692	0.05394	nitrate reductase (NADH) EC:1.7.1.1
K09680	-0.13937	0.021736	0.054012	type II pantothenate kinase EC:2.7.1.33
K01268	-0.13928	0.021828	0.05422	aminopeptidase I EC:3.4.11.22
K06145	0.139247	0.021855	0.054267	LacI family transcriptional regulator, gluconate utilization system Gnt-I transcriptional repressor
K09132	-0.13925	0.02192	0.054409	hypothetical protein; hypothetical protein
K11337	0.139066	0.022028	0.054659	3-hydroxyethyl bacteriochlorophyllide a dehydrogenase EC:1.-.-.-
K06980	-0.13911	0.022056	0.054708	pantoate kinase EC:2.7.1.169; pantoate kinase EC:2.7.1.169
K06676	0.139013	0.02208	0.054749	condensin complex subunit 2
K11050	0.138998	0.022095	0.054764	multidrug/hemolysin transport system ATP-binding protein
K03411	0.138976	0.022184	0.054966	chemotaxis protein CheD EC:3.5.1.44
K14820	0.138877	0.022212	0.055016	ribosome biogenesis protein BRX1
K14981	0.138866	0.022223	0.055023	two-component system, OmpR family, response regulator ChvI
K13038	-0.13881	0.022347	0.055311	phosphopantothenoylcysteine decarboxylase / phosphopantothenate--cysteine ligase EC:4.1.1.36 6.3.2.5
K03928	0.138782	0.022372	0.055353	carboxylesterase EC:3.1.1.1
K11805	0.138698	0.022386	0.055369	WD repeat-containing protein 68
K12826	0.138686	0.022398	0.055377	splicing factor 3A subunit 2
K13217	0.138611	0.022471	0.055538	pre-mRNA-processing factor 39
K02531	0.138574	0.022507	0.055608	transcriptional antiterminator
K01411	0.138538	0.022543	0.055675	nardilysin EC:3.4.24.61
K00068	0.138598	0.022552	0.055679	sorbitol-6-phosphate 2-dehydrogenase EC:1.1.1.140
K02243	0.138544	0.022605	0.055769	competence protein ComGA
K07302	0.138479	0.022601	0.055769	isoquinoline 1-oxidoreductase, alpha subunit EC:1.3.99.16

K00098	-0.13846	0.022623	0.055793	L-idonate 5-dehydrogenase EC:1.1.1.264
K08985	-0.13841	0.022669	0.055887	putative lipoprotein; putative lipoprotein
K01728	-0.13846	0.022686	0.055908	pectate lyase EC:4.2.2.2
K07175	-0.13843	0.022713	0.055956	PhoH-like ATPase
K03281	-0.13835	0.022801	0.056151	chloride channel protein, CIC family
K03693	0.138268	0.02281	0.056155	penicillin-binding protein
K02841	-0.13825	0.022899	0.056334	heptosyltransferase I EC:2.4.-.-
K07266	-0.13818	0.022897	0.056334	capsular polysaccharide export protein
K14656	0.138153	0.022924	0.056375	FAD synthetase
K12981	-0.13812	0.022957	0.056436	KDO transferase III EC:2.-.-.-
K02488	0.138176	0.022969	0.056445	two-component system, cell cycle response regulator
K03560	-0.13814	0.023005	0.056514	biopolymer transport protein TolR
K03388	0.138099	0.023046	0.056594	heterodisulfide reductase subunit A EC:1.8.98.1
K03803	-0.138	0.023143	0.056813	sigma-E factor negative regulatory protein RseC
K00537	0.137986	0.023159	0.056831	arsenate reductase EC:1.20.4.1
K05925	0.137896	0.023182	0.056869	mRNA (2'-O-methyladenosine-N6-)-methyltransferase EC:2.1.1.62
K07936	0.137845	0.023234	0.056975	GTP-binding nuclear protein Ran
K13117	0.137708	0.023372	0.057294	ATP-dependent RNA helicase DDX35 EC:3.6.4.13
K13820	0.137682	0.023398	0.057337	flagellar biosynthetic protein FliR/FlhB
K14153	-0.13772	0.023432	0.057384	hydroxymethylpyrimidine kinase / phosphomethylpyrimidine kinase / thiamine-phosphate diphosphorylase EC:2.7.1.49 2.7.4.7 2.5.1.3
K02148	0.137647	0.023434	0.057384	V-type H+-transporting ATPase subunit C EC:3.6.3.14
K10216	0.137606	0.023476	0.057467	2-hydroxymuconate-semialdehyde hydrolase EC:3.7.1.9
K09818	0.137572	0.02351	0.05753	manganese/iron transport system substrate-binding protein
K09969	-0.13757	0.02358	0.05768	general L-amino acid transport system substrate-binding protein
K01567	-0.13756	0.02359	0.057683	pyruvate decarboxylase EC:4.1.1.1
K09770	0.13748	0.023672	0.057863	hypothetical protein; hypothetical protein
K06694	0.137339	0.023749	0.058033	26S proteasome non-ATPase regulatory subunit 10
K05346	-0.13739	0.023765	0.05805	deoxyribonucleoside regulator; deoxyribonucleoside regulator
K02507	-0.13735	0.023803	0.058124	protein transport protein HofQ
K11534	-0.13733	0.023825	0.058154	DeoR family transcriptional regulator, deoxyribose operon repressor
K01186	-0.13732	0.023834	0.058158	sialidase-1 EC:3.2.1.18
K13800	0.137176	0.023918	0.05834	UMP-CMP kinase EC:2.7.4.- 2.7.4.14
K01041	-0.13719	0.023974	0.058457	L-seryl-tRNA(Ser) seleniumtransferase EC:2.9.1.1
K02431	0.137169	0.023993	0.058482	L-fucose mutarotase EC:5.1.3.-
K05544	0.137085	0.024012	0.058509	tRNA-dihydrouridine synthase 3 EC:1.3.1.89
K15030	0.136945	0.024158	0.058844	translation initiation factor 3 subunit M
K02122	0.136926	0.024245	0.059034	V-type H+-transporting ATPase subunit F EC:3.6.3.14
K01049	0.136818	0.024291	0.059125	acetylcholinesterase EC:3.1.1.7
K14566	0.136725	0.024389	0.059343	U3 small nucleolar RNA-associated protein 24
K11438	0.136709	0.024405	0.059362	protein arginine N-methyltransferase 7 EC:2.1.1.-
K07488	-0.13676	0.024414	0.059362	transposase; transposase
K03453	-0.13669	0.024493	0.059534	bile acid_Na+ symporter, BASS family
K07199	0.136614	0.024505	0.059542	5'-AMP-activated protein kinase, regulatory beta subunit
K12466	0.136502	0.024624	0.059801	(+)-trans-carveol dehydrogenase EC:1.1.1.275
K05641	0.136497	0.024629	0.059801	ATP-binding cassette, subfamily A (ABC1), member 1
K04652	0.136528	0.024664	0.059865	hydrogenase nickel incorporation protein HypB

K01835	0.136463	0.024733	0.060001	phosphoglucomutase EC:5.4.2.2
K11450	0.136395	0.024738	0.060001	lysine-specific histone demethylase 1 EC:1.-.-.-
K02205	0.136291	0.024849	0.06025	arginine/ornithine permease
K00985	0.136331	0.024874	0.060289	RNA-directed RNA polymerase EC:2.7.7.48
K03816	0.13629	0.024918	0.060374	xanthine phosphoribosyltransferase EC:2.4.2.22
K06935	0.136215	0.02493	0.060383	23S rRNA (adenine2503-C2)-methyltransferase EC:2.1.1.192; 23S rRNA (adenine2503-C2)-methyltransferase EC:2.1.1.192
K13923	0.136132	0.025019	0.060578	phosphotransacylase
K00351	-0.13613	0.025086	0.060718	Na+-transporting NADH_ubiquinone oxidoreductase subunit F EC:1.6.5.-
K12555	0.13604	0.025186	0.060938	penicillin-binding protein 2A EC:2.4.1.129 2.3.2.-
K01166	0.135967	0.025198	0.060947	ribonuclease T2 EC:3.1.27.1
K00274	-0.13593	0.02524	0.061026	monoamine oxidase EC:1.4.3.4
K03582	-0.13592	0.025315	0.061187	exodeoxyribonuclease V beta subunit EC:3.1.11.5
K00209	0.135729	0.025457	0.061509	enoyl-acyl-carrier-protein reductase (NADPH2, B-specific) EC:1.3.1.10
K08252	0.135776	0.025472	0.061524	receptor protein-tyrosine kinase EC:2.7.10.1
K00088	0.135674	0.025517	0.06161	IMP dehydrogenase EC:1.1.1.205
K01106	0.135617	0.025579	0.061738	inositol-1,4,5-trisphosphate 5-phosphatase EC:3.1.3.56
K01856	-0.13563	0.025636	0.061842	muconate cycloisomerase EC:5.5.1.1
K03372	0.135562	0.02564	0.061842	MFS transporter, PAT family, solute carrier family 33 (acetyl-CoA transportor), member 1 EC:2.3.1.-
K00044	-0.13558	0.025689	0.061938	estradiol 17beta-dehydrogenase EC:1.1.1.62
K00086	0.135546	0.025724	0.061984	1,3-propanediol dehydrogenase EC:1.1.1.202
K00337	-0.13554	0.025726	0.061984	NADH-quinone oxidoreductase subunit H EC:1.6.5.3
K00437	0.135496	0.025779	0.06209	cytochrome-c3 hydrogenase EC:1.12.2.1
K08851	0.135336	0.025889	0.062334	TP53 regulating kinase EC:2.7.11.1
K09946	0.135251	0.02605	0.062699	hypothetical protein; hypothetical protein
K01755	-0.13519	0.026113	0.062808	argininosuccinate lyase EC:4.3.2.1
K13006	-0.13519	0.026113	0.062808	UDP-perosamine 4-acetyltransferase EC:2.3.1.-
K07458	0.135156	0.026156	0.062888	DNA mismatch endonuclease, patch repair protein EC:3.1.-.-
K00559	0.135057	0.0262	0.062903	sterol 24-C-methyltransferase EC:2.1.1.41
K00615	0.135134	0.02618	0.062903	transketolase EC:2.2.1.1
K00812	-0.13511	0.026207	0.062903	aspartate aminotransferase EC:2.6.1.1
K01989	0.135113	0.026203	0.062903	putative ABC transport system substrate-binding protein
K01769	0.13507	0.026185	0.062903	guanylate cyclase, other EC:4.6.1.2
K14457	0.134947	0.026323	0.063159	2-acylglycerol O-acyltransferase 2 EC:2.3.1.22
K03523	0.134978	0.026355	0.063214	putative biotin biosynthesis protein BioY
K07023	0.134917	0.026423	0.063355	putative hydrolases of HD superfamily; putative hydrolases of HD superfamily
K14847	0.134843	0.02644	0.063373	ribosome production factor 2
K09975	0.134691	0.026678	0.063923	hypothetical protein; hypothetical protein
K09973	-0.13465	0.026722	0.064005	hypothetical protein; hypothetical protein
K13481	0.134527	0.026799	0.064167	xanthine dehydrogenase small subunit EC:1.17.1.4
K08478	-0.13439	0.026959	0.064529	phosphoglycerate transport regulatory protein PgtC
K09272	0.134376	0.026972	0.064537	structure-specific recognition protein 1
K01135	-0.13433	0.027094	0.064807	arylsulfatase B EC:3.1.6.12
K00100	-0.1343	0.02713	0.06487	L-lactate dehydrogenase (cytochrome) EC:1.1.2.3
K13412	0.134226	0.027144	0.064881	calcium-dependent protein kinase EC:2.7.11.1
K02515	0.134184	0.027193	0.064975	serine/threonine-protein kinase HSL1,negative regulator of Swe1 kinase EC:2.7.11.-
K08766	0.134169	0.02721	0.064993	carnitine O-palmitoyltransferase 2 EC:2.3.1.21

K12287	0.134085	0.027308	0.065205	MSHA biogenesis protein MshQ
K00349	-0.1341	0.027355	0.065293	Na+-transporting NADH_ubiquinone oxidoreductase subunit D EC:1.6.5.-
K00244	-0.13407	0.027392	0.06531	fumarate reductase flavoprotein subunit EC:1.3.99.1
K04654	-0.13401	0.027399	0.06531	hydrogenase expression/formation protein HypD
K00922	0.134021	0.027383	0.06531	phosphatidylinositol-4,5-bisphosphate 3-kinase EC:2.7.1.153
K07179	0.134023	0.02738	0.06531	RIO kinase 2 EC:2.7.11.1
K12343	-0.13404	0.027422	0.065341	3-oxo-5-alpha-steroid 4-dehydrogenase 1 EC:1.3.99.5
K01301	-0.13402	0.027449	0.065383	glutamate carboxypeptidase II EC:3.4.17.21
K00887	0.133955	0.027459	0.065385	undecaprenol kinase EC:2.7.1.66
K13731	0.13392	0.0275	0.065459	internalin B
K06178	0.133948	0.027533	0.065492	23S rRNA pseudouridine2605 synthase EC:5.4.99.22
K03941	0.133897	0.027526	0.065492	NADH dehydrogenase (ubiquinone) Fe-S protein 8 EC:1.6.5.3 1.6.99.3
K01438	-0.13387	0.02762	0.065678	acetylornithine deacetylase EC:3.5.1.16
K12857	0.133791	0.027651	0.065729	Prp8 binding protein
K10643	0.13375	0.027699	0.06582	CCR4-NOT transcription complex subunit 4 EC:6.3.2.19
K02709	0.133637	0.027832	0.066112	photosystem II PsbH protein
K03415	0.133666	0.027864	0.066166	two-component system, chemotaxis family, response regulator CheV
K13921	-0.13359	0.027889	0.066204	1-propanol dehydrogenase
K01969	0.133572	0.027909	0.066227	3-methylcrotonyl-CoA carboxylase beta subunit EC:6.4.1.4
K07807	0.133473	0.028092	0.066638	hypothetical protein NreA; hypothetical protein NreA
K07231	-0.13346	0.028107	0.066651	putative iron-regulated protein; putative iron-regulated protein
K07318	0.13335	0.028238	0.066939	adenine-specific DNA-methyltransferase EC:2.1.1.72; adenine-specific DNA-methyltransferase EC:2.1.1.72
K05770	0.133151	0.02841	0.067325	benzodiazapine receptor
K14841	0.133139	0.028424	0.067334	ribosome biogenesis protein NSA1
K13566	0.133143	0.028484	0.067454	omega-amidase EC:3.5.1.3
K03806	-0.13313	0.028502	0.067472	AmpD protein
K01295	-0.1331	0.02854	0.06754	glutamate carboxypeptidase EC:3.4.17.11
K02510	0.133084	0.028556	0.067554	2,4-dihydroxyhept-2-ene-1,7-dioic acid aldolase EC:4.1.2.-
K07767	0.132858	0.028764	0.068023	microtubule-severing ATPase EC:3.6.4.3
K14051	0.132897	0.028782	0.068041	cyclic di-GMP phosphodiesterase Gmr EC:3.1.4.52
K11755	-0.13283	0.02886	0.068203	phosphoribosyl-ATP pyrophosphohydrolase / phosphoribosyl-AMP cyclohydrolase EC:3.6.1.31 3.5.4.19
K01422	-0.13279	0.028908	0.068283	L-asparaginase EC:3.5.1.1
K03826	0.132783	0.02892	0.068283	putative acetyltransferase EC:2.3.1.-
K11063	0.13278	0.028924	0.068283	toxin A/B
K07942	0.132686	0.028973	0.068376	ADP-ribosylation factor-like 1
K02650	0.132717	0.029001	0.068418	type IV pilus assembly protein PilA
K02759	-0.13266	0.029071	0.068561	PTS system, cellobiose-specific IIA component EC:2.7.1.69
K07502	0.132608	0.029133	0.068684	hypothetical protein; hypothetical protein
K02178	0.132541	0.02915	0.0687	checkpoint serine/threonine-protein kinase EC:2.7.11.1
K00712	-0.13257	0.029174	0.068734	poly(glycerol-phosphate) alpha-glucosyltransferase EC:2.4.1.52
K03328	-0.13255	0.029201	0.068773	polysaccharide transporter, PST family
K07048	0.132513	0.02925	0.068865	phosphotriesterase-related protein
K12142	0.132448	0.029264	0.068876	hydrogenase-4 component G EC:1.-.-.-
K01968	-0.13248	0.029288	0.068907	3-methylcrotonyl-CoA carboxylase alpha subunit EC:6.4.1.4

K14769	0.132417	0.029303	0.06892	U3 small nucleolar RNA-associated protein 11
K00390	0.132445	0.029334	0.068968	phosphoadenosine phosphosulfate reductase EC:1.8.4.8
K08775	0.132368	0.029363	0.069015	breast cancer 2 susceptibility protein
K05597	0.132343	0.029394	0.069063	glutamin-(asparagin-)ase EC:3.5.1.38
K12942	-0.13238	0.029408	0.069072	aminobenzoyl-glutamate transport protein
K03289	0.132226	0.029539	0.069356	MFS transporter, NHS family, nucleoside permease
K13606	0.132145	0.02964	0.069569	chlorophyll(ide) b reductase EC:1.1.1.294
K03075	-0.13207	0.029792	0.069903	preprotein translocase subunit SecG
K01636	0.13198	0.02991	0.070157	isocitrate lyase EC:4.1.3.1
K01955	-0.1319	0.030007	0.070331	carbamoyl-phosphate synthase large subunit EC:6.3.5.5
K02821	-0.13189	0.030025	0.070331	PTS system, ascorbate-specific IIA component EC:2.7.1.69
K04761	-0.1319	0.030009	0.070331	LysR family transcriptional regulator, hydrogen peroxide-inducible genes activator
K08496	0.131837	0.030026	0.070331	golgi SNAP receptor complex member 2
K12372	-0.13181	0.030062	0.070392	dipeptide transport system ATP-binding protein
K00167	-0.13177	0.030103	0.070466	2-oxoisovalerate dehydrogenase E1 component, beta subunit EC:1.2.4.4
K07964	0.131695	0.030205	0.070671	heparanase 1 EC:3.2.1.166
K12734	0.131689	0.030211	0.070671	peptidyl-prolyl cis-trans isomerase-like 3 EC:5.2.1.8
K06660	0.131636	0.030278	0.070803	cell cycle serine/threonine-protein kinase CDC5/MSD2 EC:2.7.11.21
K00340	-0.13163	0.030352	0.070952	NADH-quinone oxidoreductase subunit K EC:1.6.5.3
K00651	-0.13157	0.03043	0.07111	homoserine O-succinyltransferase EC:2.3.1.46
K01902	-0.1315	0.03051	0.071242	succinyl-CoA synthetase alpha subunit EC:6.2.1.5
K03769	0.131499	0.030518	0.071242	peptidyl-prolyl cis-trans isomerase C EC:5.2.1.8
K08956	0.131453	0.030512	0.071242	AFG3 family protein EC:3.4.24.-
K11198	-0.13142	0.030556	0.071309	PTS system, 2-O-A-mannosyl-D-glycerate-specific IIA component EC:2.7.1.69
K04424	0.131314	0.03069	0.071596	sterile alpha motif and leucine zipper containing kinase AZK EC:2.7.11.25
K12767	0.131249	0.030772	0.071764	serine/threonine-protein kinase RIM15 EC:2.7.11.1
K04508	0.131157	0.030891	0.072017	transducin (beta)-like 1
K14949	0.131097	0.030969	0.072175	serine/threonine-protein kinase PknG EC:2.7.11.1
K00012	-0.13095	0.031225	0.072747	UDPglucose 6-dehydrogenase EC:1.1.1.22
K03475	-0.13091	0.031272	0.07283	PTS system, ascorbate-specific IIC component
K08720	-0.13083	0.031315	0.072907	outer membrane protein OmpU
K00606	-0.13087	0.031328	0.072912	3-methyl-2-oxobutanoate hydroxymethyltransferase EC:2.1.2.11
K00039	0.130729	0.031448	0.073166	ribitol 2-dehydrogenase EC:1.1.1.56
K02618	0.130709	0.031474	0.073202	oxepin-CoA hydrolase / 3-oxo-5,6-dehydrosuberyl-CoA semialdehyde dehydrogenase EC:3.7.1.16 1.17.1.7
K00638	-0.13074	0.031499	0.073237	chloramphenicol O-acetyltransferase EC:2.3.1.28
K00330	-0.13068	0.031573	0.073358	NADH-quinone oxidoreductase subunit A EC:1.6.5.3
K01233	0.13064	0.031564	0.073358	chitosanase EC:3.2.1.132
K01798	0.130589	0.031632	0.07347	maleate isomerase EC:5.2.1.1
K01939	0.130596	0.031685	0.07357	adenylosuccinate synthase EC:6.3.4.4
K04744	-0.13055	0.03174	0.073668	LPS-assembly protein
K09703	0.130548	0.031749	0.073668	hypothetical protein; hypothetical protein
K09728	0.130403	0.031877	0.073941	hypothetical protein; hypothetical protein
K05833	0.130364	0.031991	0.07418	putative ABC transport system ATP-binding protein
K02781	-0.13026	0.032125	0.074465	PTS system, glucitol/sorbitol-specific IIA component EC:2.7.1.69
K07721	0.130152	0.03221	0.074639	ArsR family transcriptional regulator; ArsR family transcriptional regulator

K11473	-0.13006	0.032336	0.074905	glycolate oxidase iron-sulfur subunit
K02219	0.130037	0.032365	0.074946	cyclin-dependent kinase regulatory subunit CKS1
K03799	-0.13004	0.032424	0.075058	heat shock protein HtpX EC:3.4.24.-
K09888	-0.12996	0.032527	0.075271	cell division protein ZapA
K04028	-0.12985	0.032676	0.075575	ethanolamine utilization protein EutN
K07696	-0.12985	0.03268	0.075575	two-component system, NarL family, response regulator NreC
K05772	0.129833	0.032701	0.075599	tungstate transport system substrate-binding protein
K02422	0.12979	0.03276	0.075709	flagellar protein FliS
K08659	-0.1297	0.032886	0.075974	dipeptidase EC:3.4.-.-
K09740	0.129547	0.033027	0.076276	hypothetical protein; hypothetical protein
K03533	-0.12955	0.033082	0.076376	TorA specific chaperone
K00684	-0.12943	0.033245	0.076701	leucyl/phenylalanyl-tRNA--protein transferase EC:2.3.2.6
K10755	0.129395	0.033235	0.076701	replication factor C subunit 2/4
K01703	-0.1294	0.033289	0.076751	3-isopropylmalate/(R)-2-methylmalate dehydratase large subunit EC:4.2.1.33 4.2.1.35
K06149	-0.1294	0.033287	0.076751	universal stress protein A
K10547	0.129344	0.033368	0.076909	putative multiple sugar transport system permease protein
K05568	0.129315	0.033408	0.076975	multicomponent Na+_H+ antiporter subunit D
K00350	-0.1292	0.033573	0.077328	Na+-transporting NADH_ubiquinone oxidoreductase subunit E EC:1.6.5.-
K02407	0.129123	0.033611	0.07738	flagellar hook-associated protein 2
K12265	-0.12912	0.033618	0.07738	nitric oxide reductase FlRd-NAD(+) reductase EC:1.18.1.-
K03276	-0.12911	0.033632	0.077388	UDP-glucose/galactose_(glucosyl)LPS alpha-1,2-glucosyl/galactosyltransferase EC:2.4.1.-
K10094	-0.12909	0.033658	0.077396	nickel transport system substrate-binding protein; nickel transport system substrate-binding protein
K12547	-0.12914	0.033651	0.077396	polysaccharidase protein
K03593	-0.1291	0.0337	0.077454	ATP-binding protein involved in chromosome partitioning
K05832	0.129099	0.033706	0.077454	putative ABC transport system permease protein
K00839	0.129068	0.033749	0.077494	aminotransferase EC:2.6.1.-
K01906	-0.12907	0.033744	0.077494	6-carboxyhexanoate--CoA ligase EC:6.2.1.14
K12813	0.129018	0.033757	0.077494	pre-mRNA-splicing factor ATP-dependent RNA helicase DHX16 EC:3.6.4.13
K08223	-0.12899	0.033852	0.077687	MFS transporter, FSR family, fosmidomycin resistance protein
K00050	0.128925	0.033948	0.077881	hydroxypyruvate reductase EC:1.1.1.81
K08876	0.12885	0.03399	0.077952	SCY1-like
K10677	-0.12879	0.034139	0.078268	inulin fructotransferase (DFA-I-forming) EC:4.2.2.17
K03152	-0.12873	0.034216	0.078418	4-methyl-5(b-hydroxyethyl)-thiazole monophosphate biosynthesis
K05275	-0.1287	0.034256	0.078483	pyridoxine 4-dehydrogenase EC:1.1.1.65
K04461	0.128648	0.034275	0.0785	protein phosphatase 1B (formerly 2C) EC:3.1.3.16
K00789	0.128563	0.034456	0.078888	S-adenosylmethionine synthetase EC:2.5.1.6
K01007	-0.12854	0.034491	0.078944	pyruvate, water dikinase EC:2.7.9.2
K13288	-0.12852	0.034517	0.078976	oligoribonuclease EC:3.1.-.-
K03382	0.128492	0.034555	0.079012	hydroxyatrazine ethylaminohydrolase EC:3.5.99.3
K12987	0.128494	0.034553	0.079012	alpha-1,6-rhamnosyltransferase EC:2.4.1.-
K00344	0.12841	0.034672	0.079251	NADPH2_quinone reductase EC:1.6.5.5
K00645	-0.12838	0.034715	0.079303	acyl-carrier-protein S-malonyltransferase EC:2.3.1.39
K14521	0.128335	0.034717	0.079303	N-acetyltransferase 10 EC:2.3.1.-
K12988	0.128348	0.03476	0.079374	alpha-1,3-rhamnosyltransferase EC:2.4.1.-
K03817	-0.12834	0.034775	0.079383	ribosomal-protein-serine acetyltransferase EC:2.3.1.-

K02149	0.128285	0.034789	0.079387	V-type H+-transporting ATPase subunit D EC:3.6.3.14
K00764	-0.12831	0.034812	0.079415	amidophosphoribosyltransferase EC:2.4.2.14
K08280	-0.1283	0.034835	0.079441	lipopolysaccharide O-acetyltransferase EC:2.3.1.-
K03391	0.128225	0.034874	0.079503	pentachlorophenol monooxygenase EC:1.14.13.50
K07334	-0.12825	0.034901	0.079538	proteic killer suppression protein
K09004	-0.12815	0.034987	0.079709	hypothetical protein; hypothetical protein
K12293	0.128137	0.035	0.079712	competence factor transport accessory protein ComB
K01461	-0.12803	0.035155	0.080011	N-acyl-D-glutamate deacylase EC:3.5.1.82
K01499	0.128037	0.035145	0.080011	methenyltetrahydromethanopterin cyclohydrolase EC:3.5.4.27
K03790	0.128031	0.035213	0.080118	ribosomal-protein-alanine N-acetyltransferase EC:2.3.1.128
K02051	0.127865	0.035453	0.080638	NitT/TauT family transport system substrate-binding protein
K03250	0.127777	0.03552	0.080763	translation initiation factor 3 subunit E
K04477	-0.12776	0.035609	0.080938	putative hydrolase
K02414	0.127727	0.035653	0.080986	flagellar hook-length control protein FliK
K02418	0.127727	0.035654	0.080986	flagellar protein FliO/FliZ
K02073	0.127684	0.035716	0.081101	D-methionine transport system substrate-binding protein
K12267	-0.12764	0.035783	0.081226	peptide methionine sulfoxide reductase msrA/msrB EC:1.8.4.11 1.8.4.12
K14196	-0.12759	0.035796	0.08123	immunoglobulin G-binding protein A
K03770	-0.1276	0.035832	0.081284	peptidyl-prolyl cis-trans isomerase D EC:5.2.1.8
K14728	0.127533	0.035876	0.081357	phthiodiolone/phenolphthiodiolone dimycocerosates ketoreductase EC:1.2.-.-; phthiodiolone/phenolphthiodiolone dimycocerosates ketoreductase EC:1.2.-.-
K13179	0.127524	0.035889	0.08136	ATP-dependent RNA helicase DDX18/HAS1 EC:3.6.4.13
K00998	-0.12746	0.036047	0.081665	phosphatidylserine synthase EC:2.7.8.8
K03079	-0.12746	0.036045	0.081665	L-ribulose-5-phosphate 3-epimerase EC:5.1.3.22
K04115	0.127445	0.036065	0.081678	benzoyl-CoA reductase subunit EC:1.3.7.8
K03267	0.127366	0.036121	0.08178	peptide chain release factor subunit 3
K01938	0.127326	0.03624	0.082022	formate--tetrahydrofolate ligase EC:6.3.4.3
K02335	0.127302	0.036275	0.082074	DNA polymerase I EC:2.7.7.7
K14980	0.127244	0.036301	0.082105	two-component system, OmpR family, sensor histidine kinase ChvG EC:2.7.13.3
K01797	0.127235	0.036375	0.082245	maleate isomerase EC:5.2.1.1
K07803	-0.12716	0.036428	0.082339	zinc resistance-associated protein
K02246	0.127102	0.036511	0.0825	competence protein ComGD
K13626	0.127064	0.036628	0.082736	flagellar assembly factor FliW
K08287	0.126967	0.036713	0.082902	dual-specificity kinase EC:2.7.12.1
K03520	0.126947	0.036744	0.082943	carbon-monoxide dehydrogenase large subunit EC:1.2.99.2
K02230	-0.12696	0.036777	0.082992	cobaltochelatase CobN EC:6.6.1.2
K00286	-0.12692	0.036847	0.083123	pyrroline-5-carboxylate reductase EC:1.5.1.2
K13602	0.126865	0.036866	0.083138	bacteriochlorophyll C12 methyltransferase EC:2.1.1.-
K02119	0.126831	0.036975	0.083357	V-type H+-transporting ATPase subunit C EC:3.6.3.14
K01855	0.126772	0.037005	0.083398	tRNA pseudouridine38/39 synthase EC:5.4.99.45
K12681	-0.12672	0.037142	0.083678	pertactin
K01618	0.1267	0.037173	0.083721	deoxyribose-phosphate aldolase EC:4.1.2.4
K10599	0.126493	0.037426	0.084264	pre-mRNA-processing factor 19 EC:6.3.2.19
K10036	-0.1263	0.037781	0.085034	glutamine transport system substrate-binding protein
K05846	-0.12628	0.037805	0.08506	osmoprotectant transport system permease protein
K09795	-0.12622	0.037849	0.085131	hypothetical protein; hypothetical protein

K00808	0.12619	0.037889	0.085194	homospermidine synthase EC:2.5.1.44
K00967	0.126113	0.038008	0.085434	ethanolamine-phosphate cytidylyltransferase EC:2.7.7.14
K03563	0.125995	0.03819	0.085787	carbon storage regulator
K00710	0.125996	0.038187	0.085787	polypeptide N-acetylgalactosaminyltransferase EC:2.4.1.41
K00302	0.125979	0.038272	0.085833	sarcosine oxidase, subunit alpha EC:1.5.3.1
K00919	0.125981	0.038268	0.085833	4-diphosphocytidyl-2-C-methyl-D-erythritol kinase EC:2.7.1.148
K08490	0.125951	0.038258	0.085833	syntaxin 5
K09725	0.125952	0.038255	0.085833	hypothetical protein; hypothetical protein
K06666	0.12596	0.038244	0.085833	glucose repression regulatory protein TUP1
K03564	-0.12596	0.038298	0.085863	peroxiredoxin Q/BCP EC:1.11.1.15
K08152	-0.12587	0.038432	0.086136	MFS transporter, DHA1 family, multidrug resistance protein B
K01789	0.125798	0.038494	0.086183	
K03183	0.125837	0.03849	0.086183	ubiquinone/menaquinone biosynthesis methyltransferase EC:2.1.1.163 2.1.1.201
K03793	0.125792	0.038503	0.086183	pteridine reductase EC:1.5.1.33
K00192	0.125806	0.038482	0.086183	acetyl-CoA decarbonylase/synthase complex subunit alpha EC:1.2.99.2
K11189	0.125818	0.03852	0.086193	phosphocarrier protein
K03797	-0.12578	0.038587	0.086314	carboxyl-terminal processing protease EC:3.4.21.102
K09712	-0.12572	0.038615	0.086349	hypothetical protein; hypothetical protein
K07168	-0.12565	0.038729	0.086575	CBS domain-containing membrane protein; CBS domain-containing membrane protein
K03589	0.125607	0.03885	0.086818	cell division protein FtsQ
K07340	0.125549	0.038883	0.086864	hypothetical protein; hypothetical protein
K07102	-0.12554	0.038904	0.086882	N-acetylmuramic acid 6-phosphate etherase EC:4.2.1.126; N-acetylmuramic acid 6-phosphate etherase EC:4.2.1.126
K07022	0.125496	0.038966	0.086993	putative hydrolases of HD superfamily; putative hydrolases of HD superfamily
K12544	0.125392	0.03913	0.08733	S-layer protein
K07461	0.125362	0.039177	0.087382	putative endonuclease; putative endonuclease
K11907	0.125361	0.039178	0.087382	type VI secretion system protein VasG
K08869	0.125333	0.039223	0.087454	aarF domain-containing kinase
K02248	0.12525	0.039355	0.087719	competence protein ComGF
K00957	-0.12525	0.039407	0.087808	sulfate adenylyltransferase subunit 2 EC:2.7.7.4
K07288	0.12512	0.03956	0.08812	uncharacterized membrane protein
K02815	-0.12511	0.039584	0.088144	PTS system, sorbose-specific IID component
K11498	0.125079	0.039626	0.088209	centromeric protein E
K10556	-0.12505	0.039723	0.088398	AI-2 transport system permease protein
K01849	-0.12504	0.039744	0.088416	methylmalonyl-CoA mutase, C-terminal domain EC:5.4.99.2
K11754	0.124968	0.039859	0.088642	dihydrofolate synthase / folylpolyglutamate synthase EC:6.3.2.12 6.3.2.17
K01845	0.124863	0.040028	0.088961	glutamate-1-semialdehyde 2,1-aminomutase EC:5.4.3.8
K14659	0.124835	0.040016	0.088961	chitooligosaccharide deacetylase EC:3.5.1.-
K00620	0.12484	0.040065	0.089014	glutamate N-acetyltransferase / amino-acid N-acetyltransferase EC:2.3.1.35 2.3.1.1
K13747	-0.12458	0.040475	0.089898	carboxynorspermidine decarboxylase EC:4.1.1.-
K02208	0.124421	0.040686	0.090336	cyclin-dependent kinase 8/11 EC:2.7.11.22 2.7.11.23
K01533	0.124424	0.040737	0.09042	Cu2+-exporting ATPase EC:3.6.3.4
K03240	0.12437	0.040768	0.090461	translation initiation factor eIF-2B subunit epsilon
K12283	-0.12434	0.040826	0.090559	MSHA biogenesis protein MshM

K10125	-0.12428	0.040966	0.090841	two-component system, NtrC family, C4-dicarboxylate transport sensor histidine kinase DctB EC:2.7.13.3
K08589	0.124233	0.040994	0.090873	gingipain R EC:3.4.22.37
K10117	0.124188	0.041121	0.091126	multiple sugar transport system substrate-binding protein
K01304	0.12412	0.041233	0.091323	pyroglutamyl-peptidase EC:3.4.19.3
K06968	-0.12408	0.041236	0.091323	23S rRNA (cytidine2498-2'-O)-methyltransferase EC:2.1.1.186
K06119	0.124076	0.041251	0.091327	sulfoquinovosyltransferase EC:2.4.1.-
K09472	-0.12406	0.041281	0.091335	gamma-glutamyl-gamma-aminobutyraldehyde dehydrogenase EC:1.2.1.-
K06316	0.124064	0.041271	0.091335	oligosaccharidyl-lipid flippase family
K05773	0.124002	0.041427	0.091628	tungstate transport system permease protein
K07571	0.123992	0.041443	0.091634	S1 RNA binding domain protein; S1 RNA binding domain protein
K07039	-0.12396	0.041491	0.091681	uncharacterized protein; uncharacterized protein
K09531	0.123935	0.041484	0.091681	DnaJ homolog subfamily C member 11
K00162	0.123909	0.041581	0.09185	pyruvate dehydrogenase E1 component subunit beta EC:1.2.4.1
K13108	0.123777	0.041746	0.092186	smad nuclear-interacting protein 1
K00441	-0.12364	0.042031	0.092785	coenzyme F420 hydrogenase beta subunit EC:1.12.98.1
K14727	0.123625	0.042054	0.092807	3-oxoadipate enol-lactonase / 4-carboxymuconolactone decarboxylase EC:3.1.1.24 4.1.1.44
K07149	0.123601	0.042094	0.092866	dolichyl-diphosphooligosaccharide--protein glycosyltransferase EC:2.4.1.119; dolichyl-diphosphooligosaccharide--protein glycosyltransferase EC:2.4.1.119
K01250	0.123582	0.042125	0.092903	pyrimidine-specific ribonucleoside hydrolase EC:3.2.-.-
K13829	0.12352	0.042175	0.092985	shikimate kinase / 3-dehydroquinate synthase EC:2.7.1.71 4.2.3.4
K11833	0.123498	0.042213	0.093038	ubiquitin carboxyl-terminal hydrolase 2/21 EC:3.1.2.15
K07101	-0.12339	0.042455	0.093512	N-acetylmuramic acid 6-phosphate etherase EC:4.2.1.126; N-acetylmuramic acid 6-phosphate etherase EC:4.2.1.126
K07298	0.123354	0.042455	0.093512	serine/threonine-protein kinase 11 EC:2.7.11.9
K02535	-0.12335	0.042508	0.093598	UDP-3-O-3-hydroxymyristoyl N-acetylglucosamine deacetylase EC:3.5.1.108
K05658	0.123324	0.042558	0.09365	ATP-binding cassette, subfamily B (MDR/TAP), member 1
K07071	-0.12333	0.042554	0.09365	UPF0755 protein; UPF0755 protein
K09964	-0.12321	0.042693	0.093917	hypothetical protein; hypothetical protein
K04770	-0.12317	0.042764	0.094013	Lon-like ATP-dependent protease EC:3.4.21.-
K01526	0.123172	0.042764	0.094013	guanosine-diphosphatase EC:3.6.1.42
K11249	0.1231	0.04294	0.094368	cysteine/O-acetylserine efflux protein
K13684	0.123091	0.042954	0.09437	putative colanic acid biosynthesis glycosyltransferase EC:2.4.-.-
K11069	0.123077	0.042978	0.094394	spermidine/putrescine transport system substrate-binding protein
K00123	0.123024	0.043069	0.094563	formate dehydrogenase, alpha subunit EC:1.2.1.2
K12247	-0.12295	0.043147	0.094703	alpha-2,3 sialyltransferase EC:2.4.99.-
K00766	-0.12294	0.043218	0.0948	anthranilate phosphoribosyltransferase EC:2.4.2.18
K09936	0.122939	0.043214	0.0948	hypothetical protein; hypothetical protein
K11991	0.122893	0.043239	0.094815	tRNA-specific adenosine deaminase EC:3.5.4.-
K10001	-0.12288	0.043255	0.094821	glutamate/aspartate transport system substrate-binding protein
K00650	0.122871	0.043277	0.094838	lecithin-cholesterol acyltransferase EC:2.3.1.43
K06956	-0.12288	0.043312	0.094885	tRNA(Met) cytidine acetyltransferase EC:2.3.1.193; tRNA(Met) cytidine acetyltransferase EC:2.3.1.193

K02080	-0.12277	0.04345	0.095157	galactosamine-6-phosphate isomerase EC:5.3.1.-
K00151	0.122761	0.043465	0.095161	5-carboxymethyl-2-hydroxymuconic-semialdehyde dehydrogenase EC:1.2.1.60
K10532	-0.12267	0.043672	0.095582	heparan-alpha-glucosaminide N-acetyltransferase EC:2.3.1.78
K06154	0.122606	0.043734	0.095687	Lrp/AsnC family transcriptional regulator, involved in the regulation of lysine biosynthesis
K07482	0.122627	0.043749	0.09569	transposase, IS30 family; transposase, IS30 family
K13537	0.12258	0.043778	0.095723	cysteine peptidase B EC:3.4.22.-
K09528	0.122565	0.043804	0.09575	DnaJ homolog subfamily C member 8
K12944	0.12255	0.04383	0.095777	nucleoside triphosphatase EC:3.6.1.-
K00518	-0.12246	0.043986	0.096088	superoxide dismutase EC:1.15.1.1
K01079	-0.12247	0.044027	0.096146	phosphoserine phosphatase EC:3.1.3.3
K10188	0.122454	0.044049	0.096163	lactose/L-arabinose transport system substrate-binding protein
K08975	0.122352	0.044176	0.09641	putative membrane protein; putative membrane protein
K12978	-0.12226	0.04433	0.096717	lipid A 4'-phosphatase EC:3.1.3.-
K02452	-0.12222	0.044402	0.096843	general secretion pathway protein C
K00797	0.122229	0.044442	0.096899	spermidine synthase EC:2.5.1.16
K11185	0.122184	0.044469	0.096928	cytosolic tryparedoxin peroxidase, trypanosomatid typical 2-Cys peroxiredoxin EC:1.11.1.15
K08651	-0.12217	0.04449	0.096943	thermitase EC:3.4.21.66
K00376	0.122148	0.044532	0.097004	nitrous-oxide reductase EC:1.7.2.4
K01366	0.122114	0.044593	0.097106	cathepsin H EC:3.4.22.16
K10907	0.122082	0.0447	0.097309	aminotransferase EC:2.6.1.-; aminotransferase EC:2.6.1.-
K08239	0.122038	0.044778	0.097447	UDP-N-acetylglucosamine-lysosomal-enzyme EC:2.7.8.17
K13995	-0.12194	0.044906	0.097694	maleamate amidohydrolase EC:3.5.1.107
K06518	-0.1219	0.045016	0.097904	holin-like protein
K02865	0.121845	0.045068	0.097954	large subunit ribosomal protein L10Ae
K10771	0.121849	0.045061	0.097954	AP endonuclease 1 EC:4.2.99.18
K01232	-0.12183	0.045145	0.098092	maltose-6'-phosphate glucosidase EC:3.2.1.122
K02603	0.121739	0.045257	0.098303	origin recognition complex subunit 1
K03199	-0.12174	0.045296	0.098311	type IV secretion system protein VirB4
K02728	0.121717	0.045295	0.098311	20S proteasome subunit alpha 3 EC:3.4.25.1
K14156	0.121713	0.045303	0.098311	choline/ethanolamine kinase EC:2.7.1.32 2.7.1.82
K11426	0.121664	0.04539	0.09847	SET and MYND domain-containing protein
K07667	0.121631	0.0455	0.098675	two-component system, OmpR family, KDP operon response regulator KdpE
K07343	0.12156	0.045577	0.098811	DNA transformation protein and related proteins
K05993	0.121579	0.045591	0.098812	
K12580	0.1215	0.045683	0.09898	CCR4-NOT transcription complex subunit 3
K07406	0.121425	0.045869	0.099352	alpha-galactosidase EC:3.2.1.22
K00076	0.121292	0.046108	0.099828	7-alpha-hydroxysteroid dehydrogenase EC:1.1.1.159
K01195	-0.12129	0.046118	0.099828	beta-glucuronidase EC:3.2.1.31
K08300	-0.12107	0.046512	0.10065	ribonuclease E EC:3.1.26.12
K00702	0.121039	0.046567	0.100689	cellobiose phosphorylase EC:2.4.1.20
K01434	0.121022	0.046548	0.100689	penicillin amidase EC:3.5.1.11
K02315	0.121035	0.046574	0.100689	DNA replication protein DnaC
K01160	0.120981	0.046623	0.100713	crossover junction endodeoxyribonuclease RusA EC:3.1.22.4
K03062	0.12097	0.046643	0.100713	26S proteasome regulatory subunit T2
K14573	0.120972	0.04664	0.100713	nucleolar protein 4
K13617	0.120982	0.046621	0.100713	protein phosphatase methylesterase 1 EC:3.1.1.89

K02195	-0.12076	0.047078	0.101619	heme exporter protein C
K09807	0.120701	0.047185	0.10182	hypothetical protein; hypothetical protein
K07486	0.120688	0.047209	0.101838	transposase; transposase
K01784	-0.12067	0.047234	0.101861	UDP-glucose 4-epimerase EC:5.1.3.2
K06043	0.120635	0.047258	0.10188	
K10866	0.120635	0.047306	0.101953	DNA repair protein RAD50 EC:3.6.-.-
K10730	0.120526	0.04746	0.102252	ATP-dependent DNA helicase Q4 EC:3.6.4.12
K07760	0.120478	0.047549	0.102413	cyclin-dependent kinase EC:2.7.11.22
K09144	0.12047	0.047612	0.102516	hypothetical protein; hypothetical protein
K13788	-0.12045	0.047658	0.102583	phosphate acetyltransferase EC:2.3.1.8
K06714	-0.12038	0.047732	0.10271	arginine utilization regulatory protein
K12382	0.120341	0.047804	0.102833	saposin
K00796	-0.12035	0.047834	0.102865	dihydropteroate synthase EC:2.5.1.15
K04780	0.120258	0.048007	0.103206	nonribosomal peptide synthetase DhbF
K01076	0.120204	0.048107	0.103389	alkaline phosphatase EC:3.1.3.1
K07132	0.120192	0.04813	0.103405	molybdenum cofactor cytidylyltransferase EC:2.7.7.76; molybdenum cofactor cytidylyltransferase EC:2.7.7.76
K02686	-0.12013	0.048192	0.103506	primosomal replication protein N
K08687	-0.12003	0.048389	0.103897	N-carbamoylsarcosine amidase EC:3.5.1.59
K11624	0.120017	0.048412	0.103915	two-component system, NarL family, response regulator YdfI
K01486	0.119947	0.04859	0.104264	adenine deaminase EC:3.5.4.2
K01709	-0.11988	0.048713	0.104495	CDP-glucose 4,6-dehydratase EC:4.2.1.45
K11930	-0.11982	0.048785	0.104618	periplasmic protein TorT
K03185	-0.1198	0.048826	0.104673	2-octaprenyl-6-methoxyphenol hydroxylase EC:1.14.13.-
K01071	0.119793	0.048882	0.104728	oleoyl-acyl-carrier-protein hydrolase EC:3.1.2.14
K11230	0.119776	0.048867	0.104728	mitogen-activated protein kinase kinase kinase EC:2.7.11.25
K04778	0.119755	0.048908	0.104751	vibriobactin synthetase
K00041	-0.11975	0.048969	0.104816	tagaturonate reductase EC:1.1.1.58
K08161	-0.11975	0.048956	0.104816	MFS transporter, DHA1 family, multidrug resistance protein
K05798	-0.11965	0.049102	0.10507	LysR family transcriptional regulator, transcriptional activator for leuABCD operon
K07448	0.119651	0.049152	0.105144	restriction system protein
K10947	0.119631	0.049189	0.10519	PadR family transcriptional regulator, regulatory protein PadR
K12945	-0.1195	0.049388	0.105583	GDP-mannose pyrophosphatase NudK EC:3.6.1.-
K12603	0.119424	0.04954	0.105874	CCR4-NOT transcription complex subunit 6
K09736	0.119267	0.049843	0.106488	hypothetical protein; hypothetical protein
K03295	-0.11926	0.049898	0.106574	cation efflux system protein, CDF family
K07204	0.119229	0.049915	0.106578	regulatory associated protein of mTOR
K06210	0.119187	0.049997	0.106718	nicotinamide mononucleotide adenylyltransferase EC:2.7.7.1 2.7.7.18
K09882	0.119202	0.050013	0.106721	
K03302	-0.11919	0.050044	0.106752	
K01246	-0.11913	0.050161	0.106905	
K12858	0.119105	0.050156	0.106905	
K12833	0.119106	0.050155	0.106905	
K11071	0.119024	0.050358	0.107291	
K08337	0.118937	0.050482	0.107522	
K07005	-0.11893	0.050542	0.107617	
K01695	-0.1189	0.050602	0.107711	

K01924	0.118852	0.050694	0.107873
K04785	-0.11881	0.050723	0.107901
K09808	-0.11879	0.050812	0.108058
K02203	0.118769	0.050857	0.10812
K10003	-0.11873	0.050895	0.108135
K12152	0.118732	0.050884	0.108135
K01372	-0.11872	0.050958	0.108183
K07275	-0.11869	0.050965	0.108183
K01406	0.118701	0.050945	0.108183
K02380	-0.11863	0.051138	0.108516
K06287	0.118561	0.051265	0.108753
K07185	0.11841	0.051563	0.109352
K00691	-0.11839	0.0516	0.109397
K07405	-0.11832	0.051737	0.109652
K05878	-0.1182	0.051972	0.110117
K13276	-0.11814	0.052102	0.110359
K09859	0.118099	0.052141	0.110407
K02346	0.118096	0.05219	0.110478
K07654	0.118064	0.052254	0.11058
K07729	0.117922	0.052539	0.111148
K05816	-0.11784	0.052705	0.111466
K06891	-0.1178	0.052793	0.111617
K03636	-0.11775	0.052838	0.111677
K08829	0.117717	0.052912	0.111774
K09313	0.117715	0.052916	0.111774
K03818	-0.11759	0.053178	0.112294
K04109	0.117574	0.053203	0.112312
K02237	0.11752	0.053353	0.112594
K01581	-0.11748	0.053439	0.112741
K12598	0.117454	0.053488	0.11281
K10004	-0.11735	0.053666	0.113151
K02900	0.1173	0.053763	0.11332
K03252	0.117234	0.053898	0.113571
K12941	-0.11724	0.053933	0.113611
K14561	0.117208	0.053953	0.113616
K09826	0.117192	0.053985	0.11365
K08843	0.117152	0.054069	0.113792
K00462	-0.11714	0.054141	0.113909
K02822	-0.11711	0.054202	0.114002
K12246	-0.11708	0.05422	0.114006
K00262	-0.11696	0.054503	0.114566
K01897	0.116948	0.054532	0.114591
K05885	-0.11687	0.054687	0.114883
K05884	0.11677	0.054902	0.1153
K09137	0.116685	0.05504	0.115554
K03930	0.116597	0.055265	0.11599
K08965	0.116549	0.055365	0.116165
K04409	0.116455	0.055524	0.116463
K02075	-0.11643	0.055606	0.116565
K02342	-0.11644	0.0556	0.116565
K02198	-0.11641	0.05565	0.116621
K00183	-0.11636	0.055725	0.116707

K02727	0.116361	0.055723	0.116707	
K05591	-0.11632	0.05584	0.116913	
K01791	-0.11624	0.056022	0.11726	
K00273	0.116219	0.056063	0.117266	
K03117	-0.11621	0.056076	0.117266	
K03380	-0.11621	0.056049	0.117266	
K00331	-0.11615	0.056215	0.117519	
K04077	0.11614	0.056232	0.117519	
K06323	0.116086	0.056308	0.117643	
K11070	0.116062	0.056397	0.117795	
K10865	0.11602	0.056449	0.117866	
K11400	0.115854	0.056806	0.118575	
K13918	-0.11583	0.056866	0.118666	
K02293	0.115761	0.057007	0.118924	
K01029	0.115768	0.057029	0.118934	
K03768	0.115683	0.057212	0.11928	
K13573	0.115663	0.057255	0.119334	
K08741	0.115603	0.057348	0.119491	
K14682	0.115556	0.057451	0.119669	
K06215	0.115458	0.057699	0.120149	
K02516	0.115405	0.05778	0.120282	
K00335	0.1154	0.057825	0.120339	
K14084	0.115339	0.057923	0.120508	
K06603	0.11532	0.058	0.120631	
K06610	0.115305	0.058033	0.120663	
K00700	0.11529	0.058067	0.120698	
K03732	0.115258	0.058102	0.120734	
K09798	-0.11524	0.058178	0.120856	
K05942	0.115213	0.058199	0.120864	
K01208	0.115093	0.058499	0.121449	
K04794	0.11499	0.058726	0.121884	
K06889	-0.11497	0.058767	0.121933	
K07267	0.114829	0.059048	0.122479	
K13059	-0.11483	0.059088	0.122524	
K00248	0.114791	0.059167	0.122651	
K10243	0.114734	0.059259	0.122733	
K11177	0.114736	0.059255	0.122733	
K00234	0.114734	0.059259	0.122733	
K06720	-0.11471	0.059304	0.122787	
K01556	0.1147	0.059336	0.122817	
K01818	-0.11468	0.059378	0.122868	
K07765	0.114656	0.059433	0.122945	
K00524	0.114626	0.0595	0.123046	
K06176	0.114557	0.059654	0.123329	
K02552	0.114465	0.059894	0.123787	
K04562	0.114426	0.059984	0.123935	
K07034	0.114396	0.060017	0.123967	
K01561	0.114294	0.060247	0.124294	
K01919	0.114309	0.060246	0.124294	
K04565	0.114313	0.060237	0.124294	
K07138	0.114309	0.060247	0.124294	
K07169	0.114252	0.060343	0.124454	

K13339	0.11414	0.060597	0.12494
K03339	0.114128	0.060623	0.124956
K06923	0.114105	0.060707	0.125092
K12989	0.113838	0.061285	0.126246
K04015	-0.11383	0.061338	0.126317
K07953	0.113609	0.061812	0.127256
K01175	0.113587	0.061892	0.127382
K01704	-0.11355	0.061985	0.127536
K01529	-0.11352	0.062051	0.127589
K03142	-0.11351	0.06204	0.127589
K15108	0.113499	0.062066	0.127589
K00965	0.113483	0.062134	0.127691
K12541	0.113446	0.062189	0.127766
K00161	0.113429	0.062259	0.12787
K12986	0.113356	0.062426	0.128177
K05954	0.113335	0.062446	0.128178
K01976	0.113267	0.062634	0.128526
K02392	0.1131	0.063024	0.129288
K01911	-0.11308	0.063071	0.129345
K07043	0.113028	0.063165	0.129488
K08966	-0.11302	0.063178	0.129488
K04014	-0.11298	0.063304	0.129708
K09660	0.112829	0.063634	0.130346
K08483	0.112831	0.063656	0.130353
K06175	-0.1128	0.063696	0.130396
K01524	-0.11279	0.063764	0.130497
K00038	0.112723	0.063913	0.130762
K06632	0.11268	0.063985	0.130871
K06963	0.112651	0.064056	0.130978
K00633	-0.11259	0.064235	0.131304
K03583	-0.11258	0.064259	0.131315
K02804	0.112552	0.064291	0.13134
K07147	-0.11254	0.064355	0.131433
K14764	0.112516	0.064377	0.131439
K10778	-0.11251	0.064424	0.131496
K09461	0.112488	0.064444	0.131498
K11535	-0.11241	0.064618	0.131814
K03224	-0.11239	0.064677	0.131896
K07636	-0.11222	0.065114	0.132748
K07320	-0.1122	0.065171	0.132824
K13124	0.112106	0.065362	0.133173
K05589	-0.11205	0.065511	0.133439
K01721	0.112024	0.065559	0.133497
K01729	-0.11188	0.065901	0.134153
K01681	-0.11183	0.066051	0.134419
K03410	0.111816	0.06609	0.13446
K11274	0.111746	0.066235	0.134714
K02314	0.111712	0.066343	0.134895
K13874	-0.11164	0.066507	0.135187
K05992	0.111608	0.066573	0.135282
K03412	0.111587	0.066649	0.135359
K06228	-0.11159	0.06665	0.135359

K14056	0.111543	0.066758	0.135538
K11843	0.111525	0.066779	0.13554
K03706	0.111442	0.067007	0.135924
K04084	0.111448	0.066991	0.135924
K13665	0.111399	0.067112	0.136098
K11477	-0.11135	0.067216	0.136268
K02436	-0.11135	0.067242	0.13628
K03752	-0.11133	0.067291	0.13634
K04649	0.111279	0.067383	0.136487
K03481	-0.11127	0.06744	0.136562
K12856	0.111073	0.067896	0.137444
K11740	-0.11099	0.068098	0.137813
K01802	-0.11099	0.068126	0.13783
K01710	-0.11081	0.06857	0.138687
K00849	0.110735	0.068765	0.139041
K00889	0.11063	0.069009	0.139452
K10751	0.110631	0.069006	0.139452
K07028	0.110537	0.069266	0.13993
K01903	-0.11052	0.06931	0.139979
K03739	0.110505	0.069347	0.140012
K02027	0.110432	0.06953	0.140341
K01400	0.110414	0.069577	0.140396
K10798	0.110361	0.069691	0.140584
K00175	-0.11028	0.069926	0.141016
K14811	0.110234	0.070015	0.141155
K02394	-0.11022	0.070062	0.141207
K03011	0.11017	0.070178	0.141391
K07952	0.110164	0.070194	0.141391
K06306	0.110099	0.070379	0.141723
K00423	-0.11004	0.070511	0.141948
K10002	-0.11003	0.070536	0.141957
K00025	0.110018	0.070568	0.141979
K02735	0.109976	0.070676	0.142155
K05988	-0.10997	0.07071	0.142182
K04026	-0.10991	0.070853	0.142428
K05566	0.109893	0.070909	0.142499
K02472	-0.10987	0.070977	0.142595
K07337	0.109834	0.071043	0.142685
K12517	-0.10975	0.071273	0.143106
K00750	0.109656	0.071505	0.143529
K00103	0.10964	0.071547	0.143572
K04781	-0.10962	0.071602	0.14364
K01586	0.109601	0.071666	0.143728
K00113	0.109556	0.071783	0.143919
K00198	0.109447	0.072067	0.144406
K07150	0.109451	0.072058	0.144406
K03577	0.109417	0.072146	0.14448
K04114	0.109417	0.072146	0.14448
K01085	0.109381	0.072222	0.144591
K08990	0.109365	0.072282	0.144668
K07116	0.10934	0.072331	0.144724
K03576	-0.10931	0.072426	0.14483

K12242	0.109309	0.072412	0.14483
K01428	-0.10929	0.072468	0.144874
K02102	0.109278	0.072511	0.144917
K00614	0.109267	0.072538	0.144929
K01337	-0.10924	0.072584	0.144978
K07493	0.109232	0.072631	0.145032
K00873	0.109217	0.07267	0.145066
K08305	-0.10915	0.072849	0.145382
K10392	0.109122	0.072903	0.145449
K06904	0.109117	0.072933	0.145466
K10728	0.109045	0.073107	0.145771
K08256	-0.10896	0.073353	0.146218
K07495	0.108916	0.073465	0.146399
K01647	0.108755	0.073895	0.147213
K09681	0.108702	0.074035	0.14745
K07731	0.108666	0.074118	0.147574
K00791	0.108655	0.074162	0.147575
K14952	0.108654	0.074148	0.147575
K01490	0.108609	0.074269	0.147746
K03140	0.108529	0.074485	0.148134
K07230	-0.1085	0.074571	0.148261
K10739	0.108488	0.074595	0.148267
K02437	-0.10847	0.074662	0.148356
K10793	0.108454	0.0747	0.148391
K10557	-0.10837	0.074916	0.148777
K01692	-0.10831	0.075079	0.149014
K07037	-0.10832	0.075058	0.149014
K08344	-0.10814	0.075537	0.14988
K01822	-0.1081	0.075652	0.150065
K03195	-0.10806	0.075746	0.150208
K13123	0.108002	0.075912	0.150495
K04079	0.107966	0.076022	0.150669
K03831	-0.10795	0.076055	0.150691
K11622	0.1079	0.076191	0.150916
K07243	-0.10788	0.076261	0.151013
K11747	-0.10782	0.07643	0.151289
K11201	-0.10781	0.076445	0.151289
K01732	0.107706	0.076724	0.151789
K10122	0.107704	0.076741	0.151789
K02173	-0.10769	0.076777	0.151817
K06901	0.107682	0.076802	0.151823
K12891	0.107665	0.076836	0.151846
K01611	0.107647	0.076899	0.151886
K03111	0.107646	0.0769	0.151886
K01430	-0.10763	0.076952	0.151944
K14850	0.107545	0.077169	0.15233
K02352	0.107474	0.077366	0.152552
K02474	-0.10748	0.077362	0.152552
K07182	-0.10748	0.07737	0.152552
K06678	0.107491	0.077318	0.152552
K00595	-0.10731	0.077835	0.153406
K00663	0.107296	0.07787	0.153406

K11517	0.107288	0.077892	0.153406
K07548	0.107288	0.077881	0.153406
K05910	0.10726	0.077959	0.153495
K11615	0.107195	0.07814	0.153808
K06887	0.107164	0.078238	0.153927
K15064	0.107158	0.078246	0.153927
K08253	0.107108	0.078395	0.154156
K02076	0.1071	0.078407	0.154156
K07268	0.107044	0.078563	0.154419
K00897	0.10702	0.078632	0.154511
K08777	0.107003	0.07868	0.154561
K03392	0.106906	0.078962	0.155071
K00980	0.106868	0.079067	0.155233
K01509	0.106856	0.079101	0.155256
K11626	-0.10675	0.079405	0.155808
K01929	0.106681	0.079595	0.156137
K02586	-0.10665	0.079679	0.156257
K00112	0.10659	0.079855	0.156554
K01760	0.106582	0.079876	0.156554
K03273	-0.10656	0.079931	0.156617
K07491	0.106483	0.080157	0.157016
K08195	0.106442	0.080269	0.157191
K02396	0.106408	0.080372	0.157348
K01466	-0.10636	0.08051	0.157573
K02398	0.106341	0.080565	0.157636
K08361	-0.10629	0.080706	0.157866
K05824	0.106216	0.080916	0.158233
K03557	-0.10614	0.081122	0.158591
K08969	0.106104	0.08124	0.158777
K01752	-0.10609	0.081271	0.158793
K03074	0.106065	0.081356	0.158914
K01819	0.105984	0.08159	0.159325
K00626	0.105928	0.081754	0.1596
K10026	-0.10592	0.081788	0.159623
K13118	0.105812	0.082085	0.160157
K01476	-0.10579	0.082138	0.160215
K08288	0.105742	0.082288	0.160462
K14154	-0.10573	0.082332	0.160501
K00795	-0.10571	0.082394	0.160578
K04505	0.105644	0.082574	0.160883
K06580	0.105586	0.082744	0.161168
K02533	-0.10554	0.082891	0.161409
K12486	0.105288	0.08362	0.162783
K01872	0.105253	0.083725	0.162942
K02386	-0.10523	0.0838	0.163041
K10942	0.105121	0.084114	0.163607
K01826	-0.1051	0.084175	0.163679
K11044	0.104973	0.084554	0.16437
K11446	0.104866	0.084873	0.164944
K05578	0.104823	0.085002	0.165147
K00048	-0.1048	0.085074	0.165215
K01884	-0.10479	0.085109	0.165215

K09565	0.104788	0.085107	0.165215
K06167	-0.10476	0.085179	0.165304
K00841	-0.10474	0.085262	0.165419
K01520	0.10464	0.085551	0.165887
K02192	0.104645	0.085536	0.165887
K02660	0.104613	0.085631	0.165996
K02361	-0.1046	0.085679	0.166043
K11262	0.104573	0.085751	0.166135
K07477	0.104558	0.085797	0.166177
K07047	0.104543	0.085841	0.166216
K08984	0.104459	0.086095	0.166662
K01103	0.10442	0.086214	0.166781
K01697	0.104418	0.086217	0.166781
K09485	0.104415	0.086229	0.166781
K11490	0.104389	0.086308	0.166886
K01181	0.104353	0.086414	0.167045
K02614	-0.1043	0.086577	0.167312
K01137	-0.10428	0.086633	0.167328
K01886	0.104281	0.086633	0.167328
K10361	0.104228	0.086795	0.167594
K11748	-0.10421	0.086838	0.16763
K09015	-0.10413	0.08709	0.168069
K03974	-0.10405	0.087342	0.168509
K12599	-0.10401	0.087449	0.168666
K08260	-0.10392	0.087731	0.169164
K02110	0.103902	0.087788	0.169227
K08341	0.103877	0.087869	0.169335
K08567	-0.10379	0.088132	0.169796
K07240	-0.10375	0.088266	0.170006
K02108	0.103702	0.088404	0.170223
K13633	-0.10366	0.088549	0.170455
K01128	0.103572	0.088806	0.170902
K11786	0.10356	0.088848	0.170935
K01771	-0.10343	0.089254	0.17167
K02500	-0.10342	0.089292	0.171694
K01270	-0.10335	0.089487	0.172021
K01012	-0.10335	0.089514	0.172026
K02853	-0.10332	0.089606	0.172155
K03549	0.103262	0.089769	0.172327
K07307	-0.10325	0.089812	0.172327
K13246	0.103267	0.08976	0.172327
K08076	0.103247	0.089821	0.172327
K12773	-0.10326	0.089793	0.172327
K12537	0.103192	0.089994	0.172611
K06974	0.103165	0.090077	0.172691
K07677	-0.10316	0.090085	0.172691
K03478	-0.10304	0.090465	0.173323
K14261	-0.10305	0.090443	0.173323
K06930	0.103022	0.090528	0.173395
K01003	0.102982	0.090647	0.173575
K05838	0.102901	0.090908	0.174027
K00215	0.102867	0.091008	0.174075

K00521	0.102869	0.091009	0.174075
K10849	0.102876	0.090988	0.174075
K07062	-0.10282	0.091149	0.174294
K10558	-0.10259	0.091889	0.175643
K10901	0.102587	0.091906	0.175643
K02393	-0.10253	0.092073	0.175916
K02387	0.102453	0.092321	0.17634
K04345	0.102389	0.092536	0.176701
K01907	0.102373	0.092587	0.176713
K10562	0.102371	0.092593	0.176713
K00265	-0.10235	0.092647	0.176766
K10364	0.102267	0.092929	0.177256
K07708	-0.10219	0.093179	0.177683
K03841	-0.10217	0.093233	0.177737
K01496	0.102064	0.093569	0.178329
K10623	-0.10205	0.093612	0.178361
K15034	0.102045	0.093644	0.178373
K15196	0.102008	0.093763	0.17855
K00145	-0.10196	0.09391	0.178738
K06880	0.101961	0.093913	0.178738
K12573	0.101943	0.093962	0.178783
K06209	0.101901	0.094097	0.17899
K02397	0.101846	0.094277	0.179282
K13924	0.101833	0.094318	0.179311
K10763	-0.10182	0.094367	0.179355
K11631	-0.10181	0.09441	0.179387
K00884	0.101615	0.095027	0.18046
K14834	0.101624	0.095011	0.18046
K06655	0.10159	0.095124	0.180594
K01643	-0.10156	0.095227	0.180741
K12297	0.101516	0.095353	0.180929
K03950	0.101472	0.095509	0.181175
K08318	-0.10143	0.095646	0.181386
K09459	-0.10141	0.09571	0.181458
K01674	0.101374	0.095833	0.18146
K03315	-0.10137	0.095827	0.18146
K06886	0.101371	0.095843	0.18146
K14437	0.101381	0.095808	0.18146
K13183	0.101396	0.09576	0.18146
K12264	0.101345	0.095913	0.181542
K12452	-0.10127	0.096165	0.18197
K01235	-0.10126	0.096201	0.181988
K06188	-0.10121	0.09635	0.18222
K01081	-0.10115	0.096569	0.182585
K06718	-0.1011	0.096733	0.182844
K01795	-0.10106	0.096859	0.183032
K01894	-0.10102	0.096993	0.183235
K00720	0.100994	0.097089	0.183366
K06919	-0.1009	0.097376	0.183858
K01159	0.100885	0.097436	0.183871
K08064	0.100898	0.097411	0.183871
K05534	-0.10087	0.097495	0.183933

K08987	0.100857	0.097547	0.183956	
K15202	0.100853	0.097561	0.183956	
K01727	-0.10083	0.097627	0.184021	
K14162	0.100826	0.097649	0.184021	
K00140	0.100758	0.097861	0.18437	
K07096	0.100731	0.097951	0.184491	
K00908	-0.10063	0.098305	0.185106	
K14680	0.10059	0.098441	0.185312	
K03613	0.100555	0.09854	0.185448	
K03362	0.100489	0.09878	0.185848	
K00078	0.100422	0.098989	0.186191	
K04071	0.100384	0.099135	0.186415	
K13989	0.100371	0.09918	0.186449	
K13876	-0.10029	0.099446	0.186897	
K01963	0.100253	0.099562	0.186978	
K05286	0.100268	0.09953	0.186978	
K05680	0.100256	0.09957	0.186978	
K01185	-0.10019	0.099773	0.187276	
K06954	0.100193	0.099783	0.187276	
K07314	-0.10016	0.099912	0.187468	
K07592	-0.1001	0.10008	0.187732	
K13533	-0.10009	0.100142	0.187796	
K02202	0.100043	0.100294	0.188031	
K02364	0.100012	0.10038	0.188141	
K05339	-0.09995	0.100578	0.18846	
K00386	0.099943	0.100617	0.188482	
K09862	-0.09989	0.100801	0.188777	
K02339	-0.09987	0.100873	0.188859	
K00368	0.099785	0.101177	0.189378	
K10591	0.099693	0.101496	0.189924	
K11292	0.099627	0.101723	0.190297	
K09746	0.099599	0.101818	0.190423	
K03934	0.099545	0.102005	0.19072	
K00930	-0.09952	0.102084	0.190817	
K03862	0.099468	0.102273	0.191118	
K13816	0.09944	0.102371	0.19125	
K10259	0.099391	0.102541	0.191515	
K00127	-0.09927	0.102925	0.192129	
K02497	-0.09928	0.102911	0.192129	
K07777	0.099258	0.10298	0.192179	
K03350	0.099184	0.10326	0.19265	
K12399	0.099131	0.103446	0.192945	
K03802	0.099104	0.103516	0.193024	
K06308	0.099071	0.103656	0.193232	
K09148	0.099049	0.103734	0.193272	
K10359	0.099054	0.103715	0.193272	
K01390	0.099025	0.103793	0.193278	
K03963	0.099034	0.103784	0.193278	
K09018	-0.09896	0.104043	0.193692	
K02413	0.098921	0.104157	0.193786	
K13628	-0.09892	0.104178	0.193786	
K12405	-0.09893	0.104145	0.193786	

K01652	-0.09887	0.104324	0.194006
K01161	0.098854	0.104418	0.194129
K03828	0.098767	0.1047	0.1946
K13640	0.098655	0.105094	0.19528
K00848	-0.09862	0.105235	0.195489
K00118	-0.09858	0.105344	0.195639
K01812	-0.09858	0.105374	0.195642
K09016	-0.09857	0.105419	0.195673
K02226	0.09851	0.105611	0.195872
K07323	-0.09851	0.105609	0.195872
K03868	0.098529	0.10557	0.195872
K12295	0.098484	0.105729	0.196037
K14358	0.098462	0.105808	0.196131
K14809	0.0984	0.106029	0.196488
K00852	-0.09837	0.106118	0.1966
K07506	-0.09832	0.106277	0.196842
K08803	0.098301	0.106381	0.196983
K06219	0.098259	0.106531	0.197207
K06929	0.09814	0.106958	0.197944
K01191	-0.09812	0.106989	0.197948
K01800	-0.09805	0.107298	0.198467
K07069	0.098025	0.107371	0.198538
K11127	-0.09802	0.107394	0.198538
K00054	0.097973	0.107529	0.198734
K02114	0.097913	0.107746	0.199083
K01666	0.097881	0.107864	0.199246
K07785	-0.09786	0.107983	0.199413
K15103	0.097822	0.108106	0.199588
K02206	-0.09781	0.108147	0.19961
K09473	0.097798	0.108191	0.199638
K00405	0.097782	0.108252	0.199697
K12995	0.097714	0.108466	0.199991
K07975	0.097722	0.10847	0.199991
K06609	-0.09759	0.108939	0.200803
K03476	-0.09753	0.109167	0.201116
K05351	-0.09754	0.109142	0.201116
K05757	0.097503	0.109265	0.201243
K05845	-0.09742	0.10954	0.201695
K00599	0.097352	0.109787	0.20205
K13010	-0.09735	0.109791	0.20205
K11710	-0.09734	0.109863	0.202121
K13009	-0.09733	0.109888	0.202121
K00316	0.097219	0.110309	0.20284
K00931	-0.09715	0.110529	0.203136
K11250	-0.09716	0.110518	0.203136
K00656	-0.09711	0.110671	0.203343
K07224	0.097057	0.110904	0.203717
K11013	0.097008	0.111086	0.203997
K09167	0.096964	0.111216	0.204182
K13745	0.096902	0.111477	0.204606
K12213	-0.09685	0.111656	0.20488
K03417	0.09676	0.11197	0.205402

K05020	-0.09675	0.112024	0.205447
K10249	0.096733	0.112106	0.205542
K13735	0.096664	0.112327	0.205893
K07233	0.096652	0.112408	0.205988
K07157	0.09661	0.112565	0.20622
K01975	0.096496	0.112956	0.206881
K00174	-0.09645	0.113136	0.207127
K00676	0.096444	0.11315	0.207127
K01613	-0.09643	0.113218	0.207141
K01689	0.09643	0.113204	0.207141
K07031	-0.0964	0.113315	0.207264
K01506	-0.09635	0.113494	0.207536
K07164	0.096312	0.113646	0.207759
K10857	-0.0963	0.113738	0.207873
K07768	0.096241	0.113914	0.208139
K00837	-0.09623	0.113969	0.208183
K15172	0.096214	0.114055	0.208287
K01420	-0.09618	0.114148	0.208346
K07979	0.09618	0.114144	0.208346
K07121	-0.09614	0.114327	0.208618
K00369	0.095967	0.11499	0.209716
K06403	0.095963	0.114968	0.209716
K09568	0.095881	0.115318	0.210259
K02389	0.095796	0.115601	0.21072
K11921	0.095749	0.115783	0.210995
K14287	0.095709	0.115975	0.211289
K10116	0.095577	0.116438	0.212078
K07714	-0.09549	0.116769	0.212624
K00071	0.095449	0.116972	0.212846
K00322	0.095457	0.116944	0.212846
K14670	-0.09545	0.116984	0.212846
K10018	0.095421	0.117081	0.212966
K01419	0.095388	0.117166	0.213066
K10439	-0.09528	0.117631	0.213686
K11472	-0.0953	0.117554	0.213686
K12943	0.095282	0.117616	0.213686
K07904	0.09528	0.117626	0.213686
K02855	0.095191	0.117926	0.214109
K04551	0.095209	0.117902	0.214109
K03249	0.095177	0.118023	0.214228
K09139	0.095169	0.118055	0.214231
K03424	0.095041	0.118508	0.214997
K07565	0.095031	0.11859	0.215089
K07065	-0.09488	0.119169	0.216081
K08681	0.094757	0.119619	0.216841
K01082	0.09473	0.119768	0.217054
K14800	0.094713	0.119834	0.217117
K09607	0.09465	0.120038	0.217314
K04783	0.094664	0.120027	0.217314
K00258	0.094663	0.120031	0.217314
K07080	0.094622	0.120149	0.217436
K13791	0.094612	0.120231	0.217436

K07228	0.09462	0.120202	0.217436
K02275	0.094613	0.120231	0.217436
K01661	-0.09454	0.120481	0.217832
K04936	-0.09445	0.120845	0.218432
K00492	0.094379	0.121107	0.218824
K08566	0.094386	0.121125	0.218824
K01118	0.094349	0.121226	0.218899
K01770	-0.09434	0.121252	0.218899
K02408	0.09434	0.121262	0.218899
K01923	0.094302	0.121412	0.219114
K03671	-0.09417	0.121915	0.219963
K01948	0.094165	0.122	0.22006
K12514	0.09413	0.122142	0.2202
K12515	0.09413	0.122142	0.2202
K03543	-0.09398	0.122686	0.221124
K09506	0.093963	0.12281	0.221289
K10241	0.093914	0.123006	0.221585
K00986	-0.09381	0.123391	0.22222
K05911	-0.0938	0.123467	0.222298
K01223	-0.09377	0.123517	0.222331
K02902	0.0937	0.123815	0.222751
K14157	0.093715	0.123805	0.222751
K12296	0.093679	0.12395	0.222935
K01468	-0.09364	0.124048	0.222996
K02763	0.093647	0.124026	0.222996
K13245	0.093546	0.124435	0.223575
K12586	0.093563	0.124416	0.223575
K00851	-0.09353	0.124515	0.223603
K02400	0.093533	0.124486	0.223603
K12582	0.093453	0.124861	0.224166
K03297	-0.09337	0.125165	0.224595
K08476	-0.09338	0.125166	0.224595
K10715	-0.09333	0.125326	0.224825
K05673	0.093228	0.125779	0.22552
K09965	0.093229	0.125773	0.22552
K14590	0.093217	0.125823	0.225541
K00101	0.09318	0.12592	0.225597
K03492	-0.09318	0.12591	0.225597
K01115	0.093145	0.126063	0.225736
K07239	-0.09315	0.126058	0.225736
K04764	-0.0931	0.126255	0.225962
K04456	0.093114	0.126241	0.225962
K13939	0.093047	0.126516	0.226372
K03278	0.093027	0.126599	0.226462
K03813	-0.09299	0.126706	0.226594
K05288	-0.09295	0.126929	0.226875
K10796	0.09294	0.126901	0.226875
K06865	0.092913	0.127068	0.227064
K14534	0.092865	0.127211	0.227261
K01572	-0.09285	0.12728	0.227326
K00090	0.092782	0.127604	0.227846
K00130	0.092697	0.127901	0.228306

K00759	0.09269	0.127928	0.228306
K10021	0.092558	0.128533	0.229326
K06888	0.09252	0.128631	0.229442
K02399	-0.09251	0.128736	0.22957
K01992	0.092419	0.129052	0.230073
K02111	0.092388	0.129179	0.230125
K05785	-0.09239	0.129159	0.230125
K12139	0.092402	0.129181	0.230125
K00971	-0.09238	0.129227	0.230148
K14050	0.092369	0.129317	0.23025
K02459	-0.09236	0.12936	0.230266
K08078	-0.09233	0.12942	0.230314
K04653	-0.09218	0.130039	0.231356
K03272	-0.09216	0.130117	0.231435
K02077	-0.09212	0.130294	0.231689
K11304	0.092071	0.130563	0.232109
K14978	-0.09203	0.130734	0.232353
K02790	-0.09187	0.131347	0.233382
K01376	0.091822	0.13155	0.233683
K03064	0.091826	0.131593	0.233699
K01854	-0.09179	0.131705	0.233838
K10537	0.09171	0.132024	0.234343
K09860	0.091538	0.132813	0.235683
K09806	0.091473	0.133029	0.236005
K06013	-0.09145	0.133139	0.23614
K07716	-0.09142	0.133254	0.236258
K13513	0.09143	0.133274	0.236258
K09797	-0.09135	0.13354	0.236646
K12144	0.091363	0.133561	0.236646
K07000	0.091312	0.133715	0.236857
K13509	0.091266	0.133976	0.23726
K01630	0.091205	0.134174	0.237548
K12266	-0.09114	0.134514	0.238028
K06869	0.091147	0.134484	0.238028
K02504	0.091119	0.134604	0.238127
K00334	0.091063	0.134781	0.238323
K01821	-0.09105	0.134836	0.238323
K02410	0.091042	0.134872	0.238323
K07566	-0.09106	0.134797	0.238323
K11263	0.091038	0.134888	0.238323
K03071	-0.091	0.135033	0.238519
K03073	-0.0909	0.135487	0.23926
K01075	-0.09088	0.135655	0.239444
K08592	0.090874	0.135661	0.239444
K08307	-0.09084	0.135742	0.239478
K13588	-0.09085	0.135749	0.239478
K01119	0.090797	0.13593	0.239736
K07146	0.090791	0.136019	0.23983
K11216	-0.09072	0.136254	0.240184
K11383	-0.0907	0.136358	0.240306
K07641	-0.09068	0.136522	0.240533
K02404	0.090652	0.136557	0.240534

K10777	0.090569	0.136985	0.241227	
K03308	0.090536	0.13706	0.241297	
K00870	-0.09053	0.137141	0.241354	
K01933	-0.09051	0.137162	0.241354	
K02388	0.090513	0.137229	0.241409	
K01156	0.090404	0.137636	0.242063	
K04568	0.090409	0.137681	0.242082	
K05836	0.090362	0.137886	0.24238	
K02451	-0.0902	0.138606	0.243583	
K06903	-0.09015	0.138766	0.243803	
K07172	-0.09015	0.138832	0.243857	
K11005	0.090131	0.138901	0.243916	
K03570	0.090085	0.139034	0.244087	
K08224	-0.09007	0.1391	0.244141	
K11897	0.090068	0.139178	0.244215	
K07090	0.090025	0.139297	0.244362	
K01485	0.089981	0.139493	0.244576	
K02416	0.089974	0.139525	0.244576	
K08900	0.089991	0.139515	0.244576	
K00013	-0.08991	0.139818	0.244969	
K06189	-0.0899	0.139837	0.244969	
K14439	0.089914	0.139856	0.244969	
K00102	-0.08986	0.140039	0.245229	
K02124	-0.08983	0.140151	0.245363	
K03553	0.089783	0.140368	0.245679	
K13629	-0.08977	0.140474	0.245803	
K07749	0.089729	0.140608	0.245976	
K01957	-0.08962	0.141091	0.246757	
K14548	0.089609	0.141211	0.246905	
K13581	0.089539	0.141453	0.247266	
K05886	-0.08953	0.141571	0.247409	
K03343	0.089513	0.141638	0.247463	
K10565	-0.08948	0.141779	0.247647	
K00129	-0.08946	0.141886	0.247771	
K00657	-0.08935	0.14228	0.248397	
K02446	-0.08934	0.142348	0.248452	
K02317	-0.08934	0.142398	0.248477	
K14293	0.089324	0.142489	0.248572	
K01104	0.089256	0.142718	0.24891	
K03366	-0.08914	0.143239	0.249628	
K05539	-0.08914	0.143236	0.249628	
K01004	0.089158	0.143233	0.249628	
K07483	0.089085	0.143489	0.250001	
K04749	0.088991	0.143913	0.250614	
K02949	0.089014	0.143881	0.250614	
K13205	0.088974	0.144065	0.250814	
K03442	0.088886	0.144385	0.251309	
K11126	-0.08889	0.14446	0.251376	
K13155	0.088865	0.144556	0.251479	
K12376	0.088845	0.144648	0.251576	
K01838	-0.08881	0.144721	0.25164	
K11939	-0.08881	0.144815	0.251739	

K10891	0.088753	0.145065	0.252047
K11481	0.088755	0.145055	0.252047
K12998	-0.08872	0.145162	0.252153
K08196	0.088717	0.145229	0.252205
K13012	0.088684	0.145305	0.252273
K14060	-0.08859	0.145711	0.252916
K09419	0.088602	0.145754	0.252927
K12434	0.08859	0.145809	0.252958
K08315	0.088542	0.146031	0.253279
K02349	0.088429	0.146548	0.254112
K02859	0.088413	0.146619	0.254172
K11228	-0.08836	0.14687	0.254542
K06131	0.088239	0.147341	0.255294
K07104	0.088125	0.147944	0.256275
K01132	-0.0881	0.148063	0.256353
K08698	0.088106	0.14803	0.256353
K06606	-0.08807	0.14811	0.256371
K02115	0.088025	0.148328	0.256682
K02381	0.087962	0.148618	0.256863
K03670	-0.08798	0.148599	0.256863
K08282	0.087968	0.14859	0.256863
K08368	-0.08798	0.148537	0.256863
K10820	0.087969	0.148587	0.256863
K00133	0.087881	0.148992	0.257405
K02621	-0.08788	0.149006	0.257405
K03303	-0.08783	0.149247	0.257756
K11721	0.087829	0.149315	0.257809
K14657	-0.08779	0.149478	0.258026
K07638	-0.08773	0.149707	0.258356
K11936	0.087643	0.150103	0.258975
K00637	0.087587	0.150444	0.2595
K00371	-0.08752	0.150772	0.26
K00810	-0.08739	0.151359	0.260947
K00147	-0.08733	0.151546	0.261203
K11308	0.087311	0.151741	0.261475
K11707	-0.08729	0.151841	0.261582
K07386	-0.08724	0.151985	0.261765
K14989	0.087216	0.152102	0.2619
K02334	0.087204	0.152161	0.261913
K14442	0.087216	0.152185	0.261913
K02121	-0.08718	0.152284	0.262017
K10800	0.087181	0.152353	0.262072
K02355	0.087146	0.152431	0.262076
K07784	-0.08717	0.152407	0.262076
K07390	0.087098	0.152744	0.262548
K02609	0.086996	0.153227	0.263313
K10912	0.086983	0.153287	0.26335
K11648	-0.08695	0.153432	0.263533
K02475	0.086932	0.153529	0.263635
K00602	-0.08688	0.153693	0.263851
K15022	0.08686	0.153788	0.263948
K10220	-0.08682	0.154075	0.264374

K12140	0.08677	0.154215	0.264484
K11840	0.086789	0.154213	0.264484
K00176	-0.08674	0.154373	0.264662
K07112	0.086732	0.154396	0.264662
K01569	0.086707	0.154604	0.264952
K14260	0.086677	0.154661	0.264984
K07567	-0.0866	0.155015	0.265525
K10795	0.086508	0.155468	0.266235
K03186	-0.08647	0.155627	0.266441
K01792	0.086445	0.155771	0.266557
K13287	0.086469	0.155744	0.266557
K01735	0.086423	0.155875	0.266603
K14729	-0.08644	0.155875	0.266603
K13091	-0.08642	0.155973	0.266704
K01412	-0.08637	0.156133	0.266911
K09155	0.086335	0.156388	0.267281
K12657	0.086233	0.156879	0.268053
K02788	-0.08619	0.157083	0.268335
K11195	-0.08616	0.157222	0.268507
K04561	0.086062	0.157703	0.269261
K01228	0.086045	0.15779	0.26931
K04058	-0.08604	0.15781	0.26931
K03555	0.085905	0.158377	0.270021
K13590	0.085904	0.158383	0.270021
K12963	-0.08593	0.158339	0.270021
K12624	0.085923	0.158378	0.270021
K06640	0.085835	0.15881	0.270683
K01657	-0.08579	0.158913	0.270792
K11708	-0.08577	0.159114	0.271067
K03643	-0.08576	0.159165	0.271087
K03547	-0.08568	0.159493	0.271578
K07088	0.085581	0.159959	0.272175
K07280	0.085601	0.159953	0.272175
K09131	0.085599	0.159961	0.272175
K03196	-0.08559	0.160005	0.272181
K09791	-0.0855	0.16045	0.272872
K11717	0.085395	0.16087	0.273518
K03148	-0.08537	0.160976	0.273632
K01355	-0.08538	0.161025	0.273647
K07783	-0.08532	0.161217	0.273906
K14048	-0.08529	0.161389	0.274131
K05582	0.085293	0.161464	0.274191
K03667	0.0852	0.161829	0.274716
K03758	-0.0852	0.161853	0.274716
K09643	-0.08518	0.162016	0.274925
K07027	0.085117	0.162237	0.275233
K12795	0.08512	0.162318	0.275303
K05541	-0.08504	0.162599	0.275672
K07246	-0.08505	0.162656	0.275672
K08277	-0.08506	0.162639	0.275672
K08988	0.084998	0.162923	0.276015
K12687	-0.085	0.162938	0.276015

K13732	0.084974	0.163045	0.276129
K08995	0.084954	0.163143	0.276226
K02106	0.084919	0.163221	0.276291
K02036	0.084807	0.163779	0.277168
K07154	-0.08472	0.16423	0.277863
K12522	-0.08469	0.16447	0.278201
K01813	-0.08464	0.164598	0.278348
K03441	-0.08465	0.164657	0.278381
K14061	-0.08458	0.164988	0.278873
K08154	0.084506	0.165379	0.279465
K00968	0.084469	0.165468	0.279546
K01663	0.084462	0.165603	0.279706
K07139	0.08435	0.166068	0.280423
K01836	0.08436	0.166117	0.280437
K07317	0.084317	0.166232	0.280562
K00005	0.084302	0.166308	0.280615
K09760	0.084295	0.166344	0.280615
K03655	0.084273	0.166454	0.280731
K00587	0.08417	0.166978	0.281476
K05837	0.084172	0.166965	0.281476
K07308	-0.08412	0.167225	0.281824
K11068	-0.08406	0.167515	0.282244
K06857	0.084036	0.167652	0.282407
K02274	-0.08403	0.167764	0.282494
K02409	0.084002	0.167826	0.282494
K02704	0.084027	0.1678	0.282494
K01087	0.083942	0.168234	0.28311
K08151	-0.08391	0.168313	0.283175
K00984	0.083897	0.168359	0.283184
K02401	0.083883	0.16843	0.283233
K11085	-0.08386	0.168565	0.283361
K12196	0.083872	0.168588	0.283361
K07821	-0.08385	0.168711	0.283499
K02020	-0.0838	0.168878	0.283711
K11633	0.083673	0.169503	0.284691
K03653	0.083468	0.170655	0.286556
K13954	0.083387	0.170969	0.287013
K15116	0.083393	0.171043	0.287068
K00082	0.0833	0.171416	0.287601
K15256	0.083295	0.171444	0.287601
K11108	0.083237	0.171847	0.288207
K12536	0.08308	0.172659	0.289499
K01588	-0.08303	0.172835	0.289723
K10202	-0.08301	0.172938	0.289826
K05593	0.08295	0.173226	0.290167
K14368	-0.08297	0.173215	0.290167
K07457	0.082931	0.173434	0.290445
K08570	-0.0829	0.173574	0.290609
K11900	-0.08289	0.173669	0.290697
K07454	0.082847	0.173762	0.290783
K06859	-0.08278	0.174099	0.291205
K15025	0.082808	0.174077	0.291205

K07746	0.082764	0.174305	0.29148
K00683	0.082704	0.174617	0.291931
K02100	-0.08264	0.174851	0.292252
K05523	-0.08265	0.174903	0.292267
K11732	-0.08252	0.17559	0.293345
K06048	0.082464	0.175765	0.293565
K10821	0.082434	0.176037	0.293949
K00314	0.082378	0.176327	0.294362
K05555	0.082338	0.176542	0.29465
K00081	-0.08222	0.17715	0.295593
K13661	0.082197	0.177285	0.295746
K12957	-0.08218	0.177392	0.295854
K01594	0.082109	0.177748	0.296376
K01177	0.082094	0.177828	0.296438
K13664	0.082064	0.17788	0.296452
K14083	0.082057	0.178027	0.296615
K15066	0.08205	0.178064	0.296615
K00009	0.081989	0.178277	0.296684
K08997	0.082028	0.178183	0.296684
K12141	0.082	0.178216	0.296684
K13815	0.081991	0.178264	0.296684
K02391	-0.08196	0.178541	0.297052
K03405	-0.08192	0.178651	0.297164
K02427	-0.08184	0.179052	0.297759
K14416	-0.08182	0.179293	0.298088
K10916	0.081803	0.17938	0.29816
K02550	-0.08177	0.17956	0.298363
K08332	-0.08176	0.179589	0.298363
K11051	0.081724	0.179799	0.298641
K14018	-0.08168	0.180023	0.298941
K09667	0.081648	0.180208	0.299147
K06599	-0.08164	0.180234	0.299147
K01095	-0.08161	0.180295	0.299177
K10941	-0.08159	0.180504	0.299329
K02604	-0.08159	0.180517	0.299329
K15176	0.081598	0.180475	0.299329
K08983	-0.08147	0.181179	0.300355
K01878	-0.08138	0.181527	0.30086
K11155	-0.08137	0.181717	0.301103
K01467	-0.08131	0.181933	0.301388
K02787	0.081272	0.182109	0.301534
K11938	-0.0813	0.182073	0.301534
K12684	-0.08114	0.182815	0.302559
K00363	-0.08116	0.182812	0.302559
K08294	-0.08114	0.182944	0.302699
K07070	-0.08111	0.183077	0.302848
K14544	0.081067	0.183337	0.303131
K14963	0.081069	0.183325	0.303131
K03829	0.081029	0.183541	0.303397
K15038	-0.08091	0.184215	0.304438
K01152	0.080813	0.184598	0.304925
K13283	0.080817	0.184575	0.304925

K00688	0.080796	0.184694	0.30501
K00799	0.080779	0.184782	0.305083
K02052	0.080685	0.185296	0.305857
K03413	0.080635	0.185572	0.306241
K03309	0.08061	0.185704	0.306244
K06972	0.08061	0.185708	0.306244
K03485	-0.08064	0.185659	0.306244
K06924	-0.08057	0.185915	0.306514
K06192	-0.08058	0.186015	0.306591
K07651	0.080547	0.186051	0.306591
K04844	-0.08054	0.186117	0.306626
K03519	0.080438	0.186649	0.30743
K07054	-0.08044	0.186753	0.307527
K05515	0.080373	0.187004	0.307813
K12290	-0.08039	0.187015	0.307813
K08643	-0.08036	0.18709	0.307862
K11737	0.080364	0.187176	0.307931
K03271	-0.08029	0.187452	0.308311
K06164	-0.08017	0.188251	0.309552
K01234	-0.08012	0.18839	0.309564
K03632	0.080135	0.188438	0.309564
K10543	0.080137	0.18831	0.309564
K10686	0.080136	0.188435	0.309564
K10974	0.080105	0.188487	0.30957
K10544	0.080084	0.188722	0.309883
K10909	0.080036	0.188867	0.310037
K14587	-0.08005	0.188906	0.310037
K02466	-0.08001	0.188984	0.310092
K07130	-0.07998	0.189171	0.310325
K07046	0.079944	0.189379	0.310592
K09768	0.079912	0.189556	0.310809
K00526	-0.07977	0.190331	0.312005
K02590	-0.07978	0.190403	0.312049
K02666	0.079766	0.190491	0.312119
K12056	-0.07975	0.190581	0.312119
K12492	0.079752	0.190568	0.312119
K00958	0.079714	0.190777	0.312366
K06165	-0.07968	0.190958	0.312588
K06858	-0.07965	0.191125	0.312788
K09692	0.079506	0.191819	0.313849
K08301	-0.07949	0.191907	0.313918
K02541	0.079426	0.192391	0.314635
K01091	0.079379	0.192533	0.314793
K14655	0.079388	0.192604	0.314834
K03652	0.079316	0.192885	0.31522
K06395	0.079302	0.192963	0.315273
K11381	-0.07929	0.193038	0.31532
K10780	0.079227	0.193513	0.31602
K05374	-0.0792	0.193662	0.31619
K09668	0.079142	0.193992	0.316653
K06860	-0.0791	0.194113	0.316775
K07695	-0.0791	0.194214	0.316867

K12057	-0.07909	0.19427	0.316882	
K08081	-0.07908	0.194332	0.316908	
K02087	0.078986	0.194877	0.317722	
K14977	-0.0789	0.195229	0.318221	
K11443	0.078839	0.195709	0.318928	
K07049	0.078779	0.196054	0.319415	
K10973	0.078719	0.196393	0.319817	
K10222	0.078727	0.196349	0.319817	
K00117	-0.0787	0.19651	0.319925	
K11896	-0.07869	0.196553	0.319925	
K03719	0.078653	0.196642	0.319996	
K03726	0.078614	0.196863	0.320279	
K03646	-0.07859	0.197014	0.32045	
K03089	-0.07853	0.197321	0.320873	
K01501	0.078504	0.19749	0.321072	
K12299	-0.07851	0.197566	0.321121	
K14326	-0.07847	0.19769	0.321246	
K06162	-0.07845	0.197773	0.321305	
K03486	0.078422	0.197963	0.321539	
K00480	-0.0784	0.198196	0.321841	
K01446	0.078355	0.198347	0.321859	
K09799	0.078379	0.198341	0.321859	
K08653	0.078387	0.198296	0.321859	
K03530	-0.07829	0.198697	0.322277	
K14427	0.078317	0.198698	0.322277	
K12994	-0.07826	0.198881	0.322498	
K06289	0.078227	0.199212	0.322958	
K09732	0.07808	0.20006	0.324257	
K12294	0.078066	0.200145	0.32432	
K02189	-0.07802	0.200289	0.32438	
K05791	-0.07802	0.200416	0.32438	
K06193	0.078033	0.200336	0.32438	
K13853	0.078019	0.200417	0.32438	
K00252	0.078035	0.200324	0.32438	
K09972	-0.07797	0.200557	0.32453	
K10847	0.077968	0.200708	0.324698	
K05562	-0.07784	0.201432	0.325794	
K07093	0.077827	0.201527	0.325809	
K10012	0.077802	0.201536	0.325809	
K04368	-0.0778	0.201696	0.325991	
K03188	-0.07771	0.202076	0.326452	
K06973	0.077717	0.202034	0.326452	
K13767	0.077694	0.2023	0.326738	
K02421	0.077641	0.202476	0.326945	
K13503	0.077646	0.20258	0.327037	
K13819	0.07761	0.202659	0.327087	
K02411	0.0776	0.202715	0.327102	
K13929	-0.07759	0.202909	0.327284	
K04336	-0.07759	0.202923	0.327284	
K10794	0.077529	0.20313	0.327542	
K12257	-0.07751	0.203211	0.327596	
K02573	-0.07749	0.203342	0.327693	

K11711	0.077488	0.203366	0.327693
K11946	0.077462	0.203655	0.328082
K11753	0.077429	0.203715	0.328103
K10398	0.077402	0.204008	0.328496
K01143	-0.07735	0.204294	0.328881
K01193	-0.07732	0.204366	0.328919
K02461	-0.07733	0.204416	0.328924
K07689	-0.0773	0.204472	0.328937
K03230	-0.0773	0.204579	0.329033
K09925	-0.07729	0.204681	0.329053
K12973	-0.07729	0.204687	0.329053
K13126	-0.07724	0.204801	0.32916
K03324	0.077234	0.204858	0.329174
K00941	-0.07722	0.204953	0.329187
K08590	-0.07721	0.204978	0.329187
K02973	0.077232	0.205009	0.329187
K08790	0.077204	0.205172	0.329372
K07474	0.077159	0.205297	0.329497
K11547	0.077135	0.205576	0.329868
K02506	0.077091	0.205837	0.330163
K07676	-0.07708	0.205883	0.330163
K11053	0.07708	0.205904	0.330163
K13016	0.077029	0.206203	0.330567
K10887	0.076967	0.206572	0.331081
K03190	-0.07692	0.206699	0.331208
K10542	0.076859	0.20707	0.331726
K03153	-0.07682	0.207461	0.332274
K02303	0.0768	0.207561	0.332354
K01111	0.076792	0.207607	0.332354
K01724	-0.07674	0.207937	0.332805
K09540	0.076719	0.208041	0.332895
K00670	-0.0767	0.208153	0.332996
K00373	-0.07667	0.208352	0.333217
K01078	-0.07664	0.208387	0.333217
K00952	-0.07657	0.2088	0.3338
K12136	0.07657	0.208927	0.333925
K03435	0.076538	0.209119	0.334154
K06890	0.076474	0.209358	0.334341
K13896	-0.07648	0.209307	0.334341
K09918	-0.07649	0.209402	0.334341
K06893	0.076486	0.209429	0.334341
K13926	0.076476	0.209488	0.334358
K14189	0.076444	0.209678	0.334584
K07709	0.076342	0.210147	0.335254
K02316	0.076261	0.210635	0.335834
K06182	0.076268	0.210591	0.335834
K10806	-0.07626	0.210656	0.335834
K01369	0.076258	0.210795	0.335977
K03082	0.076182	0.211109	0.336328
K03436	0.076197	0.211161	0.336328
K09796	0.076205	0.211114	0.336328
K10009	-0.07617	0.211311	0.336488

K09923	0.076158	0.211395	0.336545
K09503	0.075976	0.212492	0.338213
K00821	0.075942	0.212553	0.338233
K11784	-0.07595	0.212621	0.338262
K05643	0.075926	0.212795	0.338462
K06998	0.075879	0.212932	0.338524
K13254	0.075905	0.212922	0.338524
K11646	0.075876	0.213093	0.338701
K00856	0.075824	0.213267	0.338901
K02784	-0.07581	0.213481	0.339033
K02606	0.07581	0.213498	0.339033
K08475	-0.07581	0.213467	0.339033
K07455	0.07576	0.213654	0.339203
K14465	0.075772	0.213724	0.339236
K02813	-0.07575	0.213859	0.339372
K06163	-0.07571	0.214099	0.339675
K04754	-0.07563	0.214437	0.340133
K08603	0.075634	0.214565	0.340223
K11890	0.075629	0.214592	0.340223
K12214	-0.07552	0.215255	0.341195
K11924	0.075437	0.215619	0.341693
K09014	0.075388	0.215913	0.342081
K12771	0.075311	0.216532	0.342983
K09469	-0.07527	0.216812	0.343348
K01947	-0.07526	0.216868	0.343358
K08346	-0.07525	0.216934	0.343384
K01667	-0.07521	0.217015	0.343433
K09161	-0.0752	0.217232	0.343697
K07519	0.075123	0.217683	0.344332
K07036	0.07506	0.218069	0.344864
K12529	-0.07499	0.218381	0.345278
K05774	-0.07499	0.218497	0.345383
K06910	-0.07494	0.218826	0.345823
K03811	-0.07487	0.219102	0.34618
K01615	-0.07481	0.219474	0.34663
K10601	0.07483	0.219487	0.34663
K03614	0.074752	0.219824	0.347003
K11092	0.074777	0.219819	0.347003
K01649	0.074738	0.21991	0.347059
K12288	-0.07472	0.220172	0.347393
K14015	0.07466	0.220539	0.347894
K14472	-0.07464	0.220666	0.348014
K06208	0.074483	0.221643	0.349475
K01624	0.074378	0.22214	0.350179
K11895	-0.07439	0.222202	0.350197
K07282	0.074355	0.222287	0.35025
K11211	0.074364	0.222385	0.350324
K00294	-0.07431	0.222588	0.350564
K13014	0.07431	0.222718	0.35069
K05946	-0.07421	0.223213	0.351388
K10232	-0.07421	0.223356	0.351533
K02460	0.074194	0.223444	0.351592

K03406	0.074159	0.223507	0.35161
K04618	-0.07415	0.223725	0.351874
K10401	-0.07402	0.22453	0.353059
K00504	-0.074	0.224653	0.353069
K03469	0.073971	0.22469	0.353069
K07698	-0.07398	0.224614	0.353069
K13634	0.073951	0.224815	0.353185
K10017	-0.07395	0.224954	0.353324
K14986	-0.07389	0.225205	0.353637
K02568	-0.07385	0.225597	0.354172
K05781	-0.07378	0.22607	0.354797
K02467	-0.07377	0.226098	0.354797
K01500	-0.07371	0.22636	0.355128
K01262	-0.07369	0.226482	0.355238
K02598	0.073634	0.226813	0.355677
K00675	0.073621	0.226893	0.355683
K11908	-0.07364	0.22692	0.355683
K00077	0.073604	0.227004	0.355734
K01464	0.073461	0.227905	0.357065
K05851	0.073428	0.228116	0.357314
K02805	0.073358	0.22856	0.357929
K11583	0.073369	0.228648	0.357986
K00782	-0.07333	0.228744	0.358054
K01879	-0.0732	0.22955	0.35894
K03182	-0.0732	0.229543	0.35894
K05557	0.073245	0.22944	0.35894
K06331	0.073226	0.2294	0.35894
K09780	-0.0732	0.22957	0.35894
K05780	-0.0732	0.229696	0.358975
K07253	0.073207	0.229683	0.358975
K07499	0.073136	0.230134	0.359578
K04335	-0.07305	0.230667	0.360255
K08269	0.073052	0.230672	0.360255
K01169	-0.07304	0.230745	0.360288
K01564	0.072993	0.230887	0.360428
K13058	0.072993	0.23105	0.360601
K00854	-0.07293	0.231308	0.360922
K11629	0.07287	0.231836	0.361665
K07450	-0.07284	0.2319	0.361682
K11015	0.072844	0.232007	0.361768
K05796	-0.07269	0.233017	0.36326
K14107	0.072658	0.233205	0.363471
K01236	0.072637	0.233339	0.363598
K00193	-0.07253	0.234006	0.364554
K12341	-0.0725	0.234237	0.364833
K09773	-0.07244	0.234427	0.365024
K08077	-0.07246	0.234465	0.365024
K02415	-0.0724	0.234868	0.365568
K02489	0.072383	0.234981	0.365662
K06876	-0.07235	0.235209	0.365852
K13279	0.072348	0.235209	0.365852
K07008	0.07229	0.235419	0.366096

K02549	-0.07228	0.235517	0.366165
K00457	-0.07224	0.23574	0.36643
K07270	-0.07224	0.235898	0.366592
K00450	-0.07217	0.236357	0.367123
K03562	-0.07215	0.236321	0.367123
K04720	-0.07214	0.236399	0.367123
K02117	-0.07206	0.236929	0.367833
K05817	0.072045	0.237016	0.367833
K01204	-0.07208	0.236968	0.367833
K08354	-0.07204	0.237187	0.368016
K12429	0.071964	0.237709	0.368742
K12397	0.071877	0.238278	0.369542
K08163	-0.07186	0.238367	0.369598
K01471	0.071827	0.238606	0.369885
K01447	-0.07174	0.239	0.370412
K13053	-0.07176	0.23906	0.370423
K03684	-0.07172	0.239282	0.370639
K11492	0.07172	0.239307	0.370639
K13018	-0.07161	0.239883	0.371447
K02562	-0.07156	0.240363	0.372024
K11709	-0.07156	0.240353	0.372024
K06602	-0.07153	0.240578	0.372272
K00023	0.071485	0.240857	0.3726
K07186	0.071479	0.240897	0.3726
K11178	-0.07141	0.241349	0.373215
K09702	-0.07131	0.241831	0.373877
K00918	-0.07122	0.242422	0.374409
K07274	-0.07127	0.242266	0.374409
K07672	-0.07123	0.242342	0.374409
K12059	-0.07124	0.242446	0.374409
K12211	-0.07126	0.242319	0.374409
K12202	-0.07123	0.242524	0.374445
K01895	-0.07119	0.242614	0.3745
K04096	0.071167	0.24279	0.374688
K07192	-0.07112	0.24309	0.375067
K03786	0.07108	0.243366	0.375409
K11640	0.071026	0.243727	0.375882
K08982	0.070987	0.244153	0.376455
K11493	0.070971	0.244261	0.376538
K02010	0.070933	0.244349	0.376589
K12240	-0.07087	0.244751	0.377124
K02809	-0.07086	0.244823	0.377152
K12976	-0.07088	0.244895	0.377177
K00216	0.070797	0.245425	0.377696
K03837	-0.07078	0.245505	0.377696
K02133	-0.07079	0.245458	0.377696
K11090	0.070785	0.245501	0.377696
K12248	-0.07079	0.245458	0.377696
K08953	-0.07076	0.245703	0.377916
K13670	0.070689	0.246148	0.378517
K00874	-0.07062	0.246424	0.378856
K03039	0.07061	0.246674	0.379157

K09388	0.070573	0.246921	0.379452
K14667	-0.07056	0.247001	0.37949
K12605	-0.07049	0.247474	0.380132
K01609	-0.07037	0.248105	0.381017
K13497	0.070335	0.248353	0.381312
K07643	-0.07034	0.248497	0.381449
K10539	0.070283	0.248706	0.381685
K04091	-0.07029	0.248846	0.381815
K09706	0.07022	0.249126	0.382159
K12146	-0.07017	0.249625	0.382755
K03815	-0.07017	0.249616	0.382755
K12342	-0.07015	0.249782	0.38291
K03919	-0.07012	0.24998	0.383108
K13113	0.070114	0.250022	0.383108
K03546	-0.07007	0.250165	0.383243
K07449	-0.07004	0.250502	0.383673
K14026	-0.06999	0.250853	0.384125
K06221	-0.06995	0.250987	0.384246
K04056	-0.06996	0.251074	0.384293
K13930	0.06994	0.251201	0.384403
K01699	-0.06987	0.251516	0.384741
K05779	-0.06989	0.251533	0.384741
K02412	0.069813	0.251889	0.385173
K06870	0.069833	0.251927	0.385173
K10016	-0.06969	0.25293	0.386621
K05365	0.069651	0.252994	0.386632
K03731	0.069555	0.253822	0.387812
K12276	-0.06955	0.253891	0.387832
K06938	-0.06951	0.254105	0.388072
K13893	-0.06949	0.25424	0.388193
K05887	-0.06943	0.254682	0.388782
K01726	-0.06939	0.254768	0.388827
K09967	0.06938	0.254847	0.388861
K01551	-0.06935	0.255021	0.38904
K04069	0.069343	0.255103	0.389081
K07075	-0.0693	0.255385	0.389424
K00605	-0.06927	0.255571	0.389536
K03935	0.069308	0.255521	0.389536
K02424	0.069198	0.256276	0.390524
K06181	0.069142	0.256664	0.390943
K07225	-0.06915	0.256636	0.390943
K07781	-0.06913	0.256736	0.390966
K04102	-0.06908	0.257094	0.391338
K00646	0.069079	0.257093	0.391338
K03856	0.069026	0.257283	0.39154
K12291	-0.06902	0.257523	0.391818
K07556	-0.06895	0.257998	0.392454
K10559	-0.06891	0.258058	0.39246
K00040	-0.06878	0.259011	0.393648
K01734	-0.06879	0.258948	0.393648
K13919	-0.06878	0.258994	0.393648
K08793	0.068766	0.259261	0.393942

K03187	-0.06871	0.25945	0.394143	
K00824	0.068722	0.259568	0.394235	
K07079	-0.06865	0.259865	0.394513	
K12111	-0.06866	0.259815	0.394513	
K14080	0.068573	0.260423	0.395099	
K09527	-0.06861	0.260382	0.395099	
K02383	0.068603	0.260395	0.395099	
K09926	-0.06856	0.260721	0.395465	
K10235	0.068477	0.261278	0.396222	
K14591	-0.06843	0.26139	0.396305	
K05971	-0.06839	0.261735	0.396654	
K02850	-0.06841	0.261722	0.396654	
K02851	-0.06827	0.262562	0.39782	
K12843	0.068276	0.262679	0.397909	
K09937	-0.06826	0.262793	0.397995	
K00293	-0.06823	0.262983	0.398195	
K00899	-0.06814	0.26345	0.398761	
K13255	-0.06816	0.263472	0.398761	
K00099	0.068126	0.263552	0.398794	
K06021	0.068143	0.263616	0.398804	
K11391	-0.06808	0.26405	0.399372	
K01945	-0.06802	0.264263	0.399433	
K07097	0.068052	0.264256	0.399433	
K13927	-0.06804	0.264187	0.399433	
K00428	-0.06801	0.264391	0.399538	
K13632	-0.06796	0.264901	0.400221	
K02798	-0.06792	0.264994	0.400274	
K03228	-0.06791	0.265279	0.400617	
K06601	-0.06778	0.266144	0.401836	
K02823	-0.06766	0.266847	0.402808	
K09475	-0.0676	0.267431	0.403602	
K00364	-0.06754	0.267676	0.403883	
K12525	-0.06755	0.267805	0.403989	
K11404	-0.06753	0.267944	0.404111	
K00858	0.067484	0.268082	0.404232	
K12507	-0.06742	0.268567	0.404874	
K11396	0.067398	0.268883	0.405262	
K10240	0.067368	0.269099	0.405498	
K11883	0.067295	0.269614	0.406187	
K04771	0.067197	0.270127	0.40687	
K13015	0.067183	0.270226	0.40693	
K01778	-0.06712	0.270644	0.407415	
K05809	0.067148	0.270666	0.407415	
K14091	0.067097	0.27103	0.407874	
K00015	-0.06706	0.271105	0.407899	
K12875	0.067054	0.271337	0.408158	
K03029	0.067043	0.271419	0.408192	
K07702	-0.067	0.271702	0.408507	
K01510	0.066997	0.271746	0.408507	
K03640	-0.06691	0.272203	0.409104	
K05290	0.066915	0.272336	0.409127	
K12873	-0.06692	0.272332	0.409127	

K01851	0.066902	0.272427	0.409175
K11314	-0.06685	0.272821	0.409677
K00765	-0.06676	0.273295	0.410299
K00745	-0.06671	0.273634	0.410718
K02584	0.066719	0.273747	0.410799
K10921	0.06671	0.273812	0.410807
K15114	0.066699	0.273888	0.410832
K12992	0.066656	0.274007	0.410922
K12218	-0.06665	0.274276	0.411235
K11049	0.066621	0.274452	0.411409
K04025	-0.06658	0.274776	0.411806
K03635	-0.06645	0.275493	0.41254
K05590	0.066483	0.275452	0.41254
K12868	0.06647	0.275543	0.41254
K08762	0.066467	0.275565	0.41254
K02691	0.066474	0.275519	0.41254
K06322	0.066428	0.275852	0.412881
K03755	-0.0664	0.276039	0.413071
K01251	0.066357	0.27617	0.413177
K00020	-0.06626	0.276909	0.413933
K07693	-0.06628	0.276738	0.413933
K10679	-0.06626	0.276887	0.413933
K12251	-0.06625	0.276975	0.413933
K04099	-0.06628	0.276937	0.413933
K02291	0.066264	0.277037	0.413936
K10407	-0.06621	0.277246	0.41416
K07350	-0.06616	0.277804	0.414903
K03262	0.066145	0.277905	0.414964
K14159	0.066124	0.278055	0.415098
K05916	-0.06605	0.278586	0.415712
K13623	0.066052	0.278585	0.415712
K12205	-0.066	0.278955	0.416171
K07180	0.065978	0.279122	0.416241
K03226	-0.06598	0.279116	0.416241
K07181	0.065933	0.279258	0.416308
K14375	0.065955	0.279287	0.416308
K07683	0.065935	0.279438	0.416443
K11041	-0.06587	0.279703	0.416748
K12385	0.065877	0.27986	0.416891
K05775	-0.06573	0.280939	0.418318
K14813	0.065732	0.280921	0.418318
K09162	0.065702	0.281141	0.418492
K09933	-0.0657	0.281177	0.418492
K08714	-0.06568	0.281279	0.418554
K12996	-0.06561	0.281652	0.419018
K11655	-0.06556	0.282211	0.419759
K07640	-0.06552	0.282299	0.4198
K03015	0.065525	0.28244	0.419919
K14396	0.06546	0.282923	0.420456
K11839	0.06546	0.282919	0.420456
K00146	0.065442	0.283052	0.420466
K07063	-0.06542	0.283019	0.420466

K02783	-0.06534	0.283619	0.421128	
K05792	-0.06534	0.283588	0.421128	
K03924	-0.06529	0.283962	0.421546	
K02707	0.065295	0.28414	0.421629	
K13346	-0.0653	0.284092	0.421629	
K14004	-0.06526	0.284395	0.421916	
K06046	0.065198	0.284662	0.422222	
K00701	-0.06516	0.284943	0.422548	
K15111	0.065171	0.28506	0.422631	
K08094	0.065128	0.28518	0.422717	
K00990	-0.0651	0.285403	0.422958	
K11006	-0.06511	0.285476	0.422975	
K02099	0.065037	0.285855	0.423095	
K03477	-0.06508	0.285766	0.423095	
K03656	-0.06504	0.28581	0.423095	
K03727	0.065036	0.285864	0.423095	
K11735	-0.06508	0.285716	0.423095	
K00549	0.064993	0.286181	0.4233	
K00632	-0.06499	0.286186	0.4233	
K02501	-0.065	0.286105	0.4233	
K07470	-0.06499	0.286403	0.423531	
K13003	0.064973	0.286524	0.423618	
K03565	0.06491	0.2868	0.423935	
K07444	0.064898	0.286889	0.423977	
K10541	0.064769	0.287846	0.425299	
K00381	0.064769	0.288044	0.425496	
K11261	0.064735	0.288102	0.425496	
K05815	0.064721	0.288205	0.425558	
K03701	0.064705	0.288327	0.425646	
K10917	-0.06459	0.289205	0.426851	
K03414	-0.0646	0.289279	0.426869	
K00886	0.064527	0.289852	0.427532	
K03918	0.064535	0.289793	0.427532	
K10084	0.064466	0.290315	0.428122	
K00613	-0.06444	0.290501	0.428306	
K08164	-0.06432	0.291412	0.429465	
K09846	0.064322	0.291389	0.429465	
K14310	-0.0643	0.291593	0.429641	
K08313	0.06416	0.29241	0.43066	
K02276	0.064189	0.292397	0.43066	
K03248	0.064172	0.292523	0.430736	
K07234	0.064137	0.292786	0.43103	
K02623	-0.0641	0.292882	0.43108	
K00536	0.064038	0.293333	0.431467	
K02078	0.064042	0.293301	0.431467	
K03580	-0.06404	0.293287	0.431467	
K05793	0.06386	0.294882	0.433654	
K03288	-0.06384	0.295007	0.433745	
K07376	0.063831	0.295098	0.433787	
K01632	0.063814	0.295231	0.433812	
K07357	0.063779	0.295492	0.433812	
K08994	-0.06378	0.295478	0.433812	

K13584	-0.06379	0.295429	0.433812	
K14268	0.063791	0.295406	0.433812	
K11705	0.063791	0.295406	0.433812	
K00397	0.063766	0.295589	0.433861	
K02406	0.063656	0.296224	0.434701	
K02348	0.063666	0.296348	0.434791	
K02526	-0.06362	0.296526	0.434867	
K08999	-0.06362	0.296522	0.434867	
K10877	0.063609	0.296786	0.435156	
K03116	-0.06355	0.297031	0.43533	
K11229	0.063582	0.29699	0.43533	
K10218	-0.06352	0.297247	0.435554	
K09584	0.063532	0.297369	0.435585	
K11325	-0.06352	0.297458	0.435585	
K03184	-0.06352	0.297462	0.435585	
K10553	-0.06351	0.297521	0.435585	
K00028	0.063463	0.297899	0.436047	
K10552	0.063447	0.298016	0.436126	
K02090	0.063437	0.298095	0.436149	
K13587	-0.06339	0.29823	0.436254	
K02616	0.063393	0.298431	0.436452	
K03835	-0.06339	0.298492	0.436452	
K05811	-0.06337	0.298575	0.436481	
K06019	0.063192	0.299766	0.438129	
K03516	-0.06321	0.299844	0.438151	
K02032	0.063164	0.299978	0.438254	
K05594	-0.06313	0.300482	0.438897	
K04486	0.063082	0.300612	0.438994	
K13127	-0.06307	0.30087	0.439278	
K14262	-0.06304	0.3011	0.439434	
K06011	0.063044	0.301105	0.439434	
K02840	-0.06299	0.301283	0.439602	
K02109	0.062983	0.301371	0.439638	
K01243	0.062934	0.301742	0.440086	
K10546	0.062919	0.301858	0.44009	
K12961	-0.06294	0.301873	0.44009	
K05724	0.062915	0.302098	0.440326	
K13410	-0.06286	0.302533	0.440774	
K08960	-0.06286	0.302514	0.440774	
K07391	0.062816	0.302858	0.440889	
K07552	-0.06282	0.302791	0.440889	
K10943	-0.06284	0.302694	0.440889	
K07810	-0.06281	0.302868	0.440889	
K02045	0.062774	0.302978	0.440956	
K08073	0.06279	0.303062	0.440986	
K02690	0.062776	0.303169	0.441048	
K06908	-0.0627	0.303564	0.44153	
K05603	0.062683	0.303886	0.441905	
K10555	-0.06265	0.303955	0.441913	
K00116	-0.06262	0.304341	0.442381	
K00317	0.06252	0.30515	0.443463	
K03451	-0.06247	0.305332	0.443635	

K04092	0.062462	0.305598	0.44391
K05882	-0.06243	0.30565	0.44391
K01210	0.062379	0.306039	0.444007
K01362	-0.06239	0.305941	0.444007
K09699	0.062431	0.305842	0.444007
K12147	-0.06241	0.306026	0.444007
K03464	-0.06242	0.305923	0.444007
K05521	-0.06231	0.30654	0.444641
K02033	0.062286	0.306756	0.44486
K01129	0.062262	0.306948	0.444927
K11072	0.062256	0.306995	0.444927
K12993	0.062293	0.306912	0.444927
K07490	-0.06217	0.307879	0.446114
K01113	-0.06203	0.308767	0.447307
K03019	0.062032	0.30895	0.447478
K01909	0.062016	0.309072	0.447561
K13093	-0.062	0.309205	0.447659
K01799	-0.06196	0.309484	0.447829
K03525	0.061913	0.309668	0.447829
K07771	0.061941	0.309655	0.447829
K08160	-0.06196	0.309548	0.447829
K02423	-0.06193	0.309737	0.447829
K04059	-0.06193	0.309776	0.447829
K10733	0.061965	0.309474	0.447829
K07504	-0.06186	0.310102	0.448206
K10038	0.061753	0.310924	0.449301
K06952	0.061734	0.311071	0.449419
K02048	0.06172	0.31118	0.449482
K01576	0.061717	0.311415	0.449728
K03329	-0.06169	0.311589	0.449885
K07480	-0.06165	0.311976	0.450255
K11057	0.061649	0.311949	0.450255
K00534	-0.06158	0.3125	0.45054
K03446	-0.06156	0.312408	0.45054
K07578	0.06157	0.31236	0.45054
K12436	0.061586	0.312444	0.45054
K11008	0.061579	0.312499	0.45054
K02112	0.061471	0.313139	0.451367
K01915	-0.06145	0.313301	0.451506
K02636	0.061461	0.313427	0.451594
K07397	-0.06142	0.313562	0.4516
K03863	0.061449	0.313523	0.4516
K08267	0.061389	0.313994	0.452128
K12443	-0.06133	0.314458	0.452701
K02438	-0.06128	0.314624	0.452847
K05997	-0.0613	0.314703	0.452865
K11528	0.061281	0.314852	0.452985
K06596	0.061256	0.315048	0.453164
K03848	0.061249	0.315107	0.453164
K03641	-0.06117	0.315531	0.453679
K12831	0.061183	0.315625	0.453719
K01215	0.061123	0.31589	0.454005

K01942	0.061125	0.316091	0.4542
K01273	-0.06105	0.316487	0.454675
K01517	-0.06104	0.316746	0.454857
K03259	0.061049	0.31669	0.454857
K06144	-0.06101	0.316974	0.45509
K00427	-0.06096	0.317393	0.455596
K11203	-0.06092	0.317465	0.455605
K03765	-0.06092	0.317692	0.455837
K02521	-0.06085	0.318036	0.456235
K03838	-0.06085	0.318279	0.456489
K02448	0.060799	0.318468	0.456665
K07100	-0.06078	0.318833	0.457093
K09992	-0.06073	0.318983	0.457213
K02670	0.06075	0.319069	0.457242
K07351	-0.06073	0.319219	0.457362
K00598	0.060709	0.319396	0.457454
K08234	0.06068	0.319416	0.457454
K10804	-0.06068	0.319627	0.457584
K07061	0.060679	0.319639	0.457584
K09136	0.060637	0.319758	0.457659
K05922	0.060567	0.320534	0.458675
K00001	-0.06047	0.321104	0.459395
K11922	0.060473	0.321288	0.45941
K13004	-0.06045	0.321249	0.45941
K00804	-0.06047	0.321314	0.45941
K05844	0.060345	0.322096	0.460432
K00242	-0.06035	0.322302	0.460632
K02035	0.060257	0.322808	0.461092
K05831	-0.06026	0.322966	0.461092
K07236	0.060275	0.322873	0.461092
K05522	-0.06029	0.322744	0.461092
K04512	0.060257	0.323025	0.461092
K00987	0.060266	0.322946	0.461092
K14784	0.060203	0.323453	0.461608
K00864	0.0601	0.32407	0.462393
K03304	0.060108	0.324221	0.462512
K03514	0.060077	0.324468	0.46277
K05601	0.060054	0.324655	0.46294
K00214	0.060005	0.32505	0.463401
K03338	0.059962	0.32518	0.463401
K12990	0.05997	0.325116	0.463401
K14647	0.05998	0.325258	0.463417
K00772	-0.05989	0.325804	0.464003
K09143	-0.05992	0.325762	0.464003
K08219	-0.05988	0.326069	0.464189
K11423	0.059884	0.326034	0.464189
K06654	-0.05986	0.326198	0.464277
K01200	-0.05982	0.326316	0.46435
K03247	-0.05981	0.326632	0.464703
K12410	0.059729	0.327288	0.465541
K02420	0.059669	0.327553	0.465822
K14415	0.059647	0.327734	0.465983

K13529	0.059628	0.327891	0.46611
K13615	-0.05961	0.328258	0.466536
K01450	0.059588	0.32843	0.466685
K10673	-0.05944	0.329664	0.468341
K01920	0.059422	0.32978	0.46841
K08516	0.059409	0.329888	0.468467
K01610	-0.05936	0.330046	0.468594
K09134	-0.05934	0.330223	0.468749
K02487	-0.05932	0.330591	0.469175
K11685	-0.05929	0.330831	0.469419
K05778	-0.05927	0.330996	0.469557
K01023	-0.05921	0.331291	0.46988
K03896	-0.05922	0.331413	0.469955
K02278	-0.05913	0.331972	0.470652
K00276	0.059146	0.332042	0.470654
K03590	0.05911	0.332116	0.470662
K05777	-0.0591	0.332214	0.470705
K01720	-0.059	0.333045	0.471785
K08168	0.058996	0.333268	0.47181
K01483	0.059001	0.333229	0.47181
K11627	0.059011	0.333142	0.47181
K01344	-0.05895	0.333681	0.472298
K05795	0.058904	0.333802	0.47232
K09471	0.058927	0.333833	0.47232
K07056	-0.05887	0.33411	0.472614
K00743	-0.05884	0.334303	0.472728
K02665	-0.05887	0.334327	0.472728
K00669	0.058717	0.335565	0.474382
K07326	-0.05859	0.3364	0.475465
K00989	0.058517	0.336993	0.476205
K08682	-0.05843	0.337681	0.47669
K11329	-0.05845	0.337581	0.47669
K02442	-0.05846	0.337651	0.47669
K12500	-0.05848	0.33749	0.47669
K09008	0.058479	0.337527	0.47669
K11004	0.058391	0.338256	0.477404
K11689	-0.05835	0.338385	0.477489
K11175	-0.05832	0.33866	0.477531
K15037	-0.05834	0.338692	0.477531
K00148	0.058351	0.338594	0.477531
K00543	0.058345	0.33864	0.477531
K09932	0.058224	0.339647	0.47869
K10015	-0.05822	0.339653	0.47869
K02818	0.058163	0.339927	0.478963
K12066	0.058183	0.339985	0.478963
K01971	0.058127	0.340228	0.479136
K02034	0.05811	0.340373	0.479136
K00892	-0.05813	0.340386	0.479136
K03220	-0.05815	0.340271	0.479136
K02116	0.058028	0.341056	0.479921
K05573	0.058051	0.341082	0.479921
K05281	0.058013	0.341177	0.479956

K11741	0.057991	0.341358	0.480112
K10726	0.058004	0.341479	0.480185
K09023	0.057973	0.341733	0.480346
K07797	-0.05798	0.341705	0.480346
K02812	-0.05796	0.341849	0.480413
K09124	-0.05787	0.342379	0.480961
K07222	-0.0579	0.342312	0.480961
K11925	-0.05783	0.342899	0.481496
K00327	-0.05784	0.342864	0.481496
K03203	0.057772	0.343412	0.48202
K12686	0.057775	0.343389	0.48202
K09978	0.057735	0.34372	0.482353
K05814	0.057665	0.344084	0.482766
K03284	-0.05764	0.344336	0.482826
K12548	-0.05766	0.344316	0.482826
K06080	-0.05767	0.344236	0.482826
K13075	-0.05762	0.344458	0.482898
K06132	0.057611	0.344762	0.483128
K09928	0.057617	0.344709	0.483128
K06077	-0.05758	0.345038	0.483418
K14640	-0.05749	0.345515	0.483917
K10577	0.057519	0.345535	0.483917
K11647	-0.0575	0.34569	0.484037
K11635	-0.05747	0.345928	0.484271
K02047	0.057431	0.346052	0.484347
K02782	-0.05739	0.346367	0.484689
K09996	-0.05739	0.346623	0.484861
K08739	0.057389	0.34663	0.484861
K11382	0.057338	0.347054	0.485355
K03293	-0.05728	0.347311	0.485616
K03810	0.057294	0.347431	0.485686
K01448	0.057206	0.347942	0.486301
K05713	-0.05722	0.348063	0.486373
K02031	0.057083	0.34898	0.487555
K07811	-0.05709	0.349154	0.487603
K14288	-0.05709	0.349155	0.487603
K00285	-0.05698	0.350077	0.488593
K12982	-0.05698	0.350063	0.488593
K10589	0.056993	0.349968	0.488593
K10924	-0.05694	0.350419	0.488971
K13042	0.056931	0.350497	0.488982
K09116	0.056758	0.351734	0.490411
K10124	-0.05679	0.351731	0.490411
K04536	-0.05679	0.351695	0.490411
K12615	0.05674	0.351888	0.490526
K06985	0.056722	0.352267	0.49095
K11961	0.056714	0.352334	0.49095
K00820	0.056673	0.352457	0.490965
K01768	-0.05667	0.352488	0.490965
K03759	-0.05669	0.352567	0.490977
K08721	-0.05665	0.352878	0.49131
K01908	-0.05662	0.353167	0.491613

K08345	-0.05659	0.353375	0.491804
K00385	0.056523	0.353741	0.492214
K12743	0.056535	0.353867	0.49229
K00721	0.056453	0.354332	0.492683
K01535	0.056476	0.354364	0.492683
K06026	-0.05646	0.35424	0.492683
K02658	0.056457	0.354528	0.492813
K00561	-0.05642	0.354837	0.493044
K12972	0.056395	0.354827	0.493044
K07336	-0.05641	0.354964	0.49312
K13658	0.056379	0.355195	0.493342
K07269	-0.05636	0.355375	0.493492
K00073	-0.05632	0.355486	0.493548
K03181	-0.05633	0.355609	0.493619
K15192	0.056303	0.355848	0.493851
K07663	0.056265	0.355945	0.493888
K07780	-0.05624	0.3564	0.49432
K11793	0.056238	0.356399	0.49432
K02160	-0.05615	0.356948	0.49498
K06905	-0.05614	0.357025	0.494988
K06884	0.056139	0.357252	0.495004
K11066	0.056143	0.357212	0.495004
K05572	0.056152	0.357135	0.495004
K02706	0.056102	0.357568	0.495343
K03707	0.056045	0.357832	0.495609
K04940	-0.056	0.358257	0.496098
K05372	0.055954	0.35861	0.496486
K14988	0.055915	0.358947	0.496854
K08952	-0.05591	0.359226	0.49714
K02635	0.055892	0.359369	0.497238
K11073	-0.05586	0.359653	0.497533
K00973	0.05575	0.36037	0.498424
K04061	0.055725	0.360583	0.498619
K05800	0.055704	0.36076	0.498764
K02848	0.055694	0.361079	0.499105
K05966	0.055625	0.361448	0.499514
K01541	0.055636	0.361579	0.499596
K04688	0.055566	0.362187	0.500335
K10682	0.055554	0.362288	0.500375
K08710	-0.0555	0.36274	0.500818
K12049	0.0555	0.362754	0.500818
K10085	0.055444	0.363245	0.501396
K01317	0.055408	0.363558	0.501727
K05986	0.055344	0.364108	0.502387
K06373	0.055223	0.364934	0.503424
K12776	0.055233	0.365072	0.503515
K02363	-0.0552	0.365381	0.50384
K04035	-0.05508	0.366188	0.504852
K13695	-0.05504	0.366482	0.505157
K05596	-0.05501	0.367038	0.505822
K06922	-0.05495	0.367345	0.506143
K10300	-0.05496	0.367418	0.506143

K09845	0.054943	0.367599	0.506292
K00185	-0.05489	0.367861	0.506505
K05245	-0.05491	0.367901	0.506505
K02731	0.054875	0.368191	0.506706
K00505	0.054875	0.368193	0.506706
K07568	0.054723	0.36929	0.508114
K14273	0.054691	0.369802	0.508717
K03683	0.054676	0.369937	0.508801
K03342	0.054609	0.370526	0.509206
K04333	0.054611	0.370507	0.509206
K05939	0.054594	0.370426	0.509206
K01013	-0.05462	0.370442	0.509206
K14407	0.054526	0.371257	0.510108
K01008	-0.05448	0.371411	0.510219
K00019	0.054421	0.371941	0.510338
K08485	-0.05446	0.371834	0.510338
K10014	-0.05448	0.371636	0.510338
K11918	-0.05446	0.371875	0.510338
K12542	-0.05446	0.371875	0.510338
K12503	0.054455	0.371875	0.510338
K08177	-0.05438	0.372334	0.510775
K10194	-0.05439	0.372409	0.510777
K11701	0.054367	0.372648	0.511003
K10352	-0.05435	0.37283	0.511152
K13285	0.054328	0.372999	0.51118
K13938	-0.05433	0.372994	0.51118
K06918	0.054313	0.373132	0.51126
K09963	-0.05425	0.37345	0.511594
K03713	0.05421	0.37404	0.512301
K09786	0.054106	0.374952	0.513346
K09152	0.054109	0.374929	0.513346
K01505	0.054077	0.375208	0.513595
K01062	-0.05401	0.375543	0.513952
K09705	0.053999	0.375666	0.514018
K00436	0.053956	0.376284	0.514761
K01753	0.053911	0.376451	0.514786
K02492	0.053916	0.376403	0.514786
K02390	0.053794	0.377488	0.516102
K02011	0.053739	0.37797	0.516659
K03867	0.053725	0.37833	0.517048
K02622	-0.05367	0.378624	0.517246
K12673	0.053696	0.378593	0.517246
K08963	0.053591	0.379289	0.518052
K00002	-0.0536	0.379453	0.518173
K02771	-0.05349	0.380187	0.519072
K03824	-0.05343	0.380724	0.519703
K01026	0.053407	0.380929	0.51988
K07052	0.05338	0.381171	0.520108
K12369	0.053363	0.381558	0.520532
K13727	0.053326	0.381892	0.520885
K10689	0.053255	0.382526	0.521647
K06214	-0.05323	0.382715	0.521802

K02419	0.053192	0.383088	0.522001	
K09931	-0.05318	0.382961	0.522001	
K13653	0.05317	0.383053	0.522001	
K01655	0.053069	0.383956	0.52267	
K09927	0.053095	0.383958	0.52267	
K10681	-0.05311	0.38386	0.52267	
K11476	-0.0531	0.383669	0.52267	
K09880	0.05311	0.383819	0.52267	
K13635	-0.05307	0.384153	0.522832	
K08673	0.053021	0.384622	0.523367	
K10549	0.052862	0.385814	0.524886	
K00567	0.052792	0.386449	0.525646	
K02039	0.05273	0.387008	0.525917	
K03320	0.05272	0.38709	0.525917	
K03809	-0.05276	0.386764	0.525917	
K15023	0.05272	0.38709	0.525917	
K05874	-0.05275	0.387059	0.525917	
K12041	0.052745	0.387105	0.525917	
K00961	0.052666	0.387579	0.526354	
K06044	0.052695	0.387553	0.526354	
K13541	-0.05267	0.387778	0.526521	
K03735	0.05262	0.387998	0.526716	
K05297	0.052564	0.388504	0.5273	
K01484	-0.05258	0.38863	0.527366	
K02439	-0.05253	0.38881	0.527506	
K07724	-0.05244	0.389814	0.52875	
K11319	0.052438	0.389879	0.52875	
K06404	0.052337	0.390556	0.529563	
K00287	-0.05231	0.390796	0.529578	
K03459	-0.05234	0.390748	0.529578	
K09979	-0.05235	0.390662	0.529578	
K07098	0.05225	0.391343	0.530124	
K12232	-0.05228	0.391353	0.530124	
K14617	-0.05224	0.391716	0.530512	
K06073	-0.05223	0.391803	0.530524	
K03857	0.052157	0.392187	0.530868	
K09164	0.052181	0.39221	0.530868	
K12206	-0.05215	0.392509	0.531168	
K03177	0.05211	0.392619	0.531214	
K06916	0.052118	0.392779	0.531325	
K02752	0.052057	0.393336	0.531975	
K06872	-0.05195	0.394068	0.532861	
K11902	-0.05193	0.394537	0.533285	
K00387	-0.05193	0.394482	0.533285	
K02362	0.051897	0.39479	0.533418	
K09823	-0.0519	0.394745	0.533418	
K06946	0.051861	0.394888	0.533446	
K09020	-0.05184	0.395047	0.533484	
K08964	0.051867	0.39507	0.533484	
K02676	-0.05183	0.395417	0.533848	
K01139	0.051764	0.395768	0.534218	
K02063	0.051773	0.395921	0.53432	

K01151	0.051717	0.396204	0.534596
K13574	-0.05168	0.396737	0.535211
K12538	0.051634	0.397193	0.535721
K03749	-0.05156	0.39791	0.536584
K00018	0.051476	0.398407	0.536984
K06158	-0.05147	0.39844	0.536984
K12733	0.051499	0.398434	0.536984
K05358	0.051421	0.399153	0.53784
K01740	-0.05131	0.399935	0.538788
K11739	-0.05132	0.400056	0.538846
K00737	0.051279	0.400455	0.539278
K01591	-0.05123	0.400634	0.539414
K03482	0.051235	0.400856	0.539608
K04718	-0.05122	0.400982	0.539672
K09949	-0.05118	0.401128	0.539763
K11634	-0.05118	0.401348	0.539954
K05517	-0.05117	0.401434	0.539964
K01451	0.051109	0.401778	0.540322
K01590	0.051067	0.402409	0.540986
K03402	0.051039	0.402429	0.540986
K07471	-0.05104	0.402638	0.541162
K00219	-0.05099	0.402885	0.541389
K07399	0.050996	0.403058	0.541411
K11076	0.051	0.403025	0.541411
K04509	0.050965	0.403352	0.5417
K04019	0.050906	0.403654	0.542
K03642	0.050856	0.404122	0.542317
K12787	-0.05088	0.404125	0.542317
K13792	-0.05088	0.404125	0.542317
K11060	0.050833	0.404329	0.542484
K06927	-0.05079	0.404747	0.54294
K07484	-0.05078	0.404842	0.542962
K05561	-0.05078	0.405027	0.543104
K02777	-0.05066	0.405971	0.544234
K03080	0.050676	0.406027	0.544234
K00245	0.050624	0.406268	0.544284
K02558	-0.05062	0.406301	0.544284
K01577	0.050654	0.406232	0.544284
K00329	0.050608	0.406657	0.544656
K00318	-0.05056	0.407099	0.545142
K05903	-0.05052	0.407236	0.54522
K00631	0.050503	0.407635	0.545647
K05301	-0.05048	0.407853	0.545834
K01779	0.050363	0.408931	0.54717
K03623	0.050295	0.409324	0.547588
K00217	0.050313	0.409402	0.547588
K11521	-0.05025	0.409706	0.547889
K10573	0.050244	0.410045	0.548129
K10690	0.050244	0.410045	0.548129
K07290	-0.05023	0.410171	0.548192
K00566	-0.05019	0.410275	0.548224
K11926	-0.0502	0.410458	0.548363

K11082	-0.05019	0.410561	0.548394	
K03489	0.050085	0.411525	0.549575	
K05964	0.050049	0.411621	0.549598	
K00866	0.050063	0.411735	0.549644	
K12549	0.049974	0.412568	0.55065	
K01914	-0.04988	0.41323	0.551107	
K12065	-0.04992	0.413109	0.551107	
K11074	0.04991	0.413169	0.551107	
K12667	0.049907	0.413192	0.551107	
K01640	-0.04984	0.413552	0.551292	
K09954	0.049873	0.413513	0.551292	
K10190	-0.04984	0.413609	0.551292	
K12975	0.049792	0.414033	0.551751	
K06885	-0.04967	0.415223	0.553229	
K10397	0.049674	0.415384	0.553337	
K04016	0.049619	0.415896	0.553913	
K03503	0.04948	0.416965	0.555229	
K14713	0.049474	0.417263	0.55552	
K13637	0.049448	0.417507	0.555728	
K05812	0.04944	0.417588	0.555728	
K14694	0.049432	0.417661	0.555728	
K03201	0.049391	0.418044	0.556079	
K09958	0.049387	0.418086	0.556079	
K02266	0.049374	0.418208	0.556134	
K06157	-0.04936	0.418365	0.556235	
K02919	0.049319	0.418484	0.556286	
K01439	0.049245	0.41919	0.557118	
K03319	-0.0491	0.420537	0.5588	
K03311	0.049086	0.420691	0.558897	
K11007	-0.04901	0.421653	0.560067	
K03736	0.048945	0.422269	0.560778	
K00784	0.048875	0.422693	0.561017	
K14660	0.048907	0.422631	0.561017	
K13713	0.048907	0.422625	0.561017	
K02454	0.048838	0.423048	0.561272	
K02477	-0.04884	0.423002	0.561272	
K06151	0.048783	0.423811	0.562177	
K01612	-0.04872	0.424399	0.56274	
K07278	-0.0487	0.42435	0.56274	
K12231	0.048679	0.424796	0.563159	
K05310	-0.04862	0.425395	0.56381	
K05790	-0.04861	0.425451	0.56381	
K09238	0.048561	0.425925	0.56433	
K12527	0.048522	0.426057	0.564397	
K03821	-0.04853	0.4262	0.564478	
K00413	0.048498	0.426518	0.564791	
K01407	-0.04847	0.426826	0.565091	
K01514	0.048427	0.427199	0.565476	
K12666	0.048402	0.427436	0.56568	
K03820	0.04835	0.427699	0.56592	
K14062	-0.04835	0.42796	0.566158	
K00108	-0.04832	0.428261	0.566447	

K14195	-0.04824	0.428947	0.566976
K07283	-0.04826	0.428827	0.566976
K08100	0.04824	0.42899	0.566976
K12440	0.048253	0.428868	0.566976
K03602	0.048184	0.429286	0.56726
K03222	-0.04816	0.429805	0.567836
K00883	0.048091	0.430177	0.568164
K09458	-0.04809	0.430218	0.568164
K01066	-0.04802	0.430831	0.568865
K12680	0.048036	0.430952	0.568916
K05841	-0.04801	0.431235	0.569105
K07403	0.047978	0.43126	0.569105
K02458	-0.04799	0.431427	0.569216
K00573	-0.0479	0.432264	0.570212
K12979	0.047886	0.432387	0.570266
K10975	-0.04779	0.433297	0.571357
K07748	0.047741	0.433786	0.571891
K02914	0.047688	0.434058	0.572141
K00891	-0.04762	0.434722	0.572824
K06116	-0.04764	0.434742	0.572824
K10550	0.047604	0.435105	0.573085
K12368	0.047604	0.435106	0.573085
K07081	-0.04758	0.435304	0.573236
K01488	0.047535	0.435537	0.573434
K10851	0.047548	0.435648	0.57347
K09709	-0.04752	0.435966	0.57378
K03212	0.047506	0.43605	0.573781
K02570	-0.04746	0.436521	0.574292
K14348	-0.04741	0.436785	0.574529
K01247	0.047373	0.437101	0.574834
K07897	0.047377	0.437305	0.574993
K01628	0.04731	0.437714	0.575422
K04017	-0.04733	0.437804	0.57543
K03404	-0.04728	0.438012	0.575594
K09899	0.04724	0.438628	0.576262
K05818	-0.04723	0.438687	0.576262
K00370	-0.0472	0.43879	0.576288
K00163	0.047199	0.439028	0.57638
K09906	-0.04721	0.438953	0.57638
K01956	-0.04713	0.439493	0.576881
K08217	-0.0471	0.439798	0.577062
K04081	-0.04712	0.439796	0.577062
K07481	-0.047	0.440701	0.578137
K04708	0.046993	0.440789	0.578142
K10206	0.046915	0.441546	0.579026
K01905	0.046847	0.442216	0.579794
K14575	0.046861	0.44232	0.579821
K03711	-0.04679	0.442745	0.580268
K00014	0.046721	0.443445	0.581075
K13630	-0.04671	0.443826	0.581464
K07788	-0.04668	0.444099	0.58171
K07536	0.046639	0.444489	0.582111

K12991	0.046625	0.444619	0.582171
K01713	-0.0466	0.444899	0.582206
K02585	0.046582	0.444801	0.582206
K03782	-0.0466	0.444835	0.582206
K07014	-0.04655	0.445137	0.582385
K09981	0.046565	0.445204	0.582385
K08082	0.046529	0.445563	0.582744
K01405	-0.0465	0.445822	0.582972
K01716	-0.04647	0.446113	0.583242
K01926	0.046447	0.44636	0.583419
K00166	0.046442	0.446418	0.583419
K11750	-0.04641	0.446705	0.583574
K02983	0.046415	0.446676	0.583574
K14064	-0.0464	0.446828	0.583624
K07038	0.046344	0.447128	0.583906
K10697	0.04634	0.447412	0.584166
K11356	0.046293	0.447877	0.584663
K04748	0.046241	0.448141	0.584896
K12690	-0.04621	0.448707	0.585525
K06900	0.046194	0.448845	0.585594
K06320	0.04615	0.449036	0.585652
K08517	0.046172	0.449059	0.585652
K01473	-0.04606	0.44989	0.586403
K07345	-0.0461	0.44982	0.586403
K12236	0.046093	0.449845	0.586403
K04719	-0.04601	0.450395	0.586839
K13931	0.046045	0.450314	0.586839
K06217	0.04602	0.45056	0.586944
K06166	-0.04598	0.450954	0.587346
K11641	0.045912	0.451381	0.587792
K00177	0.045902	0.451483	0.587813
K02123	-0.04587	0.451792	0.587883
K02043	-0.0459	0.45179	0.587883
K09956	0.04591	0.451642	0.587883
K07113	-0.04584	0.452308	0.588333
K11734	-0.04585	0.452275	0.588333
K02256	0.045824	0.452497	0.588468
K14053	-0.04579	0.452805	0.588646
K09700	0.0458	0.452725	0.588646
K05912	0.045756	0.452917	0.588682
K01662	0.045769	0.453032	0.588719
K00407	-0.04572	0.453528	0.589253
K01737	-0.04561	0.454373	0.589981
K03638	-0.04565	0.454256	0.589981
K07128	0.045628	0.45443	0.589981
K03192	0.045638	0.454328	0.589981
K00094	-0.04557	0.454755	0.590292
K06001	-0.04552	0.455227	0.590682
K13154	-0.04555	0.455214	0.590682
K09895	0.045525	0.455446	0.590856
K08279	0.045461	0.455843	0.591259
K07700	0.045407	0.456375	0.591617

K01336	0.045431	0.456379	0.591617	
K02462	-0.04543	0.456421	0.591617	
K08276	-0.04542	0.456461	0.591617	
K03496	-0.04537	0.456758	0.591891	
K10111	0.045306	0.457624	0.592902	
K03035	-0.0453	0.457712	0.592904	
K00832	-0.04524	0.458034	0.59321	
K01825	-0.0452	0.458652	0.593787	
K03207	0.045207	0.458612	0.593787	
K03008	-0.04512	0.459473	0.594405	
K07891	-0.04512	0.459473	0.594405	
K07901	-0.04512	0.459473	0.594405	
K13129	-0.04512	0.459473	0.594405	
K05915	-0.0451	0.459647	0.594519	
K07665	0.045047	0.459962	0.594814	
K03651	-0.04503	0.46013	0.59492	
K07453	0.045009	0.46034	0.595041	
K13777	0.045027	0.460396	0.595041	
K07786	-0.045	0.460658	0.595144	
K14972	-0.045	0.460707	0.595144	
K07681	0.044994	0.460735	0.595144	
K02081	-0.04496	0.460852	0.595184	
K12962	0.044914	0.461533	0.595953	
K12203	-0.0449	0.461687	0.596041	
K02280	0.044863	0.462037	0.596268	
K08977	-0.04487	0.461967	0.596268	
K02062	0.044812	0.462549	0.596817	
K00640	-0.04467	0.463727	0.598226	
K02347	-0.04466	0.464044	0.598523	
K11914	0.044607	0.464604	0.599134	
K03033	0.044548	0.465192	0.59978	
K02936	0.04438	0.466636	0.601388	
K10201	-0.04437	0.466763	0.601388	
K07026	-0.0444	0.466686	0.601388	
K01415	0.044389	0.466788	0.601388	
K03825	0.044346	0.466982	0.601471	
K12145	0.044356	0.467114	0.601471	
K07772	-0.04436	0.467086	0.601471	
K00121	0.044347	0.467213	0.601487	
K09984	-0.0443	0.467643	0.601928	
K03179	-0.04426	0.467843	0.602073	
K07395	-0.04426	0.468041	0.602216	
K02204	0.044157	0.468881	0.602596	
K06962	0.044171	0.468741	0.602596	
K09457	-0.04419	0.468547	0.602596	
K10011	0.044166	0.468784	0.602596	
K07348	-0.04417	0.468947	0.602596	
K08712	0.0442	0.468691	0.602596	
K08973	0.044212	0.468571	0.602596	
K11075	-0.04416	0.469079	0.602652	
K14267	0.044129	0.469399	0.602952	
K01497	-0.04409	0.469847	0.603303	

K09794	-0.04409	0.469804	0.603303
K01965	-0.044	0.470413	0.603568
K10037	-0.04399	0.470523	0.603568
K03225	-0.04404	0.470338	0.603568
K03361	0.044042	0.470279	0.603568
K05875	-0.04402	0.470525	0.603568
K12289	-0.04401	0.470578	0.603568
K03310	-0.04394	0.471052	0.604064
K01840	0.043884	0.471631	0.604694
K13381	-0.04389	0.471808	0.604794
K00535	-0.04388	0.471885	0.604794
K01754	0.043706	0.473431	0.60655
K08736	-0.04374	0.473355	0.60655
K10716	-0.0437	0.473721	0.606809
K02490	-0.04366	0.474173	0.607275
K14852	0.043612	0.474631	0.60775
K02261	0.043555	0.475211	0.608042
K00414	-0.04356	0.47517	0.608042
K02571	-0.04356	0.475114	0.608042
K07394	-0.04356	0.475114	0.608042
K03472	-0.04343	0.476458	0.609523
K03807	-0.04342	0.476551	0.60953
K05977	-0.04341	0.476642	0.609533
K03615	0.043331	0.477238	0.610182
K03241	0.043271	0.478094	0.611164
K08970	0.043257	0.478239	0.611235
K07216	0.043211	0.47846	0.611404
K08296	-0.0431	0.479876	0.612988
K13683	-0.04307	0.479874	0.612988
K02325	0.043045	0.480403	0.613547
K01297	0.043009	0.48052	0.613583
K13582	0.042957	0.481293	0.614456
K12067	-0.04289	0.482002	0.61507
K12262	-0.04288	0.482041	0.61507
K13057	-0.0429	0.481925	0.61507
K03840	-0.04282	0.482699	0.615796
K00609	0.042716	0.483523	0.616733
K04753	0.042719	0.483734	0.616791
K07492	0.042689	0.483795	0.616791
K11089	-0.04271	0.483836	0.616791
K06156	0.042657	0.484129	0.61705
K11617	0.042659	0.484348	0.617215
K01817	-0.04257	0.484976	0.617581
K10772	0.042616	0.484789	0.617581
K05661	0.042596	0.484993	0.617581
K11836	0.042605	0.484905	0.617581
K03704	-0.04254	0.485323	0.617836
K04757	-0.04254	0.485372	0.617836
K00514	0.042457	0.486417	0.619052
K03229	-0.04243	0.486723	0.619327
K02053	-0.04236	0.487225	0.619852
K09456	-0.04235	0.487571	0.620177

K03335	-0.04229	0.487895	0.620476
K08484	-0.04224	0.488677	0.621355
K13689	0.042207	0.488996	0.621445
K13928	0.04222	0.488858	0.621445
K03006	0.042205	0.489018	0.621445
K03219	-0.04219	0.489177	0.621533
K03189	0.042147	0.489374	0.621669
K00261	0.042137	0.489474	0.621681
K07032	-0.04213	0.489592	0.621717
K02528	0.042092	0.48994	0.622045
K05550	-0.04207	0.490375	0.622483
K10551	0.042046	0.490654	0.622607
K11055	0.042046	0.490651	0.622607
K13479	0.042004	0.490843	0.622733
K14699	0.04199	0.49123	0.62311
K02556	0.041954	0.49136	0.623159
K13663	-0.04192	0.49174	0.623527
K01474	-0.04189	0.491977	0.623713
K14724	0.041897	0.492198	0.623878
K02617	-0.04184	0.492526	0.62418
K03800	0.041801	0.49295	0.624603
K03591	0.041767	0.493542	0.625238
K12217	-0.04172	0.49401	0.625554
K03078	0.041731	0.493913	0.625554
K14588	-0.04172	0.494063	0.625554
K00433	-0.04166	0.494431	0.625904
K11932	0.041662	0.494624	0.626033
K07465	-0.04163	0.494768	0.626102
K07305	0.041424	0.497093	0.628928
K00224	-0.04136	0.497487	0.629311
K12268	0.041363	0.497732	0.629506
K02673	0.041271	0.498453	0.630303
K02705	0.041206	0.499368	0.631343
K03425	-0.04118	0.499647	0.63135
K05784	-0.04118	0.499635	0.63135
K09711	0.041193	0.499503	0.63135
K10242	-0.04109	0.500541	0.632364
K03897	-0.04106	0.50087	0.632548
K07680	0.04107	0.500779	0.632548
K00885	0.04105	0.500996	0.63259
K14849	-0.04101	0.501378	0.632958
K00494	0.040967	0.501861	0.633345
K04338	-0.04097	0.501868	0.633345
K05984	-0.04088	0.502741	0.63433
K14136	0.040672	0.504709	0.636697
K12149	-0.04046	0.507152	0.639662
K00060	0.040412	0.507676	0.639926
K07442	0.040419	0.507601	0.639926
K08679	0.040417	0.507621	0.639926
K10965	-0.04041	0.507732	0.639926
K07918	-0.0403	0.508831	0.641193
K11733	0.040291	0.508945	0.64122

K01226	-0.04025	0.509373	0.641643
K13631	0.040209	0.509569	0.641728
K14471	-0.04023	0.509627	0.641728
K01809	-0.04014	0.510313	0.642475
K00560	0.040093	0.510796	0.64254
K01147	-0.04011	0.510662	0.64254
K01249	0.040097	0.510758	0.64254
K03445	-0.04011	0.510804	0.64254
K06310	0.040089	0.510834	0.64254
K07535	0.040081	0.510923	0.64254
K02744	-0.03998	0.512005	0.643666
K00758	0.040002	0.511995	0.643666
K00231	-0.03992	0.512572	0.643793
K08162	0.039962	0.512422	0.643793
K08358	-0.03993	0.512559	0.643793
K09982	-0.03997	0.512282	0.643793
K13650	-0.03995	0.512552	0.643793
K03449	0.039901	0.512821	0.643988
K00868	0.039754	0.514383	0.645822
K05794	0.039768	0.514469	0.645822
K03227	-0.03976	0.514593	0.64586
K11892	0.039741	0.514758	0.645951
K00075	-0.03966	0.515329	0.646549
K05501	-0.03964	0.515571	0.646736
K13599	0.039613	0.515872	0.646894
K14572	0.039612	0.515885	0.646894
K00383	-0.0396	0.515981	0.646897
K03720	0.03961	0.51614	0.64698
K08824	0.039597	0.516275	0.64703
K08326	-0.03958	0.516437	0.647117
K00690	-0.03949	0.517177	0.647285
K02030	0.039496	0.517113	0.647285
K04088	0.039485	0.517228	0.647285
K07789	-0.03951	0.517158	0.647285
K11179	-0.03955	0.516767	0.647285
K10013	-0.03951	0.517199	0.647285
K14449	-0.03954	0.516907	0.647285
K13934	0.039493	0.517385	0.647364
K07661	0.039456	0.517771	0.64773
K00058	0.039414	0.517984	0.647879
K05945	0.0394	0.518131	0.647945
K04752	-0.03938	0.518551	0.648354
K05396	0.039311	0.51908	0.648897
K11731	0.039314	0.519289	0.64904
K07715	-0.03927	0.519516	0.649207
K03737	0.039218	0.520068	0.649779
K00348	-0.0392	0.520306	0.649958
K12270	0.0392	0.520499	0.650083
K06665	-0.03918	0.520744	0.65027
K05709	0.039138	0.521154	0.650665
K10829	0.039096	0.521366	0.650811
K01782	-0.0391	0.521611	0.651

K09776	0.039061	0.521974	0.651263
K02465	-0.03906	0.522011	0.651263
K07670	-0.03901	0.522266	0.651464
K09827	0.038992	0.522718	0.65191
K08139	-0.03894	0.523064	0.652224
K01698	0.038911	0.523347	0.652459
K00685	0.038903	0.52366	0.652614
K02245	0.038889	0.523573	0.652614
K09695	-0.03881	0.524682	0.653769
K03834	0.038748	0.525088	0.654156
K07591	-0.03875	0.525285	0.654285
K06608	0.038642	0.526224	0.655335
K03665	0.03863	0.526347	0.65537
K01766	0.03864	0.526483	0.655377
K03785	0.0386	0.52667	0.655377
K06634	0.038626	0.526629	0.655377
K03277	0.038616	0.526732	0.655377
K02496	0.038607	0.526828	0.655378
K01112	-0.03856	0.527096	0.655594
K09252	0.038534	0.527374	0.655821
K13001	-0.0385	0.527762	0.656185
K12790	-0.03851	0.527903	0.656243
K00909	0.038464	0.52836	0.656574
K00660	-0.03847	0.528267	0.656574
K03778	-0.03841	0.528726	0.656911
K05345	-0.0384	0.528862	0.656948
K00673	-0.03841	0.528947	0.656948
K06445	-0.0384	0.529044	0.656952
K06595	0.038384	0.529222	0.657054
K12070	0.038322	0.529894	0.65777
K01595	0.038287	0.530035	0.657826
K09129	0.038257	0.530586	0.65823
K02592	0.038263	0.530527	0.65823
K01158	-0.03825	0.530646	0.65823
K03753	-0.03818	0.531163	0.658675
K11475	0.038179	0.531196	0.658675
K11746	-0.03815	0.531787	0.659171
K14392	0.038127	0.531747	0.659171
K03522	-0.03804	0.53267	0.660132
K07122	-0.03806	0.532753	0.660132
K12675	0.038044	0.532882	0.660173
K13795	0.038023	0.533104	0.66033
K00467	0.03783	0.535188	0.662792
K02289	-0.03781	0.535387	0.66292
K06160	0.03777	0.535836	0.663357
K13589	0.037704	0.536548	0.664119
K08359	0.037584	0.537854	0.665617
K03521	-0.03752	0.538312	0.665945
K13301	-0.03754	0.538288	0.665945
K01465	-0.03746	0.538946	0.66649
K06898	-0.03746	0.538933	0.66649
K02532	-0.03743	0.53924	0.666734

K01218	0.037379	0.539842	0.66712
K09001	-0.03738	0.539777	0.66712
K12269	0.037405	0.539794	0.66712
K07703	0.037391	0.539945	0.667128
K03400	-0.03734	0.540486	0.667454
K05341	-0.03732	0.540516	0.667454
K10219	0.037339	0.540504	0.667454
K13888	-0.0373	0.540693	0.667454
K06447	-0.03732	0.540671	0.667454
K01962	0.037262	0.541112	0.667733
K07066	-0.03727	0.541066	0.667733
K03119	-0.03723	0.541724	0.668369
K08170	-0.03717	0.542287	0.668824
K12433	0.037176	0.542279	0.668824
K05880	-0.03716	0.542453	0.668909
K06141	-0.03709	0.543245	0.669766
K01833	-0.03704	0.543763	0.670285
K01227	0.037006	0.543893	0.670325
K04021	-0.03697	0.54451	0.670846
K13770	0.036956	0.544431	0.670846
K00991	-0.03692	0.544806	0.671091
K11637	0.036915	0.54512	0.671358
K09985	-0.03688	0.545551	0.671769
K11911	-0.03683	0.54602	0.672227
K08350	0.036809	0.54627	0.672414
K02611	0.036786	0.546527	0.672491
K02281	-0.03679	0.54651	0.672491
K07045	0.036753	0.546648	0.67252
K03639	-0.03673	0.546852	0.672622
K05499	-0.03672	0.547023	0.672622
K13776	-0.03675	0.546937	0.672622
K05881	-0.0367	0.547207	0.672636
K05708	0.036721	0.547229	0.672636
K13794	0.036616	0.548143	0.673639
K04751	-0.0366	0.548334	0.673754
K08363	-0.03661	0.548483	0.673818
K11147	-0.03657	0.54889	0.674197
K03637	-0.03642	0.550307	0.675697
K07773	0.036444	0.550263	0.675697
K03833	-0.03636	0.550921	0.675971
K09126	0.036386	0.550895	0.675971
K03083	0.036385	0.550906	0.675971
K13818	0.036392	0.55083	0.675971
K11264	0.036359	0.55119	0.67618
K00429	0.036336	0.551439	0.676365
K00301	0.036315	0.55168	0.67654
K00007	0.036294	0.551903	0.676693
K05969	0.036268	0.552191	0.676927
K01222	-0.03626	0.55233	0.676976
K07401	-0.03615	0.553471	0.678255
K05681	-0.03609	0.554141	0.678956
K00155	0.036074	0.554323	0.679058

K11395	-0.03606	0.554493	0.679146
K04774	0.036038	0.554715	0.679297
K03316	0.035988	0.555032	0.679565
K00547	0.035956	0.555384	0.679803
K04080	0.035974	0.555424	0.679803
K03762	-0.03593	0.555623	0.679926
K09159	-0.03594	0.555758	0.679971
K05768	0.035886	0.556389	0.680623
K04337	-0.03581	0.557259	0.681567
K07734	-0.03579	0.557459	0.681689
K02007	-0.03557	0.559605	0.684192
K12071	-0.03554	0.560192	0.684789
K00131	0.035469	0.560985	0.685516
K02663	0.035471	0.560967	0.685516
K07190	-0.03545	0.561207	0.685666
K03554	-0.03543	0.561396	0.685775
K10440	0.035391	0.561617	0.685924
K12602	-0.03539	0.561882	0.686126
K11783	0.035229	0.563645	0.688061
K03690	-0.03523	0.563666	0.688061
K06957	0.03518	0.564183	0.68857
K12426	0.035139	0.564643	0.68901
K11898	0.03512	0.564844	0.689134
K05982	0.035024	0.565917	0.69032
K09569	0.034992	0.56627	0.69063
K03869	-0.03496	0.566624	0.690939
K11103	-0.03491	0.566992	0.691266
K06200	-0.03487	0.567435	0.691683
K10418	-0.03478	0.568571	0.692946
K03223	-0.03475	0.568931	0.693262
K06415	0.034723	0.569256	0.693535
K02118	-0.03467	0.569662	0.693892
K02445	0.03465	0.56985	0.693892
K08093	-0.03465	0.569796	0.693892
K07823	-0.03465	0.570031	0.69399
K10536	-0.03459	0.570556	0.694139
K07691	0.03462	0.570409	0.694139
K14374	0.034615	0.570463	0.694139
K04343	-0.03463	0.570282	0.694139
K01816	-0.03454	0.571026	0.694589
K00875	-0.03455	0.571148	0.694614
K02012	0.034492	0.571604	0.694957
K07674	-0.03451	0.57163	0.694957
K12148	-0.03449	0.5718	0.695041
K01487	-0.03439	0.572762	0.695998
K08728	-0.0344	0.572837	0.695998
K13685	-0.03438	0.57289	0.695998
K11014	0.034382	0.573064	0.696087
K06285	-0.03437	0.573225	0.69616
K10121	0.034334	0.573375	0.696219
K06906	-0.03431	0.573611	0.696346
K14597	-0.03432	0.573782	0.696346

K10019	0.034322	0.57373	0.696346
K02299	-0.03428	0.574148	0.696546
K11916	0.034285	0.574139	0.696546
K09857	-0.03427	0.574283	0.696587
K05921	-0.03424	0.574446	0.696663
K02430	0.034188	0.575006	0.697219
K01011	-0.03417	0.575235	0.697374
K01454	-0.03417	0.575485	0.697555
K02768	0.034102	0.575964	0.698013
K14339	-0.03404	0.576866	0.698983
K03743	-0.03399	0.577187	0.699249
K00446	0.033946	0.577935	0.700032
K01584	0.033874	0.57875	0.700896
K01040	-0.03382	0.579101	0.701099
K01058	0.033794	0.579419	0.701099
K03979	0.033796	0.579398	0.701099
K11407	0.033805	0.579521	0.701099
K10579	-0.0338	0.579526	0.701099
K10903	0.033806	0.579506	0.701099
K01786	0.033756	0.579845	0.701239
K11933	0.033778	0.579827	0.701239
K03886	0.033759	0.58004	0.701352
K02040	0.033709	0.580374	0.701558
K05810	-0.03371	0.580414	0.701558
K11736	-0.03371	0.58059	0.701647
K01690	-0.03363	0.581477	0.702482
K01555	-0.03363	0.581484	0.702482
K05393	-0.03347	0.583248	0.704425
K08334	0.033469	0.583296	0.704425
K04113	-0.03341	0.583793	0.704901
K06866	-0.03341	0.584007	0.705022
K09987	-0.0334	0.584097	0.705022
K07285	-0.03339	0.584233	0.705063
K00448	-0.03337	0.584439	0.705184
K02473	0.033359	0.584537	0.705184
K01718	0.033325	0.584913	0.70539
K12143	0.033328	0.584887	0.70539
K00769	-0.03319	0.586478	0.707154
K06330	0.033045	0.587855	0.708691
K07402	-0.03303	0.588032	0.70878
K01910	-0.033	0.588359	0.709051
K10039	0.032958	0.588843	0.709511
K02168	-0.03294	0.589007	0.709584
K10670	0.032909	0.589389	0.70992
K00132	-0.03282	0.590359	0.71084
K13498	-0.03283	0.590304	0.71084
K01252	0.032818	0.590646	0.711063
K00455	0.03279	0.590958	0.71129
K07645	-0.03276	0.591042	0.71129
K13570	0.032735	0.591587	0.711823
K12519	-0.0327	0.591966	0.71209
K02511	0.032697	0.592015	0.71209

K06363	-0.03267	0.592294	0.712177
K09522	-0.03267	0.592286	0.712177
K11444	0.032615	0.592723	0.712569
K02196	0.032581	0.593338	0.713183
K07710	0.032548	0.59349	0.713242
K08166	0.032546	0.593728	0.713403
K08357	-0.03253	0.593926	0.713517
K13920	-0.03244	0.594743	0.714126
K08308	-0.03247	0.594554	0.714126
K14296	-0.03246	0.594654	0.714126
K03488	-0.03241	0.595044	0.714364
K02054	-0.03239	0.595312	0.71456
K09891	0.032383	0.595584	0.714762
K03524	-0.03232	0.596039	0.715184
K12138	0.032226	0.597143	0.716385
K09065	0.032198	0.597466	0.716648
K00625	-0.03216	0.597892	0.716894
K09921	-0.03217	0.597983	0.716894
K05549	-0.03218	0.597884	0.716894
K12543	-0.03215	0.59824	0.717078
K14673	-0.0321	0.598804	0.717629
K03169	-0.03206	0.599067	0.717819
K06350	-0.03204	0.599245	0.717909
K15054	-0.03199	0.600082	0.718786
K03744	0.03193	0.600525	0.718942
K12872	0.031951	0.600505	0.718942
K02659	-0.03197	0.600334	0.718942
K10024	-0.03192	0.600908	0.719277
K09019	0.031877	0.601343	0.719423
K05368	-0.03188	0.601338	0.719423
K13656	-0.0319	0.601135	0.719423
K02687	0.031787	0.602157	0.720272
K14631	-0.03179	0.602349	0.720377
K02055	-0.03175	0.602543	0.720483
K07276	0.031706	0.603298	0.721262
K11891	-0.03169	0.60348	0.721355
K00055	-0.03167	0.60371	0.721505
K03783	-0.03165	0.603949	0.72154
K10040	0.031636	0.60388	0.72154
K02018	-0.0316	0.60426	0.721787
K01714	0.031579	0.604536	0.721991
K03317	-0.03157	0.604674	0.722031
K01459	-0.03148	0.605675	0.723102
K01444	0.031398	0.606601	0.723832
K07459	0.031412	0.606449	0.723832
K09759	-0.03141	0.606497	0.723832
K01364	0.031373	0.606898	0.724061
K14187	-0.03132	0.607741	0.724935
K00840	-0.03131	0.60784	0.724935
K09518	0.031281	0.608161	0.725192
K00949	0.031248	0.608323	0.72526
K13583	-0.03125	0.608558	0.725414

K15012	0.031236	0.608676	0.72543
K07109	0.031201	0.60887	0.725536
K12528	0.031178	0.609127	0.725716
K09980	-0.03112	0.610014	0.726648
K01005	0.031078	0.610284	0.726844
K04485	0.03106	0.610492	0.726966
K00378	-0.03103	0.610776	0.727179
K13979	0.031023	0.611131	0.727225
K11929	-0.03104	0.610972	0.727225
K12518	-0.03103	0.611067	0.727225
K04065	0.030978	0.611648	0.727465
K10540	0.030972	0.611497	0.727465
K14744	-0.03098	0.611638	0.727465
K02167	-0.03095	0.611951	0.7277
K01414	-0.03088	0.61258	0.728322
K03648	-0.03085	0.612955	0.728537
K09801	-0.03086	0.612971	0.728537
K07505	0.030727	0.614537	0.730111
K12339	-0.03072	0.614613	0.730111
K13444	-0.03073	0.61445	0.730111
K01601	0.030652	0.615398	0.730918
K05565	-0.03061	0.615828	0.731302
K01455	0.03058	0.616014	0.731397
K09716	-0.03057	0.616378	0.731703
K14165	-0.03052	0.61697	0.73228
K13955	-0.03049	0.617278	0.732477
K06074	-0.03047	0.617454	0.732477
K09934	-0.03048	0.617432	0.732477
K01442	-0.0304	0.618103	0.733121
K01617	0.030382	0.618519	0.733262
K06977	0.030371	0.618641	0.733262
K07174	0.030371	0.618647	0.733262
K12785	-0.03039	0.618458	0.733262
K06337	0.030334	0.619074	0.733642
K09802	0.030301	0.619458	0.733972
K01142	0.030253	0.619791	0.734122
K01780	-0.03025	0.619798	0.734122
K07393	0.030205	0.620356	0.734658
K02803	0.030167	0.620796	0.734842
K05551	-0.03018	0.620831	0.734842
K14941	-0.03018	0.620643	0.734842
K05786	-0.0301	0.621558	0.735305
K07718	-0.03009	0.621648	0.735305
K06892	-0.03013	0.621483	0.735305
K11084	-0.03013	0.621482	0.735305
K10811	-0.03007	0.622102	0.735715
K00119	0.030017	0.622736	0.736165
K01199	-0.02999	0.622802	0.736165
K02046	0.029999	0.622739	0.736165
K09805	0.029999	0.622948	0.736211
K02058	0.029968	0.623096	0.73626
K01608	-0.02977	0.625553	0.73869

K06926	0.029754	0.625581	0.73869
K08989	-0.02978	0.625536	0.73869
K11923	-0.02976	0.625504	0.73869
K07452	0.029671	0.626548	0.739706
K07722	-0.02955	0.627902	0.740941
K11996	0.029553	0.627916	0.740941
K14519	0.029579	0.627832	0.740941
K00762	-0.02955	0.628142	0.741081
K08351	-0.0295	0.628711	0.741626
K11061	-0.02947	0.629112	0.741971
K01258	-0.0294	0.629657	0.742488
K09890	0.029395	0.629971	0.742731
K00260	0.029352	0.630262	0.74282
K01031	0.029373	0.63023	0.74282
K00087	0.02933	0.630515	0.742991
K12661	-0.02929	0.630944	0.74337
K03628	-0.02927	0.631183	0.743524
K02038	0.029159	0.632512	0.744837
K10906	-0.02918	0.632502	0.744837
K10934	-0.029	0.634601	0.747169
K07352	0.028965	0.634991	0.7475
K00449	-0.02894	0.635237	0.747662
K03607	-0.02893	0.635429	0.747761
K12284	0.028831	0.636557	0.74896
K02345	-0.0288	0.636875	0.749207
K03146	0.028758	0.637406	0.749704
K03804	0.028735	0.637679	0.749897
K08658	0.028681	0.638309	0.750511
K08737	0.02859	0.639172	0.751398
K06152	0.028594	0.639329	0.751454
K07245	-0.02858	0.639473	0.751496
K12468	-0.02853	0.640117	0.751868
K05826	0.028527	0.640114	0.751868
K12441	0.028527	0.640114	0.751868
K10193	0.028495	0.640283	0.751935
K02312	-0.02848	0.640664	0.752126
K12985	0.028486	0.640593	0.752126
K09910	0.028457	0.640935	0.752278
K13021	-0.02845	0.641011	0.752278
K02858	0.028424	0.641329	0.752523
K07542	0.028381	0.641827	0.752852
K00695	0.028385	0.641786	0.752852
K04775	-0.02831	0.642674	0.753077
K04353	0.028316	0.642597	0.753077
K04437	0.028316	0.642597	0.753077
K05535	0.028316	0.642597	0.753077
K07941	0.028316	0.642597	0.753077
K12490	0.028316	0.642597	0.753077
K01973	0.028184	0.644146	0.753655
K06222	-0.0282	0.643725	0.753655
K07356	-0.02822	0.64376	0.753655
K07646	-0.02817	0.64408	0.753655

K09127	0.028197	0.64399	0.753655
K13641	-0.02821	0.643669	0.753655
K08347	-0.02818	0.64415	0.753655
K14393	-0.02821	0.643855	0.753655
K08295	-0.02824	0.643458	0.753655
K07400	-0.02816	0.644395	0.7538
K05990	0.028154	0.644492	0.7538
K01146	-0.02814	0.644702	0.753918
K00811	-0.02809	0.645024	0.754006
K09908	0.028102	0.645105	0.754006
K13237	0.028115	0.64495	0.754006
K03669	-0.02805	0.645671	0.75454
K01658	-0.02799	0.646245	0.755083
K06191	-0.02797	0.64662	0.755266
K03895	0.027978	0.646562	0.755266
K00042	-0.02791	0.647204	0.75582
K02464	-0.02789	0.647624	0.756182
K00978	-0.0278	0.648512	0.756395
K01512	-0.0278	0.648653	0.756395
K02553	0.027844	0.648144	0.756395
K04067	-0.02778	0.648683	0.756395
K07105	0.027789	0.648591	0.756395
K01061	-0.02783	0.648261	0.756395
K10027	-0.02785	0.648129	0.756395
K13063	0.027827	0.648346	0.756395
K00481	0.02776	0.649137	0.756797
K09907	-0.02765	0.650467	0.758219
K07690	0.027596	0.651072	0.758796
K02529	0.027491	0.652108	0.759876
K10113	0.027498	0.652229	0.759888
K12204	-0.02748	0.652498	0.760073
K01494	-0.02738	0.653414	0.760626
K02786	0.027393	0.653271	0.760626
K07141	-0.02739	0.653285	0.760626
K07685	0.027401	0.65338	0.760626
K02498	-0.02733	0.65422	0.761358
K10118	-0.02731	0.654263	0.761358
K11312	-0.02729	0.654634	0.761589
K09611	0.027291	0.654682	0.761589
K13936	0.027241	0.655265	0.762139
K02689	0.027213	0.655603	0.762356
K13726	0.027207	0.655672	0.762356
K09960	0.02718	0.655798	0.762374
K03927	-0.02707	0.657142	0.763808
K12064	-0.02704	0.657596	0.764185
K13657	0.027037	0.657689	0.764185
K00867	0.027013	0.657972	0.764351
K01646	-0.02699	0.658072	0.764351
K00500	-0.027	0.658163	0.764351
K11903	-0.02698	0.6584	0.764497
K13020	0.026911	0.658988	0.765052
K13714	0.026886	0.659282	0.765264

K01172	0.026884	0.659505	0.765298
K05588	-0.02688	0.659533	0.765298
K06907	-0.02681	0.660134	0.765738
K02402	-0.02683	0.660096	0.765738
K09817	0.02679	0.660428	0.76595
K06206	-0.02672	0.661281	0.76681
K03676	0.026589	0.663003	0.768679
K00104	-0.02651	0.663749	0.769339
K05299	-0.02652	0.663796	0.769339
K11937	-0.02646	0.664544	0.769818
K02259	0.026475	0.664368	0.769818
K09941	0.026464	0.664497	0.769818
K09909	-0.02645	0.664698	0.769867
K03221	-0.02643	0.664882	0.769952
K01182	0.026396	0.665107	0.770082
K01733	0.026342	0.665761	0.77071
K00065	-0.02632	0.666055	0.770793
K05581	0.026338	0.665999	0.770793
K10748	-0.02631	0.666309	0.770828
K15039	-0.02632	0.666242	0.770828
K05972	-0.02614	0.668314	0.772888
K09000	0.026144	0.668311	0.772888
K09959	0.026102	0.668815	0.773338
K07006	0.026073	0.66897	0.773388
K03823	0.026056	0.669164	0.773483
K13531	0.026049	0.669445	0.773644
K05749	-0.02604	0.669528	0.773644
K00574	-0.02601	0.669719	0.773735
K11742	-0.02599	0.670159	0.774115
K09930	-0.02595	0.670587	0.774479
K01387	0.025927	0.670904	0.774716
K12068	-0.02591	0.671097	0.774809
K02298	-0.02585	0.671772	0.775459
K07711	-0.02584	0.671917	0.775496
K10209	0.02583	0.672062	0.775534
K14762	-0.0258	0.672393	0.775786
K00111	0.025755	0.672953	0.776302
K12974	-0.02573	0.673273	0.776542
K01625	-0.02567	0.673787	0.777005
K07304	-0.02564	0.674091	0.777162
K05545	-0.02566	0.674148	0.777162
K03239	0.025641	0.674326	0.777237
K01619	0.025605	0.67456	0.777377
K00517	-0.02561	0.674735	0.777449
K03764	0.025597	0.674848	0.77745
K02037	0.025564	0.675059	0.777562
K02844	-0.02555	0.675269	0.777674
K11607	0.025536	0.675575	0.777898
K00483	0.025455	0.676549	0.778594
K04767	-0.02542	0.676744	0.778594
K10972	-0.02546	0.676505	0.778594
K06078	0.025448	0.676639	0.778594

K14191	0.025476	0.676302	0.778594
K02509	0.025365	0.677631	0.779484
K05304	0.025331	0.678036	0.779821
K01457	-0.0253	0.678177	0.779852
K03274	0.025259	0.678713	0.780092
K10200	-0.02527	0.678524	0.780092
K11180	0.025274	0.678724	0.780092
K11958	-0.02525	0.67902	0.780302
K06146	-0.0252	0.67961	0.78085
K06933	0.025151	0.680017	0.781187
K03645	0.025131	0.680436	0.781409
K10832	0.025134	0.680405	0.781409
K07057	-0.0251	0.680761	0.781652
K12534	-0.02508	0.681085	0.781894
K03314	0.025029	0.681671	0.782437
K00681	-0.02494	0.682512	0.783271
K13157	0.02492	0.682974	0.783672
K07355	0.024782	0.684641	0.785454
K00786	-0.02472	0.685192	0.785955
K11912	0.02467	0.685802	0.786525
K00826	-0.0246	0.686697	0.786711
K06917	0.024631	0.686463	0.786711
K07507	0.024598	0.686675	0.786711
K02255	-0.02464	0.686359	0.786711
K07018	-0.02461	0.686706	0.786711
K02736	0.024607	0.686747	0.786711
K09147	0.024606	0.686762	0.786711
K10984	0.024587	0.686986	0.786837
K00879	0.024542	0.687352	0.787092
K12150	0.02455	0.687437	0.787092
K13611	0.024509	0.687752	0.787322
K09961	0.024451	0.688444	0.787983
K11935	0.024456	0.688573	0.788
K01777	0.024378	0.689332	0.788564
K12165	0.024401	0.689236	0.788564
K08150	-0.02439	0.689408	0.788564
K11893	-0.0243	0.690428	0.7896
K12788	-0.0242	0.691719	0.790946
K07120	-0.02411	0.692584	0.791722
K11738	0.02412	0.692628	0.791722
K01952	-0.02406	0.693117	0.79215
K02379	-0.02405	0.693256	0.792164
K14145	-0.02406	0.693359	0.792164
K14635	0.02403	0.693723	0.792442
K03970	-0.02402	0.693832	0.792442
K02615	0.023977	0.694364	0.792919
K09578	0.023964	0.694525	0.792971
K03081	-0.02395	0.694683	0.793021
K01460	0.023924	0.694998	0.793249
K07184	-0.02391	0.695112	0.793249
K02221	-0.02386	0.695564	0.793633
K10939	0.023839	0.696031	0.794035

K00881	0.0238	0.696332	0.794111
K13532	-0.02381	0.696443	0.794111
K12784	-0.02381	0.696367	0.794111
K12420	-0.02379	0.696633	0.794196
K02572	0.023737	0.697092	0.794588
K05877	0.023713	0.697559	0.79499
K06006	0.023692	0.697812	0.795146
K04334	-0.02363	0.698587	0.795898
K07770	0.023598	0.698775	0.795981
K06121	0.023589	0.699063	0.796177
K07653	-0.02356	0.699408	0.79636
K11920	-0.02356	0.699454	0.79636
K07639	0.023534	0.699735	0.796429
K09999	0.023533	0.699745	0.796429
K11107	0.023502	0.700124	0.796729
K02525	-0.02347	0.700306	0.796805
K06075	0.023405	0.701127	0.797607
K07487	0.023324	0.70211	0.798457
K07659	0.02331	0.702278	0.798457
K04039	0.02332	0.702337	0.798457
K15019	-0.02334	0.702114	0.798457
K08353	-0.02329	0.70271	0.79875
K10805	-0.02326	0.703104	0.799066
K13789	0.023177	0.7039	0.799839
K00324	-0.02309	0.704943	0.80081
K02589	-0.02306	0.705496	0.80081
K03647	0.02304	0.705567	0.80081
K06899	0.023092	0.705104	0.80081
K09698	-0.02305	0.705405	0.80081
K14274	-0.02307	0.705346	0.80081
K07136	-0.02309	0.705078	0.80081
K07309	-0.02303	0.705838	0.800986
K05552	0.023016	0.706032	0.801075
K05373	-0.02297	0.706555	0.801536
K11917	-0.02295	0.706839	0.801727
K07315	0.022876	0.707566	0.802377
K09683	0.022884	0.707645	0.802377
K07152	-0.02285	0.708105	0.802768
K00079	0.022821	0.708236	0.802784
K02191	-0.02282	0.708447	0.802891
K10986	0.022772	0.709006	0.803128
K06653	0.022791	0.708778	0.803128
K05355	-0.02278	0.708903	0.803128
K01426	0.022732	0.709325	0.803359
K07004	0.02271	0.70959	0.803527
K02756	0.022683	0.709914	0.803762
K04022	-0.02265	0.710468	0.804137
K13832	-0.02265	0.710478	0.804137
K06213	0.02262	0.710687	0.804242
K05564	-0.02262	0.710813	0.804253
K07503	0.022609	0.710997	0.804328
K00932	-0.02258	0.711312	0.804553

K03215	0.022557	0.711461	0.804589
K02560	-0.02252	0.712025	0.804964
K08372	-0.02252	0.711918	0.804964
K02819	-0.02249	0.712499	0.805368
K08996	0.022466	0.712739	0.805375
K06638	0.022474	0.712643	0.805375
K11017	-0.02244	0.713008	0.805548
K02742	-0.02238	0.713746	0.80625
K05577	-0.02232	0.714575	0.807054
K06934	0.022257	0.715126	0.807544
K01941	-0.02223	0.715444	0.807771
K04062	-0.02223	0.715675	0.807899
K08221	-0.02216	0.716264	0.808299
K14087	0.02217	0.71619	0.808299
K08324	-0.02217	0.716424	0.808349
K01653	-0.02207	0.717431	0.808989
K11384	-0.02207	0.71746	0.808989
K14979	-0.02209	0.717378	0.808989
K12662	0.022084	0.717412	0.808989
K10211	0.022069	0.717596	0.80901
K11023	-0.02205	0.717791	0.809097
K06159	-0.02203	0.718115	0.80933
K11083	-0.02196	0.718919	0.810104
K03100	-0.02192	0.71925	0.810345
K07682	0.021918	0.719453	0.810442
K13005	-0.02187	0.720058	0.81099
K07312	-0.02181	0.72075	0.811505
K04090	-0.02182	0.7207	0.811505
K14065	-0.02178	0.7212	0.811879
K08348	-0.02176	0.721335	0.811899
K09898	-0.02175	0.721535	0.811992
K00855	0.021735	0.721694	0.812038
K00917	0.021682	0.722344	0.8123
K01631	0.021668	0.722515	0.8123
K03214	0.021702	0.722104	0.8123
K09767	-0.02168	0.722262	0.8123
K07156	-0.02167	0.722439	0.8123
K02482	0.021537	0.723967	0.813464
K04063	-0.02154	0.724073	0.813464
K07212	0.021527	0.724085	0.813464
K12532	-0.02154	0.724139	0.813464
K10799	-0.02156	0.723804	0.813464
K09904	0.021454	0.72515	0.814466
K09915	-0.02142	0.725626	0.814771
K08176	0.021413	0.725657	0.814771
K10441	0.021385	0.725828	0.814829
K12060	-0.02139	0.725945	0.814829
K15011	0.021378	0.726088	0.814857
K09943	0.02135	0.726428	0.815106
K03750	0.021327	0.726548	0.815108
K03112	-0.0213	0.727049	0.815486
K05712	-0.02129	0.727121	0.815486

K02172	0.021244	0.727565	0.815852
K03528	0.021219	0.72804	0.815913
K06864	-0.02121	0.727937	0.815913
K12072	-0.02121	0.728172	0.815913
K13766	0.021205	0.728211	0.815913
K05927	0.021214	0.728101	0.815913
K08317	-0.02118	0.728484	0.816087
K07669	-0.0211	0.729363	0.816939
K03484	-0.02107	0.729742	0.817231
K11003	-0.02106	0.729965	0.817347
K00031	0.021033	0.730166	0.817374
K00850	-0.02103	0.730225	0.817374
K13256	-0.02102	0.730525	0.817577
K06377	-0.021	0.730686	0.817625
K00575	-0.02094	0.731266	0.818009
K03971	-0.02096	0.731265	0.818009
K01776	0.020896	0.732024	0.818723
K09901	0.020883	0.732178	0.818763
K09824	0.020868	0.732369	0.818844
K12678	-0.02076	0.733658	0.820152
K07637	-0.02074	0.733965	0.820363
K01032	-0.02071	0.734335	0.820644
K07300	-0.02067	0.734776	0.821003
K01264	-0.02065	0.734914	0.821025
K06726	-0.02061	0.735399	0.821279
K05957	0.020614	0.735498	0.821279
K09804	0.02062	0.735429	0.821279
K01841	0.02054	0.736256	0.821992
K13639	-0.02051	0.736844	0.822515
K10041	-0.02048	0.737039	0.8226
K05835	-0.02048	0.737161	0.822604
K06921	-0.02042	0.737688	0.823058
K11081	0.020418	0.737919	0.823182
K07489	0.020395	0.738053	0.823199
K01220	0.020369	0.738367	0.823416
K03298	-0.02035	0.738665	0.82345
K07013	0.020328	0.738874	0.82345
K10237	-0.02035	0.738785	0.82345
K12689	-0.02035	0.738792	0.82345
K02395	-0.0203	0.73918	0.823658
K03586	-0.02029	0.739458	0.823834
K02574	-0.02026	0.739755	0.824032
K05560	-0.02024	0.740105	0.824289
K14201	-0.02023	0.740296	0.824369
K02244	-0.0202	0.740627	0.824515
K13085	0.020187	0.740785	0.824515
K00696	-0.02019	0.740712	0.824515
K07349	-0.02016	0.741136	0.824772
K04046	-0.0201	0.741844	0.825427
K06203	0.020043	0.742563	0.825905
K08299	-0.02003	0.742732	0.825905
K09160	-0.02005	0.742531	0.825905

K14052	-0.02	0.74299	0.825905	
K09916	-0.02	0.743111	0.825905	
K01477	0.020015	0.742915	0.825905	
K06136	0.020003	0.743067	0.825905	
K12228	-0.01995	0.743728	0.826457	
K02440	0.019914	0.744007	0.826635	
K03854	0.019907	0.744253	0.826774	
K08646	0.019856	0.744883	0.827341	
K11636	0.019726	0.7465	0.829004	
K03788	0.019705	0.74676	0.82916	
K03031	0.01968	0.747065	0.829364	
K11745	-0.01966	0.747259	0.829447	
K14763	0.01965	0.747445	0.829519	
K09920	-0.01962	0.747874	0.829863	
K08696	-0.0196	0.748061	0.829937	
K07188	-0.01959	0.74824	0.830002	
K11905	-0.01954	0.748765	0.830451	
K11102	0.019516	0.74895	0.830522	
K07153	-0.01948	0.74952	0.831021	
K03836	-0.01946	0.749785	0.831048	
K13622	0.019469	0.749683	0.831048	
K13171	0.019448	0.749947	0.831094	
K08075	0.019438	0.75007	0.831097	
K02847	-0.01934	0.751262	0.832284	
K12810	-0.01931	0.751618	0.832544	
K00406	0.019276	0.752091	0.832935	
K01974	-0.01924	0.752534	0.833025	
K03780	-0.01924	0.752355	0.833025	
K14475	0.019238	0.752415	0.833025	
K02839	-0.01917	0.753234	0.833532	
K01669	-0.01919	0.753145	0.833532	
K02746	-0.01915	0.753474	0.833664	
K00900	0.019152	0.753635	0.833709	
K07310	0.019122	0.754	0.833979	
K13338	0.019098	0.754306	0.834184	
K08193	0.019076	0.754577	0.83435	
K02132	0.019065	0.754713	0.834366	
K14371	0.018988	0.755676	0.835297	
K04033	0.018893	0.756857	0.836341	
K13849	-0.01889	0.756863	0.836341	
K00138	-0.01876	0.758492	0.837873	
K05526	-0.01877	0.758425	0.837873	
K06867	0.018721	0.758857	0.838143	
K02241	0.018675	0.759429	0.83864	
K01120	-0.01863	0.760002	0.838942	
K13668	-0.01864	0.759911	0.838942	
K11059	0.018636	0.760067	0.838942	
K03336	0.018573	0.760702	0.839509	
K06937	0.01853	0.761244	0.839905	
K13894	-0.01853	0.761364	0.839905	
K01513	0.018527	0.761426	0.839905	
K00556	0.01849	0.761742	0.840119	

K00066	-0.01844	0.762454	0.84077
K09844	0.018402	0.762989	0.841226
K13419	-0.01837	0.763325	0.841462
K08302	-0.01834	0.763634	0.841668
K03535	0.018321	0.76386	0.841753
K09883	0.018311	0.763981	0.841753
K11045	0.018315	0.764077	0.841753
K05338	0.018292	0.76436	0.841931
K03407	-0.0182	0.765354	0.842815
K07021	0.018208	0.765408	0.842815
K07479	-0.01819	0.765643	0.84294
K05799	-0.01808	0.766811	0.844091
K04018	-0.01808	0.767062	0.844233
K01521	-0.018	0.768068	0.845205
K09896	-0.01791	0.769099	0.846205
K01096	0.017799	0.770392	0.847027
K02302	-0.0178	0.770354	0.847027
K08352	-0.01779	0.77046	0.847027
K11940	-0.01782	0.770271	0.847027
K13793	-0.01783	0.770191	0.847027
K05994	-0.01774	0.771287	0.847699
K14998	-0.01774	0.771316	0.847699
K01644	-0.01767	0.772056	0.848376
K06873	0.017617	0.772811	0.849071
K02551	-0.01757	0.773463	0.849517
K12062	-0.01757	0.773394	0.849517
K00705	0.017489	0.774281	0.85001
K05662	0.017509	0.774171	0.85001
K08084	0.017513	0.774121	0.85001
K07389	0.017429	0.775172	0.850852
K00612	-0.01738	0.775644	0.851236
K04786	-0.01733	0.776228	0.85174
K13620	-0.01731	0.776677	0.852098
K04087	0.01722	0.77766	0.852787
K10678	-0.01722	0.777675	0.852787
K12058	0.017233	0.777642	0.852787
K11214	0.017182	0.778138	0.853158
K01126	-0.01716	0.778403	0.853313
K12530	0.017162	0.778526	0.853313
K10227	0.017114	0.779128	0.853837
K01950	-0.01705	0.779822	0.854055
K02682	0.017068	0.779709	0.854055
K06683	-0.01706	0.779781	0.854055
K11909	0.017069	0.779701	0.854055
K00059	0.016971	0.780793	0.854712
K00928	-0.01697	0.780747	0.854712
K01568	-0.01698	0.780787	0.854712
K00412	-0.01694	0.781272	0.855057
K11030	0.016937	0.781356	0.855057
K09022	0.016913	0.781522	0.855103
K03926	0.016904	0.781776	0.85511
K09523	0.016907	0.781737	0.85511

K03776	-0.01686	0.782375	0.855573
K07804	-0.01685	0.782447	0.855573
K01322	-0.01679	0.783041	0.855951
K10023	0.016809	0.782969	0.855951
K12444	-0.01677	0.783497	0.856314
K07733	-0.0167	0.784367	0.85713
K00667	-0.01662	0.78538	0.857445
K03855	-0.01665	0.784841	0.857445
K04750	0.016616	0.785401	0.857445
K07301	0.016618	0.785237	0.857445
K09917	-0.01664	0.785152	0.857445
K03812	-0.01665	0.784981	0.857445
K11231	0.016584	0.785801	0.857746
K05889	0.016494	0.786936	0.858849
K00247	0.016428	0.787775	0.859628
K12658	-0.0164	0.788082	0.859828
K00511	-0.01638	0.788394	0.860032
K14379	-0.01634	0.788923	0.860474
K03290	0.016277	0.789673	0.860571
K06902	-0.01625	0.789885	0.860571
K06971	-0.01625	0.789885	0.860571
K07091	-0.01629	0.789406	0.860571
K07237	-0.0163	0.789418	0.860571
K14581	0.016263	0.789855	0.860571
K12212	0.016307	0.789294	0.860571
K07671	0.016242	0.790113	0.860611
K14451	0.016238	0.790171	0.860611
K10908	0.016227	0.790308	0.860624
K07496	-0.01604	0.792483	0.862651
K10563	-0.01602	0.792734	0.862651
K12061	-0.01605	0.792488	0.862651
K07229	-0.01603	0.792794	0.862651
K08319	0.016034	0.792747	0.862651
K09701	0.01594	0.793933	0.863754
K01293	-0.01591	0.794315	0.864033
K09117	0.015865	0.794747	0.864367
K10022	0.015821	0.795429	0.864973
K13875	-0.01572	0.79669	0.866208
K09816	0.015694	0.796914	0.866315
K01274	0.015618	0.797883	0.866652
K03498	-0.0156	0.798082	0.866652
K07658	0.015606	0.798029	0.866652
K08261	-0.01561	0.798078	0.866652
K05710	0.01561	0.798103	0.866652
K11139	-0.01564	0.797748	0.866652
K06122	-0.01563	0.797897	0.866652
K15053	0.015567	0.798646	0.867104
K00523	-0.01553	0.799106	0.867468
K04064	-0.01549	0.799681	0.867801
K07354	0.015467	0.799916	0.867801
K04110	0.01548	0.799754	0.867801
K05548	-0.01548	0.799792	0.867801

K03980	-0.01538	0.800893	0.868725
K02457	-0.01533	0.80163	0.869387
K01083	0.015226	0.802971	0.870705
K00220	-0.01516	0.803806	0.871474
K06204	0.015101	0.804426	0.871872
K10837	0.015117	0.804346	0.871872
K05527	0.015091	0.804681	0.871875
K06216	0.015098	0.804597	0.871875
K14598	0.014928	0.80675	0.87398
K07662	-0.0149	0.806967	0.874077
K00154	0.014885	0.807289	0.874289
K07446	0.014864	0.807434	0.874309
K03532	-0.01486	0.807623	0.874376
K06351	-0.01483	0.807979	0.874624
K06882	0.014807	0.808162	0.874631
K08978	-0.0148	0.808238	0.874631
K03772	-0.01477	0.808729	0.875025
K10938	0.014731	0.809248	0.875449
K03194	0.014719	0.809395	0.875471
K04013	0.014706	0.809571	0.875524
K11181	-0.01467	0.810052	0.875907
K07279	-0.01463	0.81043	0.876178
K05554	0.014607	0.81083	0.876473
K02591	-0.01458	0.811112	0.876504
K10231	-0.01458	0.81102	0.876504
K07782	-0.01448	0.81243	0.87779
K07353	0.014464	0.812646	0.877887
K05973	0.014447	0.812853	0.877973
K06879	-0.01442	0.81306	0.878059
K10560	0.014406	0.81326	0.878137
K02297	-0.01439	0.813632	0.878265
K05803	0.014396	0.813512	0.878265
K07058	0.014357	0.813881	0.878396
K03674	-0.01434	0.814188	0.87859
K11106	0.01431	0.814605	0.878902
K00266	-0.01428	0.814887	0.879069
K10843	0.014208	0.815785	0.879901
K11744	0.014195	0.816057	0.880057
K01671	-0.01403	0.818124	0.882147
K09893	0.01402	0.818288	0.882187
K10902	0.014003	0.818511	0.882289
K10554	-0.01396	0.819111	0.882798
K02846	0.013918	0.819593	0.88318
K02757	-0.0139	0.819805	0.88327
K07751	0.013889	0.819962	0.883301
K08225	-0.01382	0.820687	0.883945
K12437	0.013816	0.820891	0.884027
K12455	-0.0138	0.821074	0.884085
K03337	0.013778	0.821264	0.884089
K03779	-0.01377	0.821333	0.884089
K04032	-0.01367	0.82261	0.885325
K04340	-0.01364	0.823119	0.885735

K13831	-0.0136	0.823526	0.886017
K10953	-0.0136	0.823637	0.886017
K01790	0.013445	0.825511	0.887894
K02543	-0.01334	0.826997	0.889354
K13772	0.013305	0.827306	0.889445
K05876	-0.0133	0.827468	0.889445
K14681	0.013306	0.827398	0.889445
K11330	-0.01328	0.827748	0.889607
K13932	0.013237	0.82828	0.89004
K06039	0.013214	0.828578	0.890083
K04792	-0.01322	0.828521	0.890083
K07769	0.013121	0.829763	0.891217
K00313	-0.01304	0.830736	0.891984
K07232	-0.01305	0.830672	0.891984
K11906	-0.01298	0.831506	0.892481
K12625	-0.01298	0.831574	0.892481
K00411	0.012978	0.831586	0.892481
K01275	0.012958	0.831844	0.89262
K13895	-0.01294	0.832036	0.892687
K09989	0.012893	0.832571	0.893122
K01239	0.012863	0.832957	0.893397
K06311	-0.01281	0.83369	0.893905
K12137	0.012813	0.833598	0.893905
K07083	-0.01274	0.834642	0.894787
K02304	0.012686	0.835218	0.895266
K09897	-0.01266	0.835593	0.895528
K00008	0.012598	0.836346	0.896057
K00477	-0.01261	0.836306	0.896057
K07050	0.01259	0.836545	0.896131
K02831	0.012544	0.83714	0.89649
K03093	-0.01254	0.837135	0.89649
K12984	0.012478	0.837985	0.897256
K11328	-0.01246	0.83816	0.897304
K07396	-0.01245	0.83833	0.897347
K10538	0.012401	0.838967	0.89789
K00164	0.012331	0.839761	0.8986
K00533	0.012302	0.840139	0.898866
K09012	0.012196	0.841599	0.900288
K02749	-0.01211	0.842637	0.9007
K05303	0.012108	0.842629	0.9007
K07286	-0.01215	0.842171	0.9007
K11604	-0.01214	0.842295	0.9007
K02214	0.012119	0.84259	0.9007
K05528	-0.01206	0.843324	0.901295
K01739	-0.01194	0.844741	0.901983
K02344	0.011947	0.844785	0.901983
K03738	0.011947	0.844695	0.901983
K08364	-0.01199	0.844294	0.901983
K12063	-0.01191	0.845144	0.901983
K05575	-0.01194	0.844897	0.901983
K07119	0.011951	0.84474	0.901983
K00785	-0.01193	0.84502	0.901983

K09950	-0.01199	0.844233	0.901983
K12370	0.011895	0.845454	0.902175
K07589	-0.01181	0.846529	0.903182
K00299	-0.01177	0.847004	0.90355
K00880	-0.0117	0.847823	0.904208
K08311	-0.0117	0.847987	0.904208
K12112	-0.0117	0.848014	0.904208
K02064	0.011662	0.848452	0.90441
K11815	-0.01166	0.848466	0.90441
K13571	-0.01163	0.848922	0.904757
K00801	0.011608	0.849145	0.904855
K11811	-0.01155	0.849849	0.905326
K05797	-0.01156	0.849745	0.905326
K11953	-0.01138	0.852051	0.90753
K02747	0.011349	0.852372	0.907732
K06958	0.011302	0.852976	0.908096
K10709	-0.01131	0.852852	0.908096
K03819	0.011295	0.85316	0.908152
K12403	0.011269	0.853499	0.908372
K07160	-0.01117	0.854644	0.90917
K15161	0.011183	0.854604	0.90917
K06680	0.011197	0.854425	0.90917
K03630	-0.0111	0.855637	0.909946
K10119	0.011103	0.855536	0.909946
K02103	0.011056	0.856149	0.910351
K07271	0.01097	0.857251	0.911382
K09938	-0.01093	0.857888	0.911919
K04787	0.010912	0.858093	0.911997
K03745	-0.01088	0.85856	0.912306
K06871	0.010851	0.858781	0.912306
K10195	-0.01086	0.858712	0.912306
K02083	0.010819	0.8592	0.912611
K03761	-0.0108	0.859553	0.912808
K07485	-0.01078	0.85965	0.912808
K13000	-0.01073	0.860371	0.913433
K11901	-0.01067	0.861147	0.914117
K06195	-0.01065	0.861464	0.914312
K11258	-0.0106	0.862059	0.914662
K08605	0.010605	0.86204	0.914662
K09902	0.010555	0.862686	0.915187
K04779	0.010519	0.863147	0.915535
K03292	-0.01042	0.864383	0.916283
K09017	-0.01043	0.864181	0.916283
K13890	0.010424	0.864282	0.916283
K11227	-0.01043	0.864238	0.916283
K14055	-0.0104	0.864678	0.916455
K12239	0.010326	0.865641	0.917335
K11959	-0.01029	0.866054	0.917632
K02403	0.01028	0.866224	0.917671
K01913	0.010227	0.866825	0.918055
K07235	0.010231	0.866853	0.918055
K09121	-0.01014	0.867973	0.919101

K00674	0.010111	0.868405	0.919235	
K02079	-0.0101	0.868499	0.919235	
K02292	0.010116	0.868341	0.919235	
K13671	-0.01009	0.868714	0.919321	
K14708	0.010039	0.869252	0.91975	
K00694	0.009917	0.870824	0.921272	
K10025	-0.0099	0.871064	0.921385	
K10228	-0.00989	0.871213	0.921401	
K04023	0.009852	0.871754	0.921694	
K08587	0.009844	0.871774	0.921694	
K09914	0.009835	0.871891	0.921694	
K06293	0.009793	0.872511	0.922067	
K08992	-0.0098	0.872394	0.922067	
K02972	-0.00975	0.873109	0.922558	
K09781	0.009733	0.873283	0.922601	
K01053	0.009698	0.873659	0.922762	
K01749	-0.0097	0.873703	0.922762	
K02505	0.00967	0.874098	0.92302	
K12282	-0.00966	0.874215	0.92302	
K02703	0.009637	0.874519	0.923059	
K07881	0.009641	0.874472	0.923059	
K03499	0.009603	0.87489	0.923309	
K11186	-0.00959	0.875121	0.923412	
K01659	0.009528	0.875856	0.924046	
K03680	-0.00951	0.876145	0.924209	
K09929	-0.00949	0.876358	0.924293	
K03672	-0.00945	0.876884	0.924707	
K00947	0.009363	0.877992	0.925142	
K01354	0.009362	0.878074	0.925142	
K01953	-0.00937	0.877914	0.925142	
K02113	-0.00935	0.878101	0.925142	
K02220	-0.00938	0.877799	0.925142	
K06118	0.00937	0.877968	0.925142	
K01312	-0.00928	0.879195	0.925871	
K02802	0.009295	0.878938	0.925871	
K06605	-0.00928	0.879099	0.925871	
K00825	0.009209	0.880047	0.926627	
K07347	-0.00916	0.880673	0.926863	
K10189	0.009155	0.880675	0.926863	
K08087	-0.00917	0.880508	0.926863	
K01480	-0.00912	0.881166	0.927239	
K01042	0.009068	0.881798	0.927613	
K02587	-0.00906	0.881907	0.927613	
K02849	-0.00906	0.882028	0.927613	
K13771	-0.00903	0.88225	0.927613	
K02463	-0.00903	0.882327	0.927613	
K03381	-0.00904	0.882234	0.927613	
K03327	0.009006	0.882609	0.927627	
K10710	0.009007	0.882586	0.927627	
K00859	0.008951	0.883319	0.927809	
K02417	0.008966	0.883124	0.927809	
K08697	0.008969	0.883152	0.927809	

K11269	0.008956	0.88332	0.927809
K07281	-0.00894	0.883522	0.92788
K07686	-0.00892	0.883829	0.92792
K12983	-0.00892	0.883829	0.92792
K00619	-0.00889	0.884233	0.928203
K02806	0.008861	0.884482	0.928323
K02672	-0.00884	0.88482	0.928395
K02597	-0.00884	0.884794	0.928395
K12583	0.008821	0.884998	0.928441
K11491	0.008725	0.886315	0.929681
K00395	-0.00852	0.888902	0.930151
K00878	-0.00867	0.886989	0.930151
K01453	-0.00856	0.888324	0.930151
K02082	-0.00865	0.88727	0.930151
K03191	-0.00852	0.888879	0.930151
K04044	-0.00854	0.888637	0.930151
K07358	0.008528	0.888866	0.930151
K09729	-0.00857	0.888347	0.930151
K13052	0.008612	0.887699	0.930151
K00228	-0.00859	0.887997	0.930151
K05839	-0.00852	0.888918	0.930151
K05830	0.008533	0.888802	0.930151
K12789	0.008633	0.887498	0.930151
K13743	0.008633	0.887498	0.930151
K12080	-0.00856	0.888485	0.930151
K01545	0.008557	0.888485	0.930151
K00610	-0.00849	0.88934	0.930348
K00835	0.008467	0.889651	0.930348
K06205	0.008468	0.889644	0.930348
K07339	0.008455	0.889746	0.930348
K08652	-0.00846	0.889781	0.930348
K10005	-0.00842	0.89016	0.930604
K00929	0.00838	0.890707	0.931034
K01458	-0.00835	0.891161	0.931353
K10221	-0.00834	0.891282	0.931353
K01417	-0.00831	0.891559	0.931499
K01515	-0.0083	0.891715	0.931499
K01756	0.008278	0.892103	0.931499
K14654	-0.00826	0.892231	0.931499
K00404	0.008288	0.891974	0.931499
K00493	-0.00828	0.892047	0.931499
K13933	-0.00824	0.892583	0.931725
K01679	0.008179	0.893318	0.932159
K07445	-0.00817	0.893404	0.932159
K06171	-0.0082	0.89314	0.932159
K01589	-0.00815	0.893748	0.932377
K07346	0.008121	0.894136	0.932641
K03597	-0.00808	0.894715	0.932974
K09994	0.00807	0.894725	0.932974
K03633	0.008059	0.894946	0.933063
K00179	-0.00797	0.895984	0.933248
K09834	-0.00798	0.895937	0.933248

K14161	-0.00797	0.89607	0.933248	
K07140	-0.008	0.895655	0.933248	
K03774	-0.00803	0.895298	0.933248	
K08967	0.008011	0.895563	0.933248	
K14430	0.007998	0.895725	0.933248	
K09021	0.007914	0.896751	0.933676	
K12786	-0.00792	0.8967	0.933676	
K02656	0.007853	0.897541	0.934217	
K02681	-0.00787	0.897423	0.934217	
K07688	0.007808	0.898198	0.934645	
K01638	-0.00781	0.898223	0.934645	
K01036	0.007682	0.899763	0.936104	
K07704	0.007672	0.899896	0.936104	
K06370	-0.00767	0.900047	0.936119	
K07687	0.007616	0.900686	0.936429	
K08088	0.007611	0.900752	0.936429	
K03016	0.00762	0.900626	0.936429	
K15036	-0.00752	0.901892	0.937474	
K09007	0.007408	0.903317	0.938814	
K14089	0.007375	0.903748	0.93912	
K12371	-0.00736	0.903995	0.939143	
K00499	0.007357	0.904042	0.939143	
K01283	-0.00728	0.904962	0.939251	
K03598	0.007289	0.904925	0.939251	
K06016	-0.00731	0.904578	0.939251	
K08306	0.007288	0.90494	0.939251	
K11533	-0.0073	0.904721	0.939251	
K08740	0.007286	0.904963	0.939251	
K04127	-0.00718	0.906403	0.940605	
K01834	-0.00716	0.906583	0.94065	
K14584	0.007109	0.90726	0.941211	
K00030	0.00704	0.908103	0.941766	
K03299	0.007042	0.908072	0.941766	
K05782	-0.00704	0.908205	0.941766	
K02545	0.007016	0.908417	0.941778	
K14160	0.007011	0.908537	0.941778	
K02634	0.007004	0.908626	0.941778	
K01219	0.006935	0.909467	0.942367	
K07666	-0.00694	0.909441	0.942367	
K02557	-0.00691	0.909835	0.942608	
K01502	0.006894	0.910057	0.942695	
K09476	-0.00685	0.910569	0.943084	
K01266	-0.00675	0.911905	0.943399	
K02626	-0.00678	0.911557	0.943399	
K03399	-0.00675	0.911877	0.943399	
K03796	-0.0068	0.911273	0.943399	
K03932	-0.00675	0.911897	0.943399	
K13527	-0.00679	0.911447	0.943399	
K08993	-0.00675	0.911966	0.943399	
K15083	-0.00677	0.911731	0.943399	
K02745	0.006688	0.912681	0.943997	
K07297	-0.00665	0.913237	0.944431	

K02593	0.006599	0.913894	0.944827
K10020	0.006599	0.913894	0.944827
K03616	0.006538	0.914634	0.944955
K04768	-0.00656	0.914451	0.944955
K10006	-0.00654	0.914702	0.944955
K05711	0.006559	0.914408	0.944955
K10764	0.006561	0.914385	0.944955
K13017	-0.0065	0.915167	0.945294
K00906	-0.00647	0.915519	0.945424
K13935	-0.00647	0.915567	0.945424
K07432	-0.0064	0.91642	0.946163
K00180	-0.00636	0.916917	0.946169
K00568	-0.00636	0.916885	0.946169
K02667	-0.00637	0.916768	0.946169
K05559	0.006362	0.916974	0.946169
K02680	0.006238	0.918582	0.947403
K09892	0.006249	0.918442	0.947403
K14090	0.006243	0.91847	0.947403
K02610	-0.00622	0.918866	0.947555
K07272	0.006177	0.919326	0.947729
K09911	0.006172	0.919447	0.947729
K13244	0.006175	0.919349	0.947729
K04762	-0.00615	0.919679	0.947827
K00354	-0.00613	0.91996	0.947975
K08137	0.006112	0.920228	0.948109
K07806	0.006092	0.920432	0.948179
K02450	0.005919	0.922685	0.94988
K02778	-0.00591	0.922772	0.94988
K05789	-0.00592	0.922706	0.94988
K02336	-0.00592	0.922678	0.94988
K11226	0.00594	0.922459	0.94988
K03717	0.005866	0.923376	0.950219
K15226	0.005873	0.923327	0.950219
K01259	-0.00584	0.923698	0.950408
K01055	0.005809	0.924115	0.950695
K07068	-0.0058	0.924267	0.95071
K08700	0.005719	0.925341	0.95139
K09470	0.005727	0.925236	0.95139
K13796	0.005727	0.925235	0.95139
K02288	0.005657	0.926139	0.952069
K06951	0.005625	0.92651	0.952308
K09912	-0.00559	0.927033	0.952703
K03518	-0.00555	0.927508	0.952766
K07451	0.005564	0.927355	0.952766
K12069	-0.00556	0.927377	0.952766
K07218	0.005489	0.928338	0.953476
K02301	-0.00543	0.929118	0.953994
K00470	-0.00543	0.929072	0.953994
K10246	0.005379	0.929764	0.954516
K11606	0.005316	0.930592	0.954998
K00137	-0.00531	0.930649	0.954998
K11016	-0.00532	0.930478	0.954998

K10380	0.00527	0.931181	0.955402
K01736	-0.00523	0.931649	0.955739
K13542	-0.0052	0.932081	0.956041
K14088	0.005073	0.933716	0.957575
K05576	0.005052	0.934026	0.957751
K09940	0.005015	0.93451	0.958105
K02565	0.004972	0.935029	0.958315
K03566	0.004973	0.935013	0.958315
K13607	0.004964	0.935131	0.958315
K09477	0.004953	0.935315	0.958361
K13651	0.004937	0.935529	0.958438
K06290	-0.00491	0.935874	0.958649
K12552	-0.00489	0.936109	0.958747
K00372	0.004849	0.936668	0.959178
K03457	0.00478	0.937529	0.959918
K06042	0.00473	0.938182	0.960443
K00787	0.004678	0.938896	0.961032
K03791	-0.00464	0.939378	0.961382
K02588	-0.00455	0.940542	0.962102
K08174	-0.00453	0.940778	0.962102
K10000	0.004544	0.940649	0.962102
K12151	0.004567	0.94034	0.962102
K02671	-0.00456	0.940485	0.962102
K02494	-0.00446	0.941749	0.96281
K02425	0.004465	0.94167	0.96281
K03808	-0.00442	0.942225	0.963154
K02019	-0.00437	0.942831	0.963631
K11743	-0.00431	0.943681	0.964357
K03325	0.004263	0.944279	0.964682
K03128	0.00427	0.944214	0.964682
K01731	0.004236	0.944625	0.964893
K01705	-0.00423	0.944798	0.964926
K00800	-0.00421	0.945019	0.964979
K13655	0.004198	0.945129	0.964979
K05760	-0.00416	0.945601	0.965318
K01255	0.004127	0.946082	0.965524
K04103	-0.00413	0.94608	0.965524
K10561	0.004105	0.946333	0.965637
K13636	-0.00407	0.946837	0.966008
K10008	-0.00403	0.947289	0.966239
K08343	-0.00403	0.947342	0.966239
K06990	-0.00401	0.947521	0.966279
K03612	0.004001	0.947695	0.966313
K03712	-0.00389	0.949143	0.967646
K13774	0.003877	0.949343	0.967708
K02300	0.003862	0.949541	0.967767
K04655	0.003821	0.95004	0.967847
K06120	0.003833	0.949924	0.967847
K07241	-0.00383	0.950008	0.967847
K01607	0.003803	0.950277	0.967945
K14696	0.003747	0.951036	0.968575
K02755	-0.00371	0.951497	0.968902

K11208	0.003687	0.951827	0.968952
K13543	-0.00369	0.951721	0.968952
K02679	-0.00361	0.952853	0.969583
K09945	-0.00362	0.952673	0.969583
K03955	-0.00361	0.952868	0.969583
K02443	-0.00351	0.954072	0.970286
K12685	-0.00354	0.953726	0.970286
K13522	0.003511	0.954121	0.970286
K11362	0.003512	0.954112	0.970286
K02640	0.00346	0.95479	0.970823
K03767	0.003434	0.955104	0.970999
K07740	-0.0034	0.955567	0.971327
K05952	0.003374	0.955905	0.971429
K09997	0.003371	0.955949	0.971429
K07757	-0.00336	0.956095	0.971435
K02637	-0.0033	0.956928	0.972138
K14440	-0.00327	0.957246	0.972318
K01429	-0.00321	0.957963	0.972473
K03571	0.003223	0.957861	0.972473
K05580	0.003236	0.957708	0.972473
K10192	-0.00322	0.957837	0.972473
K07741	0.003149	0.958822	0.973059
K08369	-0.00316	0.958731	0.973059
K02061	-0.00312	0.959253	0.973284
K13975	0.003112	0.959326	0.973284
K09512	0.003043	0.960226	0.974054
K09944	-0.00303	0.960391	0.974079
K02024	-0.00301	0.96063	0.974178
K05783	0.002955	0.961384	0.974799
K11910	0.002939	0.961591	0.974866
K09894	-0.00292	0.961809	0.974884
K12683	-0.00292	0.961891	0.974884
K12241	0.002831	0.963	0.975864
K12227	0.002791	0.963519	0.976247
K07024	0.002679	0.964967	0.977571
K03360	0.00264	0.965493	0.97796
K12442	0.002627	0.965664	0.97799
K12454	0.002586	0.966196	0.978385
K15125	0.002566	0.966449	0.978498
K06169	0.002508	0.96722	0.979135
K13008	0.002475	0.967645	0.979421
K01682	0.002453	0.967932	0.97953
K06185	-0.00245	0.968036	0.97953
K03208	0.002433	0.968193	0.979545
K13273	0.002417	0.968404	0.979615
K13061	-0.00233	0.969495	0.980575
K02156	-0.00226	0.970494	0.981441
K03291	-0.00224	0.970658	0.981464
K06197	0.002177	0.971547	0.982219
K05356	0.002165	0.971702	0.982231
K03747	0.002141	0.972009	0.982254
K11941	-0.00215	0.971897	0.982254

K02852	-0.00209	0.972661	0.982769
K05648	0.00199	0.973988	0.983966
K03480	-0.0019	0.975176	0.98491
K04411	0.001886	0.975351	0.98491
K12531	0.001887	0.975331	0.98491
K01039	-0.00177	0.976879	0.986309
K00394	-0.00162	0.978811	0.987393
K00872	-0.00167	0.978188	0.987393
K01708	0.001638	0.978574	0.987393
K01850	0.001636	0.978608	0.987393
K09009	0.001645	0.978493	0.987393
K02213	-0.00162	0.9788	0.987393
K05895	-0.00156	0.979544	0.987844
K12953	-0.00157	0.979442	0.987844
K12252	0.001481	0.980643	0.988808
K07173	0.001452	0.981003	0.989026
K01224	-0.00138	0.981965	0.989418
K07017	-0.00139	0.98187	0.989418
K10985	0.001384	0.98191	0.989418
K05849	0.001385	0.9819	0.989418
K01563	0.001337	0.982509	0.989822
K13638	0.001322	0.982706	0.989876
K13990	0.001309	0.98288	0.989906
K01772	0.001233	0.983873	0.990513
K03526	0.00123	0.983913	0.990513
K11704	-0.00124	0.983806	0.990513
K03675	0.001172	0.984675	0.990991
K07261	-0.00118	0.98461	0.990991
K08349	-0.00112	0.985326	0.991228
K11982	-0.00112	0.98537	0.991228
K15175	0.001139	0.985103	0.991228
K08482	0.00111	0.985485	0.991228
K00325	-0.00101	0.986784	0.991299
K00822	-0.00103	0.986492	0.991299
K02120	0.00107	0.986011	0.991299
K03098	-0.00104	0.986389	0.991299
K09998	0.001051	0.98626	0.991299
K12660	-0.00101	0.986847	0.991299
K03894	-0.00102	0.986719	0.991299
K09519	0.001053	0.986228	0.991299
K09947	0.001026	0.986585	0.991299
K01583	-0.00098	0.987241	0.991414
K03699	0.000975	0.987249	0.991414
K08172	0.000828	0.989169	0.992552
K09771	-0.00088	0.988538	0.992552
K09778	-0.00083	0.989134	0.992552
K09900	0.000833	0.989106	0.992552
K11209	0.000847	0.98893	0.992552
K14623	-0.00082	0.989245	0.992552
K10007	-0.00079	0.989737	0.992757
K10120	-0.00079	0.989623	0.992757
K02575	-0.00071	0.990696	0.993575

K01934	-0.00067	0.991232	0.993968
K06137	-0.00065	0.991553	0.994145
K09151	-0.00059	0.992249	0.994518
K11605	0.000589	0.992304	0.994518
K15257	0.000585	0.992356	0.994518
K07737	-0.00054	0.992936	0.99481
K14549	-0.00055	0.992824	0.99481
K14830	-0.00048	0.993745	0.995476
K03493	-0.00044	0.994204	0.995791
K04784	-0.00041	0.99464	0.996083
K08312	0.000361	0.995278	0.996578
K02480	0.000187	0.997559	0.997966
K05574	-0.0002	0.99741	0.997966
K06139	-0.00022	0.997133	0.997966
K14063	-0.00017	0.997821	0.997966
K02554	0.000212	0.997231	0.997966
K12113	0.000169	0.997791	0.997966
K13799	0.000181	0.997628	0.997966
K05579	0.000181	0.997629	0.997966
K03777	-0.00012	0.998444	0.998444
