# Supplementary material for: Human and preclinical studies of the host–gut microbiome co-metabolite hippurate as a marker and mediator of metabolic health
Source: Gut. 2021 May 11;70(11):2105–14. doi: 10.1136/gutjnl-2020-323314 (PMC8515120; doi:10.1136/gutjnl-2020-323314)
Supplement: Supplementary data [file gutjnl-2020-323314supp013.pdf]

Supplementary Table ST.4. Association between gut microbiota functional potentials mapped to gut-specific metabolic modules (GMMs) describing microbial phenylpropanoid metabolism and urine hippurate levels.

GMM	Spearman ρ	P	FDR	Detected in N samples
MC0005	0.2113	0.0005	0.0060	201
MC0014	0.1985	0.0010	0.0060	34
MC0004	0.1916	0.0015	0.0060	271
MC0002	-0.1150	0.0587	0.1594	271
MC0018	0.1117	0.0664	0.1594	270
MC0017	-0.0862	0.1570	0.3140	145
MC0020	0.0511	0.4020	0.6891	271
MC0019	0.0385	0.5276	0.7914	270
MC0011	0.0278	0.6491	0.8655	25
MC0015	-0.0212	0.7280	0.8736	10
MC0003	0.0093	0.8792	0.8792	57
MC0010	0.0101	0.8684	0.8792	271
