# Supplementary material for: Human and preclinical studies of the host–gut microbiome co-metabolite hippurate as a marker and mediator of metabolic health
Source: Gut. 2021 May 11;70(11):2105–14. doi: 10.1136/gutjnl-2020-323314 (PMC8515120; doi:10.1136/gutjnl-2020-323314)
Supplement: Supplementary data [file gutjnl-2020-323314supp014.pdf]

Supplementary Table ST.5. Association between abundance of gut-specific metabolic modules (GMMs) describing microbial phenylpropanoid metabolism and microbial gene richness.

Variable	by Variable	Spearman ρ	P	FDR
Richness	MC0002	0.069605	2.53E-01	3.38E-01
Richness	MC0003	0.085162	1.62E-01	2.78E-01
Richness	MC0004	0.631656	1.39E-31	8.35E-31
Richness	MC0005	0.338217	1.12E-08	4.49E-08
Richness	MC0010	0.048013	4.31E-01	5.17E-01
Richness	MC0011	0.018112	7.67E-01	7.67E-01
Richness	MC0014	0.232558	1.12E-04	2.68E-04
Richness	MC0015	-0.0306	6.16E-01	6.72E-01
Richness	MC0017	0.072978	2.31E-01	3.38E-01
Richness	MC0018	0.134976	2.63E-02	5.26E-02
Richness	MC0019	0.325816	4.03E-08	1.21E-07
Richness	MC0020	0.640955	9.45E-33	1.13E-31
