# Supplementary material for: Human and preclinical studies of the host–gut microbiome co-metabolite hippurate as a marker and mediator of metabolic health
Source: Gut. 2021 May 11;70(11):2105–14. doi: 10.1136/gutjnl-2020-323314 (PMC8515120; doi:10.1136/gutjnl-2020-323314)
Supplement: Supplementary data [file gutjnl-2020-323314supp015.pdf]

Supplementary Table ST.6. Phenylpropanoid metabolism potential in metagenomic species, assessed by mapping to GMMs significantly associated to urine hippurate levels.

etagenomic species (M		MM detection lev		MGS taxonomy			
MGS	MGS size	MC0004	MC0005	species	genus	phylum	superkingdom
CAG00013	5283	1	0.333	unclassified Clostridiales	unclassified Clostridiales	Firmicutes	Bacteria
CAG00014	5115	1	0.333	Lachnospiraceae bacterium x4	unclassified Lachnospiraceae	Firmicutes	Bacteria
CAG00015	5054	1	0	Hungatella hathewayi 2	Hungatella	Firmicutes	Bacteria
CAG00610	1706	1	0	Hungatella hathewayi 2	Hungatella	Firmicutes	Bacteria
CAG00025	4407	1	0	Hungatella hathewayi 1	Hungatella	Firmicutes	Bacteria
CAG00029	4210	1	0.333	unclassified Lachnoclostridium	Lachnoclostridium	Firmicutes	Bacteria
CAG00760	4085	1	0	Ruminococcus sp. CAG:177	Ruminococcus	Firmicutes	Bacteria
CAG00164	3943	1	0	Blautia wexlerae	Blautia	Firmicutes	Bacteria
CAG00036	3712	1	0.333	Clostridium innocuum	Erysipelatoclostridium	Firmicutes	Bacteria
CAG00039	3693	1	0	unclassified Firmicutes	unclassified Firmicutes	Firmicutes	Bacteria
CAG00041	3638	1	0	unclassified Firmicutes	unclassified Firmicutes	Firmicutes	Bacteria
CAG00042	3599	1	0.333	unclassified Faecalibacterium	Faecalibacterium	Firmicutes	Bacteria
CAG00044	3582	1	0.333	Clostridium symbiosum	Lachnoclostridium	Firmicutes	Bacteria
CAG00047	3568	1	0	unclassified Lachnoclostridium	Lachnoclostridium	Firmicutes	Bacteria
CAG00051	3531	1	0.333	Clostridium clostridioforme	Lachnoclostridium	Firmicutes	Bacteria
CAG00055	3490	1	0.333	Roseburia sp. CAG:182	Roseburia	Firmicutes	Bacteria
CAG00061	3388	1	0.333	unclassified	unclassified	unclassified	unclassified
CAG00103	3249	1	0	unclassified Lachnospiraceae	unclassified Lachnospiraceae	Firmicutes	Bacteria
CAG00766	3209	1	0.333	Firmicutes bacterium CAG:176	unclassified Firmicutes	Firmicutes	Bacteria
CAG00848	1429	1	0.333	unclassified Firmicutes	unclassified Firmicutes	Firmicutes	Bacteria
CAG00070	3146	1	0	Blautia hydrogenotrophica	Blautia	Firmicutes	Bacteria
CAG00077	3096	1	0.333	Erysipelotrichaceae bacterium 3_1_53	unclassified Erysipelotrichacea	Firmicutes	Bacteria
CAG00079	3078	1	0.333	Clostridium sp. CAG:7	unclassified Clostridiales	Firmicutes	Bacteria
CAG00082	3037	1	0	Erysipelatoclostridium ramosum	Erysipelatoclostridium	Firmicutes	Bacteria
CAG00084	2999	1	0	unclassified	unclassified	unclassified	unclassified
CAG00099	2880	1	0.333	unclassified Firmicutes	unclassified Firmicutes	Firmicutes	Bacteria
CAG00101	2879	1	0	Ruminococcus sp. CAG:17	Blautia	Firmicutes	Bacteria
CAG00110	2805	1	0	uncultured Clostridium sp.	unclassified Clostridiales	Firmicutes	Bacteria
CAG00113	2790	1	0	Clostridium scindens	Lachnoclostridium	Firmicutes	Bacteria
CAG00125	2735	1	0	unclassified	unclassified	unclassified	unclassified
CAG00128	2722	1	0	unclassified Blautia	Blautia	Firmicutes	Bacteria
CAG00129	2713	1	0	unclassified Desulfovibrionales	unclassified Desulfovibrionales	Proteobacteria	Bacteria
CAG00243	2625	1	0	Ruminococcus torques 1	Blautia	Firmicutes	Bacteria
CAG00200	2615	1	0.333	unclassified Flavonifractor	Flavonifractor	Firmicutes	Bacteria
CAG00183	2480	1	0	Ruminococcus sp. CAG:60	Ruminococcus	Firmicutes	Bacteria
CAG00193	2443	1	0.333	unclassified Firmicutes	unclassified Firmicutes	Firmicutes	Bacteria
CAG00197	2433	1	0	Bifidobacterium gallinarum = saeculare =	Bifidobacterium	Actinobacteria	Bacteria
CAG00204	2415	1	0	unclassified Firmicutes	unclassified Firmicutes	Firmicutes	Bacteria
CAG00215	2381	1	0.333	unclassified Clostridiales	unclassified Clostridiales	Firmicutes	Bacteria
CAG00304	2284	1	0	unclassified Firmicutes	unclassified Firmicutes	Firmicutes	Bacteria
CAG00241	2282	1	0	Anaerotruncus sp. CAG:390	Anaerotruncus	Firmicutes	Bacteria
CAG00255	2253	1	0	unclassified Blautia	Blautia	Firmicutes	Bacteria
CAG00259	2248	1	0	unclassified Firmicutes	unclassified Firmicutes	Firmicutes	Bacteria
CAG00264	2230	1	0	unclassified Lachnospiraceae	unclassified Lachnospiraceae	Firmicutes	Bacteria
CAG00277	2204	1	0	unclassified Ruminococcaceae	unclassified Ruminococcaceae	Firmicutes	Bacteria
CAG00292	2177	1	0.333	unclassified	unclassified	unclassified	unclassified
CAG00294	2176	1	0.333	unclassified Firmicutes	unclassified Firmicutes	Firmicutes	Bacteria
CAG00305	2165	1	0.333	unclassified Clostridiales	unclassified Clostridiales	Firmicutes	Bacteria

CAG00523	2163	1	0	Eubacterium sp. CAG:180	Eubacterium	Firmicutes	Bacteria
CAG00308	2153	1	0	unclassified Firmicutes	unclassified Firmicutes	Firmicutes	Bacteria
CAG00318	2129	1	0.333	Clostridium sp. CAG:510	unclassified Clostridiales	Firmicutes	Bacteria
CAG00348	2086	1	0	unclassified	unclassified	unclassified	unclassified
CAG00350	2079	1	0	unclassified	unclassified	unclassified	unclassified
CAG00351	2078	1	0.333	unclassified Faecalicoccus	Faecalicoccus	Firmicutes	Bacteria
CAG00356	2066	1	0	unclassified	unclassified	unclassified	unclassified
CAG00373	2047	1	0	unclassified Clostridiales	unclassified Clostridiales	Firmicutes	Bacteria
CAG00032_1	2037	1	0.333	unclassified Firmicutes	unclassified Firmicutes	Firmicutes	Bacteria
CAG00381	2033	1	0	Clostridium sp. CAG:242	unclassified Clostridiales	Firmicutes	Bacteria
CAG00388	2024	1	0.333	unclassified	unclassified	unclassified	unclassified
CAG00391	2019	1	0.333	unclassified Firmicutes	unclassified Firmicutes	Firmicutes	Bacteria
CAG00492	2012	1	0	unclassified Ruminococcaceae	unclassified Ruminococcaceae	Firmicutes	Bacteria
CAG00394	2007	1	0	unclassified	unclassified	unclassified	unclassified
CAG00396	2006	1	0	unclassified Firmicutes	unclassified Firmicutes	Firmicutes	Bacteria
CAG00402	2001	1	0.333	unclassified Firmicutes	unclassified Firmicutes	Firmicutes	Bacteria
CAG00418	1981	1	0	unclassified Anaerotruncus	Anaerotruncus	Firmicutes	Bacteria
CAG00732	1980	1	0.333	unclassified Eubacterium	Eubacterium	Firmicutes	Bacteria
CAG00431	1969	1	0	unclassified	unclassified	unclassified	unclassified
CAG00443	1947	1	0	unclassified Eubacterium	Eubacterium	Firmicutes	Bacteria
CAG00447	1945	1	0	Clostridium sp. CAG:413	unclassified Clostridiales	Firmicutes	Bacteria
CAG00460	1938	1	0.333	unclassified Clostridiales	unclassified Clostridiales	Firmicutes	Bacteria
CAG00488	1899	1	0	Clostridium sp. CAG:1024	unclassified Clostridiales	Firmicutes	Bacteria
CAG00982	1895	1	0.333	unclassified Eggerthellaceae	unclassified Eggerthellaceae	Actinobacteria	Bacteria
CAG00491	1889	1	0	unclassified Clostridiales	unclassified Clostridiales	Firmicutes	Bacteria
CAG00494	1885	1	0	unclassified Eggerthellaceae	unclassified Eggerthellaceae	Actinobacteria	Bacteria
CAG00497	1881	1	0	unclassified Firmicutes	unclassified Firmicutes	Firmicutes	Bacteria
CAG00500	1874	1	0	unclassified Clostridiales	unclassified Clostridiales	Firmicutes	Bacteria
CAG00509	1861	1	0	unclassified Firmicutes	unclassified Firmicutes	Firmicutes	Bacteria
CAG00518	1839	1	0	unclassified	unclassified	unclassified	unclassified
CAG00522	1835	1	0	unclassified Blautia	Blautia	Firmicutes	Bacteria
CAG01700	1828	1	0	unclassified Clostridiales	unclassified Clostridiales	Firmicutes	Bacteria
CAG00532	1816	1	0.333	Clostridium sp. CAG:678	unclassified Clostridiales	Firmicutes	Bacteria
CAG00533	1815	1	0.333	unclassified Firmicutes	unclassified Firmicutes	Firmicutes	Bacteria
CAG00537	1807	1	0	unclassified Clostridiales	unclassified Clostridiales	Firmicutes	Bacteria
CAG00543	1799	1	0	unclassified Firmicutes	unclassified Firmicutes	Firmicutes	Bacteria
CAG00550	1787	1	0	unclassified Coprobacillus	Coprobacillus	Firmicutes	Bacteria
CAG00554	1783	1	0.333	Eubacterium ramulus	Eubacterium	Firmicutes	Bacteria
CAG00555	1782	1	0.333	unclassified Clostridiales	unclassified Clostridiales	Firmicutes	Bacteria
CAG00559	1776	1	0	unclassified Firmicutes	unclassified Firmicutes	Firmicutes	Bacteria
CAG00579	1740	1	0.333	unclassified Oscillibacter	Oscillibacter	Firmicutes	Bacteria
CAG00581	1739	1	0	Ruminococcus sp. CAG:563	Ruminococcus	Firmicutes	Bacteria
CAG00586	1731	1	0.333	unclassified Blautia	Blautia	Firmicutes	Bacteria
CAG00598	1719	1	0.333	unclassified Firmicutes	unclassified Firmicutes	Firmicutes	Bacteria
CAG00603	1715	1	0.333	Firmicutes bacterium CAG:114	unclassified Firmicutes	Firmicutes	Bacteria
CAG00607	1710	1	0	Eubacterium sp. CAG:251	Eubacterium	Firmicutes	Bacteria
CAG00622	1695	1	0.333	unclassified Firmicutes	unclassified Firmicutes	Firmicutes	Bacteria
CAG00624	1693	1	0	unclassified	unclassified	unclassified	unclassified
CAG00626	1687	1	0.333	unclassified Firmicutes	unclassified Firmicutes	Firmicutes	Bacteria
CAG00628	1686	1	0.333	unclassified Faecalibacterium	Faecalibacterium	Firmicutes	Bacteria
CAG00629	1684	1	0	Firmicutes bacterium CAG:124	unclassified Firmicutes	Firmicutes	Bacteria
CAG00637	1673	1	0	unclassified Firmicutes	unclassified Firmicutes	Firmicutes	Bacteria

CAG00643	1670	1	0	unclassified	unclassified	unclassified	unclassified
CAG00648	1667	1	0	unclassified Clostridiales	unclassified Clostridiales	Firmicutes	Bacteria
CAG00649	1667	1	0	unclassified Dorea	Dorea	Firmicutes	Bacteria
CAG00651	1667	1	0	Lachnospiraceae bacterium 1_4_56FAA	unclassified Lachnospiraceae	Firmicutes	Bacteria
CAG00656	1664	1	0	Ruminococcus sp. CAG:488	Ruminococcus	Firmicutes	Bacteria
CAG00659	1657	1	0.333	unclassified Clostridiales	unclassified Clostridiales	Firmicutes	Bacteria
CAG00669	1649	1	0.333	unclassified Firmicutes	unclassified Firmicutes	Firmicutes	Bacteria
CAG00675	1643	1	0	Clostridium sp. CAG:217	unclassified Clostridiales	Firmicutes	Bacteria
CAG00684	1634	1	0	unclassified Firmicutes	unclassified Firmicutes	Firmicutes	Bacteria
CAG00691	1628	1	0	unclassified	unclassified	unclassified	unclassified
CAG00695	1618	1	0.333	unclassified Firmicutes	unclassified Firmicutes	Firmicutes	Bacteria
CAG00698	1612	1	0	unclassified Ruminococcus	Ruminococcus	Firmicutes	Bacteria
CAG00710	1603	1	0	Enorma massiliensis	Enorma	Actinobacteria	Bacteria
CAG00712	1600	1	0	unclassified Proteobacteria	unclassified Proteobacteria	Proteobacteria	Bacteria
CAG00719	1591	1	0	unclassified Firmicutes	unclassified Firmicutes	Firmicutes	Bacteria
CAG00728	1580	1	0	Anaerotruncus sp. CAG:528	Anaerotruncus	Firmicutes	Bacteria
CAG00738	1567	1	0	Ruminococcus sp. CAG:724	Ruminococcus	Firmicutes	Bacteria
CAG00749	1554	1	0	unclassified Bacteria	unclassified Bacteria	unclassified Bac	Bacteria
CAG00998	1546	1	0.333	Cryptobacterium sp. CAG:338	Cryptobacterium	Actinobacteria	Bacteria
CAG00762	1531	1	0	unclassified Eubacterium	Eubacterium	Firmicutes	Bacteria
CAG00763	1531	1	0.333	unclassified Firmicutes	unclassified Firmicutes	Firmicutes	Bacteria
CAG00770	1522	1	0	unclassified Coriobacteriia	unclassified Coriobacteriia	Actinobacteria	Bacteria
CAG00777	1510	1	0	unclassified Coriobacteriales	unclassified Coriobacteriales	Actinobacteria	Bacteria
CAG00781	1500	1	0.333	Firmicutes bacterium CAG:555	unclassified Firmicutes	Firmicutes	Bacteria
CAG00784	1498	1	0	unclassified Ruminococcaceae	unclassified Ruminococcaceae	Firmicutes	Bacteria
CAG00797	1484	1	0	Firmicutes bacterium CAG:341	unclassified Firmicutes	Firmicutes	Bacteria
CAG00799	1482	1	0	Dorea longicatena 1	Dorea	Firmicutes	Bacteria
CAG00812	1468	1	0	Catenibacterium sp. CAG:290	Catenibacterium	Firmicutes	Bacteria
CAG00818	1462	1	0	unclassified Coprobacillus	Coprobacillus	Firmicutes	Bacteria
CAG00823	1459	1	0	unclassified	unclassified	unclassified	unclassified
CAG00840	1440	1	0	unclassified	unclassified	unclassified	unclassified
CAG00846	1430	1	0	unclassified Firmicutes	unclassified Firmicutes	Firmicutes	Bacteria
CAG00849	1428	1	0.333	unclassified Clostridiales	unclassified Clostridiales	Firmicutes	Bacteria
CAG00851	1422	1	0	unclassified Firmicutes	unclassified Firmicutes	Firmicutes	Bacteria
CAG00857	1400	1	0	Eubacterium sp. CAG:841	Eubacterium	Firmicutes	Bacteria
CAG00859	1398	1	0	unclassified Firmicutes	unclassified Firmicutes	Firmicutes	Bacteria
CAG00865	1384	1	0	unclassified Clostridiales	unclassified Clostridiales	Firmicutes	Bacteria
CAG00870	1362	1	0	unclassified Firmicutes	unclassified Firmicutes	Firmicutes	Bacteria
CAG00872	1359	1	0	Firmicutes bacterium CAG:240	unclassified Firmicutes	Firmicutes	Bacteria
CAG00875	1354	1	0.333	unclassified Clostridiales	unclassified Clostridiales	Firmicutes	Bacteria
CAG00876	1354	1	0	unclassified Clostridiales	unclassified Clostridiales	Firmicutes	Bacteria
CAG00877	1353	1	0	unclassified Anaerotruncus	Anaerotruncus	Firmicutes	Bacteria
CAG00005_2	1345	1	0	unclassified Olsenella	Olsenella	Actinobacteria	Bacteria
CAG00901	1313	1	0	unclassified Firmicutes	unclassified Firmicutes	Firmicutes	Bacteria
CAG00907	1299	1	0	unclassified Firmicutes	unclassified Firmicutes	Firmicutes	Bacteria
CAG00914	1285	1	0	unclassified Clostridiales	unclassified Clostridiales	Firmicutes	Bacteria
CAG00985	1270	1	0	unclassified Oscillibacter	Oscillibacter	Firmicutes	Bacteria
CAG00946	1234	1	0.333	unclassified Holdemanella	Holdemanella	Firmicutes	Bacteria
CAG00951	1224	1	0	unclassified Clostridiales	unclassified Clostridiales	Firmicutes	Bacteria
CAG00952	1221	1	0	unclassified Lachnospiraceae	unclassified Lachnospiraceae	Firmicutes	Bacteria
CAG00956	1218	1	0	Faecalibacterium 7	Faecalibacterium	Firmicutes	Bacteria
CAG00962	1212	1	0	Dorea longicatena 2	Dorea	Firmicutes	Bacteria

CAG00967	1209	1	0.333	unclassified Firmicutes	unclassified Firmicutes	Firmicutes	Bacteria
CAG00970	1207	1	0.333	unclassified Firmicutes	unclassified Firmicutes	Firmicutes	Bacteria
CAG00978	1195	1	0	unclassified	unclassified	unclassified	unclassified
CAG00984	1182	1	0.333	unclassified Oscillibacter	Oscillibacter	Firmicutes	Bacteria
CAG00988	1175	1	0	Clostridium sp. CAG:349	unclassified Clostridiales	Firmicutes	Bacteria
CAG00995	1170	1	0	Firmicutes bacterium CAG:475	unclassified Firmicutes	Firmicutes	Bacteria
CAG01002	1163	1	0	unclassified Coprobacillus	Coprobacillus	Firmicutes	Bacteria
CAG01011	1140	1	0	unclassified Dorea	Dorea	Firmicutes	Bacteria
CAG01019	1133	1	0	unclassified Eggerthellaceae	unclassified Eggerthellaceae	Actinobacteria	Bacteria
CAG01021	1130	1	0	unclassified Firmicutes	unclassified Firmicutes	Firmicutes	Bacteria
CAG01037	1104	1	0.333	unclassified Lachnospiraceae	unclassified Lachnospiraceae	Firmicutes	Bacteria
CAG01038	1101	1	0	Acidaminococcus sp. CAG:917	Acidaminococcus	Firmicutes	Bacteria
CAG01040	1098	1	0	Clostridium asparagiforme	Lachnoclostridium	Firmicutes	Bacteria
CAG01048	1087	1	0	Clostridium sp. CAG:793	unclassified Clostridiales	Firmicutes	Bacteria
CAG01052	1082	1	0	Firmicutes bacterium CAG:103	unclassified Firmicutes	Firmicutes	Bacteria
CAG01637	1078	1	0	Firmicutes bacterium CAG:65	unclassified Firmicutes	Firmicutes	Bacteria
CAG01077	1049	1	0	unclassified Clostridiales	unclassified Clostridiales	Firmicutes	Bacteria
CAG01086	1037	1	0	unclassified Firmicutes	unclassified Firmicutes	Firmicutes	Bacteria
CAG01089	1028	1	0	unclassified Firmicutes	unclassified Firmicutes	Firmicutes	Bacteria
CAG01122	989	1	0	unclassified Desulfovibrio	Desulfovibrio	Proteobacteria	Bacteria
CAG01140	964	1	0	unclassified	unclassified	unclassified	unclassified
CAG02723	940	1	0	unclassified Olsenella	Olsenella	Actinobacteria	Bacteria
CAG01161	934	1	0	unclassified	unclassified	unclassified	unclassified
CAG01215	832	1	0.333	unclassified Clostridiales	unclassified Clostridiales	Firmicutes	Bacteria
CAG00007_4	828	1	0	unclassified Firmicutes	unclassified Firmicutes	Firmicutes	Bacteria
CAG01223	819	1	0.333	unclassified Firmicutes	unclassified Firmicutes	Firmicutes	Bacteria
CAG01225	819	1	0	unclassified Firmicutes	unclassified Firmicutes	Firmicutes	Bacteria
CAG01227	815	1	0	unclassified	unclassified	unclassified	unclassified
CAG01230	814	1	0	Clostridiales bacterium 1_7_47FAA & V	unclassified Clostridiales	Firmicutes	Bacteria
CAG01232	811	1	0	unclassified	unclassified	unclassified	unclassified
CAG01239	796	1	0	unclassified Blautia	Blautia	Firmicutes	Bacteria
CAG01247	777	1	0	Clostridium sp. D5	Clostridium	Firmicutes	Bacteria
CAG01250	774	1	0	unclassified	unclassified	unclassified	unclassified
CAG01251	773	1	0	unclassified	unclassified	unclassified	unclassified
CAG01252	771	1	0	Catenibacterium mitsuokai	Catenibacterium	Firmicutes	Bacteria
CAG01254	767	1	0	unclassified Firmicutes	unclassified Firmicutes	Firmicutes	Bacteria
CAG01256	760	1	0	unclassified Firmicutes	unclassified Firmicutes	Firmicutes	Bacteria
CAG01277	715	1	0	unclassified Clostridiales	unclassified Clostridiales	Firmicutes	Bacteria
CAG01281	711	1	0	unclassified Clostridiales	unclassified Clostridiales	Firmicutes	Bacteria
CAG01305	673	1	0	unclassified Firmicutes	unclassified Firmicutes	Firmicutes	Bacteria
CAG01326	631	1	0	unclassified Clostridium	Clostridium	Firmicutes	Bacteria
CAG01344	607	1	0	unclassified Eggerthellaceae	unclassified Eggerthellaceae	Actinobacteria	Bacteria
CAG00006_4	597	1	0	unclassified Firmicutes	unclassified Firmicutes	Firmicutes	Bacteria
CAG01357	584	1	0	unclassified Firmicutes	unclassified Firmicutes	Firmicutes	Bacteria
CAG01372	563	1	0	unclassified Oscillibacter	Oscillibacter	Firmicutes	Bacteria
CAG01375	560	1	0	unclassified Firmicutes	unclassified Firmicutes	Firmicutes	Bacteria
CAG01379	554	1	0	unclassified Hungatella	Hungatella	Firmicutes	Bacteria
CAG00096_2	528	1	0.333	unclassified	unclassified	unclassified	unclassified
CAG01410	504	1	0	unclassified Firmicutes	unclassified Firmicutes	Firmicutes	Bacteria
CAG01442	441	1	0	unclassified Lachnoclostridium	Lachnoclostridium	Firmicutes	Bacteria
CAG01450	426	1	0	unclassified	unclassified	unclassified	unclassified
CAG01471	402	1	0	unclassified Eubacterium	Eubacterium	Firmicutes	Bacteria

CAG01473	401	1	0	unclassified	unclassified	unclassified	unclassified
CAG01477	398	1	0	Ruminococcus gnavus	Blautia	Firmicutes	Bacteria
CAG50005	367	1	0	unclassified Clostridiales	unclassified Clostridiales	Firmicutes	Bacteria
CAG01523	353	1	0	unclassified Firmicutes	unclassified Firmicutes	Firmicutes	Bacteria
CAG01537	343	1	0	unclassified Firmicutes	unclassified Firmicutes	Firmicutes	Bacteria
CAG01583	319	1	0	unclassified Firmicutes	unclassified Firmicutes	Firmicutes	Bacteria
CAG01592	311	1	0	Coprobacillus sp. CAG:235	Coprobacillus	Firmicutes	Bacteria
CAG01809	258	1	0	unclassified Lachnoclostridium	Lachnoclostridium	Firmicutes	Bacteria
CAG00096_4	245	1	0.333	unclassified	unclassified	unclassified	unclassified
CAG01738	229	1	0	unclassified Firmicutes	unclassified Firmicutes	Firmicutes	Bacteria
CAG01773	215	1	0	unclassified Firmicutes	unclassified Firmicutes	Firmicutes	Bacteria
CAG01804	205	1	0	unclassified Lachnoclostridium	Lachnoclostridium	Firmicutes	Bacteria
CAG01894	178	1	0	unclassified Firmicutes	unclassified Firmicutes	Firmicutes	Bacteria
CAG01967	164	1	0	Bilophila wadsworthia	Bilophila	Proteobacteria	Bacteria
CAG01992	156	1	0	unclassified	unclassified	unclassified	unclassified
CAG02014	153	1	0	unclassified Firmicutes	unclassified Firmicutes	Firmicutes	Bacteria
CAG02064	146	1	0	unclassified Firmicutes	unclassified Firmicutes	Firmicutes	Bacteria
CAG02119	134	1	0	unclassified Candidatus Stoquefichus	Candidatus Stoquefichus	Firmicutes	Bacteria
CAG02176	125	1	0.333	unclassified	unclassified	unclassified	unclassified
CAG02177	125	1	0	unclassified Firmicutes	unclassified Firmicutes	Firmicutes	Bacteria
CAG02188	123	1	0	unclassified Catenibacterium	Catenibacterium	Firmicutes	Bacteria
CAG02251	116	1	0	unclassified Firmicutes	unclassified Firmicutes	Firmicutes	Bacteria
CAG02376	101	1	0	unclassified Firmicutes	unclassified Firmicutes	Firmicutes	Bacteria
CAG02391	99	1	0.333	unclassified	unclassified	unclassified	unclassified
CAG02499	91	1	0	unclassified Holdemanella	Holdemanella	Firmicutes	Bacteria
CAG02608	82	1	0	unclassified Firmicutes	unclassified Firmicutes	Firmicutes	Bacteria
CAG02627	81	1	0	unclassified	unclassified	unclassified	unclassified
CAG02678	78	1	0	unclassified Ruminococcaceae	unclassified Ruminococcaceae	Firmicutes	Bacteria
CAG02736	75	1	0	unclassified	unclassified	unclassified	unclassified
CAG02834	70	1	0	unclassified	unclassified	unclassified	unclassified
CAG02850	69	1	0	uncultured Faecalibacterium sp.	Faecalibacterium	Firmicutes	Bacteria
CAG03042	61	1	0	Clostridium clostridioforme	Lachnoclostridium	Firmicutes	Bacteria
CAG03081	60	1	0	unclassified	unclassified	unclassified	unclassified
CAG03192	56	1	0	unclassified	unclassified	unclassified	unclassified
CAG03247	54	1	0	unclassified Coprobacillus	Coprobacillus	Firmicutes	Bacteria
CAG03390	50	1	0	unclassified	unclassified	unclassified	unclassified
CAG00136	2680	0	0.667	Enterobacter hormaechei	Enterobacter	Proteobacteria	Bacteria
CAG00362	2058	0	0.667	unclassified Sutterella	Sutterella	Proteobacteria	Bacteria
CAG00001	12638	0	0.333	unclassified	unclassified	unclassified	unclassified
CAG00002	11313	0	0.333	unclassified	unclassified	unclassified	unclassified
CAG00003	10882	0	0.333	unclassified	unclassified	unclassified	unclassified
CAG00008	6646	0	0.333	Clostridium bolteae	Lachnoclostridium	Firmicutes	Bacteria
CAG00012	5363	0	0.667	Klebsiella pneumoniae 1	Klebsiella	Proteobacteria	Bacteria
CAG00017	4893	0	0.333	unclassified Lachnospiraceae	unclassified Lachnospiraceae	Firmicutes	Bacteria
CAG00034	3967	0	0.333	Enterobacter cloacae 2	Enterobacter	Proteobacteria	Bacteria
CAG00043	3582	0	0.333	Enterobacter asburiae	Enterobacter	Proteobacteria	Bacteria
CAG00067	3184	0	0.333	unclassified Clostridiales	unclassified Clostridiales	Firmicutes	Bacteria
CAG00090	2957	0	0.333	Anaerostipes caccae	Anaerostipes	Firmicutes	Bacteria
CAG00092	2951	0	0.667	unclassified Comamonas	Comamonas	Proteobacteria	Bacteria
CAG00093	2937	0	0.667	Klebsiella pneumoniae 2	Klebsiella	Proteobacteria	Bacteria
CAG00094	2936	0	0.333	Clostridium sp. 7_2_43FAA	unclassified Clostridiales	Firmicutes	Bacteria
CAG00114	2790	0	0.333	unclassified Lachnospiraceae	unclassified Lachnospiraceae	Firmicutes	Bacteria

CAG00115	2789	0	0.333	unclassified Lachnospiraceae	unclassified Lachnospiraceae	Firmicutes	Bacteria
CAG00122	2755	0	0.333	unclassified Lachnospiraceae	unclassified Lachnospiraceae	Firmicutes	Bacteria
CAG00127	2731	0	0.333	unclassified Lachnospiraceae	unclassified Lachnospiraceae	Firmicutes	Bacteria
CAG00134	2690	0	0.333	unclassified Cloacibacillus	Cloacibacillus	Synergistetes	Bacteria
CAG00153	2606	0	0.333	Hafnia paralvei	Hafnia	Proteobacteria	Bacteria
CAG00160	2584	0	0.333	unclassified	unclassified	unclassified	unclassified
CAG00177	2497	0	0.333	unclassified Megasphaera	Megasphaera	Firmicutes	Bacteria
CAG00185	2472	0	0.333	Veillonella sp. CAG:933	Veillonella	Firmicutes	Bacteria
CAG00186	2463	0	0.333	Streptococcus salivarius	Streptococcus	Firmicutes	Bacteria
CAG01575	322	0	0.333	Streptococcus gordonii	Streptococcus	Firmicutes	Bacteria
CAG01617	289	0	0.333	Streptococcus salivarius	Streptococcus	Firmicutes	Bacteria
CAG00187	2462	0	0.333	Sutterella wadsworthensis 1	Sutterella	Proteobacteria	Bacteria
CAG00205	2412	0	0.333	unclassified Megasphaera	Megasphaera	Firmicutes	Bacteria
CAG00217	2374	0	0.333	Dialister sp. CAG:357	Dialister	Firmicutes	Bacteria
CAG00226	2330	0	0.333	unclassified	unclassified	unclassified	unclassified
CAG00228	2321	0	0.333	unclassified Firmicutes	unclassified Firmicutes	Firmicutes	Bacteria
CAG00238	2284	0	0.333	Clostridium saccharolyticum	Lachnoclostridium	Firmicutes	Bacteria
CAG00239	2282	0	0.333	Flavonifractor plautii	Flavonifractor	Firmicutes	Bacteria
CAG00252	2258	0	0.333	unclassified Sutterella	Sutterella	Proteobacteria	Bacteria
CAG00263	2234	0	0.667	Klebsiella oxytoca	Klebsiella	Proteobacteria	Bacteria
CAG00266	2229	0	0.333	Streptococcus lutetiensis	Streptococcus	Firmicutes	Bacteria
CAG00270	2225	0	0.333	Oscillibacter sp. KLE 1728 / KLE 1745 / VI	Oscillibacter	Firmicutes	Bacteria
CAG00283	2196	0	0.333	Megasphaera sp. BL7 & NM10	Megasphaera	Firmicutes	Bacteria
CAG00293	2177	0	0.333	Firmicutes bacterium CAG:145	unclassified Firmicutes	Firmicutes	Bacteria
CAG00299	2163	0	0.333	unclassified Proteobacteria	unclassified Proteobacteria	Proteobacteria	Bacteria
CAG00302	2161	0	0.333	unclassified	unclassified	unclassified	unclassified
CAG00310	2146	0	0.333	Sutterella parvirubra	Sutterella	Proteobacteria	Bacteria
CAG00315	2139	0	0.333	Citrobacter freundii	Citrobacter	Proteobacteria	Bacteria
CAG00320	2127	0	0.333	Phascolarctobacterium sp. CAG:207	Phascolarctobacterium	Firmicutes	Bacteria
CAG00325	2121	0	0.333	unclassified Sutterellaceae	unclassified Sutterellaceae	Proteobacteria	Bacteria
CAG00326	2119	0	0.333	unclassified Firmicutes	unclassified Firmicutes	Firmicutes	Bacteria
CAG00068_1	2117	0	0.333	unclassified	unclassified	unclassified	unclassified
CAG00329	2113	0	0.333	Roseburia hominis	Roseburia	Firmicutes	Bacteria
CAG00331	2111	0	0.333	unclassified Eggerthella	Eggerthella	Actinobacteria	Bacteria
CAG00335	2102	0	0.333	Anaerostipes hadrus	Anaerostipes	Firmicutes	Bacteria
CAG00372	2047	0	0.333	unclassified Clostridiales	unclassified Clostridiales	Firmicutes	Bacteria
CAG00405	2000	0	0.333	unclassified Firmicutes	unclassified Firmicutes	Firmicutes	Bacteria
CAG00417	1981	0	0.333	Megamonas funiformis	Megamonas	Firmicutes	Bacteria
CAG00423	1975	0	0.333	unclassified Dialister	Dialister	Firmicutes	Bacteria
CAG00434	1965	0	0.333	Ruminococcus champanellensis	Ruminococcus	Firmicutes	Bacteria
CAG00437	1962	0	0.333	unclassified Burkholderiales	unclassified Burkholderiales	Proteobacteria	Bacteria
CAG00439	1952	0	0.333	unclassified Flavonifractor	Flavonifractor	Firmicutes	Bacteria
CAG00457	1940	0	0.333	unclassified Fusobacterium	Fusobacterium	Fusobacteria	Bacteria
CAG00459	1939	0	0.333	unclassified Proteobacteria	unclassified Proteobacteria	Proteobacteria	Bacteria
CAG00461	1936	0	0.333	Azospirillum sp. CAG:260	Azospirillum	Proteobacteria	Bacteria
CAG00462	1935	0	0.333	Streptococcus pasteurianus	Streptococcus	Firmicutes	Bacteria
CAG00472	1921	0	0.333	unclassified	unclassified	unclassified	unclassified
CAG00474	1919	0	0.333	Sutterella wadsworthensis 2	Sutterella	Proteobacteria	Bacteria
CAG00484	1903	0	0.333	Sutterella sp. CAG:397	Sutterella	Proteobacteria	Bacteria
CAG00489	1894	0	0.333	unclassified	unclassified	unclassified	unclassified
CAG00496	1884	0	0.333	Fusobacterium mortiferum	Fusobacterium	Fusobacteria	Bacteria
CAG00514	1851	0	0.333	unclassified Rhodospirillales	unclassified Rhodospirillales	Proteobacteria	Bacteria

CAG00519	1837	0	0.333	unclassified Lachnoclostridium	Lachnoclostridium	Firmicutes	Bacteria
CAG00540	1802	0	0.333	unclassified Sutterella	Sutterella	Proteobacteria	Bacteria
CAG00561	1774	0	0.333	unclassified Betaproteobacteria	unclassified Betaproteobacteri	Proteobacteria	Bacteria
CAG00576	1745	0	0.333	unclassified Firmicutes	unclassified Firmicutes	Firmicutes	Bacteria
CAG00593	1728	0	0.333	unclassified Phascolarctobacterium	Phascolarctobacterium	Firmicutes	Bacteria
CAG00594	1726	0	0.333	unclassified Eggerthella	Eggerthella	Actinobacteria	Bacteria
CAG00617	1699	0	0.333	Acetobacter sp. CAG:977	Acetobacter	Proteobacteria	Bacteria
CAG00638	1673	0	0.333	unclassified Lachnoclostridium	Lachnoclostridium	Firmicutes	Bacteria
CAG00647	1668	0	0.333	unclassified Sutterella	Sutterella	Proteobacteria	Bacteria
CAG00652	1667	0	0.333	unclassified Sutterella	Sutterella	Proteobacteria	Bacteria
CAG00654	1666	0	0.333	Ruminococcus sp. CAG:624	Ruminococcus	Firmicutes	Bacteria
CAG00657	1663	0	0.333	unclassified Sutterella	Sutterella	Proteobacteria	Bacteria
CAG00663	1656	0	0.333	unclassified Burkholderiales	unclassified Burkholderiales	Proteobacteria	Bacteria
CAG00668	1649	0	0.333	unclassified Rhodospirillales	unclassified Rhodospirillales	Proteobacteria	Bacteria
CAG00699	1612	0	0.333	Veillonella ratti	Veillonella	Firmicutes	Bacteria
CAG00727	1580	0	0.333	Eggerthella lenta	Eggerthella	Actinobacteria	Bacteria
CAG00730	1576	0	0.333	unclassified Dialister	Dialister	Firmicutes	Bacteria
CAG00731	1576	0	0.333	Parasutterella excrementihominis 2	Parasutterella	Proteobacteria	Bacteria
CAG00745	1558	0	0.333	Dialister invisus	Dialister	Firmicutes	Bacteria
CAG00746	1557	0	0.333	unclassified	unclassified	unclassified	unclassified
CAG00753	1549	0	0.333	Azospirillum sp. CAG:239	Azospirillum	Proteobacteria	Bacteria
CAG00779	1507	0	0.333	unclassified Megasphaera	Megasphaera	Firmicutes	Bacteria
CAG00787	1495	0	0.333	Dialister sp. CAG:486	Dialister	Firmicutes	Bacteria
CAG00788	1495	0	0.333	Eggerthella sp. CAG:1427	Eggerthella	Actinobacteria	Bacteria
CAG00801	1481	0	0.333	unclassified Burkholderiales	unclassified Burkholderiales	Proteobacteria	Bacteria
CAG00834	1446	0	0.333	Oxalobacter formigenes	Oxalobacter	Proteobacteria	Bacteria
CAG00836	1443	0	0.333	Fusobacterium gonidiaformans	Fusobacterium	Fusobacteria	Bacteria
CAG00838	1443	0	0.333	unclassified	unclassified	unclassified	unclassified
CAG00847	1429	0	0.333	unclassified Sutterella	Sutterella	Proteobacteria	Bacteria
CAG00858	1400	0	0.333	Streptococcus thermophilus	Streptococcus	Firmicutes	Bacteria
CAG00879	1352	0	0.333	unclassified Ruminococcaceae	unclassified Ruminococcaceae	Firmicutes	Bacteria
CAG00881	1351	0	0.333	unclassified Dialister	Dialister	Firmicutes	Bacteria
CAG00885	1336	0	0.333	Phascolarctobacterium sp. CAG:266	Phascolarctobacterium	Firmicutes	Bacteria
CAG00886	1334	0	0.333	Sutterella sp. CAG:351	Sutterella	Proteobacteria	Bacteria
CAG00889	1328	0	0.333	Megasphaera elsdenii	Megasphaera	Firmicutes	Bacteria
CAG00898	1318	0	0.333	unclassified Eggerthella	Eggerthella	Actinobacteria	Bacteria
CAG00902	1309	0	0.333	Firmicutes bacterium CAG:822	unclassified Firmicutes	Firmicutes	Bacteria
CAG00005_4	503	0	0.333	unclassified Clostridiales	unclassified Clostridiales	Firmicutes	Bacteria
CAG00909	1297	0	0.333	Parasutterella excrementihominis 1	Parasutterella	Proteobacteria	Bacteria
CAG00929	1264	0	0.333	Eggerthella sp. CAG:368	Eggerthella	Actinobacteria	Bacteria
CAG00931	1257	0	0.333	unclassified Oscillibacter	Oscillibacter	Firmicutes	Bacteria
CAG00936	1245	0	0.333	Eggerthella sp. CAG:209	Eggerthella	Actinobacteria	Bacteria
CAG00975	1201	0	0.333	Veillonella atypica	Veillonella	Firmicutes	Bacteria
CAG01026	1179	0	0.333	Peptoniphilus harei	Peptoniphilus	Firmicutes	Bacteria
CAG00993	1171	0	0.333	unclassified Phascolarctobacterium	Phascolarctobacterium	Firmicutes	Bacteria
CAG01007	1159	0	0.333	unclassified Burkholderiales	unclassified Burkholderiales	Proteobacteria	Bacteria
CAG01046	1089	0	0.333	unclassified Clostridiales	unclassified Clostridiales	Firmicutes	Bacteria
CAG01053	1081	0	0.333	unclassified	unclassified	unclassified	unclassified
CAG01060	1074	0	0.333	unclassified Burkholderiales	unclassified Burkholderiales	Proteobacteria	Bacteria
CAG00007_3	1061	0	0.333	unclassified Clostridiales	unclassified Clostridiales	Firmicutes	Bacteria
CAG01115	999	0	0.333	Eggerthella sp. CAG:298	Eggerthella	Actinobacteria	Bacteria
CAG01128	980	0	0.333	unclassified Dialister	Dialister	Firmicutes	Bacteria

CAG01131	977	0	0.333	Sutterella sp. CAG:521	Sutterella	Proteobacteria	Bacteria
CAG01133	975	0	0.333	Senegalimassilia anaerobia	Senegalimassilia	Actinobacteria	Bacteria
CAG01148	954	0	0.333	Clostridium scindens	Lachnoclostridium	Firmicutes	Bacteria
CAG01203	860	0	0.333	Streptococcus mutans	Streptococcus	Firmicutes	Bacteria
CAG01205	855	0	0.333	Phascolarctobacterium succinatutens	Phascolarctobacterium	Firmicutes	Bacteria
CAG01259	755	0	0.333	Streptococcus oralis	Streptococcus	Firmicutes	Bacteria
CAG01272	726	0	0.333	Adlercreutzia equolifaciens	Adlercreutzia	Actinobacteria	Bacteria
CAG01291	686	0	0.333	unclassified Clostridiales	unclassified Clostridiales	Firmicutes	Bacteria
CAG01295	682	0	0.333	unclassified Burkholderiales	unclassified Burkholderiales	Proteobacteria	Bacteria
CAG01298	678	0	0.333	Oxalobacter formigenes	Oxalobacter	Proteobacteria	Bacteria
CAG01306	670	0	0.333	unclassified Oscillibacter	Oscillibacter	Firmicutes	Bacteria
CAG01339	614	0	0.333	Peptoniphilus lacrimalis	Peptoniphilus	Firmicutes	Bacteria
CAG01348	601	0	0.333	unclassified Lachnoclostridium	Lachnoclostridium	Firmicutes	Bacteria
CAG01366	571	0	0.333	Streptococcus parasanguinis	Streptococcus	Firmicutes	Bacteria
CAG01399	524	0	0.333	unclassified Oscillibacter	Oscillibacter	Firmicutes	Bacteria
CAG01551	508	0	0.333	unclassified Oscillibacter	Oscillibacter	Firmicutes	Bacteria
CAG01644	278	0	0.333	unclassified Firmicutes	unclassified Firmicutes	Firmicutes	Bacteria
CAG01780	212	0	0.333	butyrate-producing bacterium SS3/4 - Cl	unclassified Clostridiales	Firmicutes	Bacteria
CAG01846	190	0	0.333	unclassified Ruminococcus	Ruminococcus	Firmicutes	Bacteria
CAG01871	184	0	0.333	Eggerthella sp. CAG:298	Eggerthella	Actinobacteria	Bacteria
CAG01881	181	0	0.333	Anaeroglobus geminatus	Anaeroglobus	Firmicutes	Bacteria
CAG02026	152	0	0.333	unclassified Clostridiales	unclassified Clostridiales	Firmicutes	Bacteria
CAG02067	146	0	0.333	Dialister succinatiphilus	Dialister	Firmicutes	Bacteria
CAG02116	135	0	0.333	unclassified Sutterella	Sutterella	Proteobacteria	Bacteria
CAG02385	100	0	0.333	unclassified	unclassified	unclassified	unclassified
CAG02988	63	0	0.333	unclassified Clostridiales	unclassified Clostridiales	Firmicutes	Bacteria
CAG00009	6333	0	0.333	unclassified Lachnospiraceae	unclassified Lachnospiraceae	Firmicutes	Bacteria
CAG00020	4814	0	0.333	Clostridium sp. KLE 1755 & Clostridiales l	unclassified Clostridiales	Firmicutes	Bacteria
CAG00021	4703	0	0.333	Clostridium butyricum	Clostridium	Firmicutes	Bacteria
CAG01028	4286	0	0.333	Ruminococcaceae bacterium LM158	unclassified Ruminococcaceae	Firmicutes	Bacteria
CAG00035	3871	0	0.333	unclassified Clostridiales	unclassified Clostridiales	Firmicutes	Bacteria
CAG00490	3314	0	0.333	Coprococcus sp. CAG:131 & Coprococcus s	Coprococcus	Firmicutes	Bacteria
CAG00601	3250	0	0.333	unclassified Faecalibacterium	Faecalibacterium	Firmicutes	Bacteria
CAG00146	3173	0	0.333	unclassified Butyricimonas	Butyricimonas	Bacteroidetes	Bacteria
CAG01051	3142	0	0.333	unclassified Oscillibacter	Oscillibacter	Firmicutes	Bacteria
CAG00072	3133	0	0.333	Odoribacter laneus	Odoribacter	Bacteroidetes	Bacteria
CAG00083	3030	0	0.333	Odoribacter sp. CAG:788	Odoribacter	Bacteroidetes	Bacteria
CAG00100	2893	0	0.333	Clostridium sp. JCC	unclassified Clostridiales	Firmicutes	Bacteria
CAG00102	2875	0	0.333	unclassified Lachnospiraceae	unclassified Lachnospiraceae	Firmicutes	Bacteria
CAG00580	2835	0	0.333	Clostridium sp. CAG:62	unclassified Clostridiales	Firmicutes	Bacteria
CAG00108	2820	0	0.333	Clostridium paraputrificum	Clostridium	Firmicutes	Bacteria
CAG00109	2808	0	0.333	unclassified Fusobacterium	Fusobacterium	Fusobacteria	Bacteria
CAG00119	2762	0	0.333	unclassified Clostridium	Clostridium	Firmicutes	Bacteria
CAG00123	2745	0	0.333	unclassified Clostridiales	unclassified Clostridiales	Firmicutes	Bacteria
CAG00267	2738	0	0.333	Clostridium sp. CAG:58	unclassified Clostridiales	Firmicutes	Bacteria
CAG00137	2673	0	0.333	unclassified Firmicutes	unclassified Firmicutes	Firmicutes	Bacteria
CAG00144	2645	0	0.333	Fusobacterium ulcerans	Fusobacterium	Fusobacteria	Bacteria
CAG00145	2638	0	0.333	unclassified Clostridiales	unclassified Clostridiales	Firmicutes	Bacteria
CAG00260	2624	0	0.333	Acidaminococcus intestini	Acidaminococcus	Firmicutes	Bacteria
CAG00156	2594	0	0.333	Brachyspira aalborgi	Brachyspira	Spirochaetes	Bacteria
CAG00854	2572	0	0.333	unclassified Firmicutes	unclassified Firmicutes	Firmicutes	Bacteria
CAG00563	2567	0	0.333	Clostridium sp. CAG:91	unclassified Clostridiales	Firmicutes	Bacteria

CAG00166	2542	0	0.333	unclassified Clostridiales	unclassified Clostridiales	Firmicutes	Bacteria
CAG00005_1	2507	0	0.333	unclassified Firmicutes	unclassified Firmicutes	Firmicutes	Bacteria
CAG00894	1321	0	0.333	unclassified Firmicutes	unclassified Firmicutes	Firmicutes	Bacteria
CAG00189	2459	0	0.333	Roseburia sp. CAG:100	Roseburia	Firmicutes	Bacteria
CAG00191	2450	0	0.333	Fusobacterium varium	Fusobacterium	Fusobacteria	Bacteria
CAG00192	2447	0	0.333	unclassified Firmicutes	unclassified Firmicutes	Firmicutes	Bacteria
CAG00194	2436	0	0.333	Eubacterium hallii	Eubacterium	Firmicutes	Bacteria
CAG00196	2434	0	0.333	Firmicutes bacterium CAG:534	unclassified Firmicutes	Firmicutes	Bacteria
CAG00201	2419	0	0.333	unclassified Roseburia	Roseburia	Firmicutes	Bacteria
CAG00203	2416	0	0.333	unclassified Oscillibacter	Oscillibacter	Firmicutes	Bacteria
CAG00207	2398	0	0.333	Clostridium sp. CAG:411	unclassified Clostridiales	Firmicutes	Bacteria
CAG00208	2397	0	0.333	Faecalibacterium 8 (Ruminococcus sp. CA	Faecalibacterium	Firmicutes	Bacteria
CAG00212	2388	0	0.333	Bilophila wadsworthia	Bilophila	Proteobacteria	Bacteria
CAG00216	2379	0	0.333	unclassified Roseburia	Roseburia	Firmicutes	Bacteria
CAG00221	2362	0	0.333	unclassified Clostridiales	unclassified Clostridiales	Firmicutes	Bacteria
CAG00006_1	2362	0	0.333	unclassified Oscillibacter	Oscillibacter	Firmicutes	Bacteria
CAG00023_1	2356	0	0.333	Clostridium celatum	Clostridium	Firmicutes	Bacteria
CAG00261	2342	0	0.333	unclassified Oscillibacter	Oscillibacter	Firmicutes	Bacteria
CAG00231	2306	0	0.333	Odoribacter splanchnicus	Odoribacter	Bacteroidetes	Bacteria
CAG00232	2301	0	0.333	unclassified Ruminococcaceae	unclassified Ruminococcaceae	Firmicutes	Bacteria
CAG00233	2301	0	0.333	unclassified Clostridiales	unclassified Clostridiales	Firmicutes	Bacteria
CAG00237	2287	0	0.333	unclassified Butyricimonas	Butyricimonas	Bacteroidetes	Bacteria
CAG00240	2282	0	0.333	Oscillibacter sp. CAG:155	Oscillibacter	Firmicutes	Bacteria
CAG00242	2278	0	0.333	Roseburia sp. CAG:197	Roseburia	Firmicutes	Bacteria
CAG00248	2268	0	0.333	Roseburia sp. CAG:45	Roseburia	Firmicutes	Bacteria
CAG00256	2252	0	0.333	unclassified Lachnospiraceae	unclassified Lachnospiraceae	Firmicutes	Bacteria
CAG00265	2230	0	0.333	Firmicutes bacterium CAG:194	unclassified Firmicutes	Firmicutes	Bacteria
CAG00268	2227	0	0.333	Firmicutes bacterium CAG:24 & uncultur	unclassified Firmicutes	Firmicutes	Bacteria
CAG00274	2209	0	0.333	Erysipelotrichaceae bacterium 5_2_54FA	unclassified Erysipelotrichacea	Firmicutes	Bacteria
CAG00275	2207	0	0.333	Roseburia sp. CAG:18	Roseburia	Firmicutes	Bacteria
CAG00280	2199	0	0.333	Prevotella sp. CAG:485	Prevotella	Bacteroidetes	Bacteria
CAG00282	2196	0	0.333	Clostridium sp. CAG:277	unclassified Clostridiales	Firmicutes	Bacteria
CAG00826	2193	0	0.333	unclassified Clostridiales	unclassified Clostridiales	Firmicutes	Bacteria
CAG00288	2189	0	0.333	Firmicutes bacterium CAG:95	unclassified Firmicutes	Firmicutes	Bacteria
CAG00297	2174	0	0.333	unclassified Faecalibacterium	Faecalibacterium	Firmicutes	Bacteria
CAG00303	2160	0	0.333	unclassified Clostridiales	unclassified Clostridiales	Firmicutes	Bacteria
CAG00306	2154	0	0.333	Clostridium sp. CAG:221 - Clostridium sp.	unclassified Clostridiales	Firmicutes	Bacteria
CAG00307	2153	0	0.333	Clostridium sp. CAG:590	unclassified Clostridiales	Firmicutes	Bacteria
CAG00312	2140	0	0.333	unclassified Bacteroidetes	unclassified Bacteroidetes	Bacteroidetes	Bacteria
CAG00317	2130	0	0.333	Clostridium sp. CAG:230	unclassified Clostridiales	Firmicutes	Bacteria
CAG00319	2128	0	0.333	unclassified Butyricimonas	Butyricimonas	Bacteroidetes	Bacteria
CAG00321	2126	0	0.333	Clostridium sp. CAG:138	unclassified Clostridiales	Firmicutes	Bacteria
CAG00322	2126	0	0.333	unclassified Lachnospiraceae	unclassified Lachnospiraceae	Firmicutes	Bacteria
CAG00337	2102	0	0.333	unclassified Faecalibacterium	Faecalibacterium	Firmicutes	Bacteria
CAG00338	2100	0	0.333	unclassified Megasphaera	Megasphaera	Firmicutes	Bacteria
CAG00346	2092	0	0.333	unclassified Eubacterium	Eubacterium	Firmicutes	Bacteria
CAG00352	2078	0	0.333	unclassified Acidaminococcus	Acidaminococcus	Firmicutes	Bacteria
CAG00358	2063	0	0.333	Clostridium sp. CAG:632	unclassified Clostridiales	Firmicutes	Bacteria
CAG00361	2061	0	0.333	unclassified Acidaminococcus	Acidaminococcus	Firmicutes	Bacteria
CAG00363	2056	0	0.333	unclassified Clostridiales	unclassified Clostridiales	Firmicutes	Bacteria
CAG00366	2055	0	0.333	Coprococcus comes	Coprococcus	Firmicutes	Bacteria
CAG00382	2033	0	0.333	unclassified Clostridiales	unclassified Clostridiales	Firmicutes	Bacteria

CAG00385	2032	0	0.333	Eubacterium desmolans	Eubacterium	Firmicutes	Bacteria
CAG00568	2026	0	0.333	unclassified Faecalibacterium	Faecalibacterium	Firmicutes	Bacteria
CAG00387	2025	0	0.333	unclassified Lachnospiraceae	unclassified Lachnospiraceae	Firmicutes	Bacteria
CAG00390	2020	0	0.333	Butyrivibrio sp. CAG:318	Butyrivibrio	Firmicutes	Bacteria
CAG00398	2005	0	0.333	unclassified Lachnospiraceae	unclassified Lachnospiraceae	Firmicutes	Bacteria
CAG00407	1998	0	0.333	unclassified Lachnospiraceae	unclassified Lachnospiraceae	Firmicutes	Bacteria
CAG00408	1997	0	0.333	Clostridium sp. CAG:75	unclassified Clostridiales	Firmicutes	Bacteria
CAG00410	1993	0	0.333	unclassified Erysipelotrichaceae	unclassified Erysipelotrichacea	Firmicutes	Bacteria
CAG01600	304	0	0.333	unclassified Eubacterium	Eubacterium	Firmicutes	Bacteria
CAG00422	1976	0	0.333	unclassified Oscillibacter	Oscillibacter	Firmicutes	Bacteria
CAG00428	1970	0	0.333	Eubacterium sp. CAG:603	Eubacterium	Firmicutes	Bacteria
CAG00435	1965	0	0.333	unclassified Oscillibacter	Oscillibacter	Firmicutes	Bacteria
CAG00436	1963	0	0.333	Clostridium sp. CAG:299	unclassified Clostridiales	Firmicutes	Bacteria
CAG00446	1946	0	0.333	unclassified Clostridiales	unclassified Clostridiales	Firmicutes	Bacteria
CAG00448	1945	0	0.333	Clostridium sp. CAG:122	unclassified Clostridiales	Firmicutes	Bacteria
CAG00449	1945	0	0.333	Clostridium sp. CAG:253	unclassified Clostridiales	Firmicutes	Bacteria
CAG00450	1944	0	0.333	unclassified Firmicutes	unclassified Firmicutes	Firmicutes	Bacteria
CAG00452	1942	0	0.333	Clostridium sp. CAG:167	unclassified Clostridiales	Firmicutes	Bacteria
CAG00455	1941	0	0.333	unclassified Firmicutes	unclassified Firmicutes	Firmicutes	Bacteria
CAG00458	1939	0	0.333	Fusobacterium nucleatum	Fusobacterium	Fusobacteria	Bacteria
CAG00463	1934	0	0.333	Faecalibacterium 6	Faecalibacterium	Firmicutes	Bacteria
CAG00469	1928	0	0.333	Eubacterium sp. CAG:146	Eubacterium	Firmicutes	Bacteria
CAG00485	1901	0	0.333	unclassified Roseburia	Roseburia	Firmicutes	Bacteria
CAG00493	1887	0	0.333	Clostridium sp. CAG:127	unclassified Clostridiales	Firmicutes	Bacteria
CAG00502	1871	0	0.333	Eubacterium sp. CAG:161	Eubacterium	Firmicutes	Bacteria
CAG00505	1866	0	0.333	unclassified Firmicutes	unclassified Firmicutes	Firmicutes	Bacteria
CAG00506	1864	0	0.333	unclassified Ruminococcaceae	unclassified Ruminococcaceae	Firmicutes	Bacteria
CAG00511	1857	0	0.333	Firmicutes bacterium CAG:791	unclassified Firmicutes	Firmicutes	Bacteria
CAG00516	1848	0	0.333	unclassified Lachnospiraceae	unclassified Lachnospiraceae	Firmicutes	Bacteria
CAG00531	1819	0	0.333	unclassified Faecalibacterium	Faecalibacterium	Firmicutes	Bacteria
CAG00539	1802	0	0.333	unclassified Clostridiales	unclassified Clostridiales	Firmicutes	Bacteria
CAG00542	1799	0	0.333	Butyrivibrio crossotus	Butyrivibrio	Firmicutes	Bacteria
CAG00547	1792	0	0.333	unclassified Faecalibacterium	Faecalibacterium	Firmicutes	Bacteria
CAG00548	1789	0	0.333	Coprococcus sp. CAG:782	Coprococcus	Firmicutes	Bacteria
CAG00562	1778	0	0.333	Eubacterium dolichum	unclassified Erysipelotrichacea	Firmicutes	Bacteria
CAG50002	1766	0	0.333	unclassified Clostridiales	unclassified Clostridiales	Firmicutes	Bacteria
CAG00567	1760	0	0.333	Clostridium perfringens	Clostridium	Firmicutes	Bacteria
CAG00577	1743	0	0.333	Faecalibacterium 3	Faecalibacterium	Firmicutes	Bacteria
CAG00582	1738	0	0.333	Roseburia inulinivorans	Roseburia	Firmicutes	Bacteria
CAG00612	1705	0	0.333	Faecalibacterium 2	Faecalibacterium	Firmicutes	Bacteria
CAG00616	1701	0	0.333	unclassified Ruminococcaceae	unclassified Ruminococcaceae	Firmicutes	Bacteria
CAG00619	1697	0	0.333	unclassified Faecalibacterium	Faecalibacterium	Firmicutes	Bacteria
CAG00783	1689	0	0.333	unclassified Oscillibacter	Oscillibacter	Firmicutes	Bacteria
CAG00625	1687	0	0.333	unclassified Firmicutes	unclassified Firmicutes	Firmicutes	Bacteria
CAG00633	1680	0	0.333	unclassified Lachnospiraceae	unclassified Lachnospiraceae	Firmicutes	Bacteria
CAG00634	1676	0	0.333	unclassified Prevotella	Prevotella	Bacteroidetes	Bacteria
CAG00636	1675	0	0.333	unclassified Roseburia	Roseburia	Firmicutes	Bacteria
CAG00639	1671	0	0.333	Eubacterium sp. CAG:156	Eubacterium	Firmicutes	Bacteria
CAG00644	1670	0	0.333	Clostridium sp. CAG:226	unclassified Clostridiales	Firmicutes	Bacteria
CAG00666	1653	0	0.333	Clostridium sp. CAG:43	unclassified Clostridiales	Firmicutes	Bacteria
CAG00667	1653	0	0.333	Faecalibacterium 4	Faecalibacterium	Firmicutes	Bacteria
CAG00676	1643	0	0.333	unclassified Oscillibacter	Oscillibacter	Firmicutes	Bacteria

CAG00681	1635	0	0.333	Faecalitalea cylindroides	Faecalitalea	Firmicutes	Bacteria
CAG00692	1621	0	0.333	unclassified	unclassified	unclassified	unclassified
CAG00709	1606	0	0.333	unclassified Firmicutes	unclassified Firmicutes	Firmicutes	Bacteria
CAG00716	1592	0	0.333	unclassified Firmicutes	unclassified Firmicutes	Firmicutes	Bacteria
CAG00718	1591	0	0.333	Eubacterium ventriosum	Eubacterium	Firmicutes	Bacteria
CAG00720	1590	0	0.333	Anaerotruncus colihominis	Anaerotruncus	Firmicutes	Bacteria
CAG00722	1587	0	0.333	Acidaminococcus fermentans	Acidaminococcus	Firmicutes	Bacteria
CAG00729	1584	0	0.333	unclassified Oscillibacter	Oscillibacter	Firmicutes	Bacteria
CAG00735	1570	0	0.333	unclassified Eubacterium	Eubacterium	Firmicutes	Bacteria
CAG00742	1564	0	0.333	unclassified Fusobacterium	Fusobacterium	Fusobacteria	Bacteria
CAG00751	1552	0	0.333	Firmicutes bacterium CAG:238	unclassified Firmicutes	Firmicutes	Bacteria
CAG00961	1550	0	0.333	unclassified Ruminococcaceae	unclassified Ruminococcaceae	Firmicutes	Bacteria
CAG00755	1548	0	0.333	unclassified Faecalibacterium	Faecalibacterium	Firmicutes	Bacteria
CAG00773	1519	0	0.333	unclassified Firmicutes	unclassified Firmicutes	Firmicutes	Bacteria
CAG00782	1500	0	0.333	Eubacterium rectale	Eubacterium	Firmicutes	Bacteria
CAG00791	1492	0	0.333	Clostridium sp. CAG:221 - Clostridium sp.	unclassified Clostridiales	Firmicutes	Bacteria
CAG00794	1489	0	0.333	Firmicutes bacterium CAG:536	unclassified Firmicutes	Firmicutes	Bacteria
CAG00805	1477	0	0.333	unclassified Ruminococcaceae	unclassified Ruminococcaceae	Firmicutes	Bacteria
CAG00807	1474	0	0.333	unclassified Holdemanella	Holdemanella	Firmicutes	Bacteria
CAG00808	1471	0	0.333	unclassified Firmicutes	unclassified Firmicutes	Firmicutes	Bacteria
CAG00814	1464	0	0.333	Eubacterium sp. CAG:192	Eubacterium	Firmicutes	Bacteria
CAG00819	1462	0	0.333	Acidaminococcus sp. CAG:542	Acidaminococcus	Firmicutes	Bacteria
CAG00828	1453	0	0.333	unclassified Oscillibacter	Oscillibacter	Firmicutes	Bacteria
CAG00862	1390	0	0.333	Firmicutes bacterium CAG:129 & unculti	unclassified Firmicutes	Firmicutes	Bacteria
CAG00869	1369	0	0.333	unclassified Roseburia	Roseburia	Firmicutes	Bacteria
CAG00871	1362	0	0.333	unclassified Lachnospiraceae	unclassified Lachnospiraceae	Firmicutes	Bacteria
CAG00873	1359	0	0.333	unclassified Butyricimonas	Butyricimonas	Bacteroidetes	Bacteria
CAG00880	1352	0	0.333	Subdoligranulum sp. CAG:314	Subdoligranulum	Firmicutes	Bacteria
CAG00890	1327	0	0.333	unclassified Clostridiales	unclassified Clostridiales	Firmicutes	Bacteria
CAG00893	1324	0	0.333	unclassified Clostridiales	unclassified Clostridiales	Firmicutes	Bacteria
CAG00897	1319	0	0.333	Oscillibacter sp.	Oscillibacter	Firmicutes	Bacteria
CAG00920	1280	0	0.333	unclassified	unclassified	unclassified	unclassified
CAG00941	1240	0	0.333	unclassified Oscillibacter	Oscillibacter	Firmicutes	Bacteria
CAG00949	1224	0	0.333	Ruminococcaceae bacterium D16	unclassified Ruminococcaceae	Firmicutes	Bacteria
CAG00965	1210	0	0.333	unclassified Firmicutes	unclassified Firmicutes	Firmicutes	Bacteria
CAG00969	1208	0	0.333	Clostridium sp. L2-50	unclassified Clostridiales	Firmicutes	Bacteria
CAG00974	1202	0	0.333	unclassified Eubacterium	Eubacterium	Firmicutes	Bacteria
CAG00987	1178	0	0.333	Megasphaera micronuciformis	Megasphaera	Firmicutes	Bacteria
CAG01016	1138	0	0.333	unclassified Ruminococcaceae	unclassified Ruminococcaceae	Firmicutes	Bacteria
CAG01043	1094	0	0.333	unclassified Firmicutes	unclassified Firmicutes	Firmicutes	Bacteria
CAG01065	1068	0	0.333	unclassified Lachnospiraceae	unclassified Lachnospiraceae	Firmicutes	Bacteria
CAG01070	1060	0	0.333	unclassified Clostridiales	unclassified Clostridiales	Firmicutes	Bacteria
CAG01116	999	0	0.333	unclassified Firmicutes	unclassified Firmicutes	Firmicutes	Bacteria
CAG01141	964	0	0.333	Holdemanella biformis	Holdemanella	Firmicutes	Bacteria
CAG01146	956	0	0.333	unclassified Oscillibacter	Oscillibacter	Firmicutes	Bacteria
CAG01190	879	0	0.333	Butyricimonas virosa	Butyricimonas	Bacteroidetes	Bacteria
CAG01195	867	0	0.333	unclassified Ruminococcaceae	unclassified Ruminococcaceae	Firmicutes	Bacteria
CAG01207	853	0	0.333	unclassified Oscillibacter	Oscillibacter	Firmicutes	Bacteria
CAG01217	828	0	0.333	unclassified Clostridiales	unclassified Clostridiales	Firmicutes	Bacteria
CAG01228	814	0	0.333	unclassified Lachnoclostridium	Lachnoclostridium	Firmicutes	Bacteria
CAG01237	799	0	0.333	unclassified Oscillibacter	Oscillibacter	Firmicutes	Bacteria
CAG01238	797	0	0.333	butyrate-producing bacterium SS3/4 - Cl	unclassified Clostridiales	Firmicutes	Bacteria

CAG01242	789	0	0.333	unclassified	unclassified	unclassified	unclassified
CAG01268	742	0	0.333	unclassified Lachnospiraceae	unclassified Lachnospiraceae	Firmicutes	Bacteria
CAG01321	636	0	0.333	Faecalibacterium 1	Faecalibacterium	Firmicutes	Bacteria
CAG01327	630	0	0.333	unclassified	unclassified	unclassified	unclassified
CAG01368	570	0	0.333	unclassified Bacteroidales	unclassified Bacteroidales	Bacteroidetes	Bacteria
CAG01369	570	0	0.333	Roseburia intestinalis	Roseburia	Firmicutes	Bacteria
CAG01400	522	0	0.333	unclassified Lachnospiraceae	unclassified Lachnospiraceae	Firmicutes	Bacteria
CAG01420	484	0	0.333	unclassified Clostridiales	unclassified Clostridiales	Firmicutes	Bacteria
CAG01435	456	0	0.333	unclassified Faecalibacterium	Faecalibacterium	Firmicutes	Bacteria
CAG01540	341	0	0.333	unclassified Clostridiales	unclassified Clostridiales	Firmicutes	Bacteria
CAG01553	333	0	0.333	unclassified Clostridiales	unclassified Clostridiales	Firmicutes	Bacteria
CAG01702	248	0	0.333	Porphyromonas asaccharolytica	Porphyromonas	Bacteroidetes	Bacteria
CAG01775	214	0	0.333	unclassified	unclassified	unclassified	unclassified
CAG02062	147	0	0.333	unclassified	unclassified	unclassified	unclassified
CAG02090	141	0	0.333	Anaerotruncus sp. CAG:528	Anaerotruncus	Firmicutes	Bacteria
CAG02437	95	0	0.333	unclassified Lachnoclostridium	Lachnoclostridium	Firmicutes	Bacteria
CAG02742	74	0	0.333	unclassified Firmicutes	unclassified Firmicutes	Firmicutes	Bacteria
CAG03218	55	0	0.333	Clostridium clostridioforme	Lachnoclostridium	Firmicutes	Bacteria
CAG00011	5523	0	0.333	Escherichia coli	Escherichia	Proteobacteria	Bacteria
CAG00028	4343	0	0.667	Klebsiella oxytoca	Klebsiella	Proteobacteria	Bacteria
CAG00030	4179	0	0.667	Enterobacter aerogenes	Enterobacter	Proteobacteria	Bacteria
CAG00069	3167	0	0.333	Morganella morganii	Morganella	Proteobacteria	Bacteria
CAG00078	3084	0	0.333	Proteus mirabilis	Proteus	Proteobacteria	Bacteria
CAG00104	2864	0	0.333	Enterobacter cloacae 4	Enterobacter	Proteobacteria	Bacteria
CAG00124	2742	0	0.667	Klebsiella variicola	Klebsiella	Proteobacteria	Bacteria
CAG00416	1982	0	0.333	Lelliottia amnigena	Lelliottia	Proteobacteria	Bacteria
CAG00878	1352	0	0.333	Citrobacter koseri	Citrobacter	Proteobacteria	Bacteria
CAG00883	1341	0	0.333	Escherichia coli	Escherichia	Proteobacteria	Bacteria
CAG01652	271	0	0.333	Escherichia coli	Escherichia	Proteobacteria	Bacteria
CAG02158	128	0	0.333	Escherichia coli	Escherichia	Proteobacteria	Bacteria
CAG01022	1126	0	0.333	Haemophilus paraphrohaemolyticus	Haemophilus	Proteobacteria	Bacteria
CAG01290	688	0	0.333	Enterobacter cloacae 1	Enterobacter	Proteobacteria	Bacteria
CAG01425	475	0	0.333	Escherichia fergusonii	Escherichia	Proteobacteria	Bacteria
CAG00018_1	1509	0	0.333	Klebsiella oxytoca	Klebsiella	Proteobacteria	Bacteria
CAG01099	1016	0	0.333	Raoultella ornithinolytica	Raoultella	Proteobacteria	Bacteria
CAG02057	147	0	0.333	unclassified	unclassified	unclassified	unclassified
