# Supplementary material for: Human and preclinical studies of the host–gut microbiome co-metabolite hippurate as a marker and mediator of metabolic health
Source: Gut. 2021 May 11;70(11):2105–14. doi: 10.1136/gutjnl-2020-323314 (PMC8515120; doi:10.1136/gutjnl-2020-323314)
Supplement: Supplementary data [file gutjnl-2020-323314supp016.pdf]

Supplementary Table ST.7. Association between the microbiota composition profiled as metagenomic OTUs (mOTUs) and urine hippurate levels.

Metabolite	EggNOG	Spearman ρ	P	FDR
hippurate	[Ruminococcus] gnavus	-0.3775	1.31E-10	7.24E-08
hippurate	Clostridium bolteae	-0.366	5.14E-10	1.42E-07
hippurate	motu_linkage_group_327	0.3497	3.24E-09	5.97E-07
hippurate	motu_linkage_group_307	0.3289	2.95E-08	4.08E-06
hippurate	Lachnospiraceae bacterium 1_4_56FA	-0.3119	1.59E-07	1.76E-05
hippurate	motu_linkage_group_644	0.3064	2.67E-07	2.46E-05
hippurate	motu_linkage_group_383	0.3005	4.64E-07	3.66E-05
hippurate	motu_linkage_group_611	0.2978	5.91E-07	4.09E-05
hippurate	Coprococcus eutactus	0.2911	1.08E-06	6.16E-05
hippurate	motu_linkage_group_308	0.2898	1.21E-06	6.16E-05
hippurate	motu_linkage_group_517	0.2897	1.23E-06	6.16E-05
hippurate	motu_linkage_group_456	-0.2798	2.89E-06	0.000128
hippurate	motu_linkage_group_346	0.2794	0.000003	0.000128
hippurate	motu_linkage_group_277	0.272	5.54E-06	0.000208
hippurate	Clostridium symbiosum	-0.2718	5.64E-06	0.000208
hippurate	motu_linkage_group_306	0.27	6.54E-06	0.000226
hippurate	motu_linkage_group_672	0.2674	8.09E-06	0.000263
hippurate	motu_linkage_group_460	0.2588	0.000016	0.000492
hippurate	motu_linkage_group_451	-0.2566	0.000019	0.000553
hippurate	motu_linkage_group_496	0.2546	2.21E-05	0.000611
hippurate	motu_linkage_group_334	0.2513	2.85E-05	0.000703
hippurate	motu_linkage_group_113	0.2511	0.000029	0.000703
hippurate	unclassified Lachnospiraceae	-0.2503	3.07E-05	0.000703
hippurate	motu_linkage_group_434	0.2502	0.000031	0.000703
hippurate	motu_linkage_group_652	0.2499	3.18E-05	0.000703
hippurate	Holdemania filiformis	-0.2469	3.96E-05	0.000829
hippurate	motu_linkage_group_691	0.2466	4.05E-05	0.000829
hippurate	motu_linkage_group_252	0.2453	4.47E-05	0.000863
hippurate	Clostridium ramosum	-0.2449	0.000046	0.000863
hippurate	Methanobrevibacter smithii	0.2447	4.68E-05	0.000863
hippurate	motu_linkage_group_470	0.2435	5.08E-05	0.000906
hippurate	motu_linkage_group_207	0.2409	6.15E-05	0.001061
hippurate	motu_linkage_group_87	0.2405	6.33E-05	0.001061
hippurate	motu_linkage_group_686	0.2393	0.000069	0.001122
hippurate	Clostridiales bacterium 1_7_47FAA	-0.2368	8.27E-05	0.001307
hippurate	motu_linkage_group_241	0.2341	0.0001	0.001536
hippurate	motu_linkage_group_212	0.2334	0.000105	0.001569
hippurate	motu_linkage_group_244	0.2328	0.00011	0.001601
hippurate	motu_linkage_group_504	0.2311	0.000124	0.001758
hippurate	motu_linkage_group_238	0.2302	0.000132	0.001825
hippurate	motu_linkage_group_463	0.2269	0.000165	0.002225
hippurate	motu_linkage_group_525	0.2261	0.000175	0.00225
hippurate	motu_linkage_group_771	0.2258	0.000178	0.00225
hippurate	Eubacterium siraeum	0.2257	0.000179	0.00225
hippurate	motu_linkage_group_651	0.2251	0.000186	0.002286
hippurate	motu_linkage_group_57	0.2233	0.00021	0.002525
hippurate	motu_linkage_group_317	0.2228	0.000218	0.002565
hippurate	motu_linkage_group_604	0.222	0.00023	0.00265
hippurate	Ruminococcus sp. 5_1_39BFAA	-0.2215	0.000238	0.002677

hippurate	Bacteroides dorei/vulgatus	-0.2212	0.000242	0.002677
hippurate	motu_linkage_group_407	0.2199	0.000264	0.002863
hippurate	[Ruminococcus] torques	-0.2185	0.000289	0.003031
hippurate	motu_linkage_group_638	0.2183	0.000293	0.003031
hippurate	[Bacteroides] pectinophilus	0.2182	0.000296	0.003031
hippurate	Anaerotruncus colihominis	-0.2173	0.000313	0.003147
hippurate	motu_linkage_group_734	0.2151	0.000362	0.003541
hippurate	motu_linkage_group_551	-0.215	0.000365	0.003541
hippurate	motu_linkage_group_103	0.2125	0.000428	0.004021
hippurate	Eggerthella lenta	-0.2125	0.000429	0.004021
hippurate	motu_linkage_group_531	0.2115	0.000457	0.004212
hippurate	motu_linkage_group_260	0.2101	0.0005	0.004533
hippurate	motu_linkage_group_376	0.2082	0.000562	0.005013
hippurate	motu_linkage_group_142	0.2065	0.000624	0.005477
hippurate	motu_linkage_group_625	0.2052	0.000677	0.00585
hippurate	motu_linkage_group_72	0.2048	0.000694	0.005904
hippurate	motu_linkage_group_430	0.2037	0.000744	0.006157
hippurate	motu_linkage_group_145	0.2036	0.000746	0.006157
hippurate	motu_linkage_group_124	0.2022	0.000813	0.006612
hippurate	motu_linkage_group_678	0.2011	0.000872	0.006952
hippurate	motu_linkage_group_122	0.2009	0.00088	0.006952
hippurate	Erysipelotrichaceae bacterium 3_1_53	-0.2006	0.000899	0.006957
hippurate	motu_linkage_group_565	0.2004	0.000906	0.006957
hippurate	motu_linkage_group_567	-0.2001	0.000926	0.006957
hippurate	Butyrivibrio crossotus	0.2	0.000931	0.006957
hippurate	motu_linkage_group_508	0.1995	0.000958	0.007064
hippurate	motu_linkage_group_190	0.1988	0.001	0.007276
hippurate	Collinsella stercoris	-0.197	0.001111	0.007969
hippurate	motu_linkage_group_121	0.1968	0.001124	0.007969
hippurate	motu_linkage_group_636	0.1955	0.001214	0.008498
hippurate	motu_linkage_group_219	0.195	0.001258	0.008696
hippurate	Blautia hydrogenotrophica	-0.1939	0.001335	0.009114
hippurate	Clostridium scindens	-0.1912	0.001564	0.010547
hippurate	motu_linkage_group_529	0.1905	0.001634	0.01079
hippurate	motu_linkage_group_136	0.1904	0.001639	0.01079
hippurate	motu_linkage_group_600	0.1886	0.001823	0.01186
hippurate	Bacteroides xylanisolvens	-0.1876	0.001925	0.012378
hippurate	motu_linkage_group_258	0.1872	0.001971	0.012528
hippurate	motu_linkage_group_243	0.1868	0.002015	0.012662
hippurate	motu_linkage_group_586	0.186	0.002105	0.013045
hippurate	motu_linkage_group_68	0.1859	0.002123	0.013045
hippurate	motu_linkage_group_349	-0.1856	0.002161	0.013132
hippurate	Dialister invisus	-0.1839	0.002365	0.014207
hippurate	motu_linkage_group_226	0.1836	0.002408	0.014207
hippurate	motu_linkage_group_85	0.1836	0.002415	0.014207
hippurate	butyrate-producing bacterium	-0.1825	0.002566	0.014931
hippurate	motu_linkage_group_490	-0.1823	0.002592	0.014931
hippurate	Eubacterium eligens	0.1809	0.002797	0.015595
hippurate	motu_linkage_group_404	0.1808	0.002814	0.015595
hippurate	motu_linkage_group_566	0.1808	0.002817	0.015595
hippurate	motu_linkage_group_415	0.1808	0.00282	0.015595
hippurate	motu_linkage_group_375	0.1802	0.002904	0.0159

hippurate	Pseudoflavonifractor capillosus	-0.1797	0.002995	0.016238
hippurate	motu_linkage_group_217	0.1785	0.003193	0.017085
hippurate	Anaerostipes caccae	-0.1784	0.003213	0.017085
hippurate	motu_linkage_group_120	0.176	0.003655	0.01925
hippurate	motu_linkage_group_218	0.1756	0.003723	0.019423
hippurate	Erysipelotrichaceae bacterium 5_2_54	-0.1743	0.00399	0.020621
hippurate	motu_linkage_group_362	0.1738	0.0041	0.020994
hippurate	motu_linkage_group_20	0.1729	0.004314	0.021788
hippurate	motu_linkage_group_281	-0.1728	0.004334	0.021788
hippurate	motu_linkage_group_324	0.1715	0.004648	0.022994
hippurate	motu_linkage_group_485	0.1714	0.004657	0.022994
hippurate	motu_linkage_group_509	0.1704	0.004909	0.024024
hippurate	Turicibacter sanguinis	0.1699	0.005029	0.024395
hippurate	Campylobacter hominis	0.1687	0.005371	0.025828
hippurate	Victivallis vadensis	0.1684	0.005436	0.025915
hippurate	motu_linkage_group_622	0.1676	0.005673	0.026675
hippurate	motu_linkage_group_54	0.1675	0.005692	0.026675
hippurate	motu_linkage_group_548	0.1666	0.005985	0.027813
hippurate	motu_linkage_group_320	0.163	0.007179	0.033083
hippurate	Eubacterium dolichum	-0.162	0.007533	0.034428
hippurate	Bacteroides thetaiotaomicron	-0.1606	0.008076	0.036607
hippurate	motu_linkage_group_386	0.1598	0.008396	0.037748
hippurate	Clostridium sp. HGF2	-0.1592	0.008668	0.038592
hippurate	Eubacterium cylindroides	0.159	0.008732	0.038592
hippurate	motu_linkage_group_624	-0.1588	0.008835	0.038592
hippurate	motu_linkage_group_288	0.1587	0.008863	0.038592
hippurate	motu_linkage_group_411	0.1571	0.009584	0.041406
hippurate	motu_linkage_group_224	0.1551	0.010571	0.045316
hippurate	motu_linkage_group_450	-0.1542	0.011024	0.046894
hippurate	motu_linkage_group_102	0.154	0.011133	0.046997
hippurate	motu_linkage_group_164	0.1529	0.011721	0.049104
hippurate	motu_linkage_group_257	0.1526	0.011905	0.0495
hippurate	motu_linkage_group_282	0.1513	0.012657	0.052234
hippurate	motu_linkage_group_385	0.1499	0.013474	0.054975
hippurate	motu_linkage_group_486	0.1499	0.01352	0.054975
hippurate	motu_linkage_group_176	-0.1486	0.014367	0.057992
hippurate	motu_linkage_group_248	0.1483	0.014547	0.058293
hippurate	motu_linkage_group_342	0.1478	0.014864	0.059135
hippurate	Ruminococcaceae bacterium D16	-0.1465	0.01581	0.062026
hippurate	motu_linkage_group_325	0.1465	0.015815	0.062026
hippurate	Tropheryma whipplei	-0.1458	0.016319	0.063552
hippurate	motu_linkage_group_151	0.1454	0.016613	0.064245
hippurate	motu_linkage_group_45	0.1451	0.016864	0.064762
hippurate	Granulicatella adiacens	-0.1448	0.017066	0.064784
hippurate	motu_linkage_group_621	0.1448	0.017104	0.064784
hippurate	motu_linkage_group_712	0.1445	0.017295	0.065062
hippurate	motu_linkage_group_74	0.1442	0.017523	0.065474
hippurate	motu_linkage_group_127	0.1431	0.018387	0.067982
hippurate	motu_linkage_group_51	0.1431	0.01844	0.067982
hippurate	Acidaminococcus fermentans	0.1413	0.019949	0.073058
hippurate	motu_linkage_group_25	0.1408	0.020419	0.074275
hippurate	motu_linkage_group_431	0.1406	0.02055	0.074275

hippurate	motu_linkage_group_457	0.1369	0.024197	0.086889
hippurate	motu_linkage_group_341	0.1356	0.025565	0.091209
hippurate	Streptococcus parasanguinis	0.1349	0.026325	0.093319
hippurate	butyrate-producing bacterium SS3/4	-0.134	0.027417	0.096571
hippurate	Eubacterium biforme	0.1331	0.028491	0.099719
hippurate	motu_linkage_group_177	0.1328	0.028793	0.100142
hippurate	Streptococcus sp. C150	0.1324	0.02927	0.101164
hippurate	Roseburia hominis	0.1309	0.031271	0.107409
hippurate	Methanosphaera stadtmanae	0.1299	0.032593	0.111259
hippurate	motu_linkage_group_270	0.129	0.033763	0.114546
hippurate	Bifidobacterium angulatum	0.1287	0.034175	0.115236
hippurate	motu_linkage_group_192	-0.1284	0.034555	0.115812
hippurate	motu_linkage_group_765	-0.1275	0.035996	0.119637
hippurate	Streptococcus salivarius	0.1274	0.036129	0.119637
hippurate	Oribacterium sinus	0.1258	0.038563	0.126937
hippurate	motu_linkage_group_389	0.1239	0.041503	0.135391
hippurate	Clostridium spiroforme	-0.1237	0.041857	0.135391
hippurate	motu_linkage_group_559	0.1237	0.041866	0.135391
hippurate	motu_linkage_group_10	0.123	0.043074	0.138488
hippurate	motu_linkage_group_480	0.1223	0.044236	0.141402
hippurate	Rothia mucilaginosa	0.122	0.044789	0.142347
hippurate	Staphylococcus aureus	-0.1216	0.045493	0.143758
hippurate	Clostridium sp. L2-50	0.1215	0.045758	0.143774
hippurate	Clostridium saccharolyticum	-0.1212	0.046215	0.144389
hippurate	motu_linkage_group_634	0.121	0.04657	0.144681
hippurate	Ruminococcus bromii	0.1205	0.047446	0.146158
hippurate	motu_linkage_group_703	0.1205	0.047574	0.146158
hippurate	motu_linkage_group_286	-0.12	0.048387	0.147834
hippurate	Aggregatibacter segnis	-0.1193	0.049776	0.150489
hippurate	Blautia hansenii	-0.1193	0.0498	0.150489
hippurate	motu_linkage_group_179	0.1189	0.050602	0.152081
hippurate	motu_linkage_group_592	0.1186	0.051133	0.152081
hippurate	motu_linkage_group_52	0.1186	0.051205	0.152081
hippurate	motu_linkage_group_209	0.1185	0.05144	0.152081
hippurate	Prevotella copri	0.1183	0.051702	0.152081
hippurate	motu_linkage_group_499	0.1145	0.059758	0.174847
hippurate	Lactobacillus salivarius	0.1143	0.060164	0.175109
hippurate	Gemella morbillorum	-0.114	0.060992	0.176589
hippurate	Lactobacillus casei	0.1136	0.061902	0.178291
hippurate	motu_linkage_group_14	0.1128	0.063784	0.182759
hippurate	Subdoligranulum variabile	0.1125	0.064375	0.183502
hippurate	Parvimonas micra	-0.1119	0.065899	0.186883
hippurate	motu_linkage_group_301	0.1102	0.070104	0.197793
hippurate	motu_linkage_group_42	-0.1097	0.071446	0.200557
hippurate	motu_linkage_group_161	0.1094	0.07221	0.201677
hippurate	[Ruminococcus] obeum	0.1091	0.07293	0.202665
hippurate	Salmonella enterica	-0.1084	0.074721	0.205763
hippurate	Streptococcus mutans	0.1084	0.074789	0.205763
hippurate	motu_linkage_group_111	0.1081	0.075565	0.206869
hippurate	motu_linkage_group_18	0.1076	0.077055	0.209908
hippurate	motu_linkage_group_348	0.1072	0.078115	0.211753
hippurate	Streptococcus sp. oral taxon 071	-0.1066	0.079871	0.215055

hippurate	Clostridium methylpentosum	-0.1065	0.080111	0.215055
hippurate	motu_linkage_group_38	0.1063	0.080611	0.215352
hippurate	Streptococcus constellatus	-0.1058	0.082149	0.218406
hippurate	motu_linkage_group_448	0.1051	0.084155	0.222668
hippurate	motu_linkage_group_471	0.1049	0.08474	0.223149
hippurate	Mitsuokella multacida	0.1046	0.085695	0.224594
hippurate	Bulleidia extructa	-0.1043	0.08655	0.225765
hippurate	Clostridium hylemonae	-0.1035	0.089142	0.23096
hippurate	Bacteroides uniformis	-0.1034	0.089377	0.23096
hippurate	motu_linkage_group_373	-0.1029	0.090973	0.233991
hippurate	Catonella morbi	-0.1019	0.094082	0.240867
hippurate	Parabacteroides distasonis	-0.1018	0.094564	0.240986
hippurate	Enterobacter asburiae	0.1011	0.096633	0.245129
hippurate	motu_linkage_group_33	0.1008	0.097881	0.247161
hippurate	motu_linkage_group_510	0.1	0.10053	0.250773
hippurate	motu_linkage_group_445	0.1	0.100591	0.250773
hippurate	motu_linkage_group_574	0.0999	0.100672	0.250773
hippurate	motu_linkage_group_443	-0.0994	0.102604	0.25444
hippurate	Lachnospiraceae bacterium 3_1_57FA	-0.0992	0.103149	0.254649
hippurate	Haemophilus parainfluenzae	0.0988	0.104504	0.25599
hippurate	Fusobacterium nucleatum	-0.0988	0.104618	0.25599
hippurate	motu_linkage_group_837	0.0985	0.105678	0.257445
hippurate	Enterobacter hormaechei/cloacae	0.0977	0.108469	0.262732
hippurate	motu_linkage_group_613	0.0973	0.109987	0.262732
hippurate	Neisseria subflava	-0.0972	0.110224	0.262732
hippurate	Prevotella tannerae	-0.0972	0.110224	0.262732
hippurate	Selenomonas noxia	-0.0972	0.110224	0.262732
hippurate	Olsenella uli	0.097	0.111137	0.263772
hippurate	motu_linkage_group_366	0.0968	0.111936	0.263993
hippurate	Escherichia coli	-0.0967	0.112185	0.263993
hippurate	Streptococcus australis	0.0963	0.113628	0.266255
hippurate	Roseburia inulinivorans	-0.0957	0.115849	0.270314
hippurate	motu_linkage_group_71	0.0953	0.11771	0.272751
hippurate	motu_linkage_group_378	-0.0951	0.118298	0.272751
hippurate	motu_linkage_group_528	0.0951	0.118373	0.272751
hippurate	Prevotella multisaccharivorax	0.0949	0.119072	0.273223
hippurate	Ruminococcus lactaris	0.0943	0.121576	0.277003
hippurate	motu_linkage_group_205	0.0942	0.121721	0.277003
hippurate	[Clostridium] bartlettii	0.0927	0.127773	0.289584
hippurate	motu_linkage_group_534	0.092	0.131027	0.295747
hippurate	Acidaminococcus intestini	-0.0909	0.135643	0.303722
hippurate	motu_linkage_group_617	0.0908	0.13613	0.303722
hippurate	Bacteroides fragilis	-0.0907	0.136208	0.303722
hippurate	Lactobacillus crispatus	-0.0902	0.13842	0.306185
hippurate	Prevotella melaninogenica	-0.0902	0.13842	0.306185
hippurate	motu_linkage_group_360	0.0897	0.140679	0.309942
hippurate	motu_linkage_group_26	0.0893	0.142374	0.312126
hippurate	motu_linkage_group_185	0.0893	0.142799	0.312126
hippurate	motu_linkage_group_168	0.0884	0.146719	0.31796
hippurate	Streptococcus mitis	0.0883	0.147265	0.31796
hippurate	Bilophila wadsworthia	-0.0883	0.14729	0.31796
hippurate	motu_linkage_group_3	0.088	0.148556	0.31796

hippurate	motu_linkage_group_280	0.088	0.148641	0.31796
hippurate	Fusobacterium ulcerans	-0.0879	0.148956	0.31796
hippurate	Ruminococcus champanellensis	0.0878	0.149493	0.31796
hippurate	Streptococcus pneumoniae	0.0876	0.150207	0.318255
hippurate	Lactobacillus sakei	-0.0874	0.151426	0.318757
hippurate	Prevotella buccae	-0.0873	0.151597	0.318757
hippurate	motu_linkage_group_194	-0.0871	0.152953	0.32039
hippurate	Peptostreptococcus anaerobius	0.0865	0.155481	0.324457
hippurate	motu_linkage_group_562	0.0859	0.158319	0.329137
hippurate	motu_linkage_group_211	0.0852	0.161812	0.335139
hippurate	Bacteroides ovatus	-0.0848	0.164097	0.338337
hippurate	Clostridium sp. 7_2_43FAA	0.0847	0.16458	0.338337
hippurate	motu_linkage_group_398	0.0841	0.167405	0.34287
hippurate	motu_linkage_group_314	0.0837	0.169652	0.34619
hippurate	Streptococcus thermophilus	0.0831	0.172455	0.350393
hippurate	Campylobacter concisus	-0.083	0.172979	0.350393
hippurate	Prevotella dentalis	0.082	0.178524	0.360306
hippurate	Weissella cibaria	-0.0812	0.18259	0.366249
hippurate	Coprococcus catus	0.0811	0.183047	0.366249
hippurate	Slackia exigua	-0.081	0.183633	0.366249
hippurate	Prevotella oralis	-0.0809	0.184215	0.366249
hippurate	motu_linkage_group_295	0.0808	0.18478	0.366249
hippurate	Proteus mirabilis	-0.0806	0.186011	0.367372
hippurate	Paraprevotella xylaniphila	-0.0804	0.186771	0.36756
hippurate	Lactobacillus ruminis	0.0796	0.191252	0.375044
hippurate	Fusobacterium mortiferum	-0.0793	0.192824	0.376359
hippurate	Bacteroides coprocola	0.0793	0.193284	0.376359
hippurate	motu_linkage_group_333	0.0791	0.194059	0.376543
hippurate	Enterococcus casseliflavus	0.0778	0.201724	0.388504
hippurate	Streptococcus anginosus	0.0777	0.201976	0.388504
hippurate	motu_linkage_group_65	0.0777	0.202331	0.388504
hippurate	Prevotella bivia	-0.0774	0.204013	0.390378
hippurate	Oxalobacter formigenes	0.0773	0.20487	0.390666
hippurate	motu_linkage_group_532	0.077	0.2064	0.392231
hippurate	Porphyromonas asaccharolytica	0.0768	0.207798	0.393535
hippurate	Enterococcus faecium	0.0763	0.210301	0.396916
hippurate	motu_linkage_group_240	0.0762	0.211182	0.397223
hippurate	Marvinbryantia formatexigens	-0.076	0.212525	0.398394
hippurate	Bifidobacterium dentium	0.0757	0.213938	0.399688
hippurate	Streptococcus sanguinis	0.0752	0.217494	0.404964
hippurate	motu_linkage_group_786	-0.0748	0.219659	0.406742
hippurate	Aggregatibacter aphrophilus	-0.0748	0.21992	0.406742
hippurate	motu_linkage_group_131	-0.0745	0.221561	0.408411
hippurate	Lactobacillus delbrueckii	0.0742	0.223446	0.410517
hippurate	Bacteroides coprophilus	0.074	0.224928	0.411144
hippurate	motu_linkage_group_474	0.0739	0.225274	0.411144
hippurate	motu_linkage_group_182	0.0733	0.229159	0.414703
hippurate	Gemella sanguinis	0.0733	0.229259	0.414703
hippurate	motu_linkage_group_488	0.0732	0.229474	0.414703
hippurate	Lactococcus garvieae	-0.0723	0.235209	0.423683
hippurate	Bacteroides eggerthii	0.0721	0.236729	0.425036
hippurate	Veillonella atypica	0.0717	0.239459	0.428546

hippurate	Enterobacter cancerogenus	0.0715	0.240998	0.429409
hippurate	motu_linkage_group_573	0.0714	0.241494	0.429409
hippurate	Faecalibacterium prausnitzii	-0.0712	0.242635	0.430055
hippurate	motu_linkage_group_662	0.0709	0.244561	0.432084
hippurate	Eubacterium ventriosum	0.0707	0.246398	0.433943
hippurate	motu_linkage_group_297	0.0701	0.250361	0.439523
hippurate	motu_linkage_group_414	-0.0698	0.252377	0.441554
hippurate	motu_linkage_group_117	0.0697	0.253115	0.441554
hippurate	Arcobacter butzleri	-0.0692	0.256013	0.442492
hippurate	Desulfovibrio vulgaris	-0.0692	0.256013	0.442492
hippurate	motu_linkage_group_289	0.0692	0.256053	0.442492
hippurate	Prevotella buccalis	0.0689	0.258069	0.44335
hippurate	Ruminococcus sp. SR1/5	-0.0689	0.258153	0.44335
hippurate	Dorea formicigenerans	-0.0687	0.259818	0.444828
hippurate	Bifidobacterium breve	-0.0685	0.260785	0.445105
hippurate	motu_linkage_group_519	0.0681	0.264258	0.449645
hippurate	Parasutterella excrementihominis	0.0677	0.266682	0.452173
hippurate	Enterococcus faecalis	0.0676	0.267379	0.452173
hippurate	Atopobium parvulum	0.0673	0.269238	0.453929
hippurate	Veillonella dispar	0.0661	0.278497	0.468112
hippurate	Fusobacterium gonidiaformans	-0.0659	0.280006	0.469222
hippurate	motu_linkage_group_273	0.0657	0.280925	0.46934
hippurate	motu_linkage_group_4	0.0656	0.282015	0.469742
hippurate	Shuttleworthia satelles	-0.0645	0.290213	0.481945
hippurate	motu_linkage_group_552	-0.0639	0.294264	0.48721
hippurate	motu_linkage_group_797	0.0636	0.297132	0.488921
hippurate	motu_linkage_group_228	-0.0635	0.297661	0.488921
hippurate	motu_linkage_group_135	-0.0635	0.29795	0.488921
hippurate	motu_linkage_group_63	0.062	0.309103	0.505722
hippurate	Haemophilus influenzae	-0.0616	0.312346	0.508646
hippurate	motu_linkage_group_394	0.0615	0.31273	0.508646
hippurate	motu_linkage_group_171	0.0611	0.31657	0.512411
hippurate	motu_linkage_group_666	0.061	0.316898	0.512411
hippurate	motu_linkage_group_118	-0.0607	0.319405	0.513695
hippurate	Prevotella disiens	0.0607	0.31955	0.513695
hippurate	Citrobacter youngae	-0.0604	0.32189	0.515957
hippurate	motu_linkage_group_502	0.0598	0.32681	0.522191
hippurate	motu_linkage_group_267	0.0597	0.327668	0.522191
hippurate	Anaerococcus lactolyticus	-0.0591	0.332228	0.523425
hippurate	Brevibacterium linens	-0.0591	0.332228	0.523425
hippurate	Parvimonas sp. oral taxon 393	-0.0591	0.332228	0.523425
hippurate	motu_linkage_group_731	-0.0591	0.332228	0.523425
hippurate	motu_linkage_group_195	0.0588	0.334573	0.525622
hippurate	motu_linkage_group_230	0.0584	0.337974	0.527185
hippurate	Pyramidobacter piscolens	0.0583	0.33864	0.527185
hippurate	Bacteroides clarus	0.0583	0.338866	0.527185
hippurate	motu_linkage_group_49	0.0582	0.33945	0.527185
hippurate	Leuconostoc mesenteroides	-0.0581	0.340696	0.527185
hippurate	motu_linkage_group_495	0.058	0.341408	0.527185
hippurate	Desulfovibrio desulfuricans	-0.0579	0.342241	0.527185
hippurate	Acinetobacter baumannii	0.0576	0.345131	0.53016
hippurate	Oscillibacter valericigenes	0.0572	0.347821	0.532812

hippurate	Leuconostoc citreum	-0.0568	0.3517	0.537265
hippurate	motu_linkage_group_458	0.0562	0.356798	0.540189
hippurate	Collinsella aerofaciens	0.0562	0.356831	0.540189
hippurate	motu_linkage_group_234	0.0561	0.357509	0.540189
hippurate	Finegoldia magna	0.0561	0.357521	0.540189
hippurate	Alistipes shahii	0.0559	0.359361	0.541489
hippurate	motu_linkage_group_47	0.0552	0.364944	0.547113
hippurate	Streptococcus sp. M143	-0.0552	0.365072	0.547113
hippurate	motu_linkage_group_28	-0.0546	0.370505	0.553755
hippurate	motu_linkage_group_585	0.0545	0.371889	0.554325
hippurate	Streptococcus infantis	-0.0535	0.380369	0.564444
hippurate	Thermus scotoductus	-0.0535	0.380719	0.564444
hippurate	motu_linkage_group_646	-0.0518	0.395243	0.58441
hippurate	Anaerococcus vaginalis	0.0513	0.39986	0.589083
hippurate	unclassified Fusobacterium	-0.0512	0.401425	0.589083
hippurate	Enterobacteriaceae bacterium 9_2_54	0.0512	0.401599	0.589083
hippurate	Slackia heliotrinireducens	-0.0506	0.407049	0.594491
hippurate	Klebsiella oxytoca	0.0505	0.407436	0.594491
hippurate	motu_linkage_group_357	-0.0499	0.412826	0.598212
hippurate	Escherichia albertii	0.0499	0.41307	0.598212
hippurate	Bifidobacterium longum	0.0498	0.41407	0.598212
hippurate	Lactobacillus rhamnosus	0.0498	0.414313	0.598212
hippurate	Streptococcus peroris	-0.0495	0.416835	0.599952
hippurate	motu_linkage_group_254	0.0494	0.417688	0.599952
hippurate	Enterobacter aerogenes	-0.0491	0.420433	0.60233
hippurate	Clostridium leptum	-0.0481	0.429862	0.614247
hippurate	motu_linkage_group_521	0.0476	0.435002	0.617994
hippurate	Streptococcus gordonii	0.0475	0.435893	0.617994
hippurate	Streptococcus downei	0.0475	0.436558	0.617994
hippurate	Atopobium rimae	0.0473	0.437772	0.617994
hippurate	Solobacterium moorei	-0.0473	0.438072	0.617994
hippurate	Bacteroides finegoldii	0.0471	0.439608	0.618583
hippurate	Odoribacter splanchnicus	-0.0468	0.443356	0.622274
hippurate	motu_linkage_group_391	-0.0464	0.447022	0.62307
hippurate	motu_linkage_group_225	0.0462	0.448676	0.62307
hippurate	motu_linkage_group_491	-0.0462	0.449028	0.62307
hippurate	motu_linkage_group_24	0.0461	0.450138	0.62307
hippurate	motu_linkage_group_154	0.046	0.450291	0.62307
hippurate	Pseudoalteromonas haloplanktis	0.0459	0.451761	0.62307
hippurate	Bifidobacterium catenulatum-Bifidob	-0.0459	0.45181	0.62307
hippurate	Leuconostoc gelidum	0.0451	0.459473	0.632061
hippurate	motu_linkage_group_265	0.0446	0.464479	0.637362
hippurate	motu_linkage_group_344	0.0443	0.467763	0.64028
hippurate	motu_linkage_group_69	0.0438	0.472562	0.645251
hippurate	Delftia acidovorans	-0.0436	0.475114	0.646884
hippurate	motu_linkage_group_345	0.0435	0.476097	0.646884
hippurate	motu_linkage_group_204	0.0433	0.477636	0.647384
hippurate	motu_linkage_group_696	0.0427	0.483652	0.653935
hippurate	Gardnerella vaginalis	-0.0426	0.485083	0.654207
hippurate	motu_linkage_group_439	-0.0424	0.48664	0.654207
hippurate	Anaerofustis stercorihominis	-0.0424	0.487402	0.654207
hippurate	motu_linkage_group_484	0.0418	0.492866	0.659939

hippurate	motu_linkage_group_210	0.0415	0.496206	0.662807
hippurate	motu_linkage_group_115	-0.0414	0.497667	0.663156
hippurate	Granulicatella elegans	0.0412	0.499111	0.663482
hippurate	motu_linkage_group_29	0.0408	0.503698	0.667005
hippurate	motu_linkage_group_284	0.0407	0.504174	0.667005
hippurate	Collinsella intestinalis	-0.0404	0.50762	0.669961
hippurate	motu_linkage_group_101	0.0399	0.51267	0.675016
hippurate	Actinomyces sp. oral taxon 170	0.0397	0.515457	0.677073
hippurate	motu_linkage_group_588	-0.0395	0.51709	0.677608
hippurate	Haemophilus haemolyticus	-0.0387	0.526205	0.687923
hippurate	motu_linkage_group_481	0.0383	0.529978	0.691221
hippurate	Bacteroides plebeius	0.0379	0.534366	0.691847
hippurate	Peptoniphilus harei	-0.0379	0.534776	0.691847
hippurate	motu_linkage_group_363	0.0378	0.535966	0.691847
hippurate	Streptococcus oralis	0.0376	0.537232	0.691847
hippurate	motu_linkage_group_206	-0.0376	0.537475	0.691847
hippurate	Lactobacillus gasseri	0.0376	0.537964	0.691847
hippurate	Prevotella denticola	-0.0373	0.540485	0.692488
hippurate	motu_linkage_group_159	0.0373	0.540967	0.692488
hippurate	motu_linkage_group_293	-0.0368	0.546095	0.697438
hippurate	Desulfovibrio piger	0.0364	0.550716	0.701719
hippurate	Clostridium butyricum	-0.0362	0.55298	0.702122
hippurate	Fusobacterium periodonticum	-0.0361	0.553572	0.702122
hippurate	Treponema succinifaciens	-0.0358	0.5575	0.705486
hippurate	Capnocytophaga sp. oral taxon 329	0.0356	0.559946	0.706964
hippurate	Phascolarctobacterium succinatutens	0.0353	0.562883	0.709053
hippurate	Rothia dentocariosa	0.035	0.566103	0.711489
hippurate	motu_linkage_group_246	-0.0345	0.571317	0.716413
hippurate	Dorea longicatena	0.0334	0.584131	0.730815
hippurate	Succinatimonas hippei	-0.0333	0.585445	0.730815
hippurate	motu_linkage_group_437	0.033	0.588397	0.732846
hippurate	motu_linkage_group_191	0.0328	0.590566	0.733894
hippurate	Citrobacter koseri	-0.0324	0.595954	0.738776
hippurate	motu_linkage_group_379	0.0322	0.597206	0.738776
hippurate	motu_linkage_group_174	0.0321	0.598502	0.738776
hippurate	Leuconostoc kimchii	0.0319	0.601135	0.740373
hippurate	motu_linkage_group_13	0.0315	0.605284	0.742186
hippurate	Coprobacillus sp. 29_1	-0.0315	0.605291	0.742186
hippurate	Alistipes sp. HGB5	-0.0313	0.607708	0.742307
hippurate	Megasphaera micronuciformis	-0.0313	0.608074	0.742307
hippurate	motu_linkage_group_9	0.0309	0.612333	0.745859
hippurate	Streptococcus gallolyticus	0.0306	0.615515	0.746936
hippurate	motu_linkage_group_507	0.0306	0.615918	0.746936
hippurate	Leuconostoc gasicomitatum	0.0296	0.628031	0.759959
hippurate	Propionibacterium freudenreichii	-0.0293	0.631476	0.760572
hippurate	motu_linkage_group_274	-0.0292	0.63243	0.760572
hippurate	Lactobacillus plantarum	-0.0291	0.633499	0.760572
hippurate	motu_linkage_group_7	0.029	0.634039	0.760572
hippurate	Enterobacter cloacae	0.0289	0.636245	0.761417
hippurate	motu_linkage_group_17	0.0288	0.637497	0.761417
hippurate	Desulfovibrio sp. 3_1_syn3	0.0281	0.644912	0.767412
hippurate	motu_linkage_group_302	0.0281	0.645292	0.767412

hippurate	Streptococcus vestibularis	0.0277	0.649915	0.769893
hippurate	Streptococcus agalactiae	-0.0277	0.650163	0.769893
hippurate	Pseudomonas aeruginosa	0.027	0.658078	0.77649
hippurate	Bifidobacterium bifidum	0.027	0.658542	0.77649
hippurate	motu_linkage_group_290	0.0264	0.664917	0.782339
hippurate	Peptoniphilus lacrimalis	0.0258	0.673009	0.790178
hippurate	motu_linkage_group_32	0.0253	0.678568	0.794863
hippurate	motu_linkage_group_30	-0.0251	0.681103	0.794863
hippurate	motu_linkage_group_701	0.0251	0.681311	0.794863
hippurate	Akkermansia muciniphila	0.0249	0.683768	0.79605
hippurate	Klebsiella variicola/pneumoniae	-0.0243	0.689943	0.801364
hippurate	Prevotella timonensis	0.0242	0.691481	0.801364
hippurate	Eggerthella sp. YY7918	-0.0241	0.69268	0.801364
hippurate	Gemella haemolysans	-0.0237	0.697749	0.803403
hippurate	Alistipes putredinis	-0.0236	0.698621	0.803403
hippurate	Bacteroides caccae	0.0236	0.698801	0.803403
hippurate	motu_linkage_group_575	-0.0234	0.701721	0.804093
hippurate	motu_linkage_group_104	-0.0233	0.702309	0.804093
hippurate	motu_linkage_group_90	0.0228	0.708685	0.809717
hippurate	Acinetobacter lwoffii	-0.0226	0.711594	0.811364
hippurate	Peptostreptococcus stomatis	0.0217	0.72191	0.821433
hippurate	Eubacterium hallii	-0.0215	0.724092	0.822224
hippurate	Lactobacillus fermentum	-0.0208	0.733157	0.828712
hippurate	Atopobium vaginae	0.0204	0.73826	0.828712
hippurate	Helicobacter pylori	0.0204	0.73826	0.828712
hippurate	Actinomyces viscosus	-0.0202	0.740296	0.828712
hippurate	Lactobacillus reuteri	-0.0202	0.740296	0.828712
hippurate	Lautropia mirabilis	-0.0202	0.740296	0.828712
hippurate	Streptococcus pseudopneumoniae	-0.0202	0.740296	0.828712
hippurate	motu_linkage_group_536	-0.0198	0.745798	0.833184
hippurate	motu_linkage_group_316	0.0195	0.749652	0.835802
hippurate	motu_linkage_group_253	0.0189	0.756557	0.841803
hippurate	Proteus penneri	0.0186	0.760139	0.84409
hippurate	motu_linkage_group_279	0.0181	0.766877	0.849866
hippurate	Eubacterium limosum	0.0178	0.770718	0.850826
hippurate	Lactobacillus sanfranciscensis	0.0178	0.770821	0.850826
hippurate	motu_linkage_group_694	0.0175	0.773993	0.852626
hippurate	motu_linkage_group_70	0.0173	0.776556	0.853748
hippurate	motu_linkage_group_568	-0.0171	0.778993	0.854666
hippurate	Megasphaera elsdenii	-0.0169	0.781603	0.854666
hippurate	Coprococcus comes	0.0169	0.782027	0.854666
hippurate	Lactococcus lactis	-0.0164	0.787794	0.85927
hippurate	Bacteroides stercoris	0.0151	0.804267	0.875511
hippurate	motu_linkage_group_165	0.0149	0.807315	0.876066
hippurate	Citrobacter sp. 30_2	-0.0148	0.807945	0.876066
hippurate	Veillonella parvula	0.0147	0.81011	0.876694
hippurate	Lactobacillus acidophilus	0.0132	0.829048	0.895437
hippurate	motu_linkage_group_37	-0.0128	0.833476	0.898464
hippurate	Veillonella sp. oral taxon 158	0.0117	0.848521	0.910097
hippurate	motu_linkage_group_43	0.0116	0.849574	0.910097
hippurate	motu_linkage_group_520	-0.0115	0.850389	0.910097
hippurate	motu_linkage_group_140	-0.0115	0.85085	0.910097

hippurate	motu_linkage_group_633	0.0109	0.85767	0.915621
hippurate	motu_linkage_group_158	0.0108	0.859475	0.91578
hippurate	Actinomyces odontolyticus	0.0105	0.863208	0.916995
hippurate	Pediococcus pentosaceus	0.0104	0.865301	0.916995
hippurate	Bifidobacterium animalis	-0.0103	0.86559	0.916995
hippurate	Clostridium perfringens	0.0099	0.870553	0.920489
hippurate	Eubacterium rectale	0.0092	0.879655	0.92778
hippurate	motu_linkage_group_80	0.0092	0.880804	0.92778
hippurate	Haemophilus pittmaniae	0.0089	0.883454	0.928802
hippurate	Eubacterium saphenum	-0.0086	0.888485	0.932319
hippurate	motu_linkage_group_6	-0.008	0.895083	0.937464
hippurate	Bifidobacterium adolescentis	0.0079	0.897289	0.937998
hippurate	motu_linkage_group_550	-0.0072	0.906114	0.94394
hippurate	motu_linkage_group_251	0.0072	0.906387	0.94394
hippurate	motu_linkage_group_34	0.007	0.908811	0.944339
hippurate	Porphyromonas uenonis	-0.0069	0.910186	0.944339
hippurate	Bacteroides intestinalis	-0.0063	0.918197	0.950867
hippurate	Roseburia intestinalis	0.0061	0.920238	0.951199
hippurate	Sutterella wadsworthensis	0.005	0.935147	0.964267
hippurate	motu_linkage_group_497	0.0049	0.936368	0.964267
hippurate	Streptococcus cristatus	-0.0046	0.940125	0.965123
hippurate	motu_linkage_group_310	0.0042	0.944512	0.965123
hippurate	motu_linkage_group_727	0.0042	0.945724	0.965123
hippurate	motu_linkage_group_134	-0.0039	0.949431	0.965123
hippurate	Parabacteroides merdae	0.0038	0.950574	0.965123
hippurate	motu_linkage_group_498	0.0038	0.950637	0.965123
hippurate	motu_linkage_group_365	0.0038	0.950854	0.965123
hippurate	Bacteroides fluxus	-0.0037	0.951161	0.965123
hippurate	motu_linkage_group_268	-0.0036	0.953356	0.965579
hippurate	Peptoniphilus duerdenii	-0.0032	0.95876	0.968028
hippurate	motu_linkage_group_233	-0.0031	0.959275	0.968028
hippurate	Bacteroides salanitronis	0.0028	0.963306	0.970325
hippurate	motu_linkage_group_452	-0.002	0.973525	0.978835
hippurate	Clostridium sp. D5	-0.0012	0.984563	0.988137
hippurate	Leuconostoc lactis	-0.0008	0.989829	0.991622
hippurate	motu_linkage_group_126	-0.0006	0.992307	0.992307
