# Supplementary material for: Human and preclinical studies of the host–gut microbiome co-metabolite hippurate as a marker and mediator of metabolic health
Source: Gut. 2021 May 11;70(11):2105–14. doi: 10.1136/gutjnl-2020-323314 (PMC8515120; doi:10.1136/gutjnl-2020-323314)
Supplement: Supplementary data [file gutjnl-2020-323314supp017.pdf]

Supplementary Table ST.8. Urinary hippurate concentration distribution over enterotypes (N=271). The statistical difference over the four enterotypes was tested using a non-parametric Kruskal-Wallis test, followed by post hoc Dunn tests for pairwise differences (two-sided testing).

X	Y	Kruskal-Wallis ChiSquare	DF	Kruskal-Wallis P	Level	Level2	Score Mean Difference	Std Err Dif	Z	Effect size (r)	p-value	N
Enterotypes	Hippurate	41.7878	3	4.45E-09	Rum	Bact2	92.14	16.69	5.52	0.45524	2.04E-07	147
Enterotypes	Hippurate	41.7878	3	4.45E-09	Prev	Bact2	83.38	18.36	4.54	0.4984	3.36E-05	83
Enterotypes	Hippurate	41.7878	3	4.45E-09	Rum	Bact1	50.86	11.9	4.28	0.31183	0.00011	188
Enterotypes	Hippurate	41.7878	3	4.45E-09	Prev	Bact1	42.1	14.14	2.98	0.26733	0.01747	124
Enterotypes	Hippurate	41.7878	3	4.45E-09	Rum	Prev	8.75	12.68	0.69	0.05197	1	176
Enterotypes	Hippurate	41.7878	3	4.45E-09	Bact2	Bact1	-41.26	17.83	-2.31	-0.23747	0.12383	95
