# Supplementary material for: Human and preclinical studies of the host–gut microbiome co-metabolite hippurate as a marker and mediator of metabolic health
Source: Gut. 2021 May 11;70(11):2105–14. doi: 10.1136/gutjnl-2020-323314 (PMC8515120; doi:10.1136/gutjnl-2020-323314)
Supplement: Supplementary data [file gutjnl-2020-323314supp018.pdf]

Supplementary Table ST.9. Hippurate-associated phenylpropanoid modules' distribution over enterotypes (N=271). The statistical difference over the four enterotypes was tested using a non-parametric Kruskal-Wallis test (two-sided testing and adjusted for multiple testing (AdjP, BH method), followed by post hoc Dunn tests for pairwise differences).

X	Y	Kruskal-Wallis ChiSquare	DF	Kruskal-Wallis P	Adj PValue	Level	Level2	Score Mean Difference	Std Err Dif	Z	Effect size (r)	p-value	N
Enterotypes	MC0004	40.03616	3	1.05E-08	2.09E-08	Rum	Bact2	92.946759	16.69416	5.56762	0.45921	1.55E-07	147
Enterotypes	MC0004	40.03616	3	1.05E-08	2.09E-08	Prev	Bact2	76.446759	18.36292	4.163106	0.45696	0.000188	83
Enterotypes	MC0004	40.03616	3	1.05E-08	2.09E-08	Rum	Bact1	49.886029	11.89631	4.193403	0.305835	0.000165	188
Enterotypes	MC0004	40.03616	3	1.05E-08	2.09E-08	Prev	Bact1	33.386029	14.14298	2.360608	0.211989	0.10947	124
Enterotypes	MC0004	40.03616	3	1.05E-08	2.09E-08	Rum	Prev	16.482143	12.68382	1.299462	0.097951	1	176
Enterotypes	MC0004	40.03616	3	1.05E-08	2.09E-08	Bact2	Bact1	-43.04602	17.82805	-2.414511	-0.247724	0.094538	95
Enterotypes	MC0005	22.247132	3	5.79E-05	5.79E-05	Rum	Bact2	75.299074	16.54971	4.549872	0.375267	3.22E-05	147
Enterotypes	MC0005	22.247132	3	5.79E-05	5.79E-05	Prev	Bact2	60.800265	18.20402	3.339935	0.366606	0.005028	83
Enterotypes	MC0005	22.247132	3	5.79E-05	5.79E-05	Rum	Bact1	27.472549	11.79337	2.32949	0.169896	0.118999	188
Enterotypes	MC0005	22.247132	3	5.79E-05	5.79E-05	Rum	Prev	14.480952	12.57407	1.151652	0.086809	1	176
Enterotypes	MC0005	22.247132	3	5.79E-05	5.79E-05	Prev	Bact1	12.973739	14.0206	0.925334	0.083097	1	124
Enterotypes	MC0005	22.247132	3	5.79E-05	5.79E-05	Bact2	Bact1	-47.81182	17.67379	-2.705238	-0.277552	0.040953	95
