# Supplementary material for: Human and preclinical studies of the host–gut microbiome co-metabolite hippurate as a marker and mediator of metabolic health
Source: Gut. 2021 May 11;70(11):2105–14. doi: 10.1136/gutjnl-2020-323314 (PMC8515120; doi:10.1136/gutjnl-2020-323314)
Supplement: Supplementary data [file gutjnl-2020-323314supp020.pdf]

Supplementary Table ST.10. Mann-Whitney U test for dietary (PC1) stratification in each bioclinical or main food variable between high or low PC1 groups using Benjamini-Hochberg FDR correction.

Short name	Median		pFDR
	Low PC1	High PC1	
'Ageatvisit'	55.57	56.59	0.9551
'gender'	59.52	40.30	
'gender'	40.48	59.70	
BMI'	31.33	31.18	0.9551
'weight'	91.00	96.40	0.7023
'waist'	105.00	106.00	0.7087
'hip'	112.00	111.00	0.7087
'WBTOT_PFAT'	34.05	31.90	0.5361
'pgluc'	5.75	5.70	0.9551
'insu'	45.00	53.00	0.6169
'cpep'	723.00	797.00	0.6169
'Homair'	1.70	2.00	0.6169
'pchol'	5.40	5.70	0.9551
'hdl'	1.44	1.34	0.5361
'tri'	1.15	1.27	0.5361
'FFA'	0.49	0.45	0.9551
'wbc'	5.90	5.80	0.9551
'lymph'	1.80	1.80	0.9350
'CRP'	1.60	1.90	0.7087
'IL6'	15.92	17.00	0.9558
'TNFalfa'	16.76	6.32	0.6318
'FIAF'	83.28	80.85	0.9730
'leptin'	10.94	10.02	0.6169
'Adiponectin'	8.60	7.55	0.6169
'ALAT'	18.00	22.00	0.6318
'energy'	7338.31	11293.40	$1.11x10^{-24}$
'protein_total'	64.55	101.27	$2.71x10^{-26}$
'fat_total'	60.80	100.50	$1.23x10^{-21}$
'sat_fa'	21.48	35.25	$1.22x10^{-18}$
'carbo_total'	220.27	329.91	$7.62x10^{-21}$
'alcohol'	7.10	9.96	0.6400
'fibre_total'	19.12	28.90	$1.9x10^{-13}$
'Milk'	159.20	314.30	$9.44x10^{-9}$
'Cheese'	15.99	32.14	$1.35x10^{-5}$
'Ice-cream'	4.91	4.81	0.7950
ereal and starch foo	173.55	260.25	$3.46x10^{-12}$
'Vegetables'	111.11	128.95	0.0231
'Potatoes'	101.14	137.76	0.0065
'Fruits'	236.04	342.50	0.0080
'Meat'	83.69	124.80	$6.05x10^{-8}$
'Fish'	26.77	38.46	0.0018
'Poultry'	18.76	24.32	0.0080

'Egg'	16.84	26.44	0.0008
'Spices'	3.87	5.65	$1.62x10^{-8}$
arbohydrate rich foc	681.08	908.61	$9.44x10^{-9}$
'Protein.rich.food'	278.95	537.83	$6.58x10^{-14}$
nbined.poultry.and.	46.80	67.35	$1.00x10^{-5}$
