# Supplementary material for: Human and preclinical studies of the host–gut microbiome co-metabolite hippurate as a marker and mediator of metabolic health
Source: Gut. 2021 May 11;70(11):2105–14. doi: 10.1136/gutjnl-2020-323314 (PMC8515120; doi:10.1136/gutjnl-2020-323314)
Supplement: Supplementary data [file gutjnl-2020-323314supp021.pdf]

Supplementary Table 11. Modelling hippurate urinary concentrations in function of microbiome richness and phenylpropanoid metaolism modules, enterotype, individuals' age, gender and BMI and dietary habits. Dietary habits were encoded either as food categories (N=184), or summarized in two principal components (dietary PC; N=193). The contribution of each explanatory variable in an univariate modelling of urinary hippurate is first provided (with the prefix univariate). The best model (stepwise forward selection) summary statistics are reported next with the prefix multivariate. All continuous variables were rank-transformed.

			univariate						multivariate					
Diet encoding	Y	Parameter	SS	uEstimate	RSquare	F	P	N	Step	SS	Estimate	Cumulative RSquare	P2	N2
Diet as dietary PC	Hippurate	Richness	174912	0.31652	0.10546	31.714	4E-08	271	1	155505	0.33536	0.12945	2.8E-07	193
Diet as dietary PC	Hippurate	Dietary PC1	78975.1	-20.229	0.06574	13.4406	3E-04	193	2	45281.2	-17.047	0.16715	0.00378	193
Diet as dietary PC	Hippurate	MC0020	4369.84	0.04996	0.00263	0.71063	4E-01	271	3	36237.3	-0.1898	0.19731	0.00836	193
Diet as dietary PC	Hippurate	Bacteroides2	140330	-37.989	0.08461	24.8643	1E-06	271	4	23704	-20.654	0.21704	0.03075	193
Diet as dietary PC	Hippurate	MC0005	68048.2	0.19826	0.04103	11.5091	8E-04	271						
Diet as dietary PC	Hippurate	MC0004	61063.3	0.18695	0.03682	10.2826	2E-03	271						
Diet as dietary PC	Hippurate	BMI	35018.1	-0.1418	0.02111	5.80219	2E-02	271						
Diet as dietary PC	Hippurate	MC0002	21672.7	-0.1114	0.01307	3.5617	6E-02	271						
Diet as dietary PC	Hippurate	MC0018	21061	0.11023	0.0127	3.45988	6E-02	271						
Diet as dietary PC	Hippurate	Age	13896.6	0.97445	0.00838	2.27297	1E-01	271						
Diet as dietary PC	Hippurate	MC0017	7510.58	-0.0662	0.00453	1.2237	3E-01	271						
Diet as dietary PC	Hippurate	MC0003	4783.49	-0.0535	0.00288	0.77809	4E-01	271						
Diet as dietary PC	Hippurate	Gender	2813.25	-3.2336	0.0017	0.45706	5E-01	271						
Diet as dietary PC	Hippurate	MC0019	2444.3	0.0376	0.00147	0.39703	5E-01	271						
Diet as dietary PC	Hippurate	Dietary PC2	1184.25	-2.4771	0.00099	0.18848	7E-01	193						
Diet as dietary PC	Hippurate	MC0010	197.369	0.01065	0.00012	0.03202	9E-01	271						
Diet as food categories	Hippurate	Richness	174912	0.31652	0.10546	31.714	4E-08	271	1	148633	0.37112	0.12742	6.6E-07	184
Diet as food categories	Hippurate	Bacteroides2	140330	-37.989	0.08461	24.8643	1E-06	271	2	28206.1	-21.729	0.1516	0.02431	184
Diet as food categories	Hippurate	Gender	2813.25	-3.2336	0.0017	0.45706	5E-01	271	3	21586.1	-12.677	0.17011	0.04663	184
Diet as food categories	Hippurate	MC0020	4369.84	0.04996	0.00263	0.71063	4E-01	271	4	23549.6	-0.1836	0.1903	0.03602	184
Diet as food categories	Hippurate	MC0005	68048.2	0.19826	0.04103	11.5091	8E-04	271						
Diet as food categories	Hippurate	MC0004	61063.3	0.18695	0.03682	10.2826	2E-03	271						
Diet as food categories	Hippurate	BMI	35018.1	-0.1418	0.02111	5.80219	2E-02	271						
Diet as food categories	Hippurate	Fish	21165.3	0.39756	0.0177	3.40565	7E-02	191						
Diet as food categories	Hippurate	MC0002	21672.7	-0.1114	0.01307	3.5617	6E-02	271						
Diet as food categories	Hippurate	MC0018	21061	0.11023	0.0127	3.45988	6E-02	271						
Diet as food categories	Hippurate	Meat	13090.5	-0.1727	0.0109	2.10431	1E-01	193						
Diet as food categories	Hippurate	Age	13896.6	0.97445	0.00838	2.27297	1E-01	271						
Diet as food categories	Hippurate	Cereal and st	8771.5	0.0797	0.0073	1.40492	2E-01	193						
Diet as food categories	Hippurate	Fruits	7375.66	0.02972	0.00623	1.18562	3E-01	191						
Diet as food categories	Hippurate	Egg	6070.74	-0.2709	0.00505	0.97015	3E-01	193						
Diet as food categories	Hippurate	MC0017	7510.58	-0.0662	0.00453	1.2237	3E-01	271						
Diet as food categories	Hippurate	Milk	4940.37	-0.0207	0.00411	0.78876	4E-01	193						
Diet as food categories	Hippurate	MC0003	4783.49	-0.0535	0.00288	0.77809	4E-01	271						
Diet as food categories	Hippurate	Potatoes	3157.06	-0.045	0.00263	0.50329	5E-01	193						
Diet as food categories	Hippurate	MC0019	2444.3	0.0376	0.00147	0.39703	5E-01	271						
Diet as food categories	Hippurate	Poultry	1151.71	0.10935	0.00096	0.18177	7E-01	191						
Diet as food categories	Hippurate	Cheese	482.706	-0.0602	0.0004	0.07612	8E-01	190						
Diet as food categories	Hippurate	Vegetables	364.466	0.01337	0.0003	0.05797	8E-01	193						
Diet as food categories	Hippurate	MC0010	197.369	0.01065	0.00012	0.03202	9E-01	271						
