# Supplementary material for: Human and preclinical studies of the host–gut microbiome co-metabolite hippurate as a marker and mediator of metabolic health
Source: Gut. 2021 May 11;70(11):2105–14. doi: 10.1136/gutjnl-2020-323314 (PMC8515120; doi:10.1136/gutjnl-2020-323314)
Supplement: Supplementary data [file gutjnl-2020-323314supp023.pdf]

Supplementary Table 12. Modelling hippurate urinary concentrations in function of microbiome richness and phenylpropanoid metaolism modules, enterotype, individuals' age, gender and BMI and dietary habits. Dietary habits were encoded either as food categories (N=184), or summarized in two principal components (dietary PC; N=193). The contribution of each explanatory variable in an univariate modelling of urinary hippurate is first provided (with the prefix univariate). The best model (stepwise forward selection) summary statistics are reported next with the prefix multivariate. All continuous variables were rank-transformed.

		High PC1			Low PC1			High PC2			Low PC2		
		Median		pFDR	Median		pFDR	Median		pFDR	Median		pFDR
Bioclinical variables and units	Short name	Low Hippurate	High Hippurate		Low Hippurate	High Hippurate		Low Hippurate	High Hippurate		Low Hippurate	High Hippurate	
amination and sa	'Ageatvisit'	55.6	60.6	0.0581	55.8	55.3	0.9798	56.0	59.2	0.5988	55.5	56.3	0.9435
female (%)	'gender'	44.2	26.7		56.4	72.0		68.7	69.6		40.5	33.3	
male (%)	'gender'	55.8	73.3		43.6	28.0		31.3	30.4		59.5	66.7	
y mass index, kg	BMI'	32.0	25.2	0.0081	31.6	30.6	0.6384	31.1	25.7	0.3632	32.0	31.3	0.8798
weight in kg	'weight'	98.8	78.0	0.0199	91.5	83.7	0.6384	90.0	78.0	0.3632	97.3	97.1	0.9750
t circimference i	'waist'	108.0	94.5	0.0747	106.0	103.0	0.6384	103.0	93.0	0.4276	108.0	110.5	0.9750
circumference ir	'hip'	112.0	106.0	0.0426	112.0	111.0	0.6996	112.0	106.0	0.3971	112.0	111.0	0.8798
t percentage frc	'WBTOT_PFAT'	32.4	26.0	0.0576	34.0	34.9	0.9798	36.3	33.8	0.3971	32.5	31.2	0.9435
sma-Glucose mr	'pgluc'	5.7	5.6	0.5353	5.7	5.9	0.8156	5.6	5.6	0.9567	5.8	6.0	0.8798
rum-Insulin pmo	'insu'	59.5	29.0	0.0199	49.0	37.0	0.4531	44.0	26.0	0.0317	63.0	53.0	0.8798
C peptide	'cpep'	939.5	597.0	0.0192	796.0	603.0	0.6384	744.0	550.0	0.0829	900.0	804.0	0.9435
essment of insuli	'Homair'	2.3	1.1	0.0199	1.9	1.3	0.4531	1.6	0.9	0.0347	2.3	2.0	0.8798
a-cholesterol mi	'pchol'	5.7	5.8	0.5370	5.4	5.5	0.8286	5.5	5.6	0.6890	5.6	5.5	0.9750
HDL-Cholesterol	'hdl'	1.3	1.4	0.3621	1.4	1.5	0.9342	1.4	1.6	0.3971	1.4	1.4	0.9750
a-Triglyceride n	'tri'	1.4	1.1	0.0426	1.1	1.2	0.9798	1.1	1.0	0.3971	1.3	1.3	0.9750
fatty acids (mr	'FFA'	0.5	0.4	0.1673	0.5	0.5	0.9342	0.5	0.5	0.8100	0.5	0.4	0.9750
eucocytes count	'wbc'	6.0	5.6	0.2862	5.9	5.7	1.0000	5.8	5.2	0.3971	6.0	6.3	0.9435
mphcytes count	'lymph'	1.9	1.7	0.4743	1.9	1.8	0.9798	1.8	1.7	0.5877	1.9	2.0	0.8798
active protein (r	'CRP'	2.2	1.0	0.0573	1.5	2.1	0.8156	1.6	1.3	0.6072	1.8	2.2	0.9750
terleukine 6 (ng	'IL6'	17.2	10.5	0.1673	16.4	11.8	0.6542	12.6	17.0	0.8870	20.2	11.6	0.8798
ecrosis factor al	'TNFalfa'	12.7	2.0	0.3212	23.9	0.6	0.2134	16.8	0.0	0.0931	28.3	3.0	0.8798
ed adipose facto	'FIAF'	83.3	63.7	0.0574	81.2	90.3	0.5284	83.5	83.8	0.9889	82.9	75.3	0.9750
ptin (microgram	'leptin'	11.3	5.6	0.0426	11.3	10.9	0.9798	13.0	9.0	0.3971	11.3	9.2	0.8798
diponectin (mg/	'Adiponectin'	7.3	9.7	0.2048	8.3	11.6	0.4531	9.6	11.6	0.3971	7.2	8.0	0.9138
amino transfera	'ALAT'	22.0	14.0	0.4746	19.5	15.0	0.2134	21.0	14.0	0.0235	21.0	22.5	0.9750
Energy (KJ)	'energy'	11386.1	11293.4	0.9580	7347.2	7329.4	0.8857	8787.5	8060.2	0.5182	8169.3	9300.8	0.4391
Protein, total (g)	'protein_total'	97.8	103.5	0.9580	64.1	65.4	0.7773	78.9	67.7	0.5182	68.4	81.2	0.4391
Fat, total (g)	'fat_total'	100.7	99.7	0.9580	61.5	57.5	0.8857	69.3	57.8	0.5964	75.4	88.2	0.4391
Saturated fat (g)	'sat_fa'	34.7	36.5	0.9580	22.3	18.7	0.3461	22.9	18.7	0.5182	27.8	33.3	0.4391
bohydrate, tota	'carbo_total'	332.6	328.6	0.9580	218.6	234.2	0.7773	278.0	277.7	0.5964	222.8	262.3	0.4887
alcohol (g)	'alcohol'	9.8	16.5	0.6733	8.3	4.6	0.4755	7.2	8.0	0.5964	9.7	3.8	0.4391
fibre_total (g)	'fibre_total'	29.3	27.5	0.9580	18.9	21.8	0.3570	26.8	27.1	0.8171	17.6	20.4	0.4391
Milk (g/day)	'Milk'	314.5	308.0	0.9580	149.3	170.6	0.8857	198.4	190.0	0.7881	174.8	206.5	0.9471
Cheese (g/day)	'Cheese'	24.1	48.3	0.9580	17.3	12.5	0.4014	19.7	11.9	0.5182	20.4	29.3	0.4391
ce cream (g/day	'Ice-cream'	4.8	4.8	0.9580	5.1	4.8	0.4755	5.5	4.8	0.5182	4.8	4.8	0.9471
and starch food	eal and starch fo	241.9	310.5	0.4482	174.2	164.2	0.8857	188.8	178.0	0.8171	189.0	215.5	0.4391
egetables (g/day	'Vegetables'	133.6	118.8	0.9580	107.3	132.0	0.3461	156.8	161.8	0.8171	95.6	98.9	0.9471
Potatoes (g/day)	'Potatoes'	127.7	192.2	0.4482	111.5	68.4	0.3461	95.2	77.1	0.6819	126.7	132.7	0.9471
Fruits (g/day)	'Fruits'	360.1	329.9	0.8937	184.9	351.5	0.3461	483.6	436.5	0.5182	125.5	128.9	0.9471

Meat (g/day)	'Meat'	123.5	147.0	0.9580	85.3	74.7	0.6998	83.7	74.6	0.5182	107.1	127.5	0.4391
Fish (g/day)	'Fish'	33.0	63.0	0.1031	26.4	27.2	0.8857	31.4	45.2	0.5182	27.1	28.2	0.9471
Poultry (g/day)	'Poultry'	25.0	22.6	0.9580	17.6	25.6	0.3570	23.9	27.6	0.7087	18.0	17.7	0.9471
Egg (g/day)	'Egg'	26.7	23.7	0.9580	17.7	15.1	0.7773	19.8	15.3	0.5182	19.6	19.6	0.9471
Spices (g/day)	'Spices'	5.6	5.8	0.9580	3.9	4.0	0.7773	3.9	4.4	0.7881	4.7	4.7	0.9471
ydrate rich food	bohydrate rich f	913.7	908.6	0.9580	674.5	758.0	0.4755	951.7	908.6	0.6949	587.7	629.9	0.9471
tein rich food (g/	Protein.rich.fooc	547.0	535.2	0.9580	279.5	278.4	0.8825	335.0	295.2	0.5182	357.8	388.7	0.9471
d poultry and fis	ined.poultry.an	61.0	85.0	0.5475	46.4	56.2	0.4485	57.6	71.4	0.5182	45.4	53.8	0.9471
